# Genome-wide SNP and InDel analysis of three Philippine mango species inferred from whole-genome sequencing

**DOI:** 10.1186/s43141-022-00326-3

**Published:** 2022-03-11

**Authors:** Cris Q. Cortaga, John Albert P. Lachica, Darlon V. Lantican, Eureka Teresa M. Ocampo

**Affiliations:** 1 Institute of Crop Science, College of Agriculture and Food Science, University of the Philippines Los Baños, College, 4031 Laguna, Philippines; 2 Institute of Plant Breeding, College of Agriculture and Food Science, University of the Philippines Los Baños, College, 4031 Laguna, Philippines

**Keywords:** Philippine mango, Whole-genome sequencing, SNPs, InDels, Variants

## Abstract

**Background:**

The Philippines is among the top 10 major exporters of mango worldwide. However, genomic studies of Philippine mangoes remain largely unexplored and lacking. Here, we sequenced the whole genome of the three Philippine mango species, namely, *Mangifera odorata* (Huani), *Mangifera altissima* (Paho), and *Mangifera indica* “Carabao” variety using Illumina HiSeq 2500, to identify and analyze their genome-wide variants (SNPs and InDels).

**Results:**

The high confidence variants were identified by successfully mapping 93–95% of the quality-filtered reads to the Alphonso and Tommy Atkins mango reference genomes. Using these two currently available mango genomes, most variants were observed in *M. odorata* (4,353,063 and 4,277,287), followed by *M. altissima* (3,392,763 and 3,449,917), and lastly, *M. indica* Carabao (2,755,267 and 2,852,480). Approximately 50, 46, and 38% of the variants were unique in the three Philippine mango genomes. The analysis of variant effects and functional annotation across the three mango species revealed 56,982 variants with high-impact effects mapped onto 37,746 genes, of which 25% were found to be novel. The affected mango genes include those with potential economic importance such as 6945 genes for defense/resistance/immune response, 323 genes for fruit development, and 338 genes for anthocyanin production.

**Conclusions:**

To date, this is the first sequencing effort to comprehensively analyze genome-wide variants essential for the development of genome-wide markers specific to these mango species native to the Philippines. This study provides an important genomic resource that can be used for the genetic improvement of mangoes.

**Supplementary Information:**

The online version contains supplementary material available at 10.1186/s43141-022-00326-3.

## Background

The Philippines is among the top 10 major exporters of fresh and dried mangoes in the world. The country’s mango export is valued at USD 91 million and contributes a 4% share of the global market [1, 2]. The mango industry also supports about 2.5 M Filipino farmers [3]. In the first quarter of 2021, 97.9 thousand Mt of mangoes were produced in the Philippines and around 83% of which came from the Carabao mango variety (*Mangifera indica*) [4]. The Carabao mango is the Philippines’ export variety which is known as one of the world’s finest, superior quality, and sweetest mango varieties. Hence, Carabao is the country’s flagship variety in the mango global value chain.

Mango belongs to the kingdom Plantae, order Sapindales, family Anacardiaceae (cashew family), subfamily Anacardioideae, and genus *Mangifera*. *Mangifera indica*, the common mango, is a juicy drupe that is usually found in tropical countries. It has varying sweetness and texture across cultivars and has a high incidence of hybridization with other members of its genus. This results in new varieties or species such as *Mangifera odorata* (*M. indica* x *M. foetida*) which is commonly known as Huani in the Philippines [5]. Huani is also known for its characteristic pungent smell and taste of turpentine. Another native species of mango in the country is *Mangifera altissima* which is locally known as Paho. Its unripe fruits are small and oftentimes used in salads in the Philippines.

Mango has a diploid chromosome (*n*=20 chromosomes), and its haploid genome size is relatively small (approximately 400 Mb) but complex due to its innate heterozygosity [6, 7]. The mango seed exhibits apomixis and can produce one seedling (monoembryony) or multiple seedlings (polyembryony) in one seed. The former is common in varieties originating from India and mainland China [8] while the latter is observed in varieties that evolved in places closer to the equator such as the Philippines [9]. The complex (heterozygous) genome and polyembryonic nature of mango in the Philippines pose a significant challenge in genomics and plant breeding studies. Hence, despite the agricultural and economical importance of mango in the country, the genomic studies of Philippine mangoes remain lacking and largely unexplored.

Recently, the chromosome-level whole-genome sequencing (WGS) of Alphonso [7] and Tommy Atkins [6] was completed, providing high-quality reference genomes for mango. Both varieties are of the same species (*M. indica*) and are important varieties in the mango international trade. With the availability of WGS data, in-depth genome analysis can be performed to unravel gene networks, reveal intron-exon boundaries, detect transposable elements, discover novel biological processes, develop molecular markers tagging economically important traits for breeding (e.g., insect pest and disease resistance), and identify genome-wide variants such as single-nucleotide polymorphisms (SNPs) and insertions-deletions (InDels) [10–14]. SNPs and InDels are differences and variations in the genome which can have a huge impact on the biological and physical traits of an organism.

In this study, we sequenced the whole genomes of three Philippine mango species, namely, *Mangifera odorata* (Huani), *Mangifera altissima* (Paho), and *Mangifera indica* ‘Carabao’ using Illumina HiSeq, to identify and characterize their genome-wide variants (SNPs and InDels). The high confidence variants were identified by successfully mapping the quality-filtered reads to the Alphonso and Tommy Atkins mango reference genomes. This study provides valuable information and resources for mango breeding and genetic studies.

## Methods

### Mango species used and DNA extraction

Three mango species native to the Philippines were used in this study, namely, *Mangifera indica* Carabao, *Mangifera odorata* (Huani), and *Mangifera altissima* (Paho). A high-quality DNA was extracted from three mango trees of the same species using the method of Inglis et al. [15] with modifications. Fresh, young leaves of mango were cut into small pieces (excluding the midrib and leaf veins) and then pulverized using liquid nitrogen for 20 s (2 to 3 cycles). About 150 g of the pulverized tissues was transferred to a microcentrifuge tube and then pre-washed by adding a sorbitol solution pre-added with 2-mercaptoethanol (1% v/v). The tube was centrifuged at 12,000 rpm for 5 min, then the supernatant was discarded. The pulverized tissues were lysed by adding 700 μL of CTAB in the tube, vortexed for 5 s, then heated at 65 °C for 1 h with inversion of the tube every 10 min. The tube was then left at room temperature for 10 min, and 700 μL of 24:1 chloroform to isoamyl alcohol solution (CIA) was added to separate the cellular components. The tube was vortexed for 10 s, then centrifuged at 12,000 rpm for 5 min. Afterwards, the supernatant was transferred to a new tube and 10% of 3M sodium acetic acid and ice-cold isopropanol (2x volume) were added. The tube was incubated for 1 h at −20 °C, then centrifuged at 10,000 rpm for 10 min. The supernatant was discarded, and the pellet (DNA) was washed with 1 mL of ice-cold 70% ethanol, then centrifuged at 10,000 rpm for 10 min. The ethanol was carefully removed, and the pellet was air-dried for 1 h and resuspended by adding 100 μL of Tris-EDTA (pre-added with RNAse). Afterwards, the tube was incubated at 37 °C for 30 min and then stored at −20 °C.

The quality of DNA was checked via gel electrophoresis using 1.5% agarose with SYBR Safe nucleic acid stain (Life Technologies Corporation, USA) and viewed using a gel documentation system (Gel Doc 1000, Bio-Rad Laboratories, USA). DNA samples showing bands were further checked using Epoch Microplate Spectrophotometer and fluorometer (DeNovix QFX Fluorometer), to ensure high-quality DNA that is amenable for the next-generation sequencing.

### Whole-genome sequencing

The extracted high-quality DNA from three mango species were submitted for sequencing using the Illumina HiSeq 2500 platform (Macrogen, Korea) with a sequencing coverage of 1X per sample. Three DNA samples were sequenced per mango species. The raw reads of all samples were deposited in the NCBI under the BioProject number PRJNA740276.

### Pre-processing of short reads

The low-quality base score sequences and adapter sequences from raw reads produced by Illumina HiSeq 2500 sequencing (short reads) were removed using Trimmomatic v0.36 [16] following these parameters: SLIDINGWINDOW:4:25, LEADING:3, TRAILING:3, MINLEN:75. The trimmed reads were subsequently evaluated for quality using the FastQC toolkit [17].

### Mapping of pre-processed short reads

The pre-processed paired sequences of three samples per mango species were concatenated and then mapped to the recently published mango reference genomes of Alphonso [7] (BioProject PRJNA487154) and Tommy Atkins [6] (BioProject PRJNA450143) using Burrows-Wheeler Aligner tool (BWA) [18]. The bwa index and bwa mem commands were used for indexing of reference genomes and alignment of short reads, respectively. The sequence alignment map (SAM) produced was used to count the mapped reads and determine the alignment rate of short reads to the reference genomes using SAMtools [19] and BamTools [20], respectively.

### Variant calling

Using the SAM file from the read mapping step as input, an analysis-ready binary alignment map (BAM) file was generated using the Picard tools [21] following the SortSAM, FixMateInformation, MarkDuplicates, and AddOrReplaceReadGroups commands. The reference genome was indexed using the SAMtools faidx command and a sequence dictionary was created using the CreateSequenceDictionary command of Picard tools. Variants (such as SNPs and InDels) between the three Philippine mango species and reference genomes of Alphonso and Tommy Atkins were detected following the Genome Analysis Toolkit (GATK) Best Practices workflow [22]. The read mapping artifacts were minimized through local realignment around InDels by using the RealignerTargetCreator and IndelRealigner commands. Variants were called using the HaplotypeCaller command by setting the output mode to EMIT_VARIANTS_ONLY and calling the confidence threshold (stand_call_conf) to 20. The raw variant call format (VCF) file produced was filtered using the VariantFiltration command following the recommended parameters for SNPs and InDels. Using the SelectVariants -ef command, only the SNPs and InDels that pass the first filtering were printed and considered in the new VCF output. Then, base quality score recalibration was performed using BaseRecalibrator and PrintReads commands, to correct the bias of the per-base estimate of error generated by the sequencing platform. Afterwards, the second round of variant calling and filtering using the HaplotypeCaller and VariantFiltration commands, respectively, was performed to identify high-confidence SNPs and InDels. The final VCFs containing high confidence variants were then used as input to CircosVCF [23] for visualization of variant density in circos plots. The VCFtools [24] was used to create an InDel histogram.

### Variant effects, phylogenetic relationship, and kinship analysis

The generated VCFs of the three mango species were analyzed for variant effects on the gene regions using the SnpEff toolbox [25]. The SnpEff functional classes detected in all SNPs and InDels were 3′ and 5′ untranslated region (UTR) variant; downstream and upstream gene variant; intergenic region; intragenic variant; intron variant; splice acceptor, splice donor, and splice region variant; start lost and start retained variant; and stop gained, stop lost, and stop retained variant. The functional classes detected only for SNPs were 5′ UTR premature start codon gain variant, initiator codon variant, missense variant, and synonymous variant. Meanwhile, the functional classes detected only for InDels include 3′ and 5′ UTR truncation, bidirectional gene fusion, conservative inframe insertion and deletion, disruptive inframe insertion and deletion, exon loss variant, frameshift variant, and non-coding transcript variant. Other important information provided by SnpEff are the variant rate details (per chromosome), variant types, base changes for SNPs including transitions (Ts) and transversions (Tv) ratio, allele data, and variant effects by impact which are classified as high, moderate, low, and modifier. Only the SNPs and InDels identified as high impact were considered for further analysis. The generated VCFs were also used to construct a UPGMA phylogenetic tree using VCF2PopTree [26] as well as for kinship analysis using the vcf2kinship command of Rvtests [27] following the identity-by-state (IBS) model.

### Gene ontology (GO), GO enrichment, and KEGG analyses of high-impact variants

The protein sequences of gene IDs identified as high impact were retrieved and Gene Ontology (GO) analysis was performed using the BLAST2GO package [28]. The homology of the protein sequences was determined using the UniProtKB/SwissProt protein database via BLASTp analysis (with an *e* value of 1e–3). The BLAST results were then mapped and annotated to produce the GO annotations from the three domains of molecular function (MF), biological processes (BP), and cellular component (CC) assigned to each protein sequence. GO enrichment analysis of biological processes was performed using agriGO [29, 30]. The hypergeometric statistical test method and Yekutieli multi-test adjustment method [with False Discovery Rate (FDR) under dependency] were the parameters used for the analysis. The significance level was set at *P* < 0.05. KEGG analysis [31] was also performed using the single-directional best hit method and BLAST search program with representative data set for eukaryotes.

## Results

### Mapping of reads to the reference genomes

Trimming/filtering of the raw sequences produced a total of 22.8 million reads for *M. odorata* (Huani), 20.7 million reads for *M. altissima* (Paho), and 18.9 million reads for *M. indica* Carabao (Table 1). These were used for alignment and mapping to the two reference genomes from Alphonso and Tommy Atkins varieties. A total of 21.7 million (95.07%), 19.3 million (93.39%), and 17.8 million (94.46%) high-quality-filtered reads of *M. odorata*, *M. altissima*, and *M. indica* Carabao, respectively, were successfully mapped to the Alphonso reference genome with sequencing coverage of 4.30, 3.98, and 3.55X, respectively (Table 1). Meanwhile, 21.6 million (94.71%), 19.3 million (93.14%), and 17.8 million (93.99%) high-quality-filtered reads of *M. odorata*, *M. altissima*, and *M. indica* Carabao, were successfully mapped to the Tommy Atkins reference genome with sequencing coverage of 3.53, 3.26, and 2.91X, respectively (Table 1).

**Table 1:** Mapping of sequences (short reads) of three Philippine mango species to Alphonso and Tommy Atkins mango reference genomes

Reference genome	Mapping statistics	Huani^a^	Paho^b^	Carabao^c^
Alphonso	Total reads	22,856,937	20,769,897	18,930,358
	Mapped reads	21,728,943 (95.07%)	19,397,174 (93.39%)	17,881,109 (94.46%)
	Properly paired	19,974,549 (87.39%)	17,539,532 (84.45%)	16,351,261 (86.38%)
	Singletons	352,354 (1.54%)	454,708 (2.20%)	330,257 (1.74%)
	Sequencing coverage	4.30	3.98	3.55
Tommy Atkins	Total reads	22,869,467	20,785,198	18,945,242
	Mapped reads	21,660,395 (94.71%)	19,359,194 (93.14%)	17,806,147 (93.99%)
	Properly paired	19,711,973 (86.19%)	17,302,309 (83.24%)	16,061,097 (84.78%)
	Singletons	391,435 (1.71%)	482,123 (2.32%)	368,930 (1.95%)
	Sequencing coverage	3.53	3.26	2.91

^a^*M. odorata*, ^b^*M. altissima*, ^c^*M. indica*

### Identification of SNPs and InDels

By mapping the reads to the Alphonso genome (Table 2), 4,353,063 variants were detected in *M. odorata*. This comprised of 3,826,194 SNPs and 526,869 InDels with an average variant rate of one SNP every 93 bases and one InDel every 678 bases. In *M. altissima*, 3,392,763 variants were found comprising of 2,918,359 SNPs and 474,404 InDels with an average variant rate of one SNP every 122 bases and one InDel every 753 bases. In *M. indica* Carabao, 2,755,267 variants were detected consisting of 2,355,481 SNPs and 399,786 InDels with an average variant rate of one SNP every 151 bases and one InDel every 894 bases. Meanwhile, when the reads were mapped to the Tommy Atkins genome (Table 2), a total of 4,277,287 variants were found in *M. odorata*. This consisted of 3,777,813 SNPs and 499,474 InDels with an average variant rate of one SNP every 99 bases and one InDel every 755 bases. For *M. altissima*, 3,449,917 variants were detected comprising of 2,990,377 SNPs and 459,540 InDels with an average variant rate of one SNP every 126 bases and one InDel every 821 bases. *M. indica* Carabao had a total of 2,852,480 variants which include 2,448,630 SNPs and 403,850 InDels, with an average variant rate of one SNP every 154 bases and one InDel every 934 bases.

**Table 2:** Number of SNPs and InDels identified in three Philippine mango species

Reference genome	Variant	Huani^a^	Paho^b^	Carabao^c^
Alphonso	SNPs	3,826,194	2,918,359	2,355,481
	Insertions	261,867	236,119	199,857
	Deletions	265,002	238,285	199,929
	Total	4,353,063	3,392,763	2,755,267
Tommy Atkins	SNPs	3,777,813	2,990,377	2,448,630
	Insertions	242,643	223,650	197,129
	Deletions	256,831	235,890	206,721
	Total	4,277,287	3,449,917	2,852,480

^a^*M. odorata*, ^b^*M. altissima*, ^c^*M. indica*

### Distribution of SNPs and InDels

**Fig. 1:**
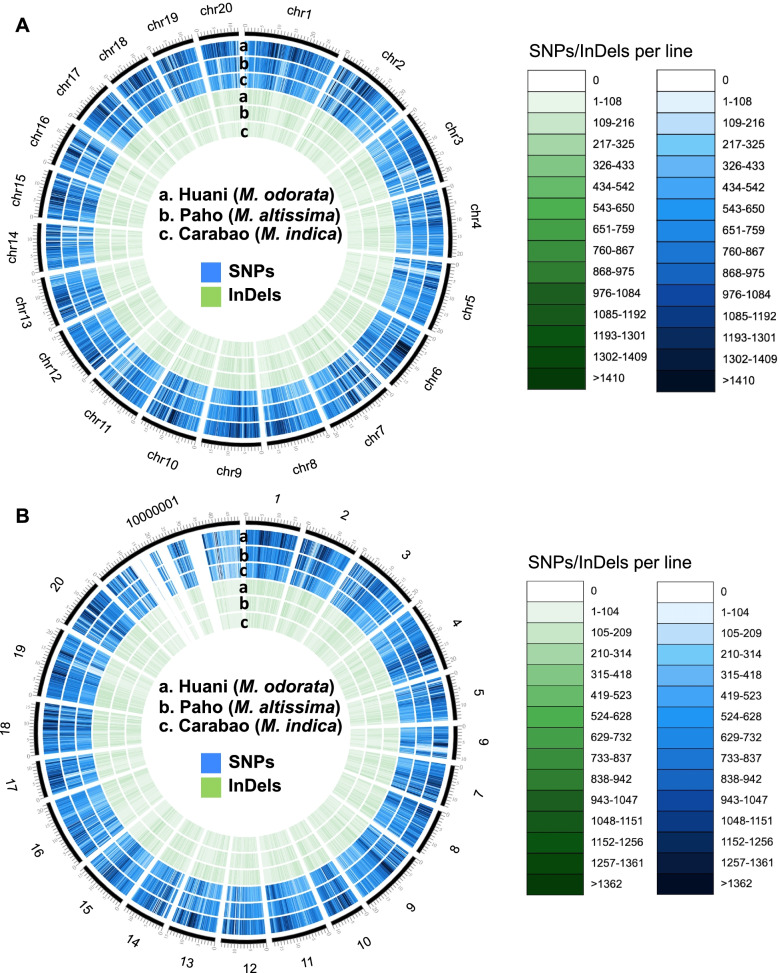
Density of SNPs and InDels in the chromosomes (*n*=20) of three Philippine mango species using Alphonso (**a**) and Tommy Atkins (**b**) reference genomes

**Fig. 2:**
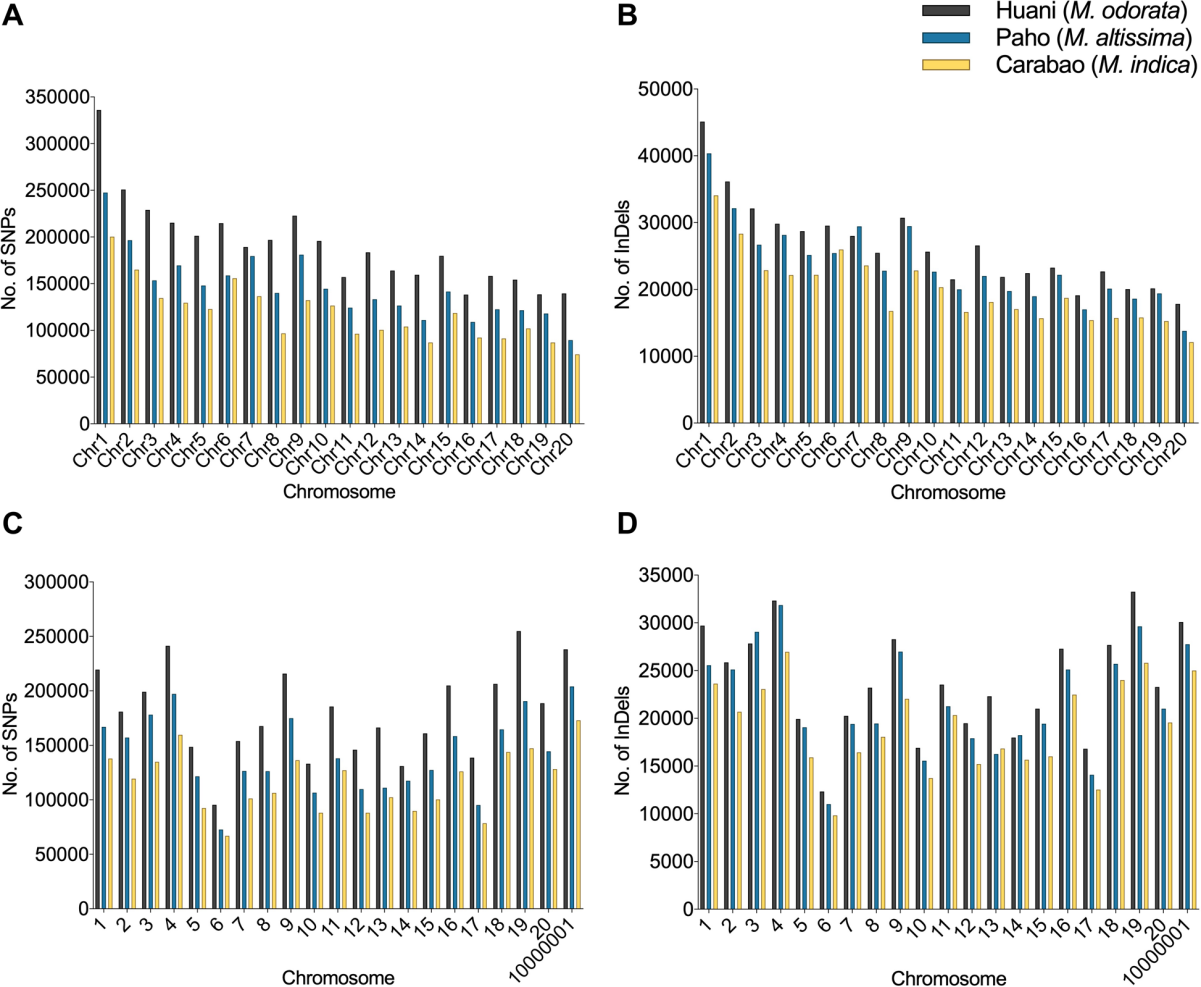
Frequency of SNPs and InDels in the chromosomes (*n*=20) of the three Philippine mango species using Alphonso (**a** and **b**, respectively) and Tommy Atkins (**c** and **d**, respectively) reference genomes

**Fig. 3:**
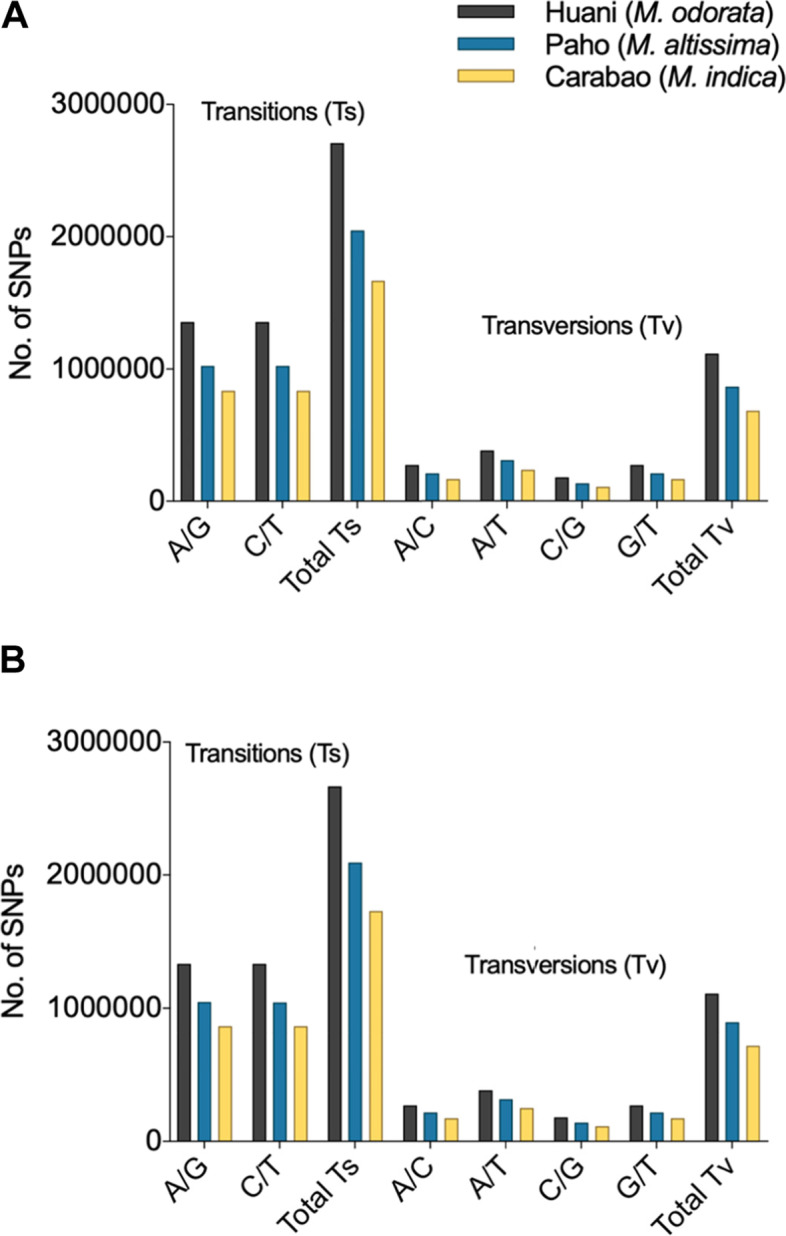
Transition (Ts) and transversion (Tv) substitutions in SNPs in the three Philippine mango species using Alphonso (**a**) and Tommy Atkins (**b**) reference genomes

**Fig. 4:**
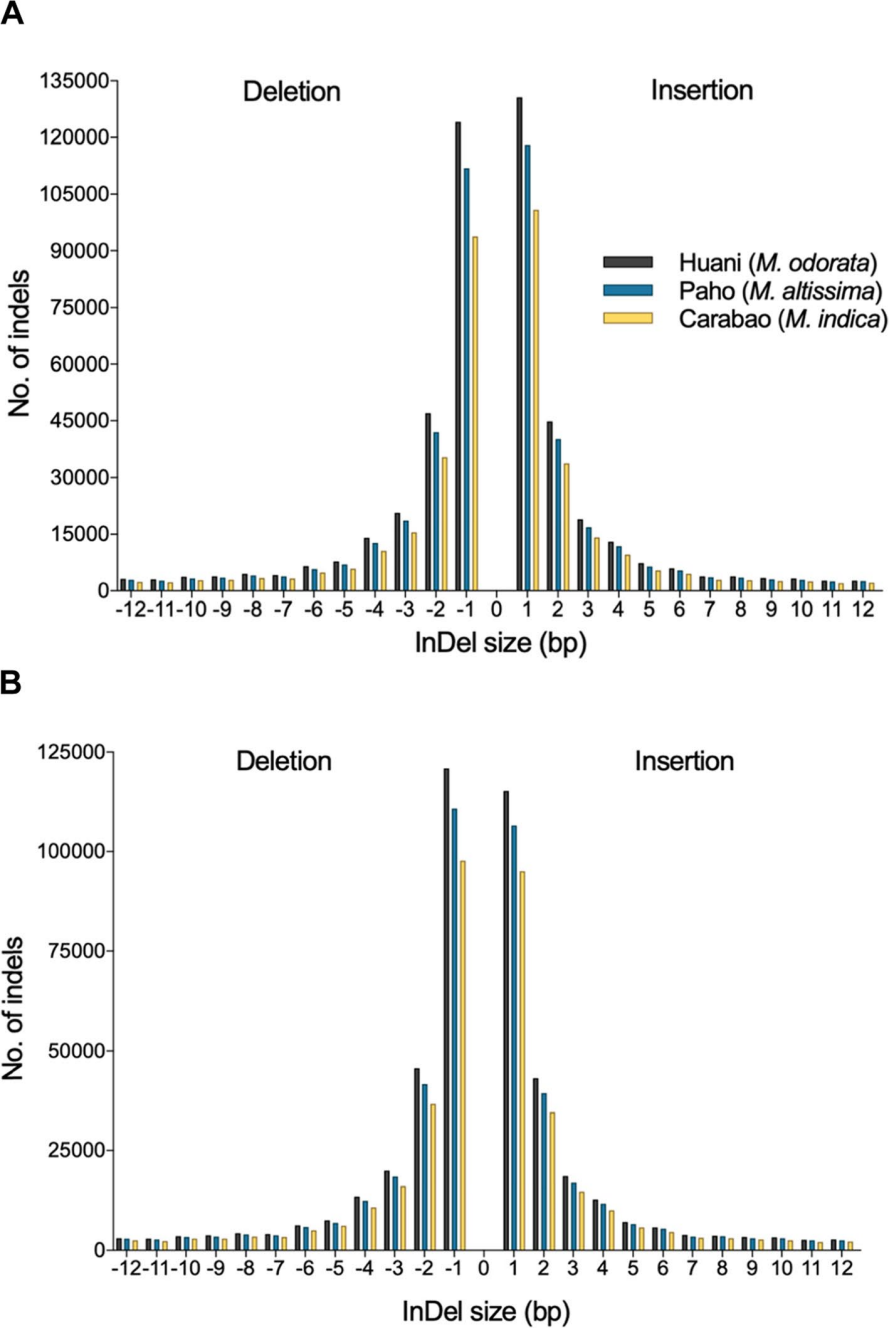
Distribution of InDels in the three Philippine mango species using Alphonso (**a**) and Tommy Atkins (**b**) reference genomes

The density and frequency of SNPs and InDels in mango chromosomes (*n*=20) are presented in Figs. 1 and 2. The Alphonso variety has a decreasing chromosome size; thus, higher SNPs and InDels were observed in chromosome 1 and lowest at chromosome 20 in all mango species used (Fig. 2a, b). On the other hand, for the Tommy Atkins genome, a non-uniform distribution of SNP and InDels across the 20 mango chromosomes was observed in all mango species analyzed (Fig. 2c, d). Chromosome 6 showed the least number of SNPs and InDels as this is the smallest chromosome in Tommy Atkins. The SNPs were generally highest in *M. odorata* and lowest in *M. indica* Carabao (Figs. 1 and 2). The detected nucleotide substitutions in the SNPs are classified as transitions (Ts) which involve A/G and C/T substitution, and transversions (Tv) which include A/C, A/T, C/G, and G/T substitutions (Fig. 3). In the Philippine mangoes studied, Ts substitution was the most abundant (70%) compared to Tv substitution (30%) regardless of the reference genome used. With this, the Ts/Tv ratios of the three mango species used ranged from 2.33 to 2.43 upon mapping to the Alphonso and Tommy Atkins genome. In Ts, the number of A/G is almost equal to the C/T type in each mango species, while for Tv, A/T substitution was the highest comprising 35–36% of Tv substitutions (Fig. 3). Similar to SNPs, InDels were also highest in *M. odorata* and lowest in *M. indica* Carabao (Figs. 1 and 2). The predominant length of InDels ranged from 1 to 12 bp which accounts for around 92% of the total number of InDels, of which 48% were mononucleotide InDels (Fig. 4).

### Shared and unique SNPs and InDels

**Fig. 5:**
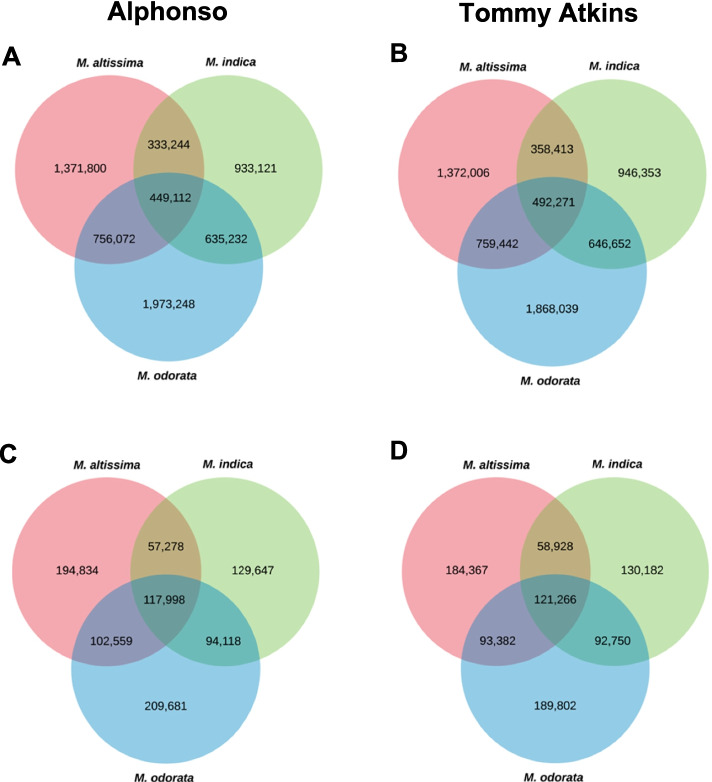
Number of shared and unique SNPs (**a** and **b**) and InDels (**c** and **d**) among the three Philippine mango species using Alphonso and Tommy Atkins reference genomes

The three mango species shared 449,112 and 492,271 SNPs relative to the Alphonso and Tommy Atkins reference genomes, respectively (Fig. 5a, b). Likewise, the three species shared 117,998 and 121,266 InDels based on the two reference genomes (Fig. 5c, d). Meanwhile, 1,973,248 (51.57%), 1,371,800 (47%), 933,121 (39.61%) SNPs and 209,681 (39.80%), 194,834 (41.07%), and 129,647 (32.43%) InDels were unique to *M. odorata*, *M. altissima*, and *M. indica* Carabao, respectively, upon mapping to the Alphonso reference genome (Fig. 5a, c). On the other hand, 1,868,039 (49.45%), 1,372,006 (45.88%), and 946,353 (38.65%) SNPs and 189,802 (38%), 184,367 (40.12%), and 130,182 (32.24%) InDels were unique to *M. odorata*, *M. altissima*, and *M. indica* Carabao, respectively, upon mapping to the Tommy Atkins reference genome (Fig. 5b and d).

### Analysis of variant effects

Analysis of the functional classes of identified SNPs are shown in Table 3. Majority of the SNPs observed were in the intergenic (14,016,127; 76.52%) and intronic (3,305,720; 18.05%) regions, and 9,020,409 (49.25%) and 8,384,036 (45.77%) SNPs were positioned in the upstream and downstream regions from the genes set, respectively. Meanwhile, 569,575 (3.11%) SNPs were missense variants. SNPs such as 3′/5′ UTR variants (362,213), initiator codon variants (162), intragenic variants (219), splice variants (91,281), start lost/retained variants (1196), stop gained/lost/retained variants (12,945), and many synonymous variants (414,577) were also detected. For the functional classes of InDels (Table 4), most of the InDels observed were also identified in the intergenic (2,126,610; 76.94%) and intronic (564,396; 20.42%) regions, and 1,641,997 (59.41%) and 1,466,121 (53.04%) InDels were positioned in the upstream and downstream regions from the genes set, respectively. Meanwhile, 34,917 (1.26%) of InDels were frameshift variants. InDels such as 3′/5′ UTR variants (73,304), bidirectional gene fusion (16), conservative inframe InDel (7,465), disruptive inframe InDel (10,680), exon loss variant (19), intragenic variant (69), non-coding transcript variant (950), splice variants (16,620), start lost/retained variants (657), and stop gained/lost/retained variants (1755) were also detected. The complete SnpEff results are provided in Supplemental Files 1A (Alphonso) and 1B (Tommy Atkins).

**Table 3:** Functional annotation of the detected SNP variants in three Philippine mango species

Type	Alphonso genome			Tommy Atkins genome		
	Huani^a^	Paho^b^	Carabao^c^	Huani^a^	Paho^b^	Carabao^c^
3 prime UTR variant	34,712	26,993	21,424	58,036	46,735	36,749
5 prime UTR premature start codon gain variant	3,076	2,351	1,821	4,850	3,751	2,897
5 prime UTR variant	19,305	14,370	11,386	30,836	24,082	18,839
Downstream gene variant	1,903,377	1,501,470	1,139,595	1,570,171	1,300,734	968,689
Initiator codon variant	30	24	18	32	33	25
Intergenic region	3,033,446	2,310,351	1,862,224	2,801,150	2,196,966	1,811,990
Intragenic variant				104	80	35
Intron variant	528,400	412,723	327,832	823,307	675,164	538,294
Missense variant	125,037	91,959	79,309	111,102	88,532	73,636
Splice acceptor variant	643	494	411	731	580	491
Splice donor variant	545	406	345	711	584	486
Splice region variant	16,351	12,542	10,269	18,521	14,905	12,266
Start lost	302	214	207	183	147	113
Start retained variant	13	9	8			
Stop gained	2,601	1,887	1,651	1,630	1,393	1,055
Stop lost	492	339	304	309	231	190
Stop retained variant	191	154	140	143	133	102
Synonymous variant	87,448	63,950	54,816	86,133	66,813	55,417
Upstream gene variant	2,040,834	1,627,526	1,203,524	1,702,492	1,419,809	1,026,224

^a^*M. odorata*, ^b^*M. altissima*, ^c^*M. indica*

**Table 4:** Functional annotation of the detected InDel variants in three Philippine mango species

Type	Alphonso genome			Tommy Atkins genome		
	Huani^a^	Paho^b^	Carabao^c^	Huani^a^	Paho^b^	Carabao^c^
3 prime UTR truncation					1	
3 prime UTR variant	6,098	5,528	4,744	10,397	9,645	8,874
5 prime UTR truncation		3	3	2		3
5 prime UTR variant	3,913	3,489	2,987	6,417	5,935	5,265
Bidirectional gene fusion	3	1	1	5	2	4
Conservative inframe deletion	708	579	550	587	489	502
Conservative inframe insertion	796	746	643	664	637	564
Disruptive inframe deletion	1,266	1,086	1,026	1,114	947	907
Disruptive inframe insertion	842	706	682	743	692	669
Downstream gene variant	299,752	273,125	225,389	244,707	228,871	194,277
Exon loss variant		4	3	5	3	4
Frameshift variant	7,571	6,757	6,054	5,307	4,857	4,371
Intergenic region	422,962	380,721	318,804	369,574	338,484	296,065
Intragenic variant	2	4	4	21	21	17
Intron variant	83,767	75,690	65,064	123,064	114,750	102,061
Non-coding transcript variant	118	98	74	267	209	184
Splice acceptor variant	151	147	129	229	221	177
Splice donor variant	252	187	178	287	244	240
Splice region variant	2,412	2,078	1,851	2,815	2,647	2,375
Start lost	112	108	90	104	94	93
Start retained variant	11	9	7	9	8	12
Stop gained	242	212	188	198	192	170
Stop lost	96	80	88	75	76	70
Stop retained variant	11	13	8	11	12	13
Upstream gene variant	335,143	307,696	249,591	275,331	258,779	215,457

^a^*M. odorata*, ^b^*M. altissima*, ^c^*M. indica*

### GO analysis and annotation of high-impact variants

**Fig. 6:**
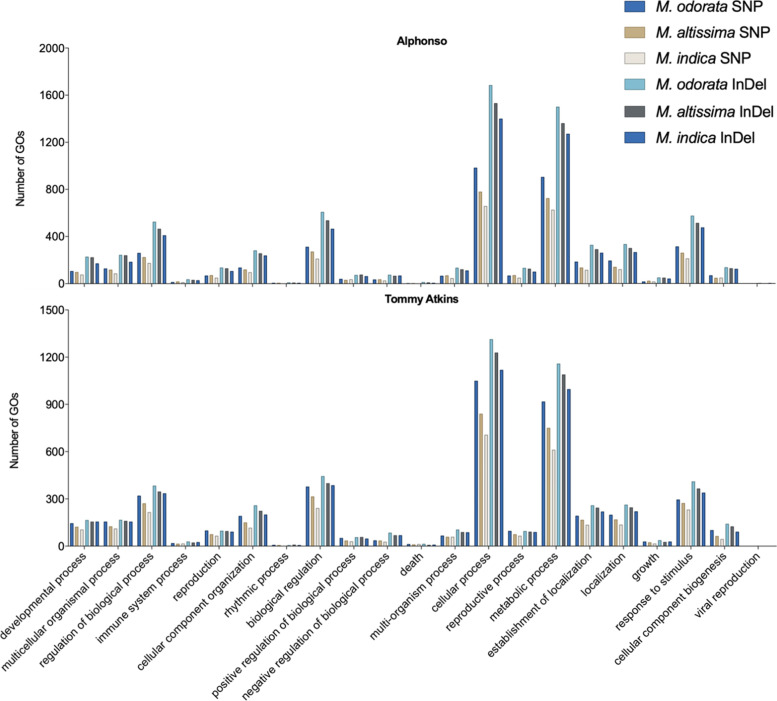
Gene Ontology (GO) enrichment analysis of high-impact variants from the three Philippine mango species

The SNPs and InDels with high-impact effects were functionally annotated and used for GO enrichment analysis. A total of 21 GO-enriched terms for biological process (GO:0008150) were detected in the genes with high-impact variants (Supplemental File 2). GO enrichment analysis showed that regulation of biological processes (GO:0050789), biological regulation (GO:0065007), response to stimulus (GO:0050896), and most especially, cellular process (GO:0009987) and metabolic process (GO:0008152) were the highly enriched biological processes in the three mango species (Fig. 6 and Supplemental File 2). In this study, a total of 56,982 high-impact variants were identified and mapped onto 37,746 genes across the three mango species (Supplemental Table 1). Around 75% (28,337) of these genes containing high-impact variants were well-known, while 25% (9409) remain unknown (Supplemental Table 1). Among the high-impact variants found in well-annotated genes include those with potential economic importance and useful for breeding, i.e., 6945 genes for defense/resistance/immune response to insects and pathogens, 323 genes for fruit development, and 338 genes for anthocyanin production found across the Philippine mango species studied (Table 5). The complete GO enrichment analysis (with FDR values) is provided in Supplemental File 2, and the complete functional annotation of genes with high-impact variants is provided in Supplemental Files 3A (Alphonso) and 3B (Tommy Atkins).

**Table 5:** Number of selected genes with potential economic importance from high-impact variants in three Philippine mango species

Reference genome	Species	Defense/ resistance/ immune response	Fruit development	Anthocyanin production
Alphonso	Huani (*M. odorata*)	1,331	65	60
	Paho (*M. altissima*)	1,289	56	49
	Carabao (*M. indica*)	1,105	49	55
Tommy Atkins	Huani (*M. odorata*)	1,151	59	69
	Paho (*M. altissima*)	1,102	48	53
	Carabao (*M. indica*)	967	46	52

### Analysis of shared and unique genes with high impact variant effects

**Fig. 7:**
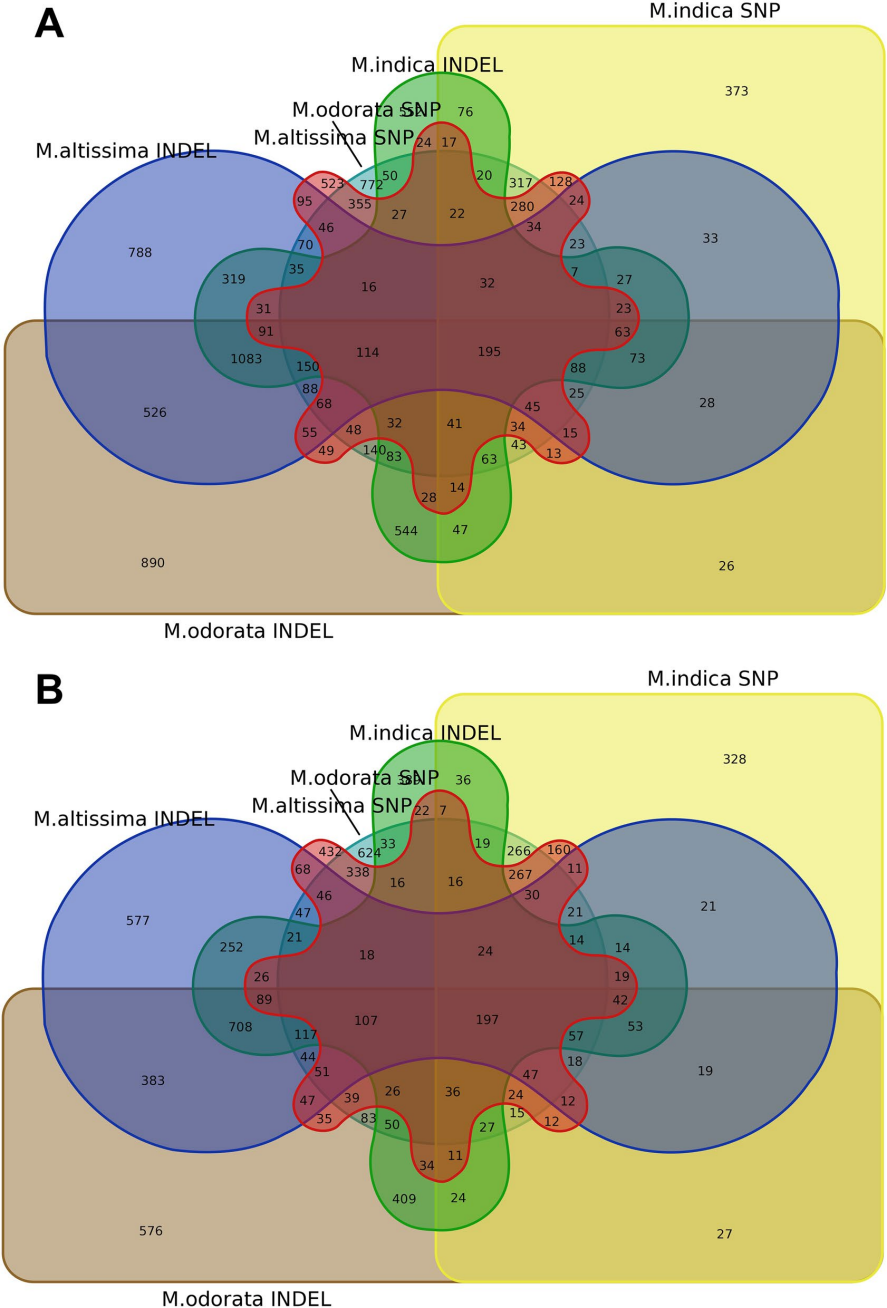
Venn diagram showing the overlap of genes with high-impact SNPs and InDels in all studied Philippine mango species using Alphonso (**a**) and Tommy Atkins (**b**) reference genomes

Compared to the Alphonso genome, 772 and 890 genes with high-impact SNPs and InDels, respectively, were found unique to *M. odorata*, 523 and 788 genes for *M. altissima*, and 373 and 552 genes *for M. indica* Carabao (Fig. 7a). Compared to Tommy Atkins genome, 624 and 576 genes with high-impact SNPs and InDels, respectively, were found unique to *M. odorata*, 432 and 577 genes for *M. altissima*, and 328 and 389 genes *for M. indica* Carabao (Fig. 7b). Meanwhile, 195 and 197 genes with high-impact variant effects were shared among the three Philippine mangoes using the two reference genomes, respectively (Fig. 7, Supplemental File 4).

### Phylogenetic and kinship analyses

In terms of alleles observed in the mango species, *M. odorata* showed the highest number of alleles (1.5 million), followed by *M. altissima* (1.3 million), and lastly *M. indica* Carabao (1.1 million) using the two reference genomes. All allele data (i.e., number of alleles, total heterozygous alleles, total missing alleles, and total polymorphic alleles) are presented in Supplemental Table 2. Meanwhile, phylogenetic analysis revealed 2 clades: clade I includes *M. odorata* and *M. indica* Carabao while clade II includes *M. altissima* only (Supplemental Fig. 1). Kinship analysis showed an IBS value of 1.594 between *M. indica* Carabao and *M. odorata*, IBS value of 1.589 between *M. altissima* and *M. odorata*, and IBS value of 1.531 between *M. indica* Carabao and *M. altissima* (Supplemental Table 3).

## Discussion

Genome-wide variant analysis revealed that most variants (SNPs and InDels) were observed in *M. odorata* (4,353,063 and 4,277,287 for Alphonso and Tommy Atkins genomes, respectively) and least in *M. indica* Carabao (2,755,267 and 2,852,480 for Alphonso and Tommy Atkins genomes, respectively) (Table 2). This result is expected as *M. odorata* is a heterozygous variety and believed to be a cross between *M. indica* and *M. foetida* [5]. *M. indica* and *M. foetida* belong to separate *Mangifera* subgenus: *Mangifera Mangifera* and *Mangifera Limus*, respectively. Hence, *M. odorata* showed the highest variation as a hybrid of the two subgenera. It is followed by *M. altissima*, a highly homozygous, self-pollinating, mango species which belong to the subgenus *Mangifera*. The Carabao variety, although a heterozygous cultivar, showed the least number of variants which could be explained by its conspecificity with the two reference genomes (*M. indica*). Phylogenetic and kinship analyses also revealed that *M. indica* Carabao is more related to *M. odorata* than with *M. altissima*, as shown in the clustering in the dendrogram and kinship (IBS) values (Supplemental Fig. 1, Supplemental Table 3). A pioneering effort of analyzing Philippine mango accessions was reported by Lachica et al. [32] wherein 31,208 SNPs were identified across 341 mango accessions via genotyping-by-sequencing (GBS) (using DArTseq platform). Wang et al. [7] compared 53 mango accessions to the constructed Alphonso genome and identified a total of 21,040,730 variants or 53.9 variants per kilobase on average. These high-confidence variants include 19,433,034 SNPs and 1,607,696 InDels. Meanwhile, by comparing the Kensington Pride mango onto the Tommy Atkins TA4 assembly, Bally et al. [6] identified a total of 9,030,142 variants which comprised of 6,291,666 SNPs, 1,568,959 multi-nucleotide polymorphisms (MNPs), 468,881 InDels, and 700,636 mixed variants, with an average variant rate of one variant every 41 bp.

Many overlapping variants were observed in the three mango species (Fig. 5). These could be utilized for further research of common function or phenotype of *Mangifera* species. On the other hand, approximately 50, 46, and 38% of the variants were unique to *M. odorata*, *M. altissima*, and *M. indica* Carabao, respectively, upon comparison to the two currently available mango reference genomes (Fig. 5). The unique variants could be used for further characterization and genetic research of specific mango species or varieties. The observed Ts/Tv ratios are comparable to the findings of Bally et al. [6] for mango, thus indicating the correctness of the workflow used in this study. The high occurrence of Ts (Fig. 3) is termed as “transition bias” and has been reported in many crop species such as rice [33, 34], foxtail millet [35], maize [36], tea plant [37], and soybean [38]. The high rate of A/G and C/T substitutions (Fig. 3) is likely attributed to the methylation of C when it is adjacent to G (CpG dinucleotides), forming a 5-methylcytosine that can transition into T upon deamination, thus also causing a G to A substitution on the other hand [38, 39]. The number of InDels tends to decrease gradually as the length of InDel increases (Fig. 4). In this study, the predominant InDel length for the mango was 1 to 12 bp with almost half consisting of mononucleotide InDels. In tea plants, the predominant InDel length is 1 to 20 bp with mononucleotide InDels as the most abundant type [37]. More high-impact variants were observed in InDels than SNPs, leading to a greater number of genes with high-impact InDels (Supplemental Table 1). High-impact variants result in protein truncation or triggering loss/gain of function, frameshift variant, or splice donor variant [40].

In the Philippines, the occurrence of insect pests (e.g., oriental fruit fly, cecid fly) and diseases (e.g., anthracnose, scab, stem-end rot) [41–44] limits the country from maximizing mango export potential. These biotic constraints are often difficult to control and can affect mango at different developmental stages causing a significant reduction in fruit yield and quality [45, 46]. Thus, breeding of mango for resistance can provide a long-term solution for the Philippines. The source reference genomes Alphonso and Tommy Atkins are reported for their long shelf life which is also associated to their considerable resistance to diseases [47–49]. This highlights the importance of the identified defense/resistance/immune response-related genes totaling to 6945 genes (Table 5, Supplemental Files 3A and 3B). The two reference varieties also express red/pink blush on their fruit peel, in contrast to the Philippine mango species studied which only appear green or yellow throughout their fruit stages until ripening. In recent years, the Philippines has been interested in developing a mango export variety with a red/pink blush appearance to target international markets that prefer this type of mango. The red/pink blush coloration of mango peel is mainly attributed to anthocyanin production [50] wherein genes related to this biochemical process have been identified in this study totaling to 338 genes (Table 5, Supplemental Files 3A and 3B). KEGG analysis revealed that these genes (including other genes with high impact variants) are involved in the flavonoid biosynthesis pathways which provide precursors for the biosynthesis of anthocyanins (Supplemental Fig. 2).

Analysis of variant effects and functional annotation across the three mango species revealed that 25% of genes containing high-impact variants were found to be novel, or their biological functions have not yet been investigated in mangoes (Supplemental Table 1). Meanwhile, approximately 200 genes with high-impact variants were commonly shared among all mango species which imply consistent gene variations to the two reference genomes (Fig. 7, Supplemental File 4). Analysis of this gene set showed that more than 30% encode proteins related to defense/resistance/immune response against pests and diseases (Supplemental File 4). Among these include the disease resistance proteins At4g27190, At4g27220, At5g63020, and At3g14460 which are proteins reported from *Arabidopsis thaliana*; RPP proteins (RPP13, RPP8, RPP13-like proteins 1, 2, and 3) which provide resistance against downy mildew caused by *Peronospora parasitica* [51–53]; RGA/RGA-blb proteins (RGA1-blb, RGA3-blb, and RGA4-blb) which are known to confer resistance against the devastating late blight disease caused by *Phytophthora infestans* [54, 55]; RPS (RPS2 RPS4, RPS5, and RPS6) and RPM1 proteins which provide resistance against the pathogen *Pseudomonas syringae* [56–58]; and LRK10L-1.2 protein which confers resistance against leaf rust caused by *Puccinia triticina* [59, 60]. Among these proteins, Lantican et al. [12] reported that the mango-specific orthogroup containing disease resistance protein At4g27220 was observed to have the highest number of members among the orthologous RGA (resistance gene analogs) gene sets in mango. Meanwhile, the RPP13-like protein 1 orthogroup is among the largest families of resistance genes in many crops and was also observed to have the highest frequency of gene duplication events in mango [12]. This suggests that these proteins also contributed to the evolutionary adaptation of mango during selective pressure caused by biotic stresses.

## Conclusion

The whole genome of three Philippine mango species *M. odorata* (Huani), *M. altissima* (Paho), and *M. indica* Carabao was successfully sequenced and compared to two currently available mango reference genomes. This revealed the genome-wide variants (SNPs and InDels) including those putative genes with high-impact effects on economically important traits. To date, this is the first sequencing effort to comprehensively analyze genome-wide variants essential for the development of genome-wide markers specific to the Philippine mango species. The availability of this information provides novel genomic resources positioned to revolutionize the mango breeding programs in the Philippines.

## Supplementary Information

**Additional file 1: Supplemental Figure 1**. Phylogenetic analysis of mango species.

**Additional file 2: Supplemental Figure 2**. KEGG pathway (flavonoid biosynthesis).

**Additional file 3: Supplemental File 1**. A_Alphonso SnpEff. B_Tommy Atkins SnpEff.

**Additional file 4: Supplemental File 2**. GO enrichment analysis results.

**Additional file 5: Supplemental File 3**. A_Alphonso Blast2GO results. B_Tommy Atkins Blast2GO results.

**Additional file 6: Supplemental File 4**. Commonly shared genes with high impact variants.

**Additional file 7: Supplemental Table 1**. Breakdown of genes with high impact variants.

**Additional file 8: Supplemental Table 2**. Alleles observed in the mango species.

**Additional file 9: Supplemental Table 3**. Kinship analysis of mango species.

## Data Availability

All data generated or analyzed during this study are included in this published article and its supplementary information files.

## Abbreviations

BAM: Binary alignment map
BLAST: Basic Local Alignment Search Tool
BWA: Burrows-Wheeler aligner
GATK: Genome analysis toolkit
GO: Gene ontology
IBS: Identity-by-state
InDels: Insertions-deletions
KEGG: Kyoto Encyclopedia of Genes and Genomes
LRK10L-1.2: Leaf rust 10 disease-resistance locus receptor-like protein kinase-like 1.2
RGA: Resistance gene analog
RPP: Resistance to *Peronospora parasitica*
RPS: Resistance to *Pseudomonas syringae*
Rvtests: Rare variant tests
SNPs: Single-nucleotide polymorphisms
Ts: Transitions
Tv: Transversions
VCF: Variant call format
