# Supplementary figures and images for: Genome-wide SNP and InDel analysis of three Philippine mango species inferred from whole-genome sequencing
Source: J Genet Eng Biotechnol. 2022 Mar 11;20:46. doi: 10.1186/s43141-022-00326-3 (PMC8917249; doi:10.1186/s43141-022-00326-3)

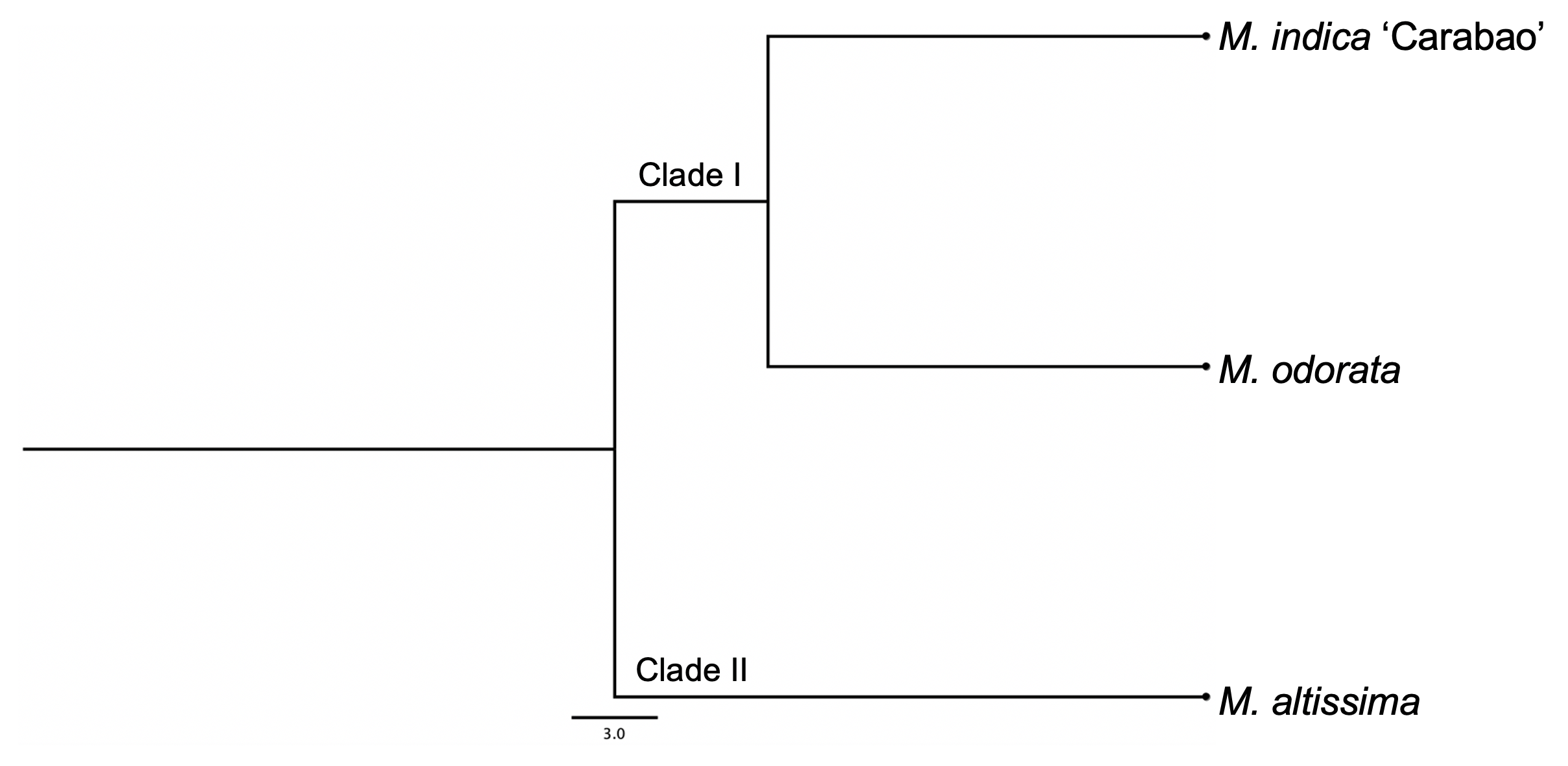

Supplement: Supplementary file 1 — Additional file 1: Supplemental Figure 1. Phylogenetic analysis of mango species. [file 43141_2022_326_MOESM1_ESM.png]

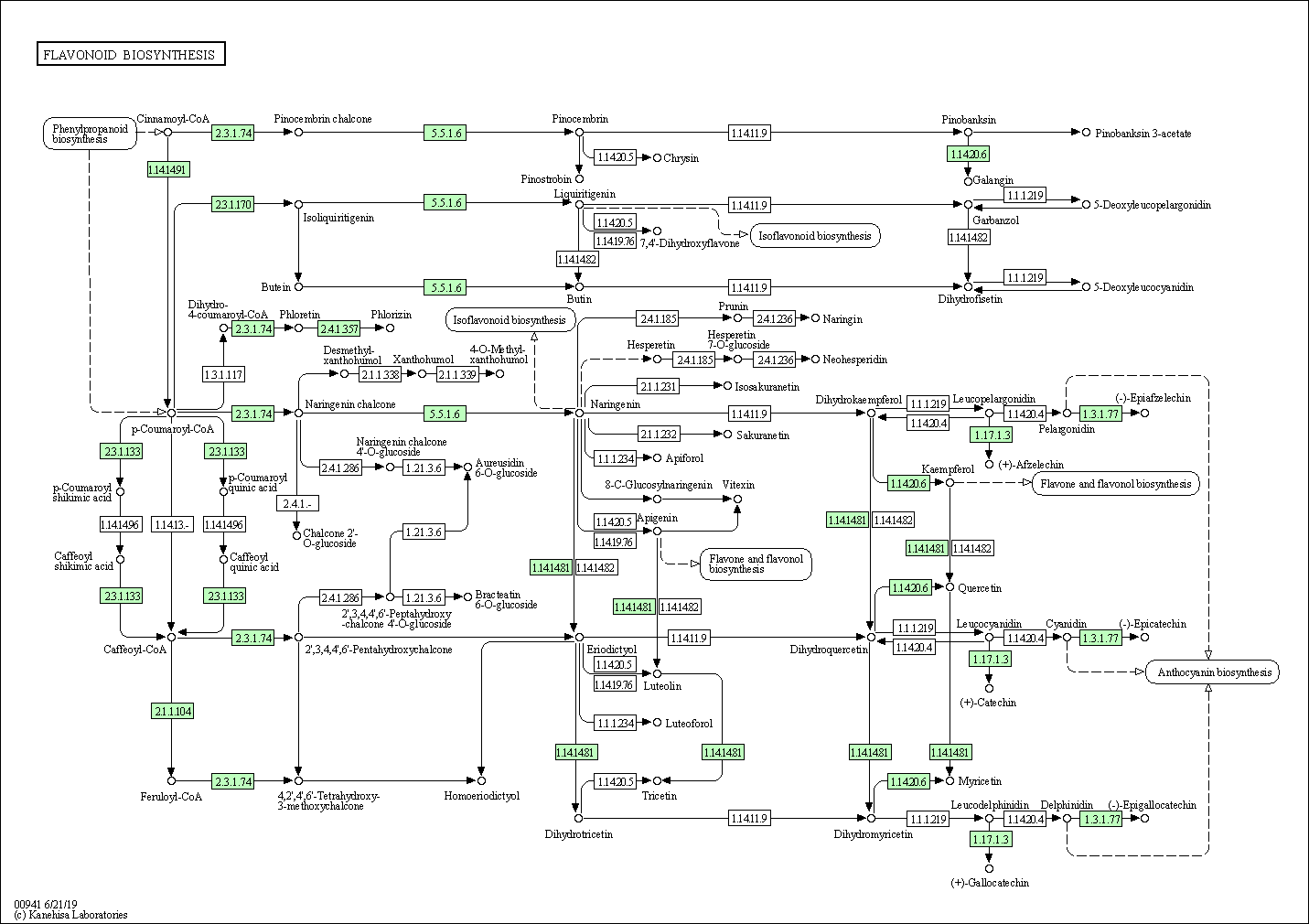

Supplement: Supplementary file 2 — Additional file 2: Supplemental Figure 2. KEGG pathway (flavonoid biosynthesis). [file 43141_2022_326_MOESM2_ESM.png]
