# Supplementary material for: Genome-wide SNP and InDel analysis of three Philippine mango species inferred from whole-genome sequencing
Source: J Genet Eng Biotechnol. 2022 Mar 11;20:46. doi: 10.1186/s43141-022-00326-3 (PMC8917249; doi:10.1186/s43141-022-00326-3)
Supplement: Supplementary file 3 — Additional file 3: Supplemental File 1. A_Alphonso SnpEff. B_Tommy Atkins SnpEff. [file 43141_2022_326_MOESM3_ESM.zip › Supplemental File 1A_Alphonso SnpEffR2.pdf]

SnpEff: Variant analysis

Contents

Summary
Variant rate by chromosome
Variants by type
Number of variants by impact
Number of variants by functional class
Number of variants by effect
Quality histogram
InDel length histogram
Base variant table
Transition vs transversions (ts/tv)
Allele frequency
Allele Count
Codon change table
Amino acid change table
Chromosome variants plots
Details by gene

Summary

**Genome**	manindi
**Date**	2021-05-22 14:21
**SnpEff version**	`SnpEff 5.0e (build 2021-03-09 06:01), by Pablo Cingolani`
**Command line arguments**	`SnpEff manindi /home/cocogenomics/mango_genome/mango_ref_genome/M.odorata_ref_SNP_final.vcf`
**Warnings**	393,002
**Errors**	0
**Number of lines (input file)**	3,813,664
**Number of variants (before filter)**	3,826,194
**Number of not variants (i.e. reference equals alternative)**	0
**Number of variants processed (i.e. after filter and non-variants)**	3,826,194
**Number of known variants (i.e. non-empty ID)**	0 (0%)
**Number of multi-allelic VCF entries (i.e. more than two alleles)**	12,530
**Number of effects**	7,779,942
**Genome total length**	391,108,416
**Genome effective length**	357,440,369
**Variant rate**	1 variant every 93 bases

Variants rate details

Chromosome	Length	Variants	Variants rate
1	29,456,600	336,179	87
2	24,397,897	250,878	97
3	23,139,393	229,080	101
4	21,507,500	215,264	99
5	21,083,371	201,318	104
6	18,811,960	214,790	87
7	20,623,120	189,318	108
8	18,243,469	196,823	92
9	18,233,274	222,767	81
10	17,652,500	195,893	90
11	17,144,574	157,189	109
12	16,029,966	183,526	87
13	15,457,994	164,140	94
14	14,810,209	159,569	92
15	14,765,960	179,890	82
16	13,817,227	138,400	99
17	13,506,273	158,389	85
18	13,371,754	154,479	86
19	13,093,066	138,580	94
20	12,294,262	139,722	87
**Total**	**357,440,369**	**3,826,194**	**93**

Number variants by type

Type	Total
**SNP**	3,826,194
**MNP**	0
**INS**	0
**DEL**	0
**MIXED**	0
**INV**	0
**DUP**	0
**BND**	0
**Total**	**3,826,194**

Type	Total
INTERVAL	0
Total	3,826,194

Number of effects by impact

Type (alphabetical order)	Count	Percent
HIGH	4,583	0.059%
LOW	102,829	1.322%
MODERATE	125,037	1.607%
MODIFIER	7,547,493	97.012%

Number of effects by functional class

Type (alphabetical order)	Count	Percent
MISSENSE	125,841	58.229%
NONSENSE	2,605	1.205%
SILENT	87,668	40.566%

Missense / Silent ratio: 1.4354

Number of effects by type and region

Type

Type (alphabetical order)	Count	Percent
3_prime_UTR_variant	34,712	0.445%
5_prime_UTR_premature_start_codon_gain_variant	3,076	0.039%
5_prime_UTR_variant	19,305	0.248%
downstream_gene_variant	1,903,377	24.412%
initiator_codon_variant	30	0%
intergenic_region	3,033,446	38.906%
intron_variant	528,400	6.777%
missense_variant	125,037	1.604%
splice_acceptor_variant	643	0.008%
splice_donor_variant	545	0.007%
splice_region_variant	16,351	0.21%
start_lost	302	0.004%
start_retained_variant	13	0%
stop_gained	2,601	0.033%
stop_lost	492	0.006%
stop_retained_variant	191	0.002%
synonymous_variant	87,448	1.122%
upstream_gene_variant	2,040,834	26.175%

Region

Type (alphabetical order)	Count	Percent
DOWNSTREAM	1,903,377	24.465%
EXON	214,408	2.756%
INTERGENIC	3,033,446	38.991%
INTRON	515,819	6.63%
SPLICE_SITE_ACCEPTOR	643	0.008%
SPLICE_SITE_DONOR	545	0.007%
SPLICE_SITE_REGION	13,777	0.177%
UPSTREAM	2,040,834	26.232%
UTR_3_PRIME	34,712	0.446%
UTR_5_PRIME	22,381	0.288%

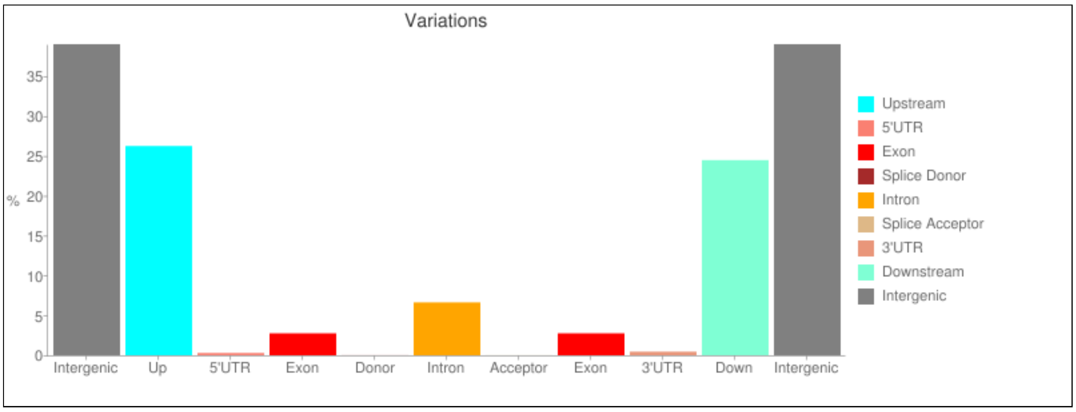

Quality:

**Min**	10
**Max**	81,395
**Mean**	146.986
**Median**	118
**Standard deviation**	214.835
**Values**	10,11,12,13,14,15,16,17,18,19,20,21,22,23,24,25,26,27,28,29,30,31,32,33,34,35,36,37,38,39,40,41,42,43,44,45,46,47,48,49,50,51,52,53,54,55,56,57
**Count**	1438,1853,4531,2634,1941,1482,3996,5409,14764,14896,13939,20938,23038,19053,28711,32983,22705,28949,30322,15123,11181,19323,7634,64

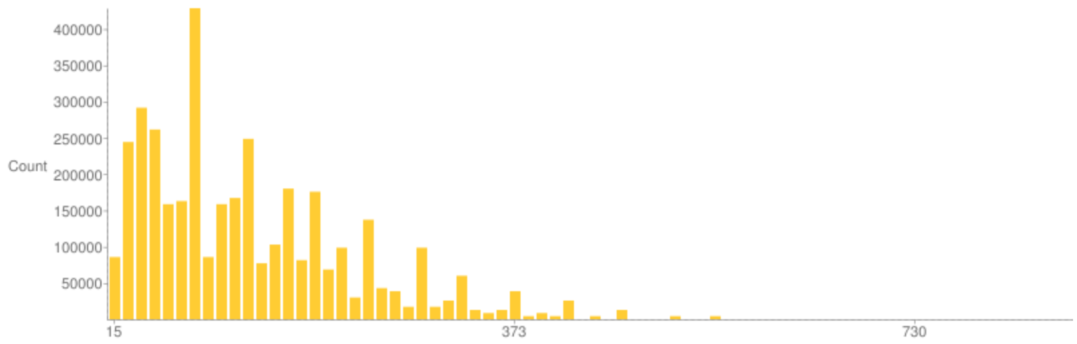

Insertions and deletions length:

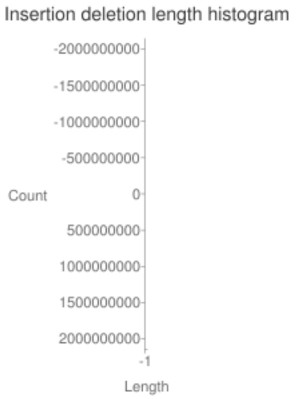

Base changes (SNPs)

	A	C	G	T
**A**	0	134,175	650,137	193,056
**C**	139,889	0	90,962	703,977
**G**	705,516	90,679	0	140,813
**T**	193,459	649,432	134,099	0

Ts/Tv (transitions / transversions)

Note: Only SNPs are used for this statistic.
Note: This Ts/Tv ratio is a 'raw' ratio (ratio of observed events).

**Transitions**	3,585,404
**Transversions**	1,462,720
**Ts/Tv ratio**	2.4512

All variants:

```
Sample ,readname,Total
Transitions ,3585404,3585404
Transversions ,1462720,1462720
Ts/Tv ,2.451,2.451
```

Only known variants (i.e. the ones having a non-empty ID field):

```
No results available (empty input?)
```

Allele frequency

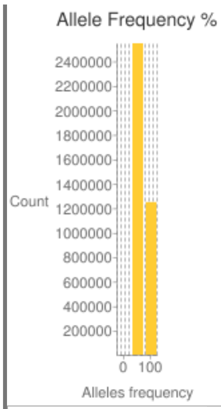

**Min**	0
**Max**	100
**Mean**	66.513
**Median**	50
**Standard deviation**	23.608
**Values**	0,50,100
**Count**	3324,2547496,1262844

Allele Count

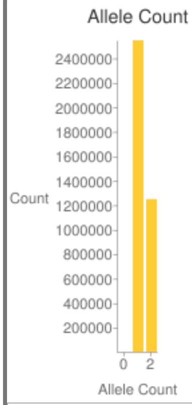

**Min**	0
**Max**	2
**Mean**	1.33
**Median**	1
**Standard deviation**	0.472
**Values**	0,1,2
**Count**	3324,2547496,1262844

Hom/Het per sample

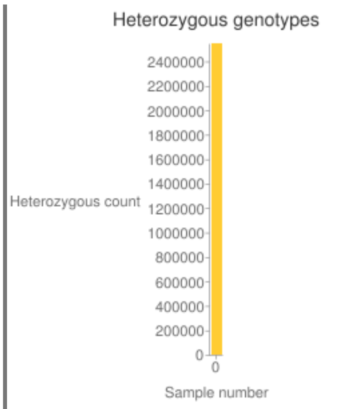

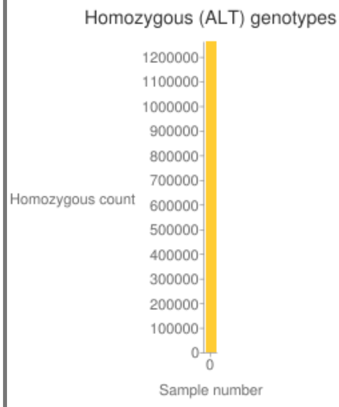

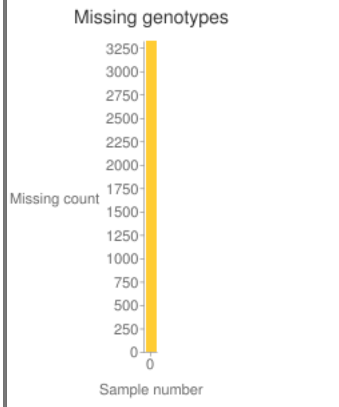


```
Sample_names , readname
Reference , 0
Het , 2547496
Hom , 1262844
Missing , 3324
```

Codon changes

How to read this table:
- Rows are reference codons and columns are changed codons. E.g. Row 'AAA' column 'TAA' indicates how many 'AAA' codons have been replaced by 'TAA' codons.
- Red background colors indicate that more changes happened (heat-map).
- Diagonals are indicated using grey background color
- WARNING: This table may include different translation codon tables (e.g. mamalian DNA and mitochondrial DNA).

	AAA	AAC	AAG	AAT	ACA	ACC	ACG	ACT	AGA	AGC	AGG	AGT	ATA	ATC	ATG	ATT	CAA	CAC	CAG	CAT	CCA
**AAA**		302	1,532	427	298				837				224				350				
**AAC**	361		301	1,563		157				606				105				118			
**AAG**	1,632	308		398			163				886				269				323		
**AAT**	378	1,362	371					309				930				256				259	
**ACA**	308					270	968	480	209				720								125
**ACC**		191			238		176	1,191		184				332							
**ACG**			189		1,159	183		262			120				671						
**ACT**				301	470	1,069	197					392				768					
**AGA**	876				226					127	758	140	190								
**AGC**		687				181			148		134	975		85							
**AGG**			810				85		761	124		171			145						
**AGT**				901				402	110	849	153					221					
**ATA**	188				607				122					291	743	470					
**ATC**		95				295				82			332		171	1,301					
**ATG**			237				694				152		974	172		318					
**ATT**				288				626				206	450	1,069	321						
**CAA**	408																	161	1,255	227	201

	AAA	AAC	AAG	AAT	ACA	ACC	ACG	ACT	AGA	AGC	AGG	AGT	ATA	ATC	ATG	ATT	CAA	CAC	CAG	CAT	CCA
**CAC**		112															160		176	782	
**CAG**			274														1,297	206		192	
**CAT**				281													217	689	185		
**CCA**					168												184				
**CCC**						96												65			231
**CCG**							47												117		1,531
**CCT**								169												188	403
**CGA**									278								849				99
**CGC**										80								435			
**CGG**											200								677		
**CGT**												164								876	
**CTA**													160				148				428
**CTC**														155				69			
**CTG**															213				192		
**CTT**																339				181	
**GAA**	1,466																412				
**GAC**		611																93			
**GAG**			1,302																401		
**GAT**				1,355																306	
**GCA**					1,116																229
**GCC**						552															
**GCG**							209														
**GCT**								1,054													
**GGA**									779												
**GGC**										496											
**GGG**											469										
**GGT**												886									
**GTA**													764								
**GTC**														701							
**GTG**															874						
**GTT**																1,435					
**TAA**	23																73				
**TAC**		112																282			
**TAG**			16																47		
**TAT**				236																620	
**TCA**					218																568
**TCC**						109															
**TCG**							60														
**TCT**								252													
**TGA**									16												
**TGC**										97											
**TGG**											86										
**TGT**												173									
**TTA**													207								
**TTC**														126							
**TTG**															300						
**TTT**																300					

Amino acid changes

How to read this table:
- Rows are reference amino acids and columns are changed amino acids. E.g. Row 'A' column 'E' indicates how many 'A' amino acids have been replaced by 'E' amino acids.
- Red background colors indicate that more changes happened (heat-map).
- Diagonals are indicated using grey background color
- WARNING: This table may include different translation codon tables (e.g. mamalian DNA and mitochondrial DNA).

	*	?	A	C	D	E	F	G	H	I	K	L	M	N	P	Q	R	S	T	V	W	[illegible]
*****	**191**			18		32		14			39	37				120	66	33			80	
**?**		**1**																				
**A**			**7,937**		352	419		826							608			1,023	2,931	2,880		
**C**	60			**1,427**			261	231									710	646			141	[illegible]
**D**			291		**3,409**	1,591		1,068	399					1,966						242		[illegible]
**E**	238		433		1,598	**2,956**		1,218			2,768					813				373		
**F**				272			**2,828**			426		1,972						862		466		[illegible]
**G**	82		763	264	1,065	1,186		**6,041**									1,639	1,382		555	99	
**H**					365				**1,471**			231		393	220	738	997					[illegible]
**I**							413			**3,913**	188	773	1,235	383			122	288	1,528	2,483		
**K**	147					2,198				224	**3,164**		269	1,435		673	1,723		461			
**L**	184						2,193		250	861		**13,248**	513		1,532	340	336	1,258		1,407	157	
**M**										1,464	237	583					152		694	726		
**N**					1,627				377	361	1,411			**2,925**				1,536	466			[illegible]
**P**			563						253			1,804			**6,633**	301	295	1,734	480			
**Q**	674					763			786		682	369			296	**2,552**	1,292					

	*	?	A	C	D	E	F	G	H	I	K	L	M	N	P	Q	R	S	T	V	W	[illegible]
**R**	357			916				1,396	1,311	190	1,686	400	145		344	1,526	**4,611**	806	311		480	
**S**	180		919	700			1,024	1,240		306		1,518		1,588	1,590		736	**9,573**	1,222		93	[illegible]
**T**			2,512							1,820	497		671	492	417		329	1,205	**6,663**			
**V**			2,436		219	291	540	482		2,900		1,469	874							**6,059**		
**W**	473			142				86				143					414	78				
**Y**	210			818	360		520		902					348				336				**[illegible]**

Variants by chromosome

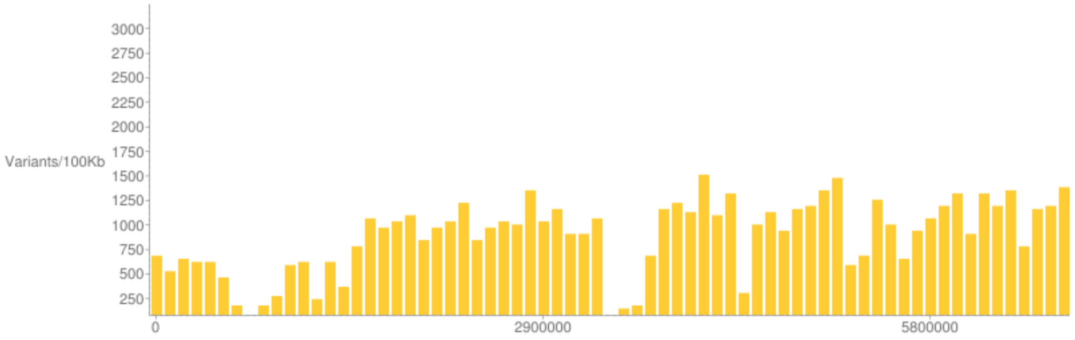

1, Position,0,100000,200000,300000,400000,500000,600000,700000,800000,900000,1000000,1100000,1200000,1300000,1400000,
1,Count,692,538,650,648,646,463,202,102,177,278,603,644,251,641,387,806,1076,997,1031,1099,868,967,1029,1236,865,982,1031,1004,1361,

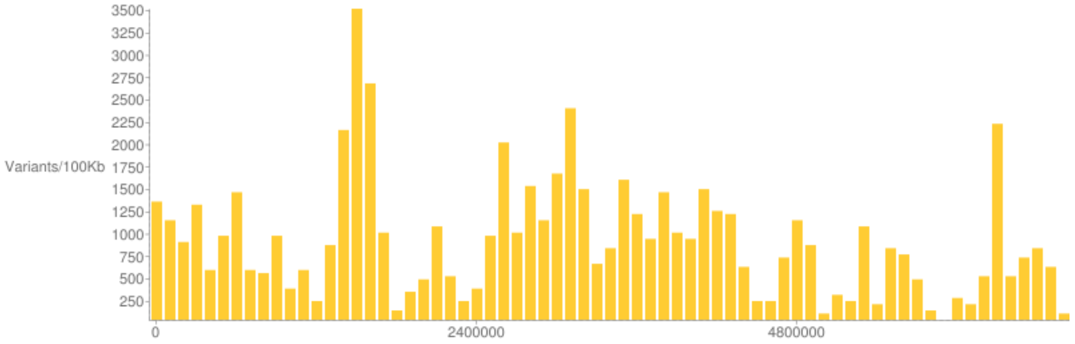

2, Position,0,100000,200000,300000,400000,500000,600000,700000,800000,900000,1000000,1100000,1200000,1300000,1400000,
2,Count,1379,1181,911,1352,606,1008,1495,607,561,1004,419,627,256,894,2167,3515,2694,1045,161,375,514,1082,529,269,396,990,2032,1016,

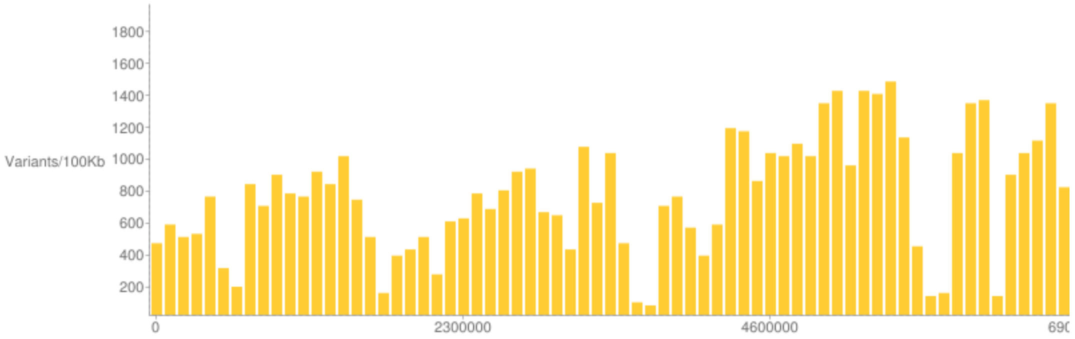

3, Position,0,100000,200000,300000,400000,500000,600000,700000,800000,900000,1000000,1100000,1200000,1300000,1400000,
3,Count,475,606,521,532,773,318,216,845,717,918,795,772,928,854,1021,756,509,163,404,432,515,291,611,635,788,685,804,923,946,675,656,

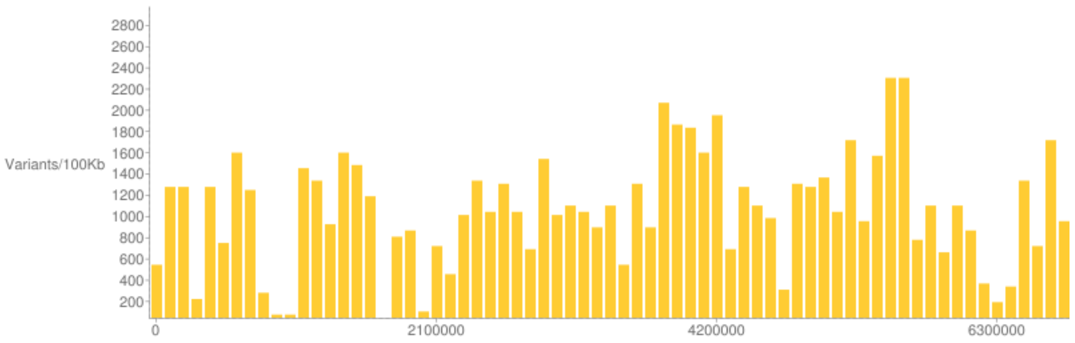

4, Position,0,100000,200000,300000,400000,500000,600000,700000,800000,900000,1000000,1100000,1200000,1300000,1400000,
4,Count,566,1274,1298,235,1294,746,1617,1266,286,82,87,1466,1339,923,1597,1482,1210,41,805,891,109,731,456,1012,1355,1066,1317,1067,7

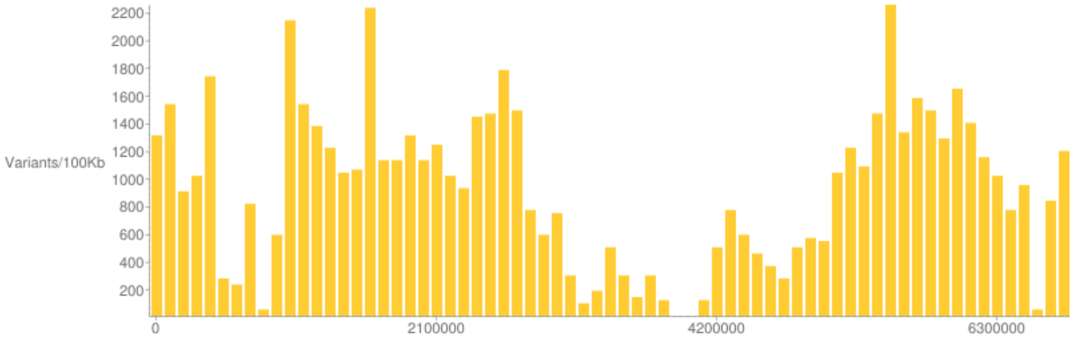

5, Position,0,100000,200000,300000,400000,500000,600000,700000,800000,900000,1000000,1100000,1200000,1300000,1400000,
5,Count,1313,1550,926,1024,1753,284,239,841,68,598,2147,1559,1382,1240,1044,1068,2239,1149,1150,1334,1145,1263,1031,933,1457,1490,180

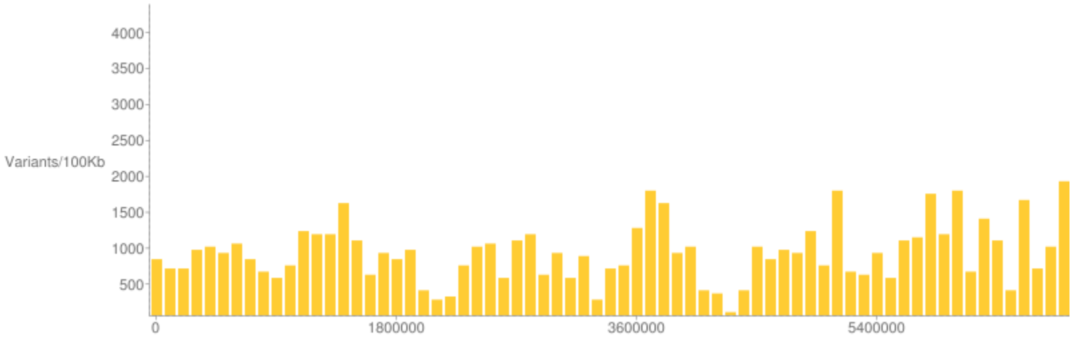

6, Position,0,100000,200000,300000,400000,500000,600000,700000,800000,900000,1000000,1100000,1200000,1300000,1400000,
6,Count,862,723,733,978,1052,933,1058,856,694,585,774,1261,1197,1196,1639,1116,661,967,871,993,407,298,347,790,1053,1057,601,1105,120

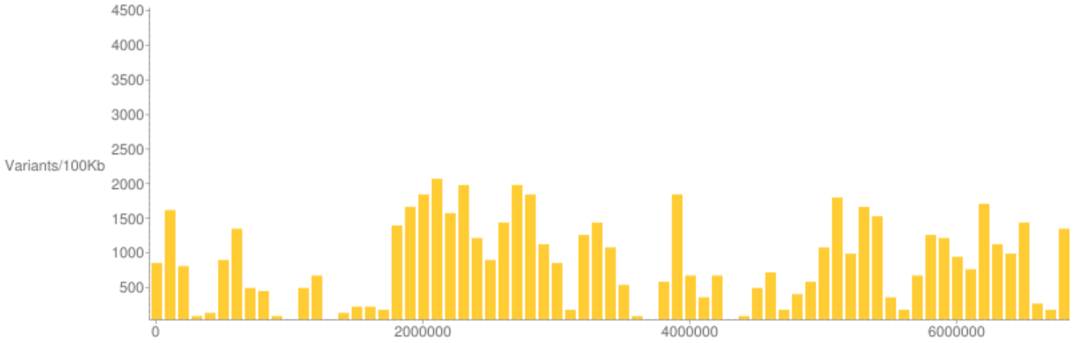

7, Position,0,100000,200000,300000,400000,500000,600000,700000,800000,900000,1000000,1100000,1200000,1300000,1400000,
7,Count,850,1647,819,106,129,926,1357,507,483,102,43,524,671,34,154,230,247,170,1394,1687,1855,2076,1606,1998,1225,928,1439,1978,1863,

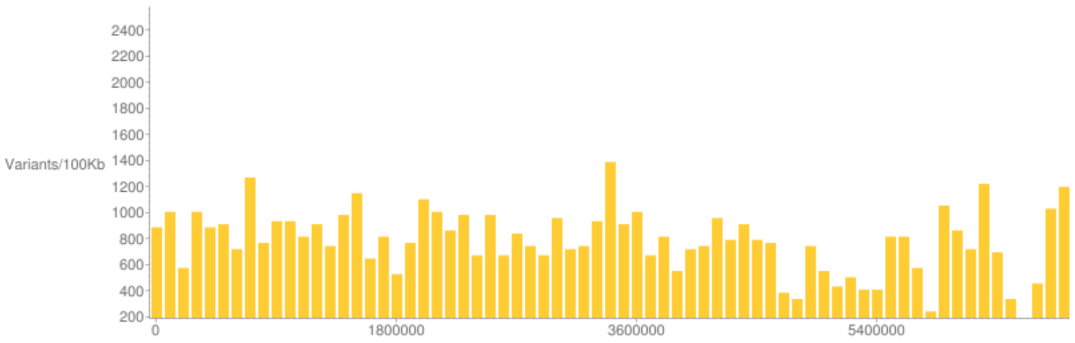

8, Position,0,100000,200000,300000,400000,500000,600000,700000,800000,900000,1000000,1100000,1200000,1300000,1400000,
8,Count,888,1017,576,1010,897,925,716,1266,775,950,930,833,920,738,998,1152,658,812,526,762,1118,1022,878,988,676,980,683,837,756,671,

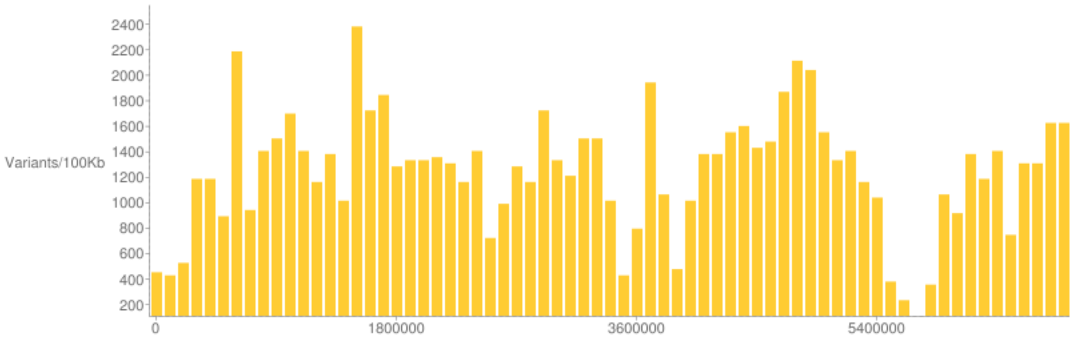

9, Position,0,100000,200000,300000,400000,500000,600000,700000,800000,900000,1000000,1100000,1200000,1300000,1400000,
9,Count,456,427,538,1196,1201,910,2196,941,1404,1522,1713,1410,1174,1391,1026,2394,1743,1855,1281,1332,1348,1356,1326,1176,1420,720,

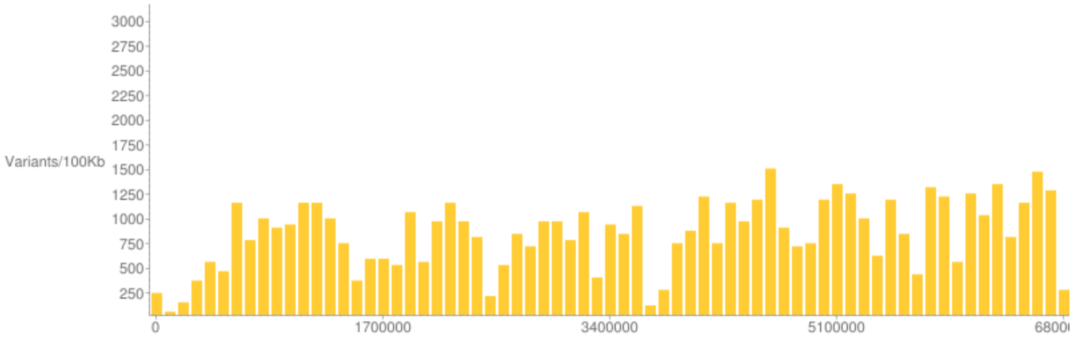

10, Position,0,100000,200000,300000,400000,500000,600000,700000,800000,900000,1000000,1100000,1200000,1300000,1400000,
10,Count,266,56,171,371,569,470,1189,783,1026,931,965,1180,1182,1029,768,401,606,620,531,1095,583,999,1175,985,815,230,547,870,723,

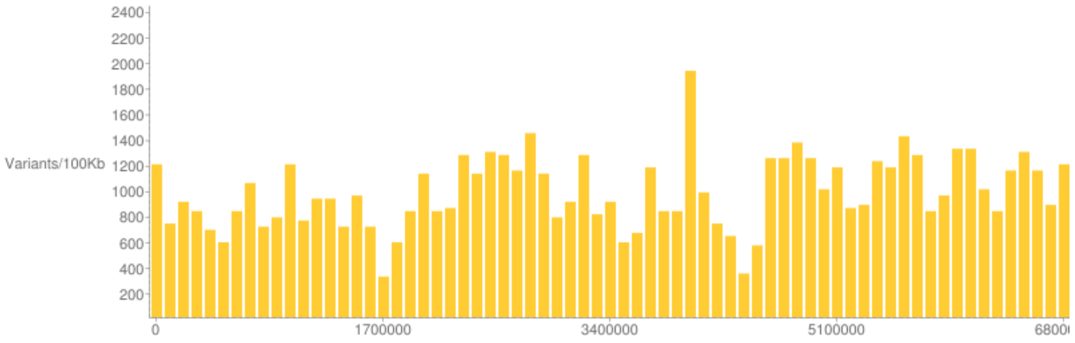

11, Position,0,100000,200000,300000,400000,500000,600000,700000,800000,900000,1000000,1100000,1200000,1300000,1400000,
11,Count,1210,752,941,846,710,604,864,1067,729,800,1218,780,946,957,732,979,730,344,605,845,1139,844,889,1301,1138,1325,1296,1161,

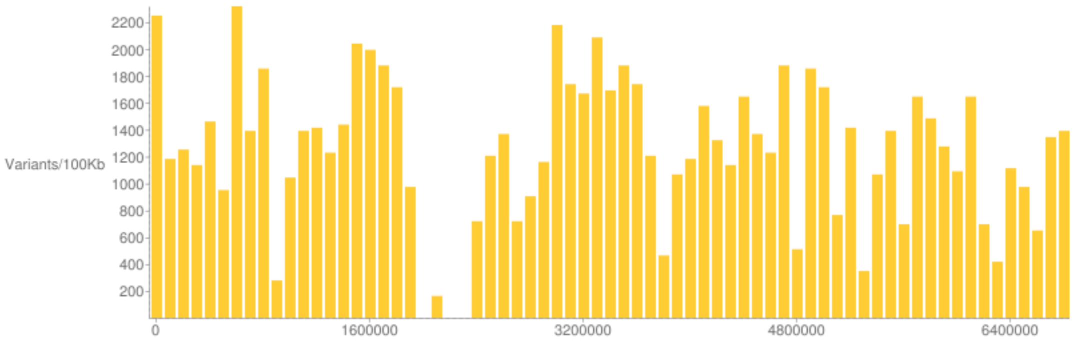

12, Position,0,100000,200000,300000,400000,500000,600000,700000,800000,900000,1000000,1100000,1200000,1300000,1400000,
12,Count,2264,1202,1263,1142,1476,973,2319,1392,1873,290,1064,1414,1424,1251,1453,2051,2011,1888,1731,994,1,172,23,18,733,1222,1370,7

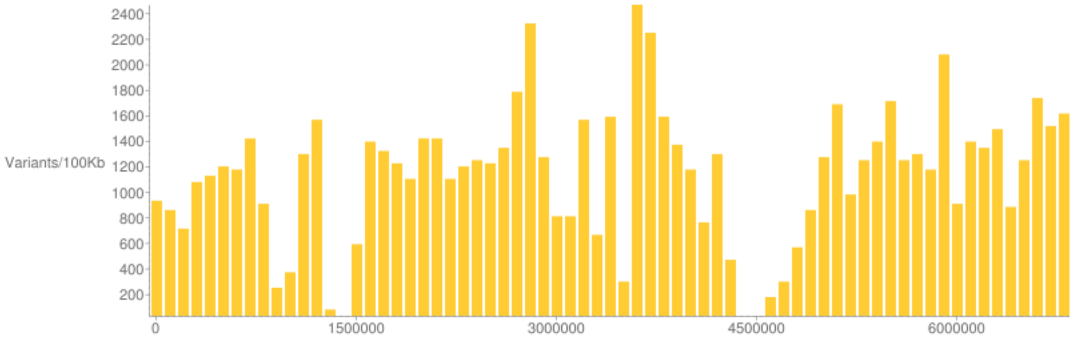

13, Position,0,100000,200000,300000,400000,500000,600000,700000,800000,900000,1000000,1100000,1200000,1300000,1400000,
13,Count,951,875,714,1092,1149,1216,1186,1428,909,260,381,1302,1569,89,29,590,1403,1344,1231,1118,1426,1421,1119,1207,1256,1234,1363,

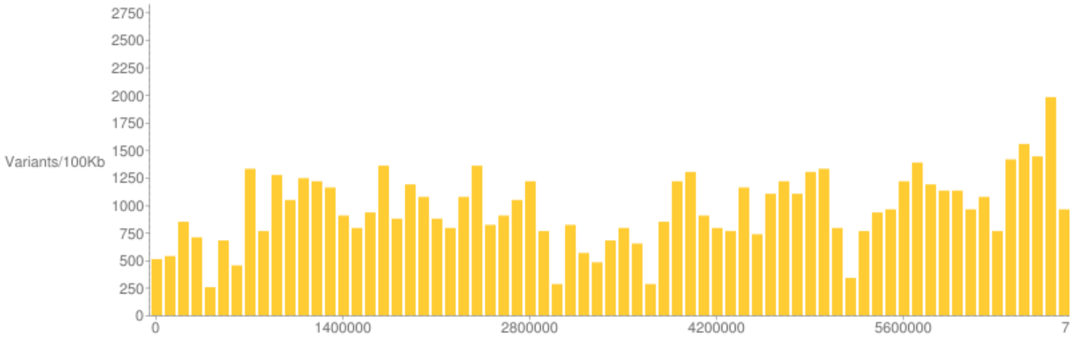

14, Position,0,100000,200000,300000,400000,500000,600000,700000,800000,900000,1000000,1100000,1200000,1300000,1400000,
14,Count,536,550,861,710,275,695,474,1351,764,1296,1060,1250,1237,1187,915,798,933,1372,900,1191,1078,881,811,1087,1376,831,906,1071,

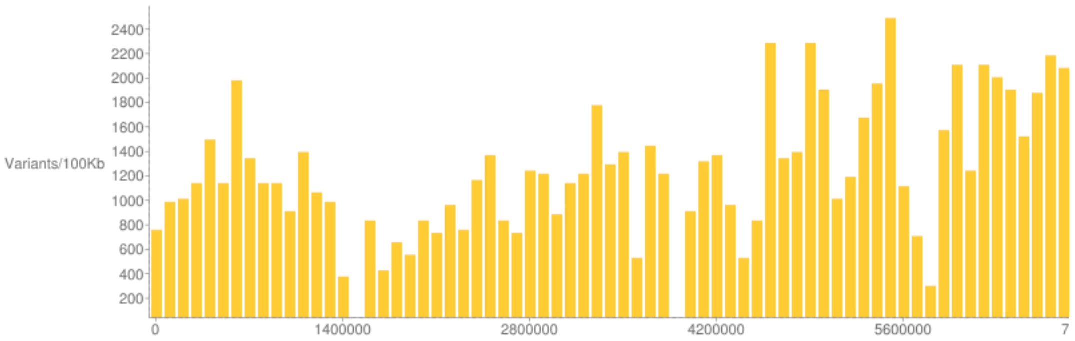

15, Position,0,100000,200000,300000,400000,500000,600000,700000,800000,900000,1000000,1100000,1200000,1300000,1400000,
15,Count,778,998,1027,1150,1517,1158,1994,1351,1160,1154,914,1395,1085,1002,398,45,850,435,661,573,834,730,975,776,1162,1383,839,742,

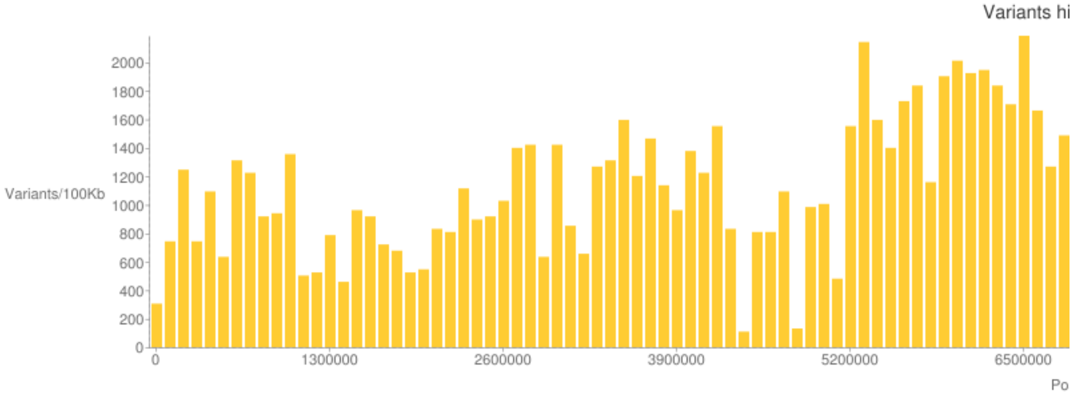

16, Position,0,100000,200000,300000,400000,500000,600000,700000,800000,900000,1000000,1100000,1200000,1300000,1400000,
16,Count,323,754,1250,765,1105,642,1329,1238,934,944,1366,523,533,809,464,973,922,732,690,535,561,844,825,1137,905,932,1046,1411,1431,

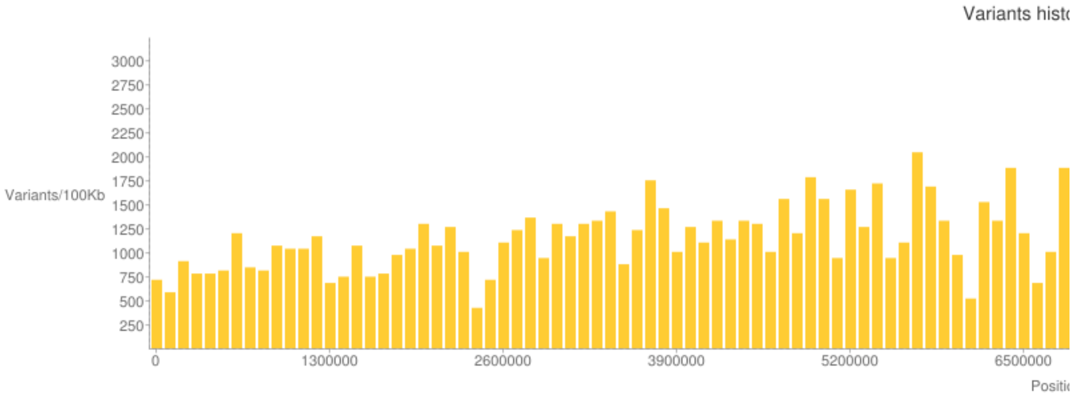

17, Position,0,100000,200000,300000,400000,500000,600000,700000,800000,900000,1000000,1100000,1200000,1300000,1400000,
17,Count,741,597,933,782,796,825,1206,867,835,1104,1040,1051,1173,696,752,1093,772,799,1000,1057,1312,1102,1270,1035,439,734,1130,124

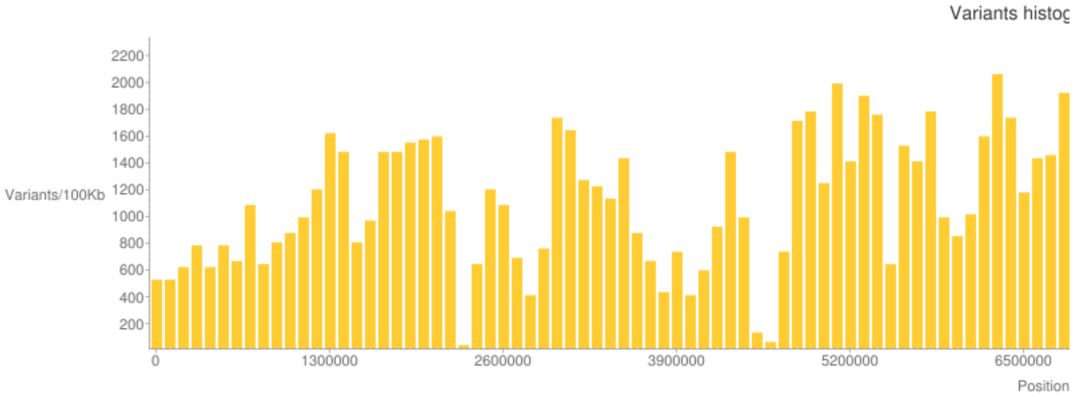

18, Position,0,100000,200000,300000,400000,500000,600000,700000,800000,900000,1000000,1100000,1200000,1300000,1400000,
18,Count,534,530,624,779,628,783,667,1094,659,822,877,1005,1207,1634,1477,803,969,1483,1494,1560,1584,1607,1035,55,647,1197,1094,703,

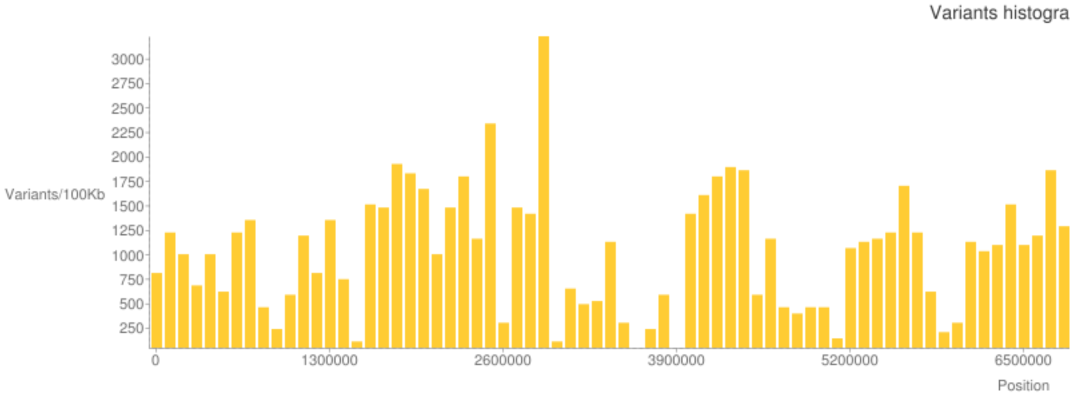

19, Position,0,100000,200000,300000,400000,500000,600000,700000,800000,900000,1000000,1100000,1200000,1300000,1400000,
19,Count,821,1245,1029,684,1000,647,1253,1352,482,264,615,1208,832,1352,765,127,1531,1478,1929,1838,1668,1023,1505,1798,1176,2346,312,

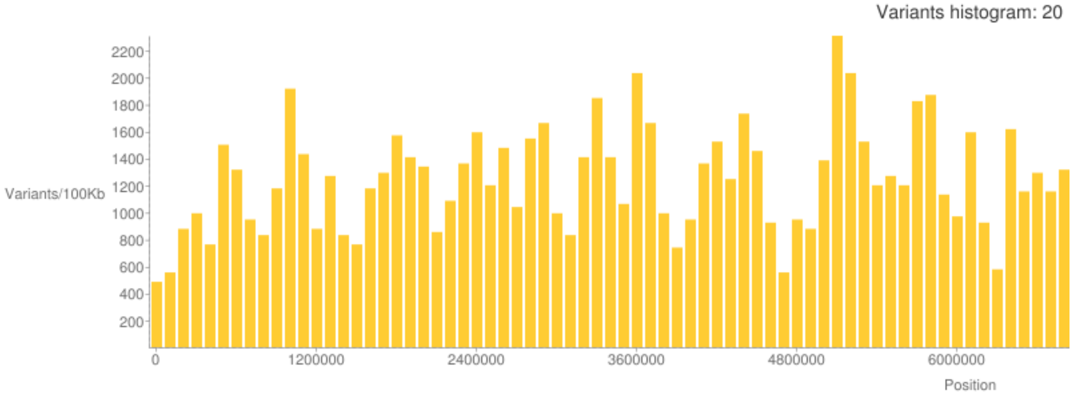


```
20, Position,0,100000,200000,300000,400000,500000,600000,700000,800000,900000,1000000,1100000,1200000,1300000,1400000,
20,Count,493,560,882,1005,781,1515,1333,972,843,1190,1939,1441,901,1278,855,768,1189,1297,1591,1432,1343,864,1107,1378,1613,1225,1491,
```

Details by gene

Here you can find a tab-separated table.

SnpEff: Variant analysis

Contents

Summary
Variant rate by chromosome
Variants by type
Number of variants by impact
Number of variants by functional class
Number of variants by effect
Quality histogram
InDel length histogram
Base variant table
Transition vs transversions (ts/tv)
Allele frequency
Allele Count
Codon change table
Amino acid change table
Chromosome variants plots
Details by gene

Summary

**Genome**	manindi
**Date**	2021-05-22 14:39
**SnpEff version**	`SnpEff 5.0e (build 2021-03-09 06:01), by Pablo Cingolani`
**Command line arguments**	`SnpEff manindi /home/cocogenomics/mango_genome/mango_ref_genome/M.odorata_ref_INDEL_final.vcf`
**Warnings**	68,296
**Errors**	0
**Number of lines (input file)**	524,356
**Number of variants (before filter)**	526,869
**Number of not variants (i.e. reference equals alternative)**	0
**Number of variants processed (i.e. after filter and non-variants)**	526,869
**Number of known variants (i.e. non-empty ID)**	0 (0%)
**Number of multi-allelic VCF entries (i.e. more than two alleles)**	2,513
**Number of effects**	1,162,995
**Genome total length**	391,108,416
**Genome effective length**	357,440,369
**Variant rate**	1 variant every 678 bases

Variants rate details

Chromosome	Length	Variants	Variants rate
1	29,456,600	45,114	652
2	24,397,897	36,154	674
3	23,139,393	32,112	720
4	21,507,500	29,828	721
5	21,083,371	28,717	734
6	18,811,960	29,564	636
7	20,623,120	28,015	736
8	18,243,469	25,469	716
9	18,233,274	30,719	593
10	17,652,500	25,671	687
11	17,144,574	21,527	796
12	16,029,966	26,578	603
13	15,457,994	21,861	707
14	14,810,209	22,440	659
15	14,765,960	23,258	634
16	13,817,227	19,123	722
17	13,506,273	22,685	595
18	13,371,754	20,044	667
19	13,093,066	20,151	649
20	12,294,262	17,839	689
**Total**	**357,440,369**	**526,869**	**678**

Number variants by type

Type	Total
**SNP**	0
**MNP**	0
**INS**	261,867
**DEL**	265,002
**MIXED**	0
**INV**	0
**DUP**	0
**BND**	0
**Total**	**526,869**

Type	Total
INTERVAL	0
Total	526,869

Number of effects by impact

Type (alphabetical order)	Count	Percent
HIGH	7,963	0.685%
LOW	1,954	0.168%
MODERATE	3,503	0.301%
MODIFIER	1,149,575	98.846%

Number of effects by functional class

Type (alphabetical order)	Count	Percent

Missense / Silent ratio: 0

Number of effects by type and region

Type

Type (alphabetical order)	Count	Percent
3_prime_UTR_variant	6,098	0.523%
5_prime_UTR_variant	3,913	0.336%
bidirectional_gene_fusion	3	0%
conservative_inframe_deletion	708	0.061%
conservative_inframe_insertion	796	0.068%
disruptive_inframe_deletion	1,266	0.109%
disruptive_inframe_insertion	842	0.072%
downstream_gene_variant	299,752	25.703%
frameshift_variant	7,571	0.649%
intergenic_region	422,962	36.268%
intragenic_variant	2	0%
intron_variant	83,767	7.183%
non_coding_transcript_variant	118	0.01%
splice_acceptor_variant	151	0.013%
splice_donor_variant	252	0.022%
splice_region_variant	2,412	0.207%
start_lost	112	0.01%
start_retained_variant	11	0.001%
stop_gained	242	0.021%
stop_lost	96	0.008%
stop_retained_variant	11	0.001%
upstream_gene_variant	335,143	28.737%

Region

Type (alphabetical order)	Count	Percent
DOWNSTREAM	299,740	25.773%
EXON	11,178	0.961%
GENE	3	0%
INTERGENIC	422,962	36.368%
INTRON	81,586	7.015%
SPLICE_SITE_ACCEPTOR	111	0.01%
SPLICE_SITE_DONOR	196	0.017%
SPLICE_SITE_REGION	1,954	0.168%
TRANSCRIPT	120	0.01%
UPSTREAM	335,143	28.817%
UTR_3_PRIME	6,094	0.524%
UTR_5_PRIME	3,908	0.336%

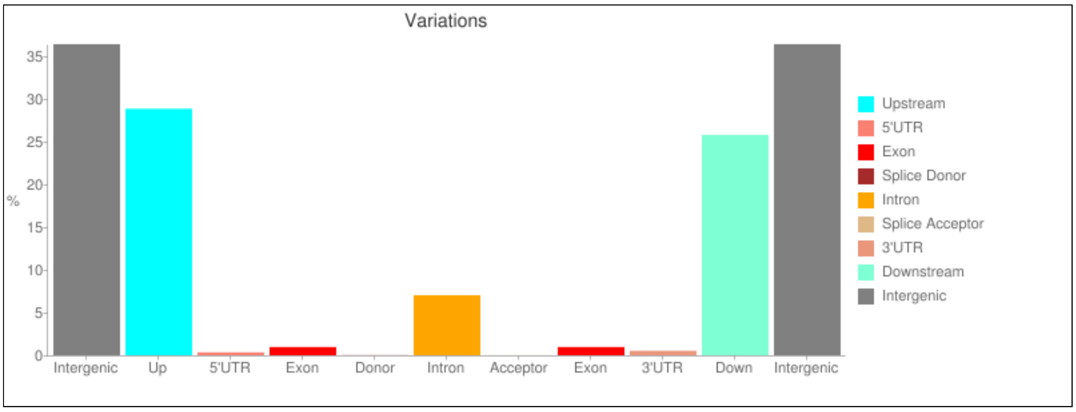

Quality:

**Min**	10
**Max**	92,508
**Mean**	128.464
**Median**	98
**Standard deviation**	350.016
**Values**	10,11,12,13,14,15,16,17,18,19,20,21,22,23,24,25,26,27,28,29,30,31,32,33,34,35,36,37,38,39,40,41,42,43,44,45,46,47,48,49,50,51,52,53,54,55,56,57
**Count**	1492,2065,2513,2078,1533,1564,2223,1646,1674,2306,1499,1475,2770,1541,1433,3439,1416,1359,4758,1445,1526,7403,1976,1762,10375,1452,1

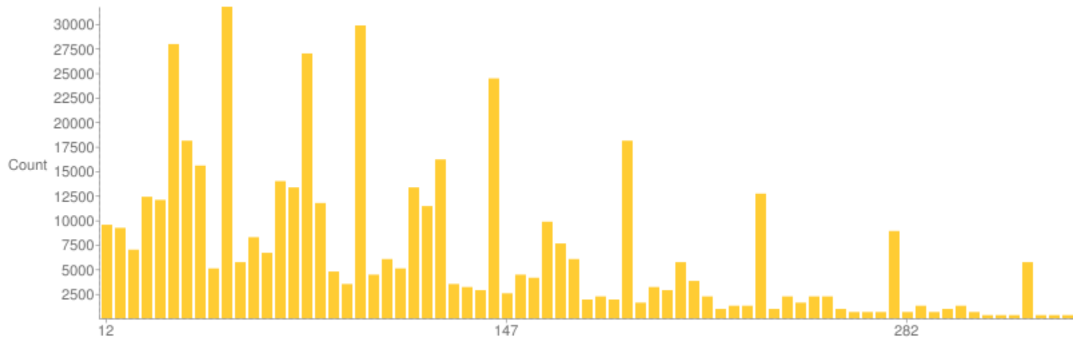

Insertions and deletions length:

**Min**	0
**Max**	227
**Mean**	2.137
**Median**	1
**Standard deviation**	5.74
**Values**	0,1,2,3,4,5,6,7,8,9,10,11,12,13,14,15,16,17,18,19,20,21,22,23,24,25,26,27,28,29,30,31,32,33,34,35,36,37,38,39,40,41,42,43,44,45,46,47,48,49,50,51
**Count**	130674,309832,18999,13066,7373,6004,3857,3871,3409,3288,2729,2777,1983,1688,1529,1388,1132,1119,936,903,861,708,606,561,510,483,496,4

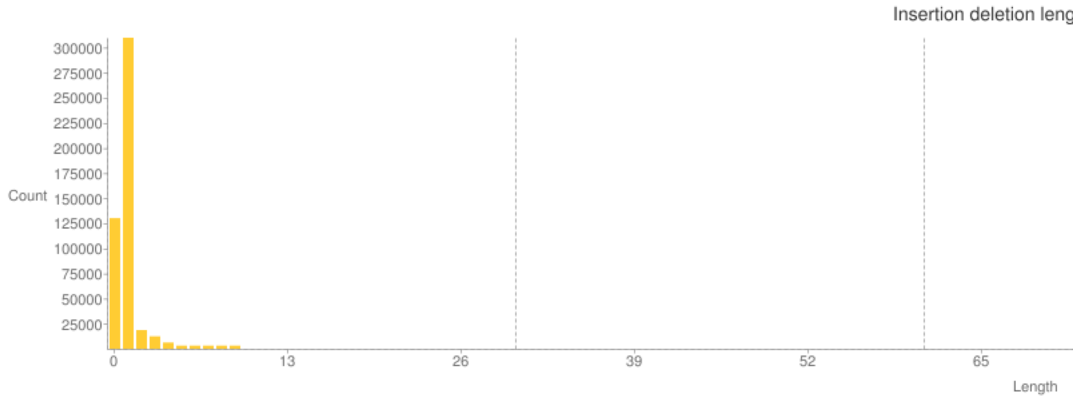

Base changes (SNPs)

	A	C	G	T
**A**	0	0	0	0
**C**	0	0	0	0
**G**	0	0	0	0
**T**	0	0	0	0

Ts/Tv (transitions / transversions)

Note: Only SNPs are used for this statistic.
Note: This Ts/Tv ratio is a 'raw' ratio (ratio of observed events).

**Transitions**	0
**Transversions**	0
**Ts/Tv ratio**	0

All variants:

`No results available (empty input?)`

Only known variants (i.e. the ones having a non-empty ID field):

`No results available (empty input?)`

Allele frequency

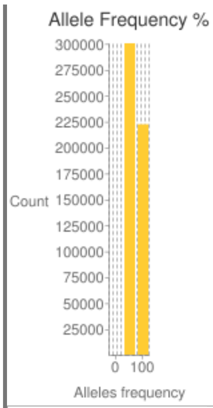

**Min**	0
**Max**	100
**Mean**	71.296
**Median**	50
**Standard deviation**	24.778
**Values**	0,50,100
**Count**	278,300468,223610

Allele Count

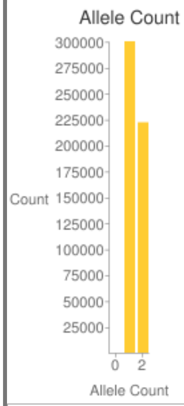

**Min**	0
**Max**	2
**Mean**	1.426
**Median**	1
**Standard deviation**	0.496
**Values**	0,1,2
**Count**	278,300468,223610

Hom/Het per sample

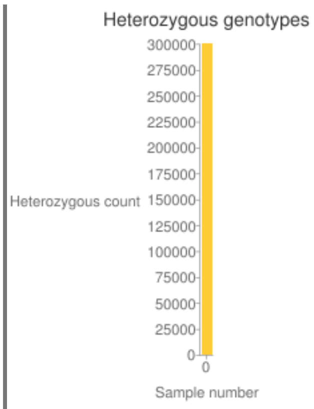

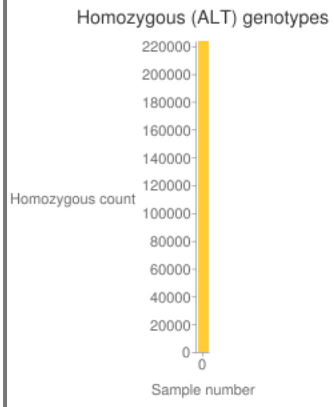

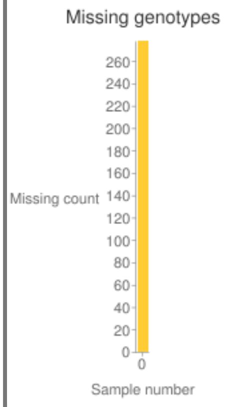


```
Sample_names , readname
Reference , 0
Het , 300468
Hom , 223610
Missing , 278
```

Codon changes

How to read this table:
- Rows are reference codons and columns are changed codons. E.g. Row 'AAA' column 'TAA' indicates how many 'AAA' codons have been replaced by 'TAA' codons.
- Red background colors indicate that more changes happened (heat-map).
- Diagonals are indicated using grey background color
- WARNING: This table may include different translation codon tables (e.g. mamalian DNA and mitochondrial DNA).

	-	AAA	AAC	AAG	AAT	ACA	ACC	ACG	ACT	AGA	AGC	AGG	AGT	ATA	ATC	ATG	ATT	CAA	CAC	CAG	CAT	CCA	CC[illegible]
**-**		216	118	172	215	112	82	39	119	144	93	85	111	94	105	123	131	163	57	119	139	188	5
**AAA**	404	**57**	15	43	16	2		1	1	14	1	4	2	7	2	5	3	6		3		1	
**AAC**	239	21	**19**	12	21	5	3	2	5	5	2	1	2	3	2	4	1			1		1	
**AAG**	499	68	14	**29**	13	3	3	5	1	6	2	5	3	1	1	4	3	7		1		1	
**AAT**	392	51	13	19	**21**	6	2	1	4	8	2	2	5	5	1	8	2			1	2	1	
**ACA**	231	4	10	3		**20**	11	5	17	3	5	2	4	4	2	2	5	3	1				
**ACC**	153	3	15	4		6	**11**	5	12	1	1		2	1	4	2	1		2			1	
**ACG**	82	2	3	1		6	6	**11**	8	1	1	1	2	1		1	1	1					
**ACT**	221	3	21	3	4	18	13	1	**11**	2	4	1	6	3	5		4	1	2			1	
**AGA**	238	7	1	21	1	3	1			**20**	6	17	16	1	1	4	3						
**AGC**	125	1	4	11	4		1	3	1	12	**7**	5	13	2		1	2			2			
**AGG**	164	4	2	14	2		1	4		22	3	**16**	13	2	1	5	2						
**AGT**	227	2	1	11	3	3		1	7	13	7	11	**9**	2		2	2			1	1		
**ATA**	161	3		1	10	5	3	2	4	4	1	1	3	**4**	7	9	16	1			2	1	
**ATC**	131	5	2	4	6	2	5	1	2	2	1		2	6	**9**	5	14		1		3		
**ATG**	302	5	1	9	31	6		3	3	3			3	19	6	**22**	13				1		
**ATT**	246	3	2	2	18	1	6	1	8		2		4	10	16	11	**12**				2		

	-	AAA	AAC	AAG	AAT	ACA	ACC	ACG	ACT	AGA	AGC	AGG	AGT	ATA	ATC	ATG	ATT	CAA	CAC	CAG	CAT	CCA	CC[illegible]
**CAA**	304	1		2		9				1						1	1	**28**	11	24	15	10	
**CAC**	126					2												8	**1**	10	11	3	
**CAG**	221				2	1		2										29	6	**15**	17	12	
**CAT**	221				2										1			17	18	7	**9**	15	
**CCA**	274						3				1							5	1	1	4	**15**	2
**CCC**	117		2		1		2											3	2		1	5	**15**
**CCG**	90													1					2	2		15	1
**CCT**	257			1			1			1								2	2	5	4	25	2
**CGA**	70							1										5		3		2	
**CGC**	53																	1	3	2			
**CGG**	43		1												1			3		5		1	
**CGT**	109		1	1									1					2		1	7	1	
**CTA**	120								2									1	1	2	4	2	
**CTC**	155	1							2									3	3		2	2	
**CTG**	151								2									2	2	3	5		
**CTT**	290		2		1				1		1	1			1			4	2	1	8	1	
**GAA**	493			2						7			1	1			1				1		
**GAC**	203		3	1	1					6						1					1		
**GAG**	387	1		9	1	1	1			6		1		1							1	1	
**GAT**	438			3	2					3		1				1				1	2		
**GCA**	265	1	1	2							2					1				1	1		
**GCC**	145		1	1			2				2											1	
**GCG**	71			1																1			
**GCT**	312			1					1		6					1				1			
**GGA**	308	1								2		7		1		1	1	1					
**GGC**	172			3	2							3	1			1							
**GGG**	181				1							4	1								1		
**GGT**	291				1							3	1	1							1		
**GTA**	139			1																			
**GTC**	116	1		2									1										
**GTG**	202											1	2			1							
**GTT**	270			1	1				1	1			4		1	1				1		1	
**TAA**	23				1									2									
**TAC**	107			1	3													1		1			
**TAG**	18																						
**TAT**	208										1			1			1	1					
**TCA**	299														1		1					2	
**TCC**	155	1			1										1		1		1	1			
**TCG**	95				1										1								
**TCT**	372	1			1	1	1		2						3								
**TGA**	28					1										1							
**TGC**	96	1	1													2							
**TGG**	140															6							
**TGT**	144	1		1	1					1	1										1		
**TTA**	217		2	1					1								3						
**TTC**	226																1	1			1		
**TTG**	298								1								5						
**TTT**	341				2												4						

Amino acid changes

How to read this table:
- Rows are reference amino acids and columns are changed amino acids. E.g. Row 'A' column 'E' indicates how many 'A' amino acids have been replaced by 'E' amino acids.
- Red background colors indicate that more changes happened (heat-map).
- Diagonals are indicated using grey background color
- WARNING: This table may include different translation codon tables (e.g. mamalian DNA and mitochondrial DNA).

	*	-	?	A	C	D	E	F	G	H	I	K	L	M	N	P	Q	R	S	T	V	W	Y
*****	**15**	67	2		3						2		10	1	1				8	1		2	5
**-**	160		3,520	358	155	351	451	266	514	196	330	388	563	123	333	427	282	366	740	352	389	71	181
**?**																							
**A**	2	793		**166**	5	24	27	1	52	1		6	3	2	2	1	3	1	14	3	44	1	
**C**	6	240		1	**17**			12		1		3	14	2	2			3	20		3	12	4
**D**	7	641		23	1	**88**	95		41	3		4	3	2	6	1	1	13	2		23		2
**E**	8	880		25		91	**160**		74	2	3	12	2		1	1		18	3	2	30	2	1
**F**	9	567		2	13			**114**		1	5		36		2	1	1	1	46		12	4	17
**G**	1	952		26	3	30	61		**271**	2	3	4	1	2	4	3	1	25	6		37	4	
**H**		347			1	4	2	1	3	**39**	1		19		2	30	42	14	3	2			1
**I**	3	538		2	1	3	3	1	2	8	**94**	18	6	25	38	2	1	7	15	40	2	2	8
**K**	11	903		2		2	20	3	4		17	**197**	3	9	58	2	17	32	9	16	2		
**L**	10	1,231		9	8	2	4	101	1	27	9	2	**233**		5	50	16	18	45	9	12	3	14
**M**	2	302				3	1		2	1	38	14	2	**22**	32			3	6	12	3		5
**N**	9	631		4			9	1	6	2	14	103	2	12	**74**	3	2	18	13	28	1	1	1

	*	-	?	A	C	D	E	F	G	H	I	K	L	M	N	P	Q	R	S	T	V	W	Y
**P**		738		14	1			4		16	1	1	46		3	**241**	18	23	19	6	2	1	
**Q**		525		10	1		4	4	1	49	1	3	23	1	2	34	**96**	24	8	12			1
**R**	1	677		7	1	1	5	2	4	10	11	47	23	9	8	23	22	**129**	41	10	1		
**S**	16	1,273		5	17	2	4	75	6	2	16	27	29	3	15	8	4	41	**297**	20	9	2	12
**T**	1	687		3	2	5	3	2		5	31	23	2	5	53	5	5	12	25	**161**	1		2
**V**	1	727		43	8	37	36	3	53		1	5	6	2	1	3	1	5	8	1	**148**	4	
**W**	6	140			13		1						10	6					7		1	**12**	2
**Y**	31	315		1	9		5	4	2		2	1	26		3		3	1	18		6	3	**33**

Variants by chromosome

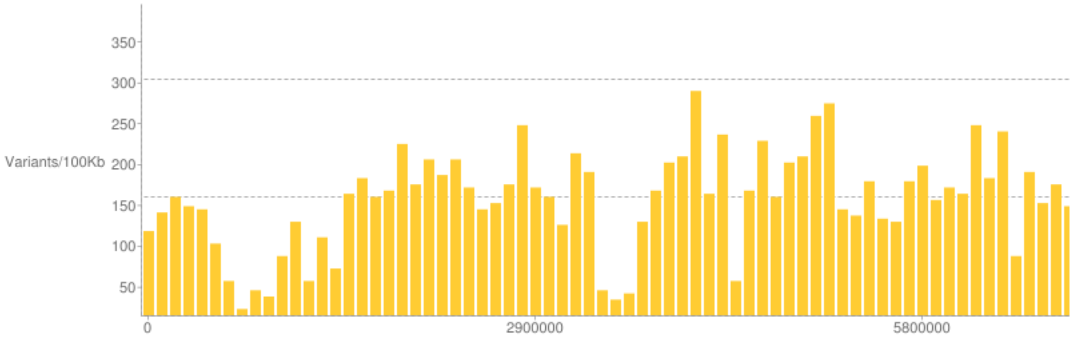

1, Position,0,100000,200000,300000,400000,500000,600000,700000,800000,900000,1000000,1100000,1200000,1300000,1400000,
1,Count,119,144,162,151,148,106,57,23,46,39,91,130,58,113,75,164,184,163,170,227,176,206,187,209,173,148,155,178,251,172,163,126,215,

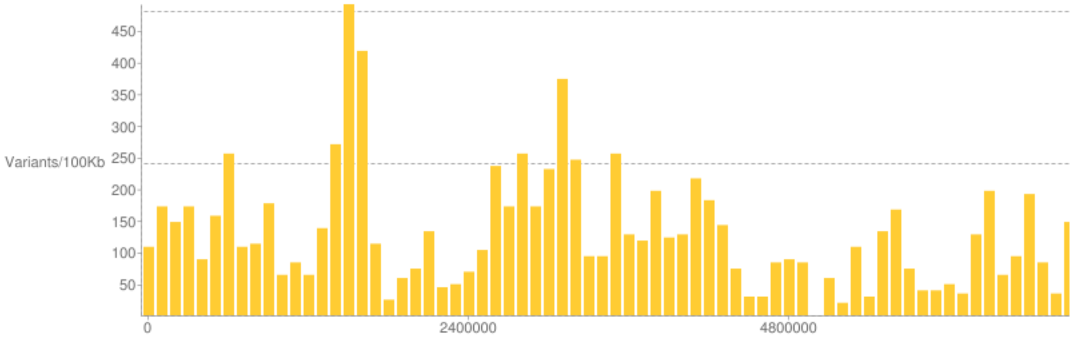

2, Position,0,100000,200000,300000,400000,500000,600000,700000,800000,900000,1000000,1100000,1200000,1300000,1400000,
2,Count,111,173,151,173,94,163,260,113,114,181,68,86,66,140,275,492,420,114,29,64,78,135,47,52,71,107,239,173,257,175,233,377,248,97,

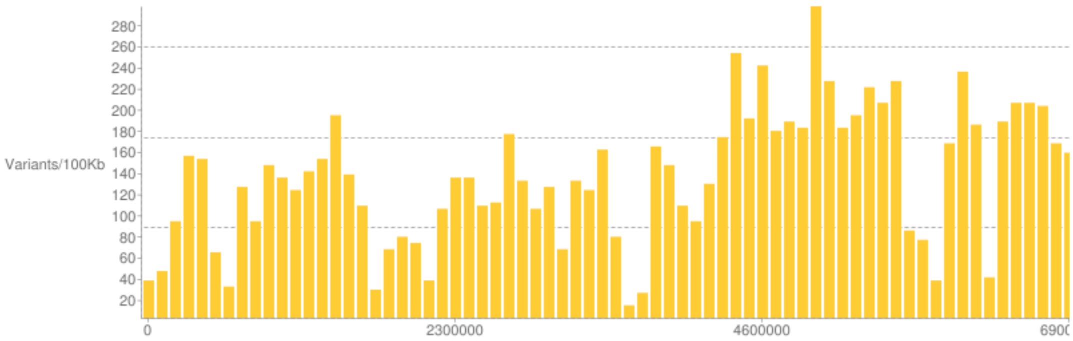

3, Position,0,100000,200000,300000,400000,500000,600000,700000,800000,900000,1000000,1100000,1200000,1300000,1400000,
3,Count,40,49,97,157,154,65,33,128,97,149,137,124,142,156,195,139,112,30,70,82,76,41,109,138,137,111,114,178,135,108,129,70,134,124,

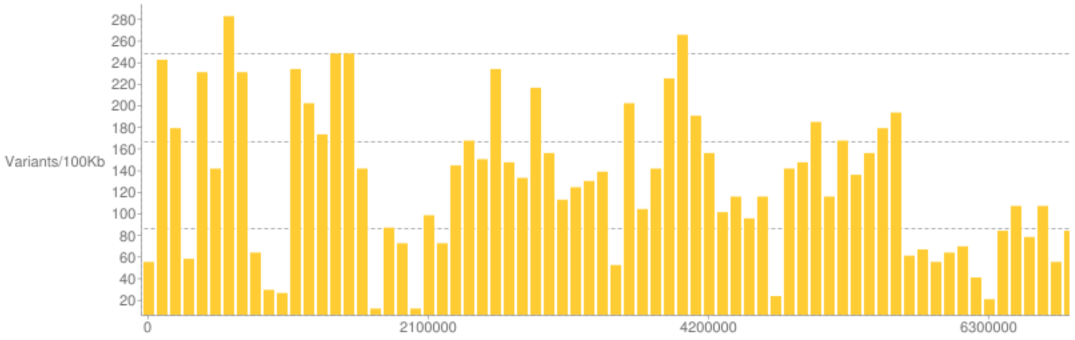

4, Position,0,100000,200000,300000,400000,500000,600000,700000,800000,900000,1000000,1100000,1200000,1300000,1400000,
4,Count,57,244,180,59,231,143,284,232,64,30,28,235,204,174,250,250,142,14,89,75,13,99,74,146,169,150,235,148,133,218,157,115,126,130,

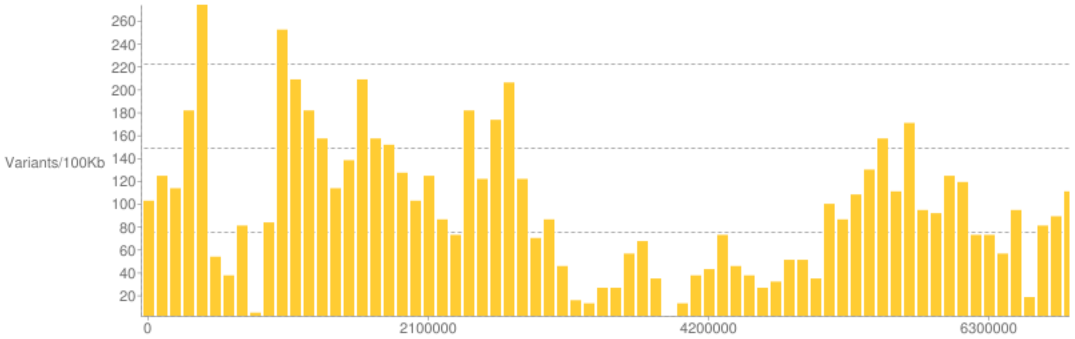

5, Position,0,100000,200000,300000,400000,500000,600000,700000,800000,900000,1000000,1100000,1200000,1300000,1400000,
5,Count,105,125,114,182,274,56,38,81,6,84,254,210,182,159,115,140,209,158,152,128,103,125,89,73,182,122,175,208,123,70,87,46,16,14,28,

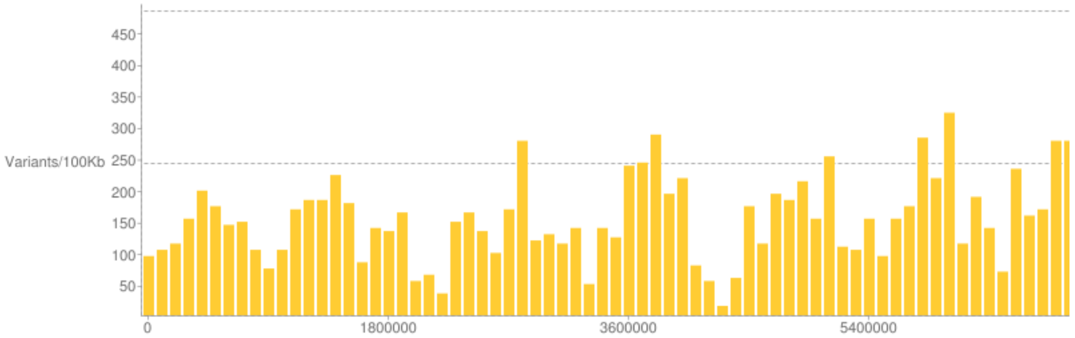

6, Position,0,100000,200000,300000,400000,500000,600000,700000,800000,900000,1000000,1100000,1200000,1300000,1400000,
6,Count,98,111,120,160,201,176,151,154,109,82,107,172,190,190,229,183,87,143,139,170,62,70,41,156,167,137,103,173,280,124,135,119,145,

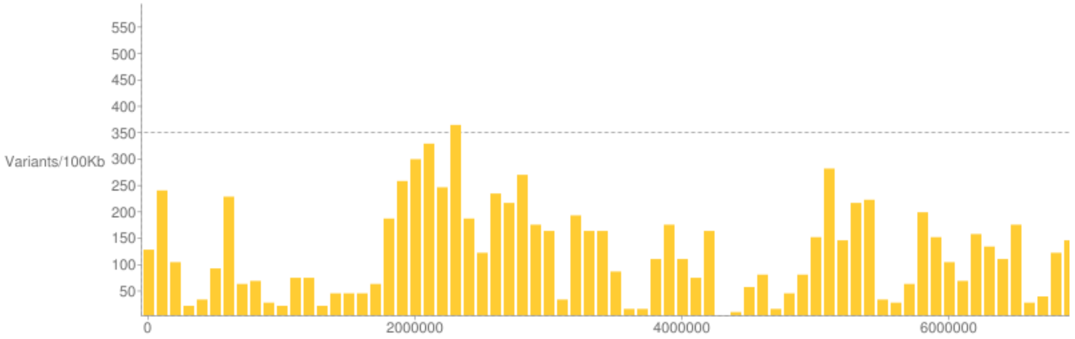

7, Position,0,100000,200000,300000,400000,500000,600000,700000,800000,900000,1000000,1100000,1200000,1300000,1400000,
7,Count,130,240,108,26,33,94,233,68,71,31,26,77,74,21,46,48,45,64,188,259,301,329,247,364,187,124,237,217,273,175,168,34,194,165,168,

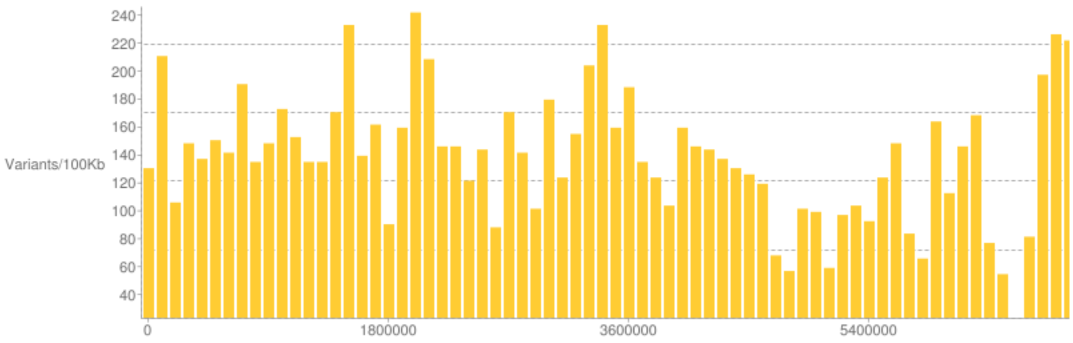

8, Position,0,100000,200000,300000,400000,500000,600000,700000,800000,900000,1000000,1100000,1200000,1300000,1400000,
8,Count,131,212,107,149,138,151,142,191,135,149,173,154,136,135,172,234,141,163,92,161,243,210,146,146,123,144,89,171,143,103,181,125,

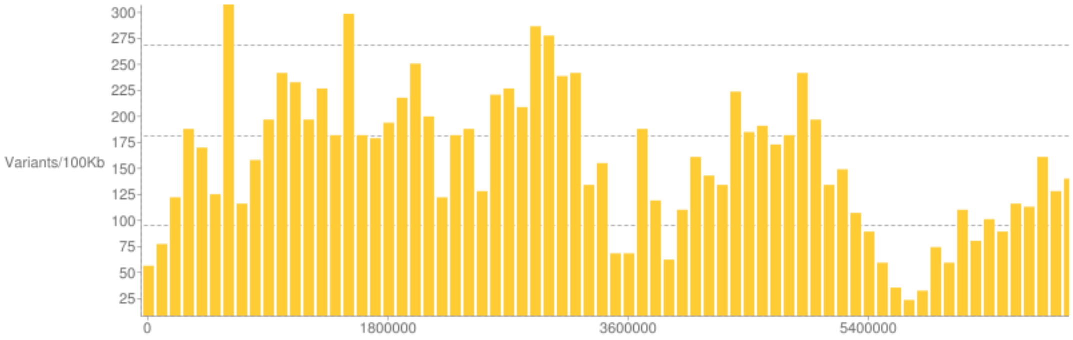

9, Position,0,100000,200000,300000,400000,500000,600000,700000,800000,900000,1000000,1100000,1200000,1300000,1400000,
9,Count,57,78,124,189,170,127,307,116,159,197,243,233,197,227,183,301,183,180,196,220,253,200,123,184,190,129,223,229,210,289,280,239,

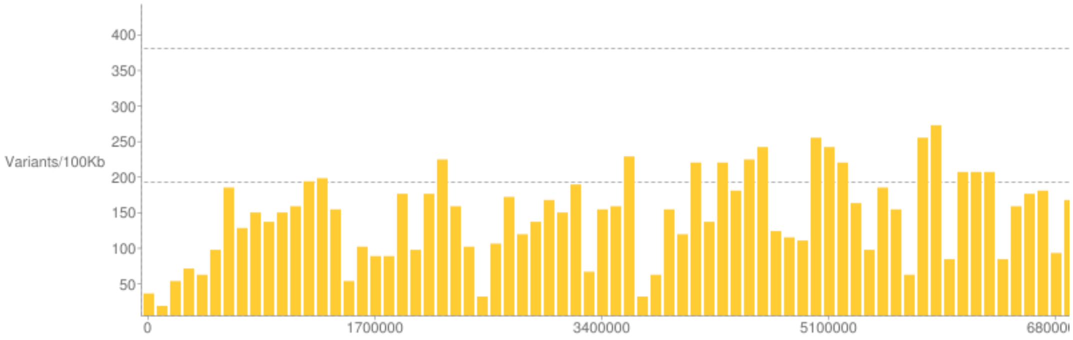

10, Position,0,100000,200000,300000,400000,500000,600000,700000,800000,900000,1000000,1100000,1200000,1300000,1400000,
10,Count,39,22,57,74,63,99,188,128,152,139,152,161,195,198,158,55,103,92,91,180,101,178,224,162,103,35,106,172,121,138,168,152,191,68,

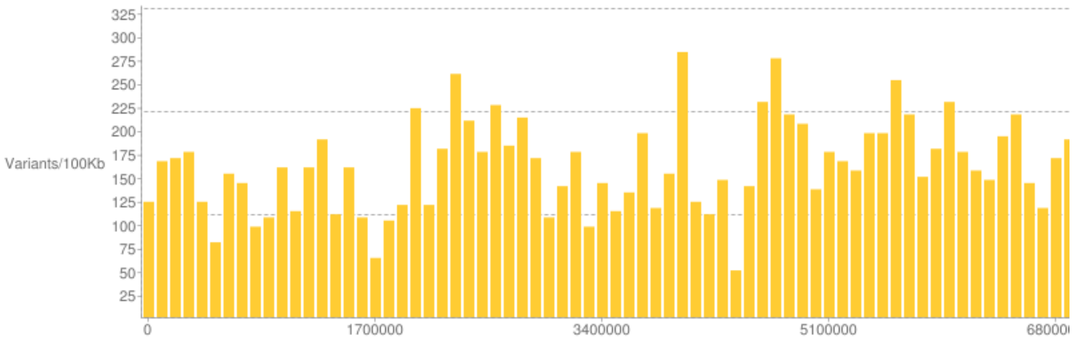

11, Position,0,100000,200000,300000,400000,500000,600000,700000,800000,900000,1000000,1100000,1200000,1300000,1400000,
11,Count,128,168,173,181,125,83,155,145,100,111,162,116,164,193,112,163,109,68,106,124,227,124,182,263,213,179,230,185,217,173,110,14[illegible]

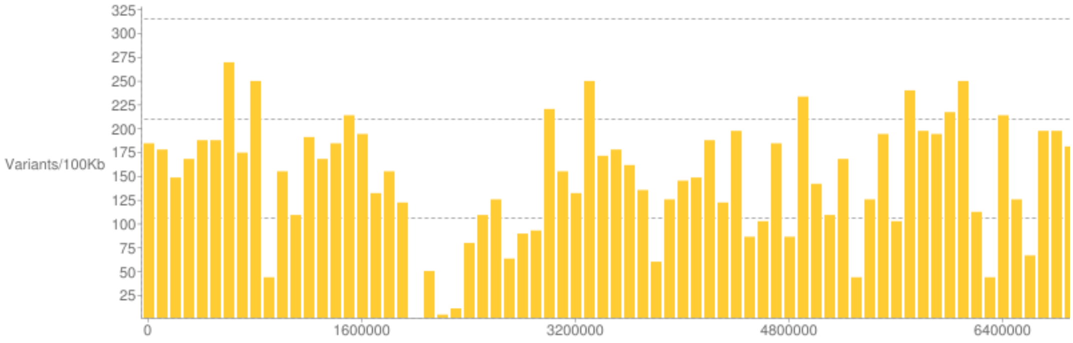

12, Position,0,100000,200000,300000,400000,500000,600000,700000,800000,900000,1000000,1100000,1200000,1300000,1400000,
12,Count,185,179,151,168,190,189,271,176,252,46,157,111,191,170,186,215,197,132,156,124,1,51,5,11,80,110,127,64,90,95,223,155,132,250,

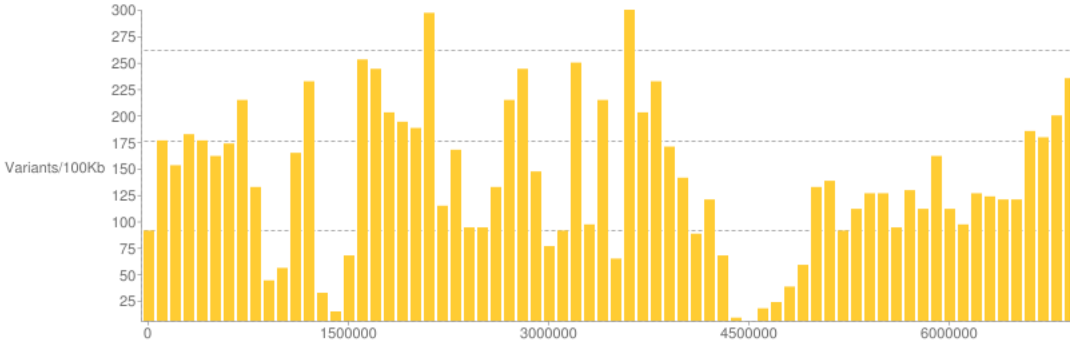

13, Position,0,100000,200000,300000,400000,500000,600000,700000,800000,900000,1000000,1100000,1200000,1300000,1400000,
13,Count,93,178,155,183,178,162,176,215,134,47,58,165,233,34,16,70,253,245,204,196,190,298,116,170,96,95,134,215,246,149,78,94,251,10[illegible]

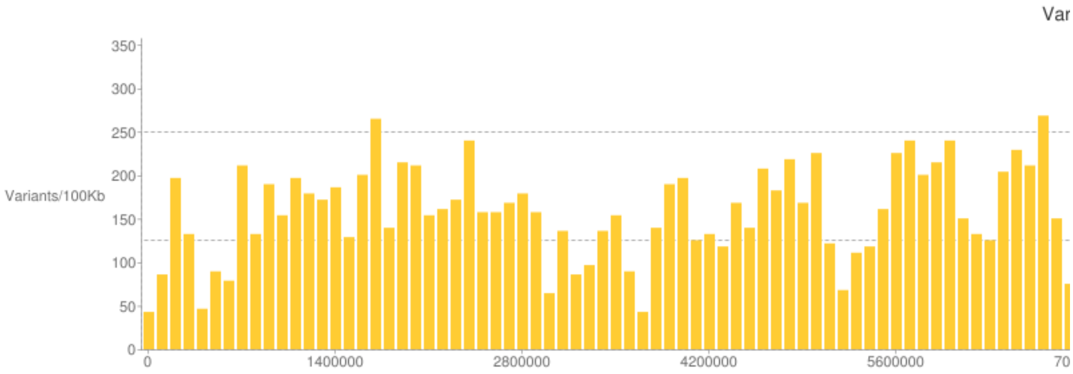

14, Position,0,100000,200000,300000,400000,500000,600000,700000,800000,900000,1000000,1100000,1200000,1300000,1400000,
14,Count,46,89,199,135,50,90,82,214,133,192,156,198,181,174,189,132,202,266,141,216,212,155,163,175,242,161,161,170,182,160,65,138,86,

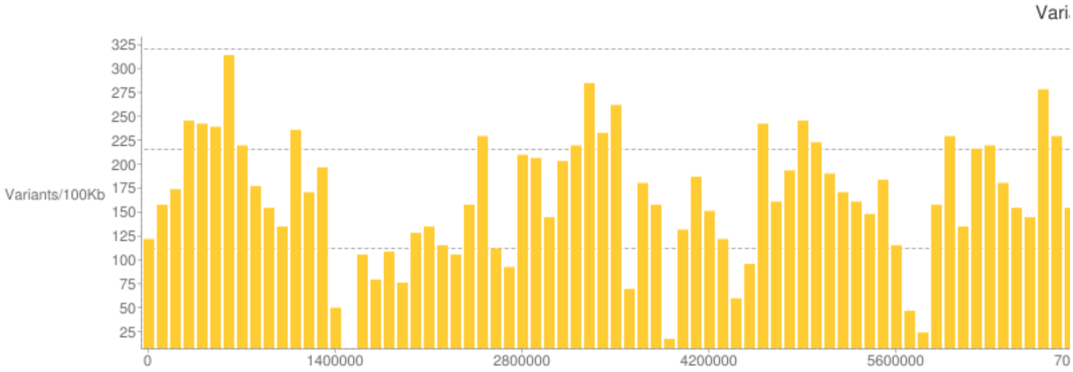

15, Position,0,100000,200000,300000,400000,500000,600000,700000,800000,900000,1000000,1100000,1200000,1300000,1400000,
15,Count,124,159,176,247,243,241,314,221,178,154,136,238,170,199,50,7,106,81,111,76,128,137,117,108,160,230,114,92,211,207,146,204,22[illegible]

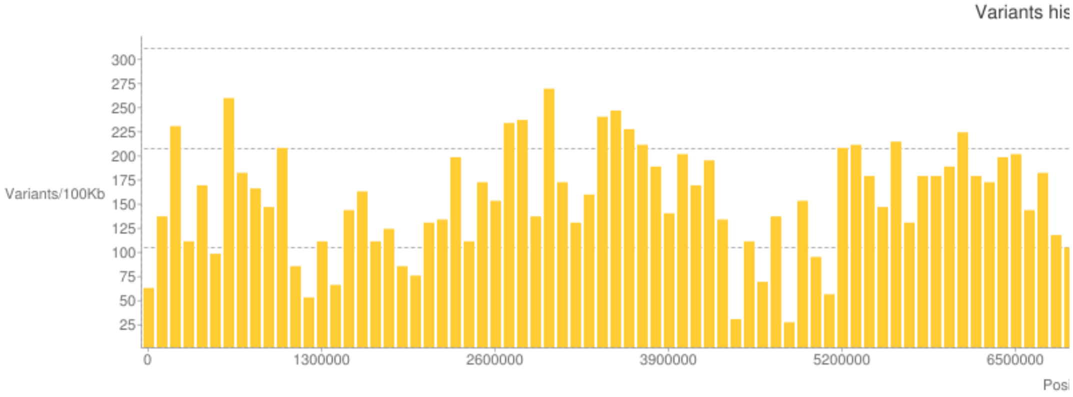

16, Position,0,100000,200000,300000,400000,500000,600000,700000,800000,900000,1000000,1100000,1200000,1300000,1400000,
16,Count,64,138,232,113,171,101,261,183,167,147,208,85,54,112,66,145,163,113,126,87,77,131,134,199,111,175,156,235,239,139,271,175,13

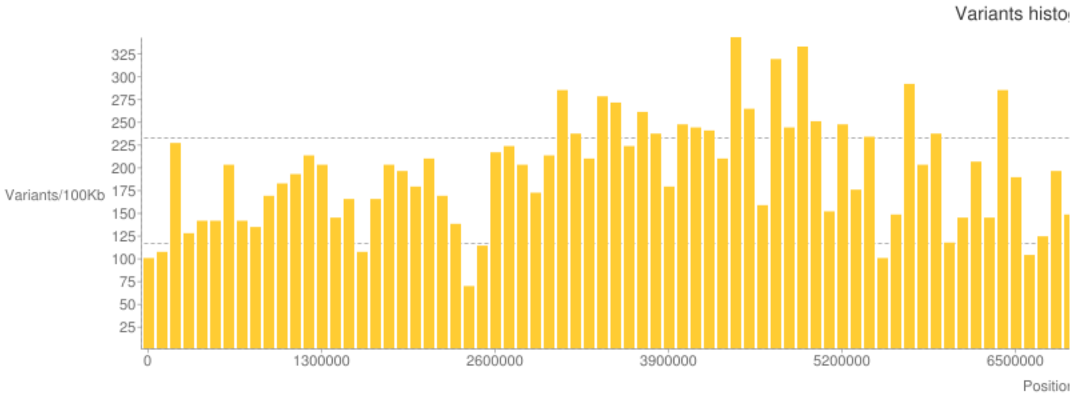

17, Position,0,100000,200000,300000,400000,500000,600000,700000,800000,900000,1000000,1100000,1200000,1300000,1400000,
17,Count,101,108,228,130,143,144,203,144,137,169,183,195,215,206,145,166,109,168,204,199,180,210,170,140,72,115,219,226,206,173,215,2

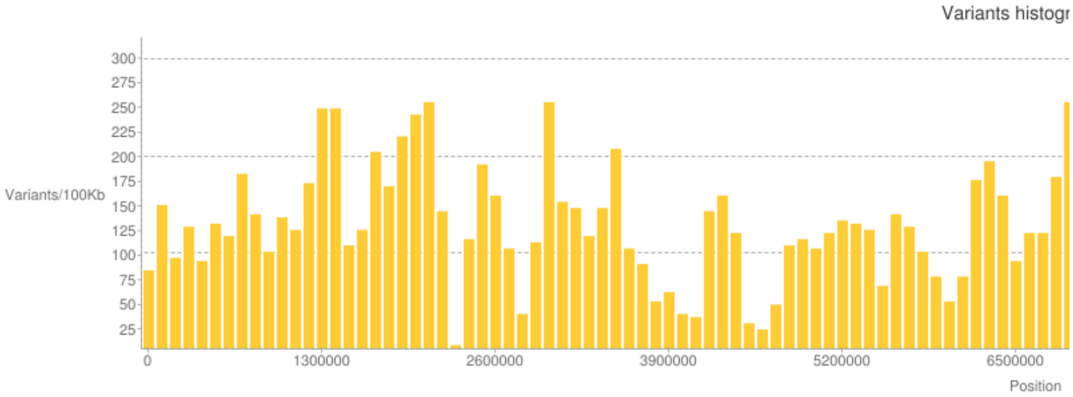

18, Position,0,100000,200000,300000,400000,500000,600000,700000,800000,900000,1000000,1100000,1200000,1300000,1400000,
18,Count,87,151,97,130,96,133,119,183,144,104,138,128,175,249,251,112,128,206,171,222,245,256,145,9,116,192,161,109,41,113,256,156,14

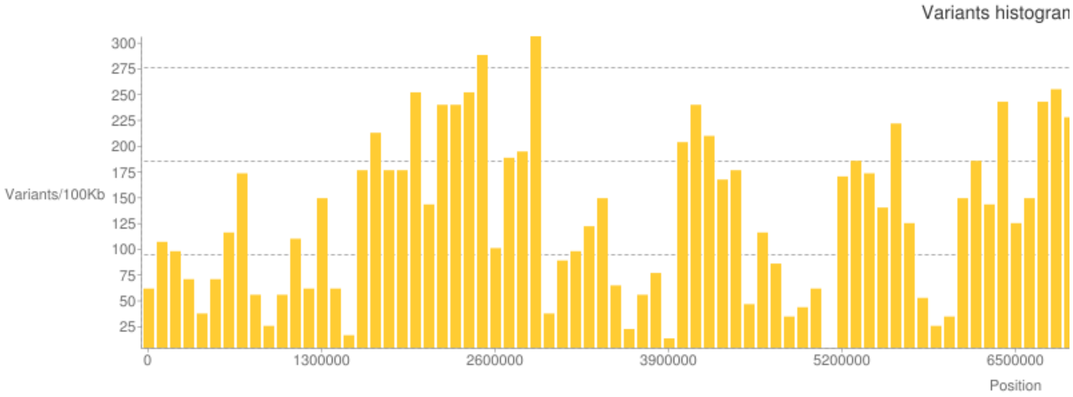

19, Position,0,100000,200000,300000,400000,500000,600000,700000,800000,900000,1000000,1100000,1200000,1300000,1400000,
19,Count,64,109,98,73,40,73,118,175,57,26,58,112,64,151,62,18,178,215,178,179,253,143,241,241,254,289,101,189,196,306,39,91,100,122,1

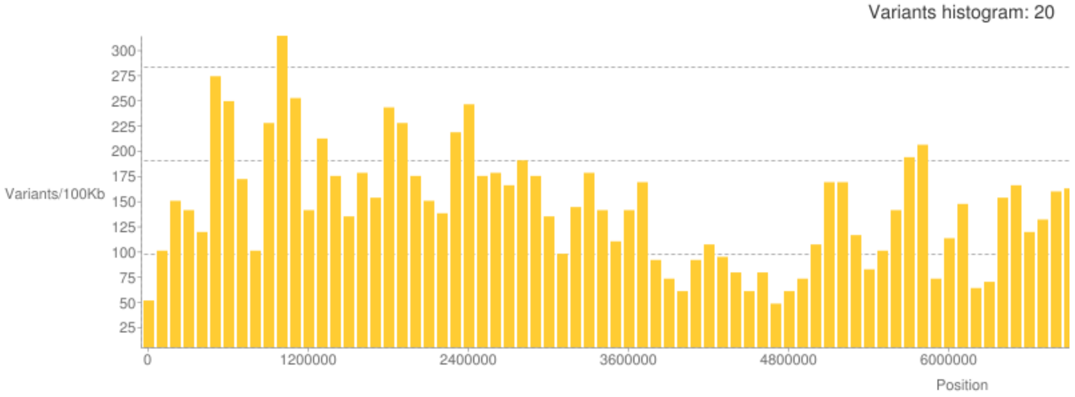


```
20, Position,0,100000,200000,300000,400000,500000,600000,700000,800000,900000,1000000,1100000,1200000,1300000,1400000,
20,Count,53,103,153,142,121,274,252,174,103,228,314,255,144,214,178,136,181,155,245,229,176,153,138,219,249,177,180,166,193,177,136,9
```

Details by gene

Here you can find a tab-separated table.

SnpEff: Variant analysis

Contents

Summary
Variant rate by chromosome
Variants by type
Number of variants by impact
Number of variants by functional class
Number of variants by effect
Quality histogram
InDel length histogram
Base variant table
Transition vs transversions (ts/tv)
Allele frequency
Allele Count
Codon change table
Amino acid change table
Chromosome variants plots
Details by gene

Summary

**Genome**	manindi
**Date**	2021-05-22 14:07
**SnpEff version**	`SnpEff 5.0e (build 2021-03-09 06:01), by Pablo Cingolani`
**Command line arguments**	`SnpEff  manindi /home/cocogenomics/mango_genome/mango_ref_genome/M.altissima_ref_SNP_final.vcf`
**Warnings**	317,354
**Errors**	0
**Number of lines (input file)**	2,910,228
**Number of variants (before filter)**	2,918,359
**Number of not variants (i.e. reference equals alternative)**	0
**Number of variants processed (i.e. after filter and non-variants)**	2,918,359
**Number of known variants (i.e. non-empty ID)**	0 (0%)
**Number of multi-allelic VCF entries (i.e. more than two alleles)**	8,131
**Number of effects**	6,054,860
**Genome total length**	391,108,416
**Genome effective length**	357,440,369
**Variant rate**	1 variant every 122 bases

Variants rate details

Chromosome	Length	Variants	Variants rate
1	29,456,600	247,631	118
2	24,397,897	196,725	124
3	23,139,393	153,690	150
4	21,507,500	169,563	126
5	21,083,371	148,062	142
6	18,811,960	158,950	118
7	20,623,120	179,505	114
8	18,243,469	140,170	130
9	18,233,274	181,109	100
10	17,652,500	144,538	122
11	17,144,574	124,276	137
12	16,029,966	133,445	120
13	15,457,994	126,716	121
14	14,810,209	111,248	133
15	14,765,960	141,677	104
16	13,817,227	109,061	126
17	13,506,273	122,573	110
18	13,371,754	121,716	109
19	13,093,066	118,198	110
20	12,294,262	89,506	137
**Total**	**357,440,369**	**2,918,359**	**122**

Number variants by type

Type	Total
**SNP**	2,918,359
**MNP**	0
**INS**	0
**DEL**	0
**MIXED**	0
**INV**	0
**DUP**	0
**BND**	0
**Total**	**2,918,359**

Type	Total
INTERVAL	0
Total	2,918,359

Number of effects by impact

Type (alphabetical order)	Count	Percent
HIGH	3,340	0.055%
LOW	75,878	1.253%
MODERATE	91,959	1.519%
MODIFIER	5,883,683	97.173%

Number of effects by functional class

Type (alphabetical order)	Count	Percent
MISSENSE	92,524	58.362%
NONSENSE	1,887	1.19%
SILENT	64,125	40.448%

Missense / Silent ratio: 1.4429

Number of effects by type and region

Type

Type (alphabetical order)	Count	Percent
3_prime_UTR_variant	26,993	0.445%
5_prime_UTR_premature_start_codon_gain_variant	2,351	0.039%
5_prime_UTR_variant	14,370	0.237%
downstream_gene_variant	1,501,470	24.745%
initiator_codon_variant	24	0%
intergenic_region	2,310,351	38.076%
intron_variant	412,723	6.802%
missense_variant	91,959	1.516%
splice_acceptor_variant	494	0.008%
splice_donor_variant	406	0.007%
splice_region_variant	12,542	0.207%
start_lost	214	0.004%
start_retained_variant	9	0%
stop_gained	1,887	0.031%
stop_lost	339	0.006%
stop_retained_variant	154	0.003%
synonymous_variant	63,950	1.054%
upstream_gene_variant	1,627,526	26.823%

Region

Type (alphabetical order)	Count	Percent
DOWNSTREAM	1,501,470	24.798%
EXON	157,292	2.598%
INTERGENIC	2,310,351	38.157%
INTRON	402,973	6.655%
SPLICE_SITE_ACCEPTOR	494	0.008%
SPLICE_SITE_DONOR	406	0.007%
SPLICE_SITE_REGION	10,634	0.176%
UPSTREAM	1,627,526	26.88%
UTR_3_PRIME	26,993	0.446%
UTR_5_PRIME	16,721	0.276%

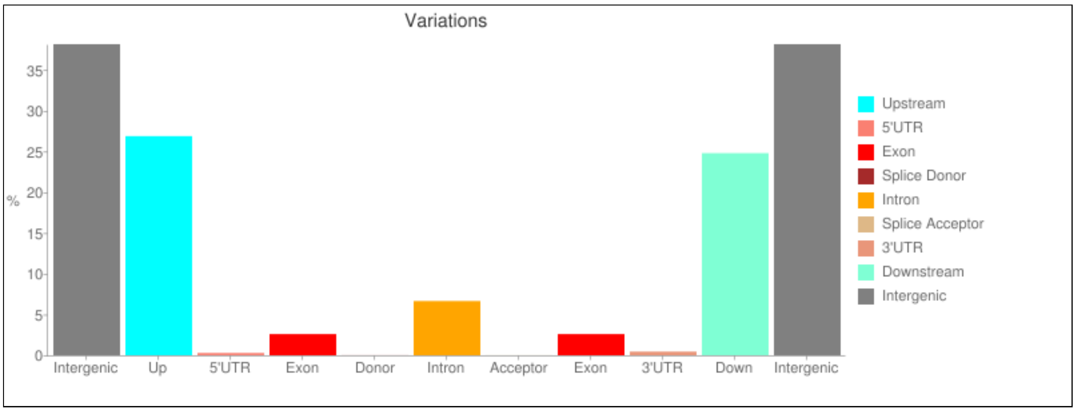

Quality:

**Min**	10
**Max**	63,368
**Mean**	143.742
**Median**	110
**Standard deviation**	344.415
**Values**	10,11,12,13,14,15,16,17,18,19,20,21,22,23,24,25,26,27,28,29,30,31,32,33,34,35,36,37,38,39,40,41,42,43,44,45,46,47,48,49,50,51,52,53,54,55,56,57
**Count**	2347,1823,14107,4829,5036,4114,6845,9032,12580,12961,17127,15371,19571,19162,20264,25328,21150,17848,16623,9757,11189,13703,11230,1[illegible]

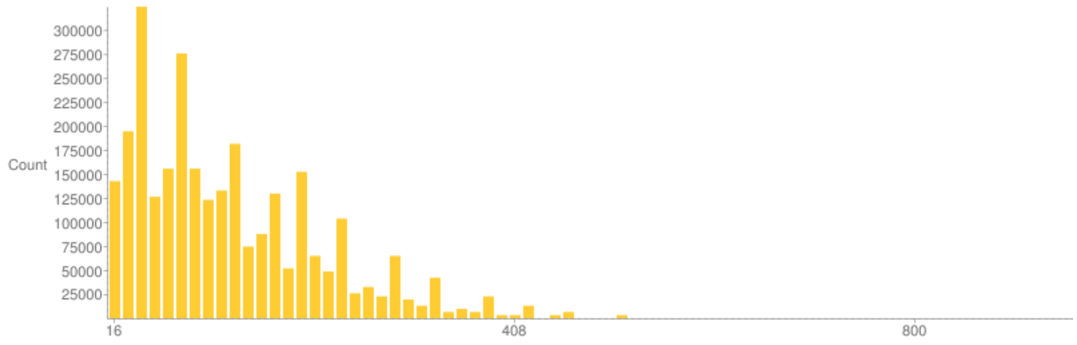

Insertions and deletions length:

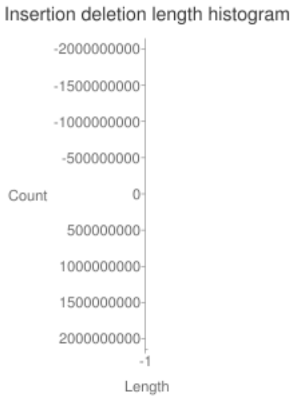

Base changes (SNPs)

	A	C	G	T
A	0	101,146	485,203	156,614
C	109,276	0	67,980	539,147
G	540,010	68,236	0	109,725
T	155,724	484,498	100,800	0

Ts/Tv (transitions / transversions)

Note: Only SNPs are used for this statistic.
Note: This Ts/Tv ratio is a 'raw' ratio (ratio of observed events).

Transitions	2,811,798
Transversions	1,184,169
Ts/Tv ratio	2.3745

All variants:

```
Sample ,readname,Total
Transitions ,2811798,2811798
Transversions ,1184169,1184169
Ts/Tv ,2.374,2.374
```

Only known variants (i.e. the ones having a non-empty ID field):

```
No results available (empty input?)
```

Allele frequency

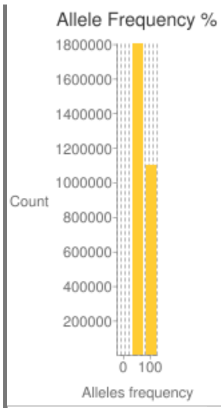

**Min**	0
**Max**	100
**Mean**	68.933
**Median**	50
**Standard deviation**	24.327
**Values**	0,50,100
**Count**	2106,1804015,1104107

Allele Count

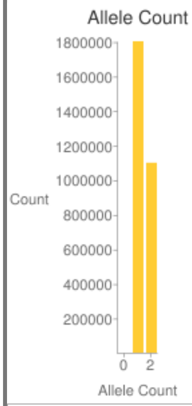

**Min**	0
**Max**	2
**Mean**	1.379
**Median**	1
**Standard deviation**	0.487
**Values**	0,1,2
**Count**	2106,1804015,1104107

Hom/Het per sample

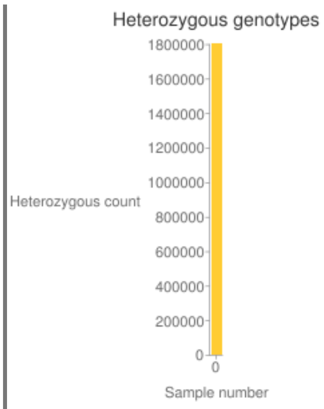

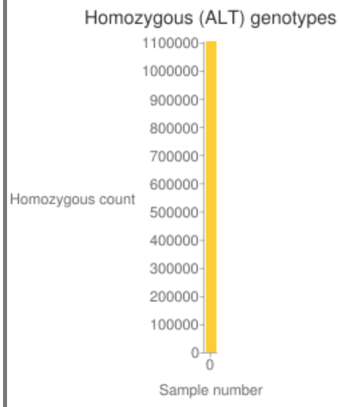

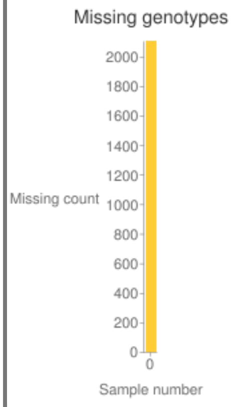


```
Sample_names , readname
Reference , 0
Het , 1804015
Hom , 1104107
Missing , 2106
```

Codon changes

How to read this table:
- Rows are reference codons and columns are changed codons. E.g. Row 'AAA' column 'TAA' indicates how many 'AAA' codons have been replaced by 'TAA' codons.
- Red background colors indicate that more changes happened (heat-map).
- Diagonals are indicated using grey background color
- WARNING: This table may include different translation codon tables (e.g. mamalian DNA and mitochondrial DNA).

	AAA	AAC	AAG	AAT	ACA	ACC	ACG	ACT	AGA	AGC	AGG	AGT	ATA	ATC	ATG	ATT	CAA	CAC	CAG	CAT	CCA	CCC
**AAA**		219	1,133	316	214				636				176				275					
**AAC**	250		223	1,145		102				486				73				88				
**AAG**	1,264	194		292			111				597				217				207			
**AAT**	327	953	273					224				675				194				213		
**ACA**	266					183	648	390	165				475								99	
**ACC**		154			177		89	902		120				245								48
**ACG**			143		904	129		214			85				527							
**ACT**				222	344	673	147					291				592						
**AGA**	704				165					94	481	87	130									
**AGC**		559				126			99		101	722		69								
**AGG**			628				70		632	103		134			105							
**AGT**				696				273	102	587	104					182						
**ATA**	148				407				116					232	541	400						
**ATC**		85				196				62			262		145	956						
**ATG**			195				439				135		735	151		265						
**ATT**				185				473				163	342	843	220							
**CAA**	308																	116	852	184	141	

	AAA	AAC	AAG	AAT	ACA	ACC	ACG	ACT	AGA	AGC	AGG	AGT	ATA	ATC	ATG	ATT	CAA	CAC	CAG	CAT	CCA	CCC
**CAC**		83															117		124	578		41
**CAG**			214														977	119		171		
**CAT**				230													189	462	129			
**CCA**					125												149					152
**CCC**						50												51			161	
**CCG**							24												76		1,080	103
**CCT**								115												113	325	586
**CGA**									199								682				76	
**CGC**										52								308				33
**CGG**											152								523			
**CGT**												113								639		
**CTA**													133				131				296	
**CTC**														96				53				113
**CTG**															163				151			
**CTT**																257				155		
**GAA**	1,064																320					
**GAC**		457																71				
**GAG**			1,007																252			
**GAT**				1,025																222		
**GCA**					778																161	
**GCC**						452																61
**GCG**							174															
**GCT**								811														
**GGA**									539													
**GGC**										390												
**GGG**											361											
**GGT**												675										
**GTA**													582									
**GTC**														518								
**GTG**															584							
**GTT**																1,080						
**TAA**	14																40					
**TAC**		102																197				
**TAG**			10																34			
**TAT**				207																433		
**TCA**					167																385	
**TCC**						75																150
**TCG**							47															
**TCT**								210														
**TGA**									8													
**TGC**										82												
**TGG**											55											
**TGT**												133										
**TTA**													147									
**TTC**														91								
**TTG**															263							
**TTT**																235						

Amino acid changes

How to read this table:
- Rows are reference amino acids and columns are changed amino acids. E.g. Row 'A' column 'E' indicates how many 'A' amino acids have been replaced by 'E' amino acids.
- Red background colors indicate that more changes happened (heat-map).
- Diagonals are indicated using grey background color
- WARNING: This table may include different translation codon tables (e.g. mamalian DNA and mitochondrial DNA).

	*	A	C	D	E	F	G	H	I	K	L	M	N	P	Q	R	S	T	V	W	Y
*	**154**		13		23		9			24	34				74	35	27			53	47
A		**5,717**		235	334		570							374			782	2,215	2,212		
C	43		**1,085**			231	151									486	506			114	593
D		211		**2,419**	1,221		691	293					1,482						193		333
E	167	297		1,155	**2,190**		803			2,071					572				250		
F			209			**2,138**			326		1,395						645		322		363
G	50	527	221	853	911		**4,312**									1,185	1,065		473	68	
H				249				**1,040**			174		313	150	559	679					777
I						361			**3,035**	148	607	906	270			116	225	1,076	1,788		
K	110				1,728				176	**2,397**		217	1,021		482	1,233		325			
L	136					1,692		208	633		**9,880**	426		1,015	282	216	951		966	120	
M									1,151	195	452					135		439	475		
N				1,194				301	267	1,073			**2,098**				1,161	326			278
P		426						164			1,306			**4,663**	225	224	1,240	314			
Q	465				597			590		522	255			222	**1,829**	940					
R	274		661				989	947	130	1,332	302	105		236	1,205	**3,319**	583	235		354	

	*	A	C	D	E	F	G	H	I	K	L	M	N	P	Q	R	S	T	V	W	Y
**S**	141	640	517			804	812		251		1,073		1,255	1,070		545	**6,974**	898		64	287
**T**		1,692							1,312	409		527	376	297		250	941	**4,800**			
**V**		1,660		182	240	394	315		2,180		1,063	584							**4,637**		
**W**	335		128				64				108					248	54				
**Y**	166		557	260		400		630					309				211				**1,438**

Variants by chromosome

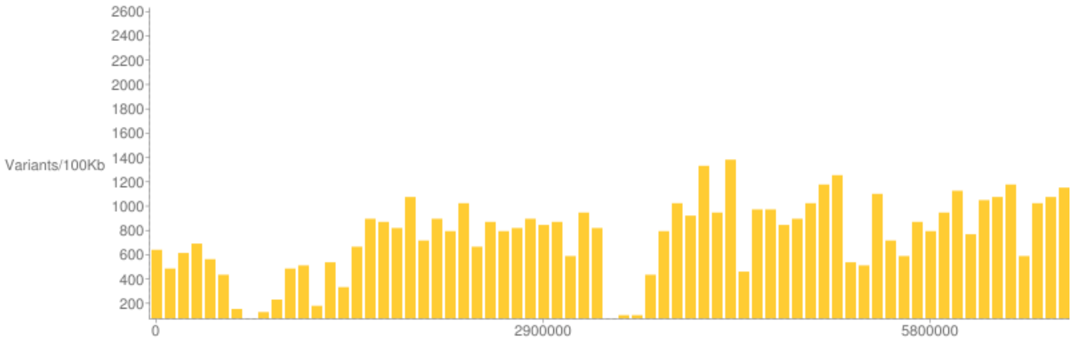


```
1, Position,0,100000,200000,300000,400000,500000,600000,700000,800000,900000,1000000,1100000,1200000,1300000,1400000,
1,Count,657,490,622,698,560,438,168,72,135,234,490,511,178,534,338,686,892,867,838,1080,733,892,807,1028,683,883,811,818,911,840,878,
```

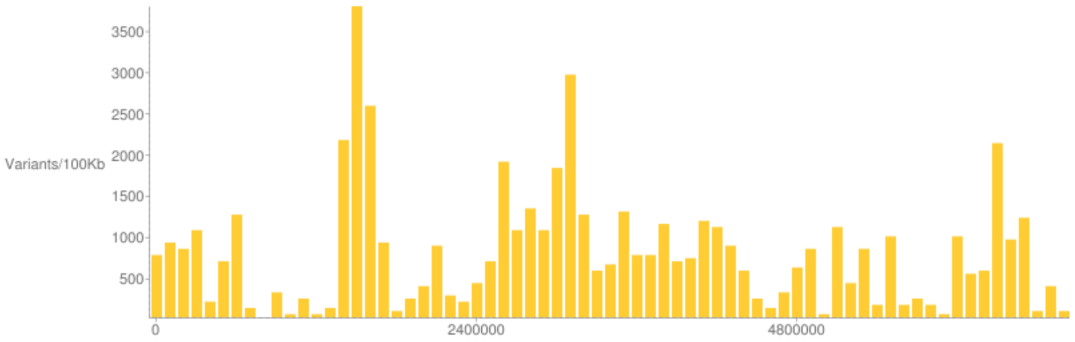


```
2, Position,0,100000,200000,300000,400000,500000,600000,700000,800000,900000,1000000,1100000,1200000,1300000,1400000,
2,Count,818,935,861,1115,242,718,1288,141,62,356,98,273,76,172,2213,3800,2598,942,116,274,417,901,323,225,446,715,1943,1089,1350,1120,
```

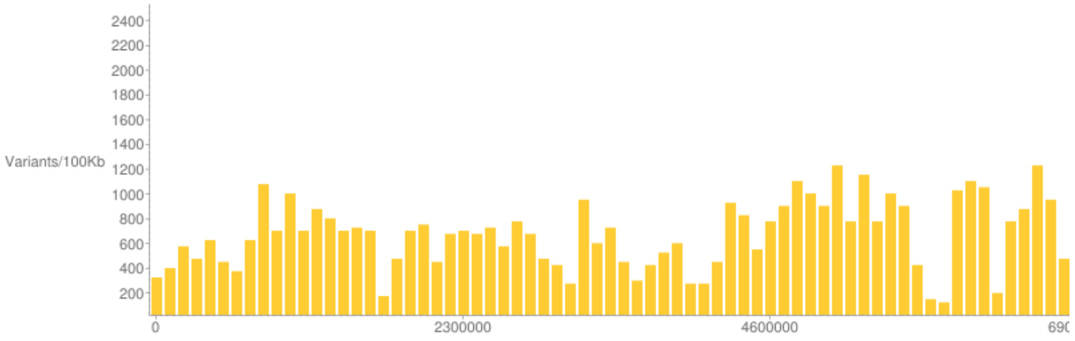


```
3, Position,0,100000,200000,300000,400000,500000,600000,700000,800000,900000,1000000,1100000,1200000,1300000,1400000,
3,Count,328,420,575,485,643,470,380,640,1083,717,1002,704,876,805,720,744,704,191,495,711,758,463,678,709,684,731,593,797,692,485,434,
```

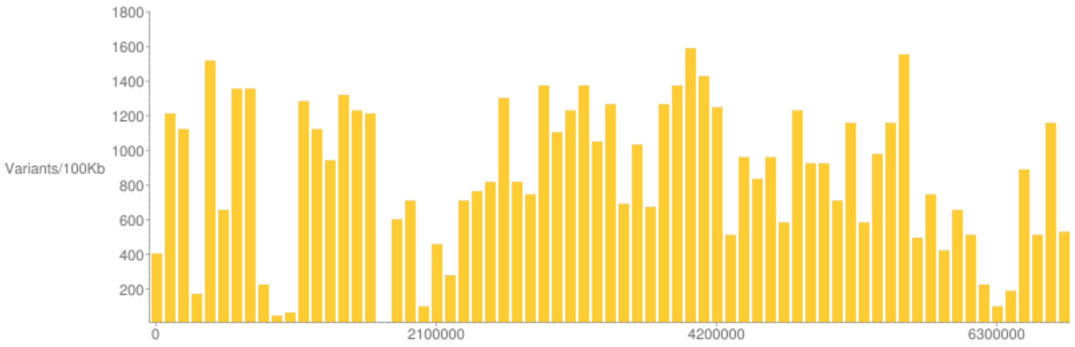

4, Position,0,100000,200000,300000,400000,500000,600000,700000,800000,900000,1000000,1100000,1200000,1300000,1400000,
4,Count,412,1214,1124,171,1529,673,1364,1360,236,59,63,1289,1139,950,1329,1236,1223,9,609,718,102,471,293,712,763,827,1318,829,746,

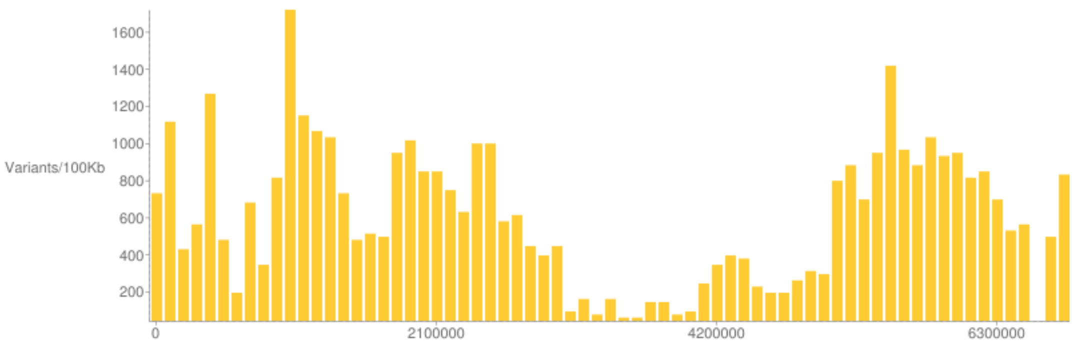

5, Position,0,100000,200000,300000,400000,500000,600000,700000,800000,900000,1000000,1100000,1200000,1300000,1400000,
5,Count,743,1129,440,562,1268,487,206,694,351,814,1718,1155,1068,1044,733,485,517,508,951,1017,862,850,748,642,1012,1010,580,618,445,

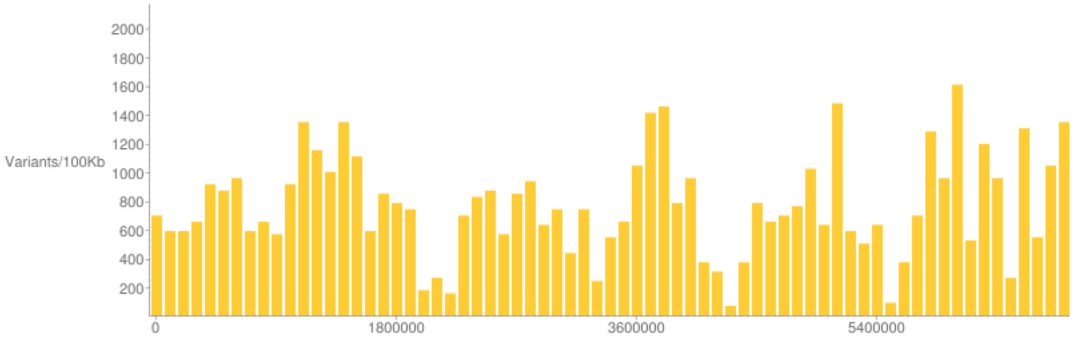

6, Position,0,100000,200000,300000,400000,500000,600000,700000,800000,900000,1000000,1100000,1200000,1300000,1400000,
6,Count,704,610,595,661,925,877,971,608,659,580,935,1353,1162,1021,1351,1119,608,870,793,750,202,280,180,701,840,884,585,867,945,636,

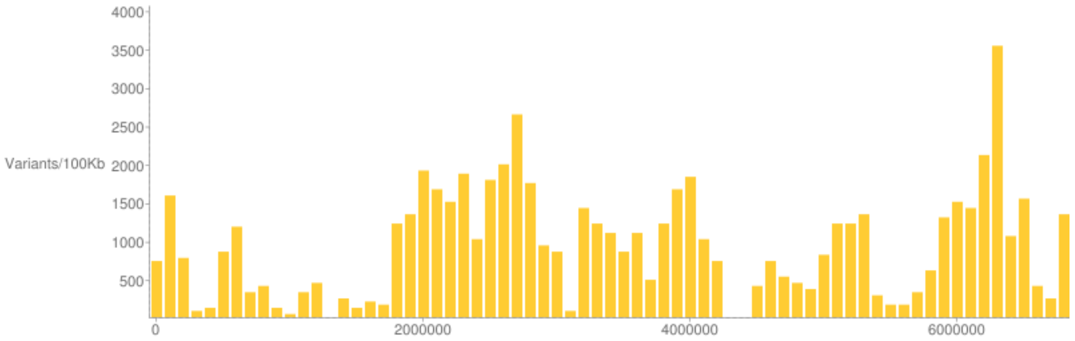

7, Position,0,100000,200000,300000,400000,500000,600000,700000,800000,900000,1000000,1100000,1200000,1300000,1400000,
7,Count,779,1606,797,101,141,874,1223,372,444,139,62,354,499,55,270,153,229,181,1254,1365,1962,1693,1544,1888,1066,1822,2017,2669,

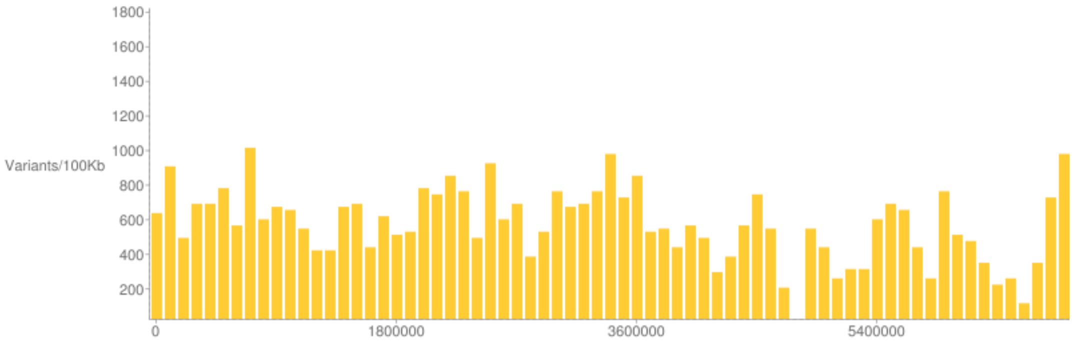

8, Position,0,100000,200000,300000,400000,500000,600000,700000,800000,900000,1000000,1100000,1200000,1300000,1400000,
8,Count,650,922,497,707,707,784,570,1019,613,685,664,562,427,432,680,694,454,635,511,540,787,744,859,763,509,928,607,694,396,541,775,

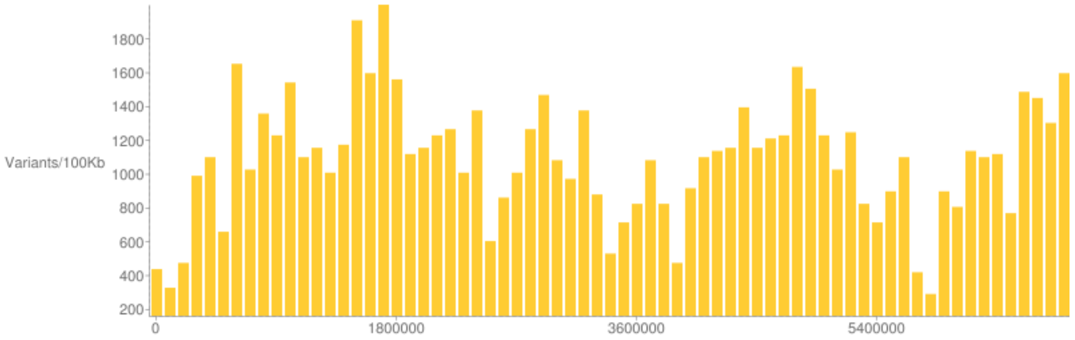

9, Position,0,100000,200000,300000,400000,500000,600000,700000,800000,900000,1000000,1100000,1200000,1300000,1400000,
9,Count,437,335,481,994,1101,673,1650,1032,1367,1239,1539,1105,1159,1006,1177,1912,1605,1999,1560,1132,1167,1229,1268,1023,1378,610,8

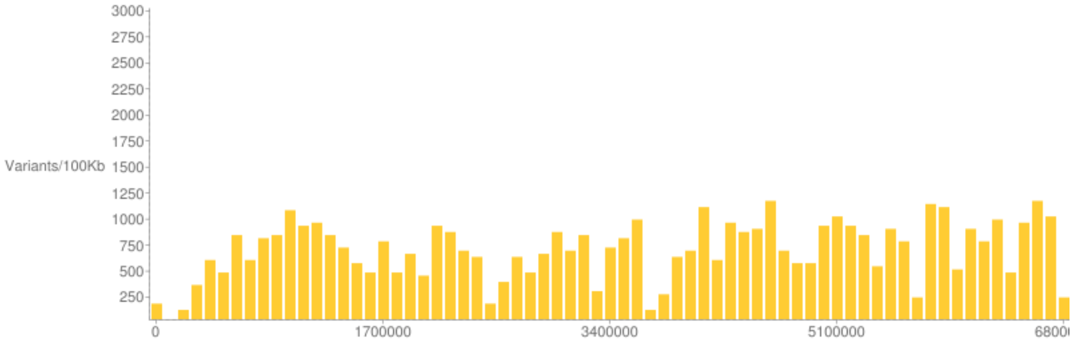

10, Position,0,100000,200000,300000,400000,500000,600000,700000,800000,900000,1000000,1100000,1200000,1300000,1400000,
10,Count,190,32,150,372,602,508,856,606,831,854,1080,945,975,852,746,589,505,790,481,688,465,931,879,715,634,189,405,645,502,685,890,

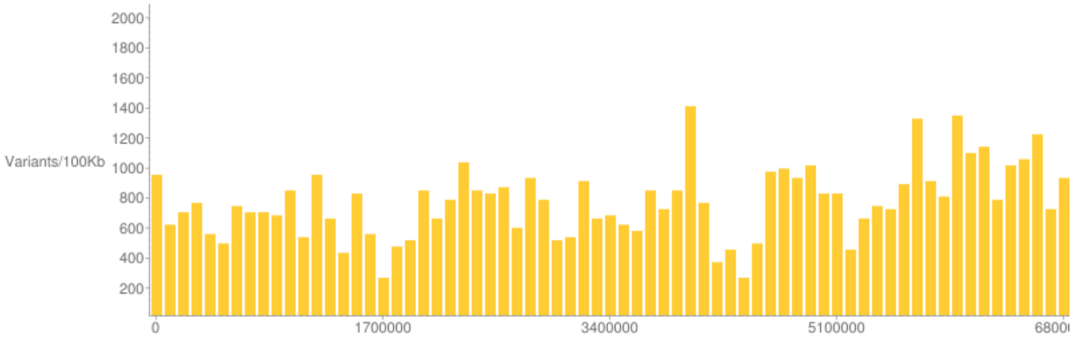

11, Position,0,100000,200000,300000,400000,500000,600000,700000,800000,900000,1000000,1100000,1200000,1300000,1400000,
11,Count,955,626,710,779,575,510,761,713,722,696,864,554,968,679,452,830,569,283,486,529,855,661,797,1038,861,846,877,606,930,793,530,

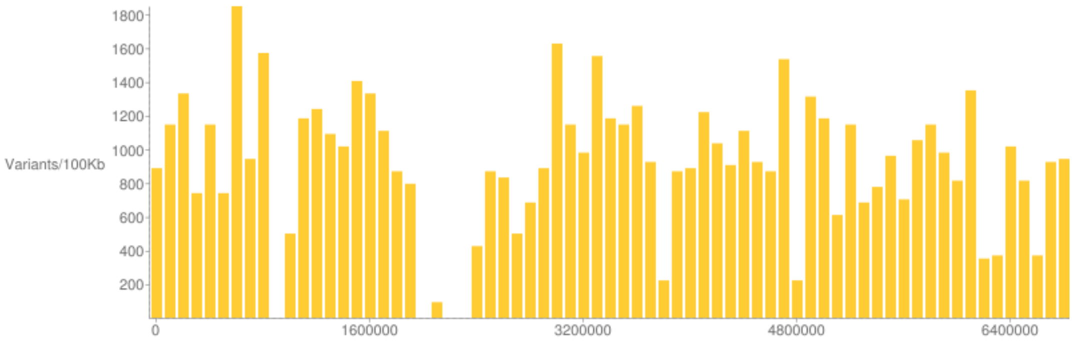

12, Position,0,100000,200000,300000,400000,500000,600000,700000,800000,900000,1000000,1100000,1200000,1300000,1400000,
12,Count,907,1164,1347,741,1148,748,1849,948,1578,5,519,1190,1244,1103,1036,1424,1335,1119,871,797,3,98,2,9,431,871,844,514,697,904,1

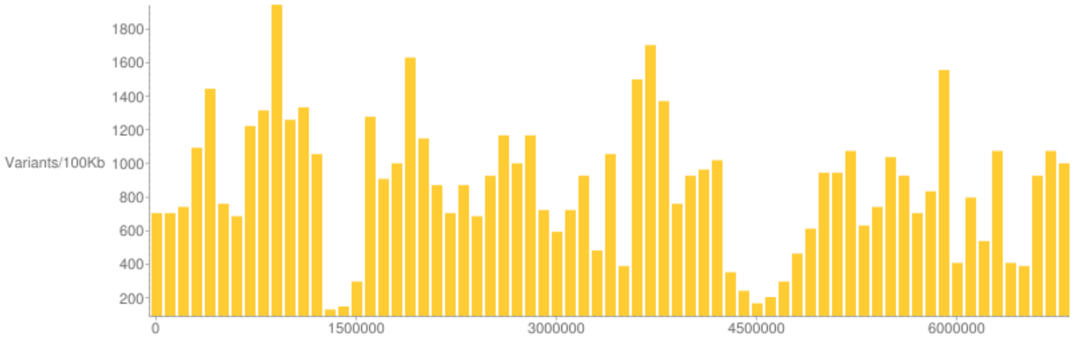

13, Position,0,100000,200000,300000,400000,500000,600000,700000,800000,900000,1000000,1100000,1200000,1300000,1400000,
13,Count,715,701,751,1092,1449,758,683,1237,1324,1941,1263,1332,1056,145,152,297,1286,915,1011,1639,1149,871,706,876,692,935,1182,100

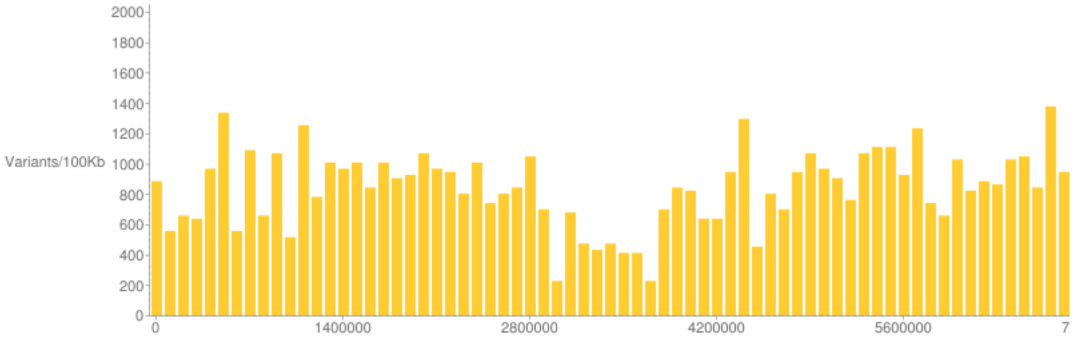

14, Position,0,100000,200000,300000,400000,500000,600000,700000,800000,900000,1000000,1100000,1200000,1300000,1400000,
14,Count,894,568,662,645,984,1338,567,1100,673,1076,525,1253,784,1012,969,1019,850,1018,910,936,1079,974,958,803,1007,756,804,852,104

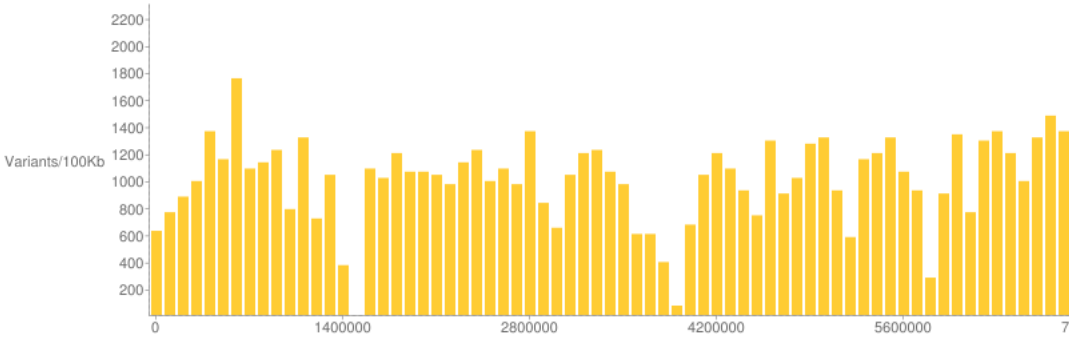

15, Position,0,100000,200000,300000,400000,500000,600000,700000,800000,900000,1000000,1100000,1200000,1300000,1400000,
15,Count,653,785,904,1012,1372,1169,1766,1104,1148,1253,810,1329,736,1059,388,11,1102,1039,1230,1073,1075,1051,994,1146,1241,1015,109

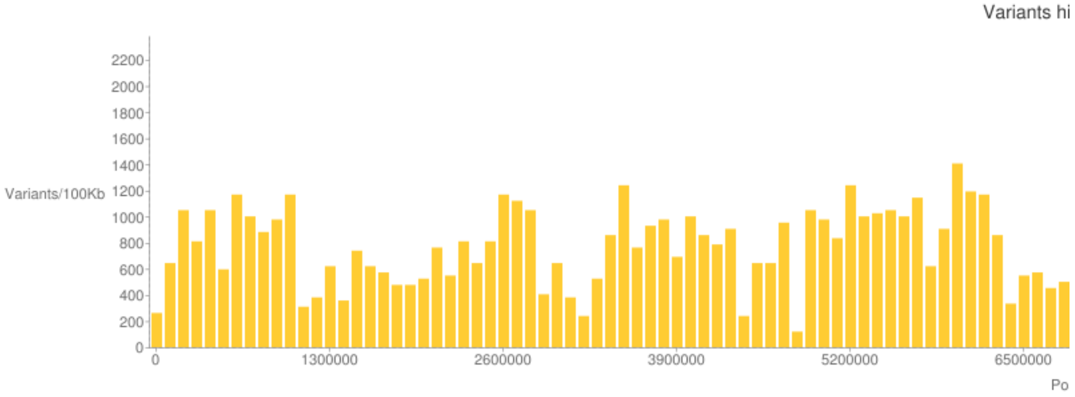

16, Position,0,100000,200000,300000,400000,500000,600000,700000,800000,900000,1000000,1100000,1200000,1300000,1400000,
16,Count,286,665,1057,814,1057,610,1184,1019,888,992,1184,328,390,641,373,754,643,575,498,479,538,780,551,831,653,817,1184,1122,1052,

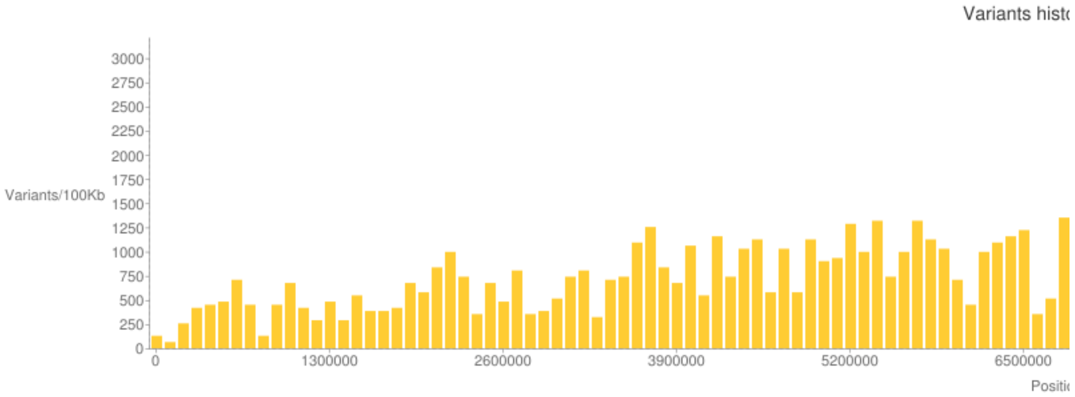

17, Position,0,100000,200000,300000,400000,500000,600000,700000,800000,900000,1000000,1100000,1200000,1300000,1400000,
17,Count,147,79,279,435,470,492,719,480,136,467,694,422,292,500,302,553,407,408,446,680,588,839,1019,762,381,679,507,817,370,410,531,

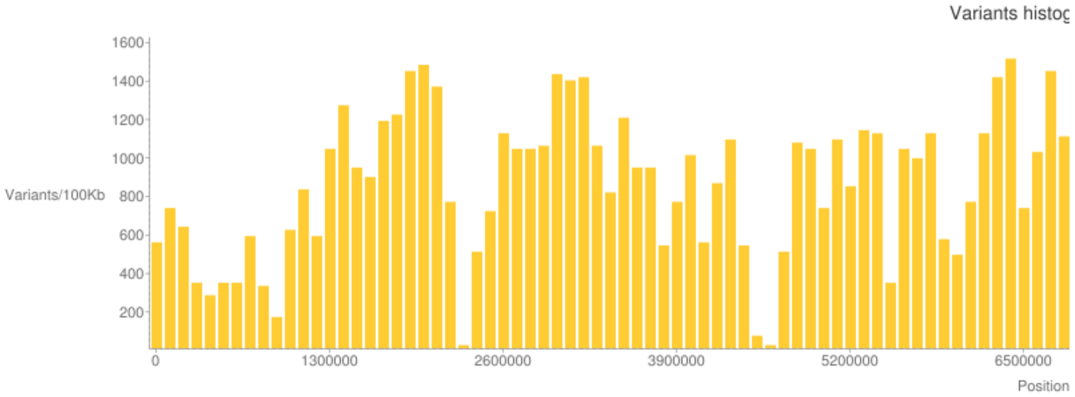

18, Position,0,100000,200000,300000,400000,500000,600000,700000,800000,900000,1000000,1100000,1200000,1300000,1400000,
18,Count,566,742,645,362,298,357,364,606,334,174,638,848,605,1051,1271,963,907,1195,1231,1465,1495,1369,780,39,522,725,1139,1047,1060,

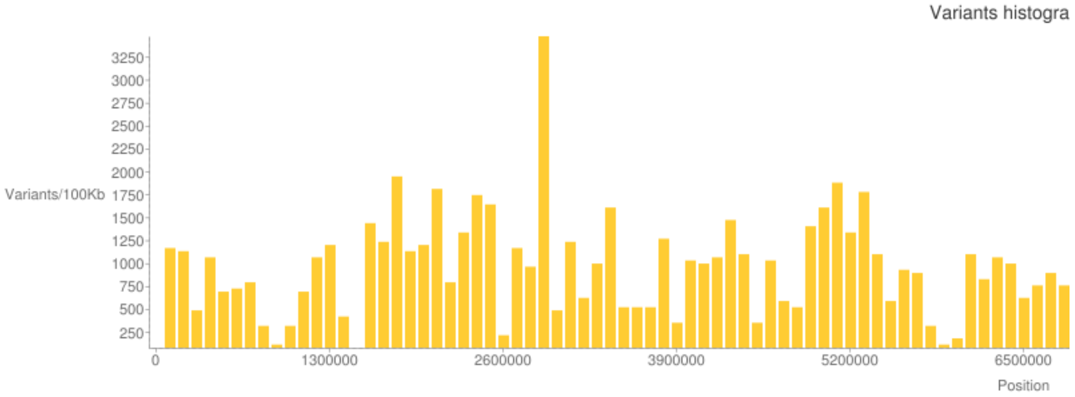

19, Position,0,100000,200000,300000,400000,500000,600000,700000,800000,900000,1000000,1100000,1200000,1300000,1400000,
19,Count,89,1193,1158,516,1091,708,752,792,344,142,348,694,1083,1216,443,77,1453,1247,1968,1132,1227,1830,809,1363,1761,1650,228,1181,

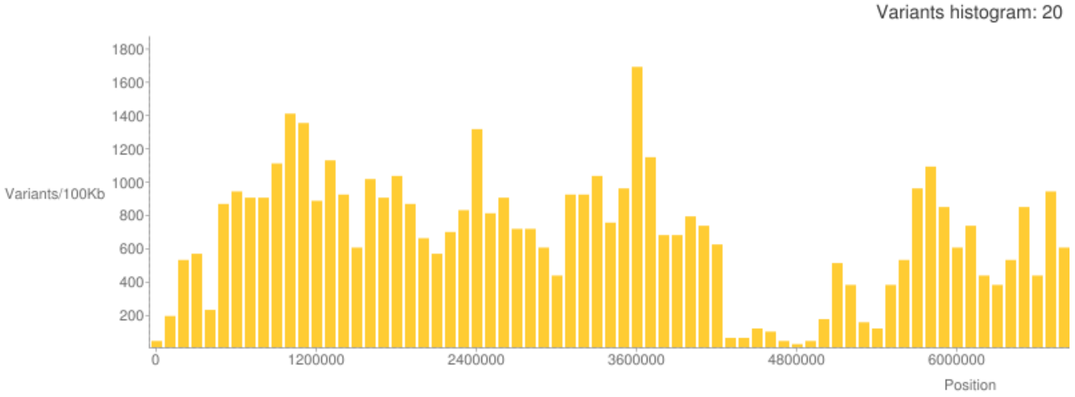

20, Position,0,100000,200000,300000,400000,500000,600000,700000,800000,900000,1000000,1100000,1200000,1300000,1400000,
20,Count,46,204,545,568,234,871,952,907,916,1126,1410,1364,888,1129,926,618,1024,917,1050,878,672,573,706,845,1324,825,915,719,729,62

Details by gene

Here you can find a tab-separated table.

SnpEff: Variant analysis

Contents

Summary
Variant rate by chromosome
Variants by type
Number of variants by impact
Number of variants by functional class
Number of variants by effect
Quality histogram
InDel length histogram
Base variant table
Transition vs transversions (ts/tv)
Allele frequency
Allele Count
Codon change table
Amino acid change table
Chromosome variants plots
Details by gene

Summary

**Genome**	manindi
**Date**	2021-05-22 14:33
**SnpEff version**	SnpEff 5.0e (build 2021-03-09 06:01), by Pablo Cingolani
**Command line arguments**	SnpEff manindi /home/cocogenomics/mango_genome/mango_ref_genome/M.altissima_ref_INDEL_final.vcf
**Warnings**	62,896
**Errors**	0
**Number of lines (input file)**	472,669
**Number of variants (before filter)**	474,404
**Number of not variants (i.e. reference equals alternative)**	0
**Number of variants processed (i.e. after filter and non-variants)**	474,404
**Number of known variants (i.e. non-empty ID)**	0 (0%)
**Number of multi-allelic VCF entries (i.e. more than two alleles)**	1,735
**Number of effects**	1,056,265
**Genome total length**	391,108,416
**Genome effective length**	357,440,369
**Variant rate**	1 variant every 753 bases

Variants rate details

Chromosome	Length	Variants	Variants rate
1	29,456,600	40,387	729
2	24,397,897	32,173	758
3	23,139,393	26,692	866
4	21,507,500	28,148	764
5	21,083,371	25,171	837
6	18,811,960	25,448	739
7	20,623,120	29,461	700
8	18,243,469	22,798	800
9	18,233,274	29,470	618
10	17,652,500	22,671	778
11	17,144,574	20,014	856
12	16,029,966	22,024	727
13	15,457,994	19,755	782
14	14,810,209	18,977	780
15	14,765,960	22,200	665
16	13,817,227	17,022	811
17	13,506,273	20,142	670
18	13,371,754	18,635	717
19	13,093,066	19,411	674
20	12,294,262	13,805	890
**Total**	**357,440,369**	**474,404**	**753**

Number variants by type

Type	Total
**SNP**	0
**MNP**	0
**INS**	236,119
**DEL**	238,285
**MIXED**	0
**INV**	0
**DUP**	0
**BND**	0
**Total**	**474,404**

Type	Total
INTERVAL	0
Total	474,404

Number of effects by impact

Type (alphabetical order)	Count	Percent
HIGH	7,087	0.671%
LOW	1,688	0.16%
MODERATE	3,014	0.285%
MODIFIER	1,044,476	98.884%

Number of effects by functional class

Type (alphabetical order)	Count	Percent

Missense / Silent ratio: 0

Number of effects by type and region

Type

Type (alphabetical order)	Count	Percent
3_prime_UTR_variant	5,528	0.522%
5_prime_UTR_truncation	3	0%
5_prime_UTR_variant	3,489	0.329%
bidirectional_gene_fusion	1	0%
conservative_inframe_deletion	579	0.055%
conservative_inframe_insertion	746	0.07%
disruptive_inframe_deletion	1,086	0.103%
disruptive_inframe_insertion	706	0.067%
downstream_gene_variant	273,125	25.789%
exon_loss_variant	4	0%
frameshift_variant	6,757	0.638%
intergenic_region	380,721	35.949%
intragenic_variant	4	0%
intron_variant	75,690	7.147%
non_coding_transcript_variant	98	0.009%
splice_acceptor_variant	147	0.014%
splice_donor_variant	187	0.018%
splice_region_variant	2,078	0.196%
start_lost	108	0.01%
start_retained_variant	9	0.001%
stop_gained	212	0.02%
stop_lost	80	0.008%
stop_retained_variant	13	0.001%
upstream_gene_variant	307,696	29.053%

Region

Type (alphabetical order)	Count	Percent
DOWNSTREAM	273,117	25.857%
EXON	9,869	0.934%
GENE	1	0%
INTERGENIC	380,721	36.044%
INTRON	73,814	6.988%
SPLICE_SITE_ACCEPTOR	101	0.01%
SPLICE_SITE_DONOR	149	0.014%
SPLICE_SITE_REGION	1,688	0.16%
TRANSCRIPT	102	0.01%
UPSTREAM	307,696	29.131%
UTR_3_PRIME	5,523	0.523%
UTR_5_PRIME	3,484	0.33%

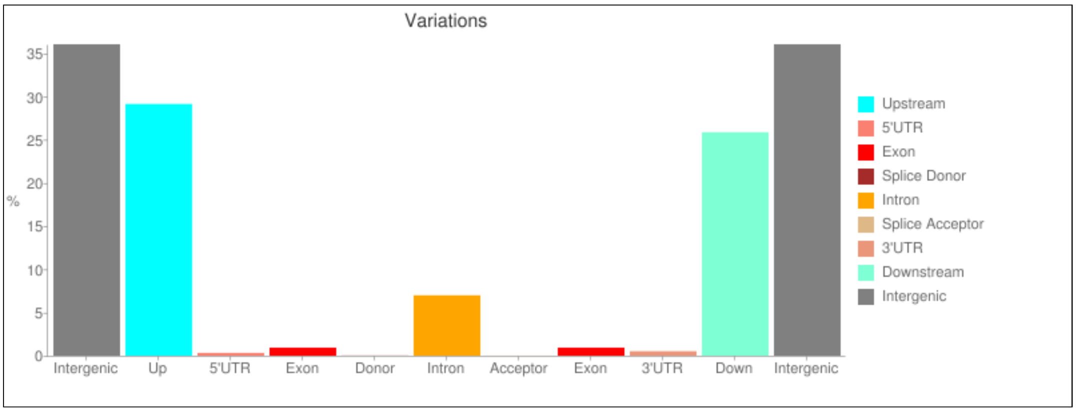

Quality:

**Min**	10
**Max**	61,696
**Mean**	123.704
**Median**	97
**Standard deviation**	350.541
**Values**	10,11,12,13,14,15,16,17,18,19,20,21,22,23,24,25,26,27,28,29,30,31,32,33,34,35,36,37,38,39,40,41,42,43,44,45,46,47,48,49,50,51,52,53,54,55,56,57
**Count**	1554,2112,2580,1946,1524,1563,1881,1603,1683,2236,1543,1659,2482,1583,1704,3110,1509,1587,4025,1562,1731,6136,1961,1729,8610,1409,15[illegible]

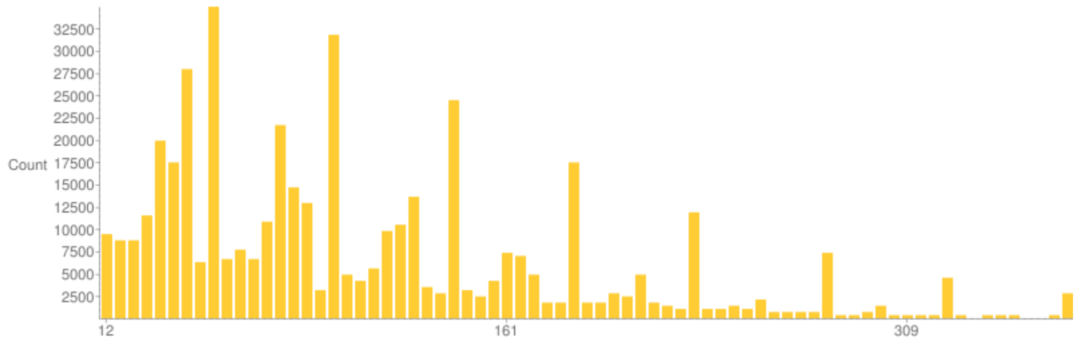

Insertions and deletions length:

**Min**	0
**Max**	222
**Mean**	2.122
**Median**	1
**Standard deviation**	5.661
**Values**	0,1,2,3,4,5,6,7,8,9,10,11,12,13,14,15,16,17,18,19,20,21,22,23,24,25,26,27,28,29,30,31,32,33,34,35,36,37,38,39,40,41,42,43,44,45,46,47,48,49,50,51
**Count**	118045,278476,16984,11909,6517,5439,3602,3598,3106,2989,2486,2616,1794,1586,1324,1295,1014,997,866,833,725,644,547,519,415,384,402,34

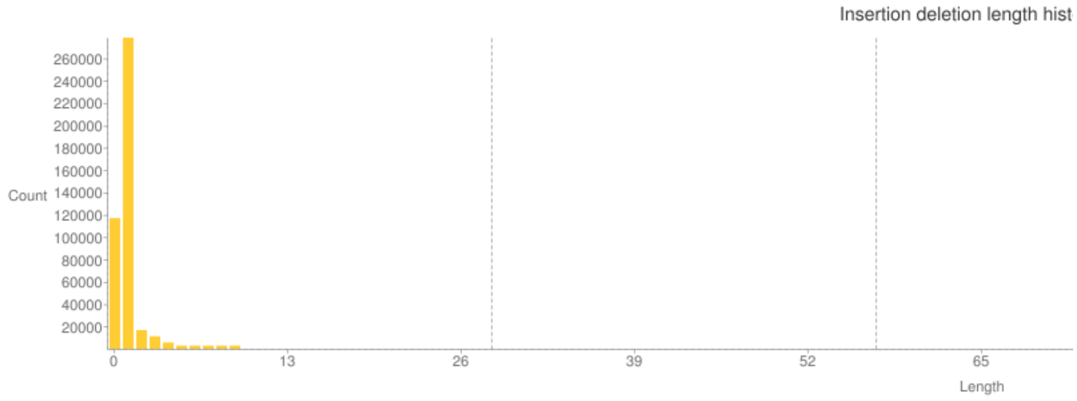

Base changes (SNPs)

	A	C	G	T
**A**	0	0	0	0
**C**	0	0	0	0
**G**	0	0	0	0
**T**	0	0	0	0

Ts/Tv (transitions / transversions)

Note: Only SNPs are used for this statistic.
Note: This Ts/Tv ratio is a 'raw' ratio (ratio of observed events).

**Transitions**	0
**Transversions**	0
**Ts/Tv ratio**	0

All variants:

`No results available (empty input?)`

Only known variants (i.e. the ones having a non-empty ID field):

`No results available (empty input?)`

Allele frequency

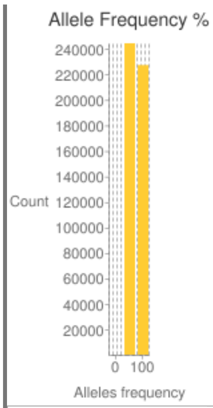

**Min**	0
**Max**	100
**Mean**	74.094
**Median**	50
**Standard deviation**	25.04
**Values**	0,50,100
**Count**	266,244371,228032

Allele Count

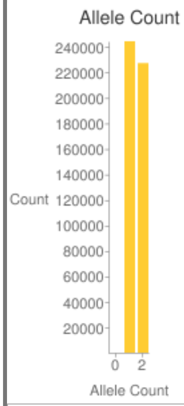

**Min**	0
**Max**	2
**Mean**	1.482
**Median**	1
**Standard deviation**	0.501
**Values**	0,1,2
**Count**	266,244371,228032

Hom/Het per sample

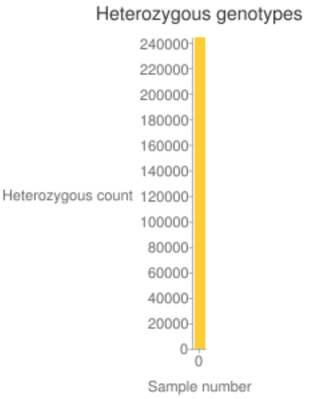

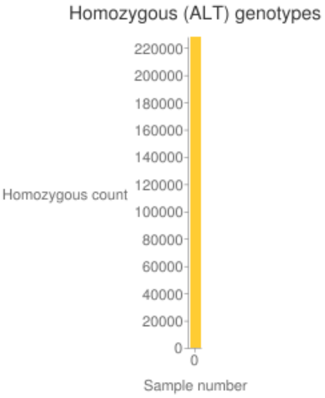

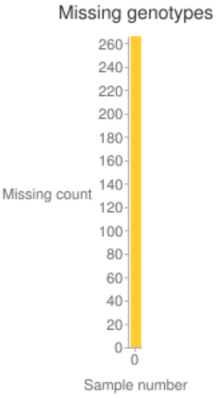


```
Sample_names , readname
Reference , 0
Het , 244371
Hom , 228032
Missing , 266
```

Codon changes

How to read this table:
- Rows are reference codons and columns are changed codons. E.g. Row 'AAA' column 'TAA' indicates how many 'AAA' codons have been replaced by 'TAA' codons.
- Red background colors indicate that more changes happened (heat-map).
- Diagonals are indicated using grey background color
- WARNING: This table may include different translation codon tables (e.g. mamalian DNA and mitochondrial DNA).

	-	AAA	AAC	AAG	AAT	ACA	ACC	ACG	ACT	AGA	AGC	AGG	AGT	ATA	ATC	ATG	ATT	CAA	CAC	CAG	CAT	CCA	CC[illegible]
**-**		173	94	153	184	104	65	36	102	122	68	82	98	96	70	108	122	149	63	109	120	139	5
**AAA**	373	**47**	14	33	10	6		2	3	14	1	2	5	8	2	6	3	5		2			
**AAC**	197	20	**14**	17	20	6			2	6	4	1	1	2	1	6	1	1	1				
**AAG**	389	58	10	**26**	21	3	1	2	3	11		3	1	2		6	4	5		2			
**AAT**	333	35	15	13	**25**	6	1		5	7	6	2	4	5	1	5	3	2			3		
**ACA**	181	1	10	4	1	**14**	12	3	14	4	2	1	5	4	2		6	2					
**ACC**	119		9	1		10	**6**	5	11	1	1			2	3	2	2		4				
**ACG**	74	2	2	1		8	3	**10**	9		1		2		1		3	1					
**ACT**	195	3	14	5	3	16	15	3	**13**	1	3	2		3	5	2	3					1	
**AGA**	198	4		18	2	3			1	**13**	5	23	12	4	3	3	2						
**AGC**	115	3	3	11	5				2	8	**3**	7	6		1	2	3			3			
**AGG**	149	4	2	13	3		2	2	1	20	4	**20**	12	1	2	1							
**AGT**	190	2		11	5	3	1	2	4	12	9	6	**9**	1		3	4			2	1	1	
**ATA**	155	2		1	11	4	2		2	3			3	**9**	5	8	11	2			1		
**ATC**	114	4	1	2	8	4	4		2	1	1		1	4	**7**	7	11				4		
**ATG**	293	6	1	6	33	5	1	2	3	6			3	16	3	**19**	17						
**ATT**	213	2	3	2	9	1	4		6	1	1	1	8	11	16	13	**10**		1	1	1		

	-	AAA	AAC	AAG	AAT	ACA	ACC	ACG	ACT	AGA	AGC	AGG	AGT	ATA	ATC	ATG	ATT	CAA	CAC	CAG	CAT	CCA	CC
**CAA**	246	1	1	1	1	7											1	**25**	4	17	15	10	
**CAC**	90			2		1												6	**2**	8	13	3	
**CAG**	174				1	1	1										1	28	2	**13**	13	5	
**CAT**	183			1		2												18	16	7	**10**	10	
**CCA**	232						3			1								3	1	2	8	**14**	1
**CCC**	90		1				1				1							1	1			8	**16**
**CCG**	73		1																	6		16	1
**CCT**	229						1		3	1			1					1	2	4	1	20	2
**CGA**	58							1										4		3	1	1	
**CGC**	39																		2	1			
**CGG**	51																1	3		4			
**CGT**	77			1													1			1	3	1	
**CTA**	112					1							1					1	1		4	4	
**CTC**	118								1									2	1		3	1	
**CTG**	132								3									2	1	1	8	1	
**CTT**	203		1						1									7	3		7	2	
**GAA**	503	1		7						5			1	1									
**GAC**	171		2	1						7					1	1		1					
**GAG**	369	1	1	8			1			5				1									
**GAT**	432			1	2					4			1			1				1	1		
**GCA**	222			1		1		1			2					1						2	
**GCC**	100		1				2				2											1	
**GCG**	67			1			1																
**GCT**	251			1					2		5					2							
**GGA**	275	1								1		3		2		1							
**GGC**	143		1	2	1		1					2	1							1			
**GGG**	150			1								4									1		
**GGT**	238			1					1			3	2			1					1		
**GTA**	142												1										
**GTC**	104		1	2									1										
**GTG**	178												1			3						1	
**GTT**	240			1			1	1	1	1			4		1	2						1	
**TAA**	16				1																		
**TAC**	102			1	1			1		1			1								1		
**TAG**	12																						
**TAT**	180			1	1						1			3			1	1			1		
**TCA**	244					1									3		1						
**TCC**	139						1								2		1			1		2	
**TCG**	84				1										2						1		
**TCT**	340			1					4						2		1	1			2		
**TGA**	25																						
**TGC**	69	1	1												1	4		1			1		
**TGG**	122	1														4							
**TGT**	119	1		1	2					1	1												
**TTA**	145	1	1	1									1	1			3						
**TTC**	164				1									1					1		1		
**TTG**	230								1	1					1		4						
**TTT**	290				1												10						

Amino acid changes

How to read this table:
- Rows are reference amino acids and columns are changed amino acids. E.g. Row 'A' column 'E' indicates how many 'A' amino acids have been replaced by 'E' amino acids.
- Red background colors indicate that more changes happened (heat-map).
- Diagonals are indicated using grey background color
- WARNING: This table may include different translation codon tables (e.g. mamalian DNA and mitochondrial DNA).

	*	-	?	A	C	D	E	F	G	H	I	K	L	M	N	P	Q	R	S	T	V	W	Y
*****	**14**	52	1		3	1							13		1				6			1	8
**-**	147		3,170	301	152	298	377	252	443	183	288	326	501	108	278	357	258	317	648	307	327	76	170
**?**																							
**A**		640		**134**	4	19	23	1	30			3	4	3	1	4		4	12	7	36		
**C**	4	188		1	**28**	1		12		1	1	3	19	4	3		1	2	17		3	18	6
**D**	9	603		24	1	**74**	73		46	1	1	2	2	2	4		2	13	3		22		1
**E**	6	872		25		80	**149**	1	82		2	17	4		1	1		15	5	1	21	4	2
**F**	10	454		2	10			**104**	1	2	11		31		2	1		1	37		9	4	18
**G**	1	806		24		32	41		**236**	2	2	5	2	2	2	2	1	18	4	2	35	4	
**H**		273		2	1	4		2	2	**41**		3	13			23	39	10	3	3	1		4
**I**	2	482		2	1	5		2	1	7	**84**	13	5	28	32		3	6	16	29	7	2	7
**K**	7	762				2	17	2	4		19	**164**	5	12	55		14	30	8	20	3		
**L**	5	940		5	6	2	1	90	2	28	9	2	**189**		2	52	13	21	40	7	5	3	7
**M**	2	293		1		1	1				36	12	1	**19**	34			6	4	11	2		4
**N**	6	529	1	3		1	4	1	2	4	13	85	2	11	**74**	1	3	17	16	20	1		

	*	-	?	A	C	D	E	F	G	H	I	K	L	M	N	P	Q	R	S	T	V	W	Y
**P**		624		10	1		2	2	1	13			36		2	**208**	17	24	14	8	1		
**Q**		420		12			1	1	2	34	2	2	20		3	27	**83**	16	9	9	1		1
**R**	6	572		6			4	4	6	6	14	40	19	4	7	15	16	**117**	39	10	1	1	1
**S**	15	1,112		4	13		2	69	7	4	21	28	16	5	14	8	7	33	**281**	18	8	4	15
**T**	2	569		4		2	3	2	1	4	34	17	5	4	39	2	3	10	17	**152**	2	2	3
**V**		664		24	2	33	36	3	42		1	3	4	5	1	2		3	9	3	**130**	2	
**W**	4	122			9			1	1			1	13	4				1	3		1	**10**	2
**Y**	28	281	1		8		3	5	3	2	4	2	15		2		1	3	22	1	3	2	**38**

Variants by chromosome

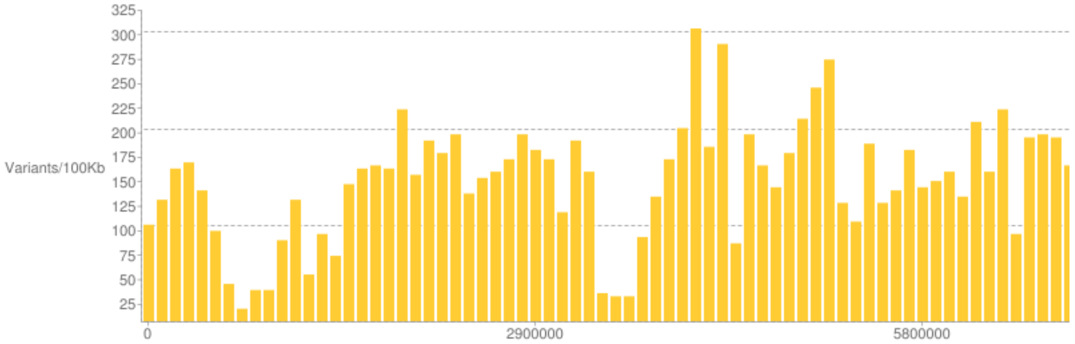


```
1, Position,0,100000,200000,300000,400000,500000,600000,700000,800000,900000,1000000,1100000,1200000,1300000,1400000,
1,Count,108,132,164,171,143,100,47,21,40,39,92,132,57,99,74,149,164,166,164,226,158,194,180,199,139,155,160,175,198,182,174,119,193,1
```

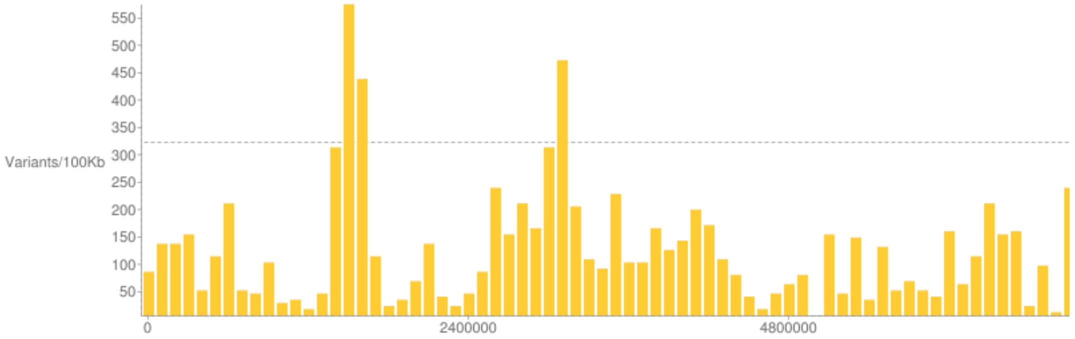


```
2, Position,0,100000,200000,300000,400000,500000,600000,700000,800000,900000,1000000,1100000,1200000,1300000,1400000,
2,Count,90,140,138,158,54,115,215,55,48,108,32,36,21,48,314,574,441,115,27,40,72,138,41,25,51,90,242,159,211,168,315,477,209,113,94,2
```

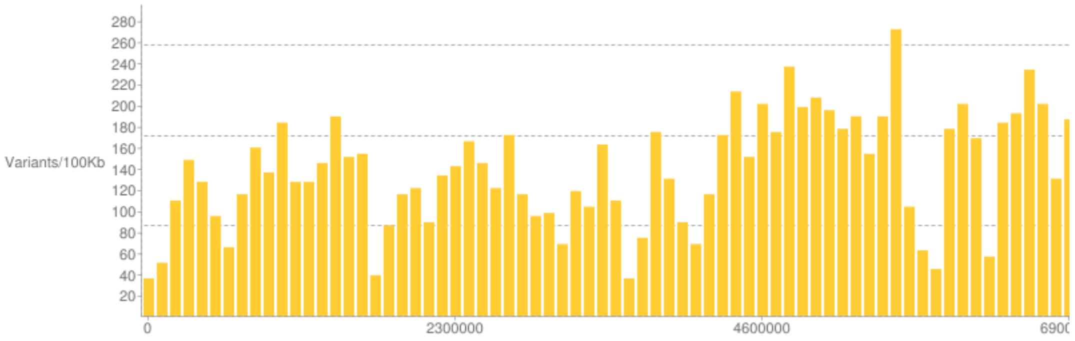


```
3, Position,0,100000,200000,300000,400000,500000,600000,700000,800000,900000,1000000,1100000,1200000,1300000,1400000,
3,Count,37,53,113,150,129,98,68,117,163,139,185,130,129,148,190,152,156,40,89,118,124,90,136,144,169,147,123,174,117,98,99,71,121,105,
```

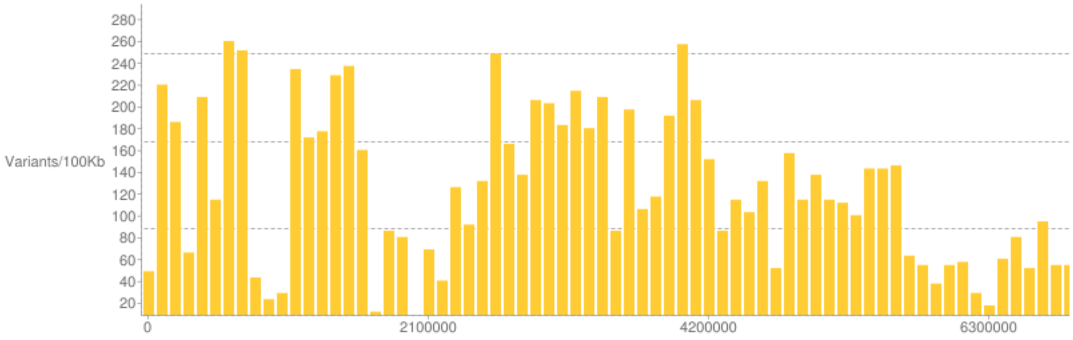

4, Position,0,100000,200000,300000,400000,500000,600000,700000,800000,900000,1000000,1100000,1200000,1300000,1400000,[illegible]
4,Count,49,220,187,67,211,117,262,252,44,26,31,235,172,179,230,237,162,12,86,82,9,71,41,126,93,133,250,166,140,208,203,184,215,181,20[illegible]

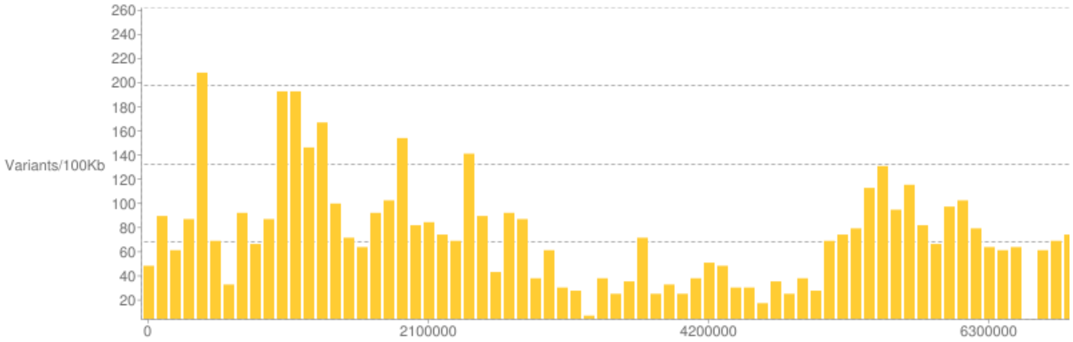

5, Position,0,100000,200000,300000,400000,500000,600000,700000,800000,900000,1000000,1100000,1200000,1300000,1400000,[illegible]
5,Count,49,90,61,88,208,69,34,92,66,89,193,194,147,167,100,73,64,94,104,154,82,85,75,69,143,90,43,94,87,40,62,31,28,7,38,25,36,72,27,[illegible]

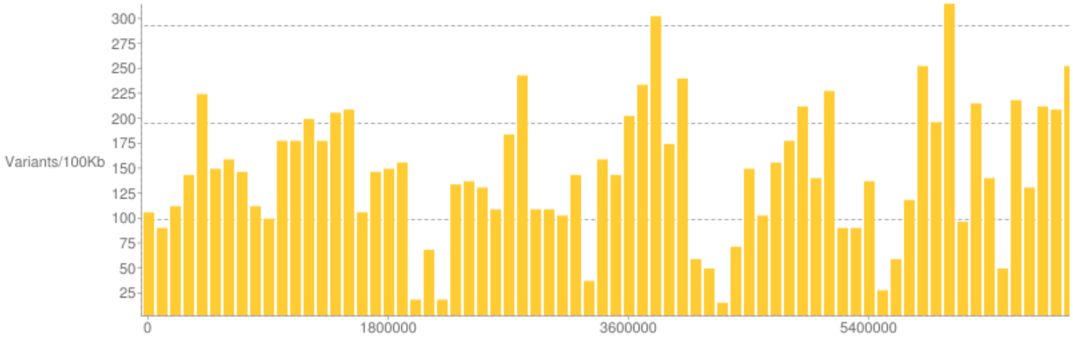

6, Position,0,100000,200000,300000,400000,500000,600000,700000,800000,900000,1000000,1100000,1200000,1300000,1400000,[illegible]
6,Count,106,92,113,145,225,151,158,148,113,100,178,178,200,179,206,211,108,147,149,156,20,69,19,136,137,133,109,183,245,111,110,103,1[illegible]

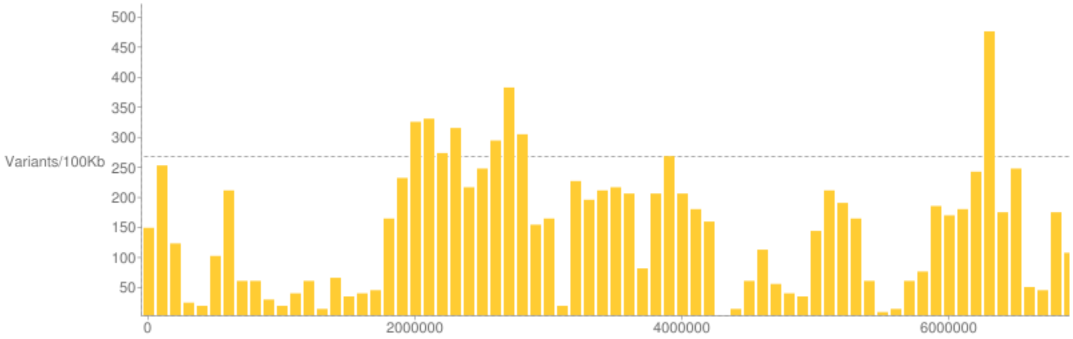

7, Position,0,100000,200000,300000,400000,500000,600000,700000,800000,900000,1000000,1100000,1200000,1300000,1400000,[illegible]
7,Count,153,253,124,25,22,102,213,63,62,31,19,43,64,16,67,39,41,47,167,233,327,332,275,315,220,247,296,385,305,156,167,22,228,198,213,[illegible]

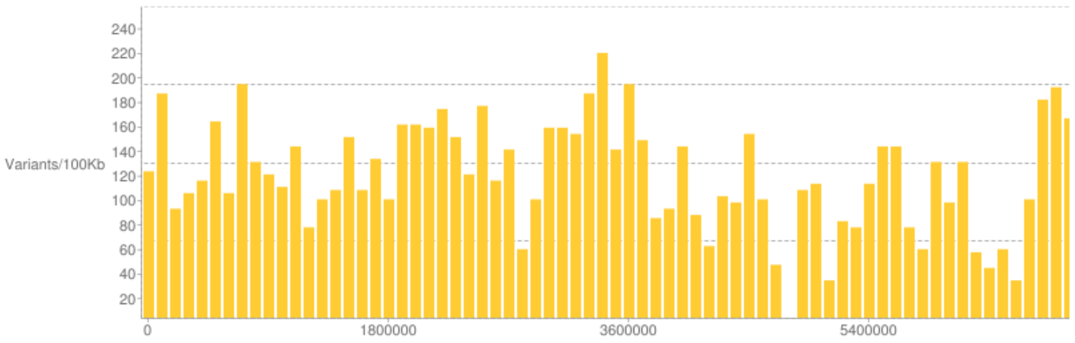

8, Position,0,100000,200000,300000,400000,500000,600000,700000,800000,900000,1000000,1100000,1200000,1300000,1400000,
8,Count,125,188,94,108,116,165,108,197,133,122,112,144,79,102,109,153,109,135,103,162,163,160,176,153,121,178,117,142,61,102,160,160,

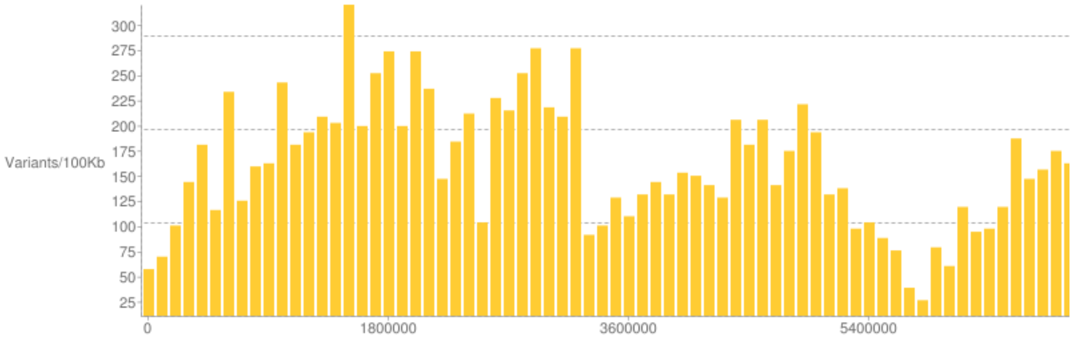

9, Position,0,100000,200000,300000,400000,500000,600000,700000,800000,900000,1000000,1100000,1200000,1300000,1400000,
9,Count,58,71,101,146,183,119,235,128,161,163,244,184,194,210,204,320,200,254,274,200,275,239,148,185,214,104,229,218,254,278,220,211,

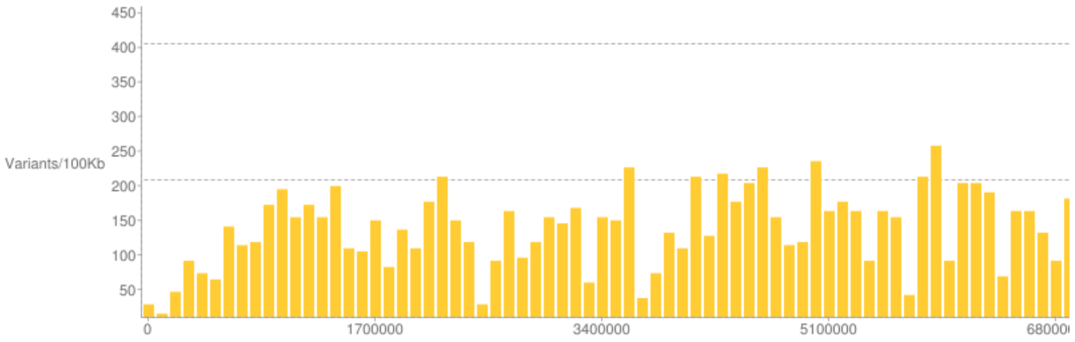

10, Position,0,100000,200000,300000,400000,500000,600000,700000,800000,900000,1000000,1100000,1200000,1300000,1400000,
10,Count,31,16,50,95,76,68,142,117,119,174,198,158,176,156,200,111,105,152,84,137,110,178,215,151,122,30,95,166,99,118,158,149,171,62,

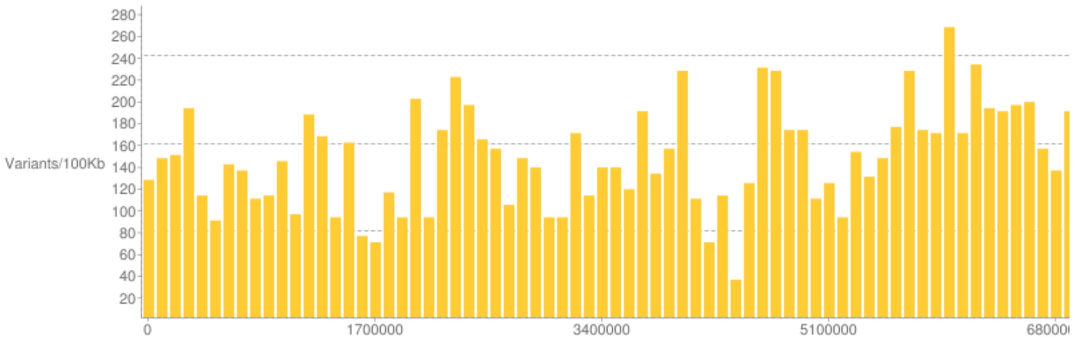

11, Position,0,100000,200000,300000,400000,500000,600000,700000,800000,900000,1000000,1100000,1200000,1300000,1400000,
11,Count,129,148,152,194,115,92,144,137,113,115,145,98,188,169,94,163,79,73,117,95,203,96,174,224,197,167,159,106,150,141,94,95,173,

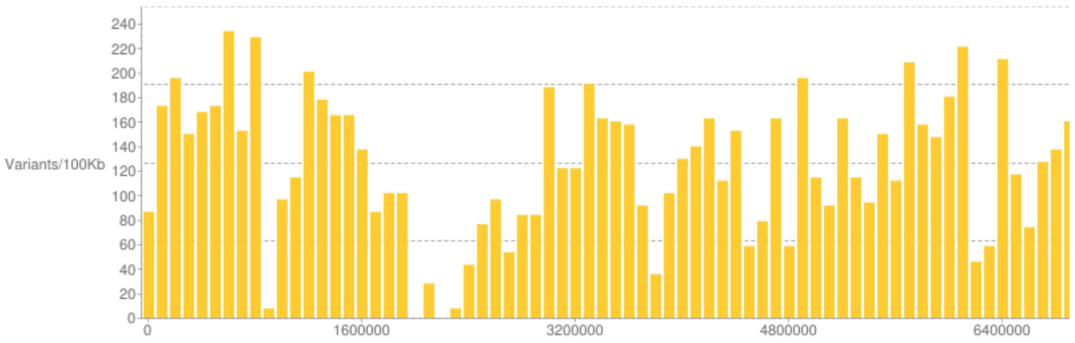

12, Position,0,100000,200000,300000,400000,500000,600000,700000,800000,900000,1000000,1100000,1200000,1300000,1400000
12,Count,87,174,196,151,169,175,235,153,231,8,99,115,201,180,166,167,139,87,102,103,0,30,1,9,45,78,99,55,85,85,190,123,123,193,165,16

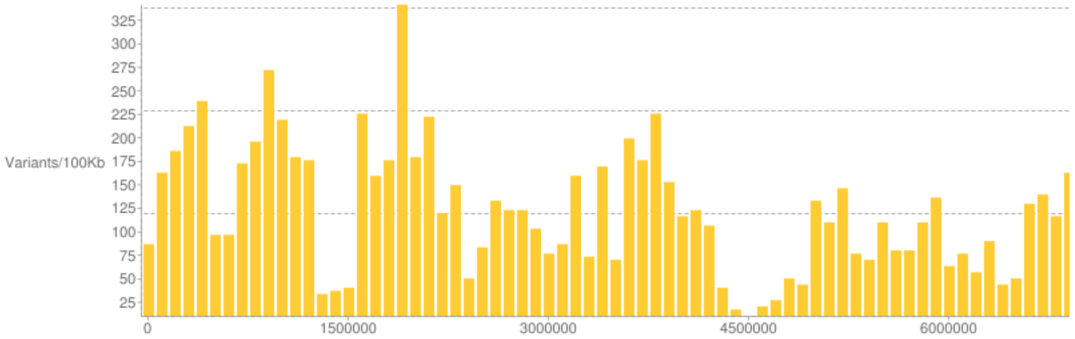

13, Position,0,100000,200000,300000,400000,500000,600000,700000,800000,900000,1000000,1100000,1200000,1300000,1400000
13,Count,87,165,186,214,239,99,98,175,196,273,220,179,178,36,38,40,227,162,176,341,180,225,120,152,51,83,134,123,123,103,79,87,162,76

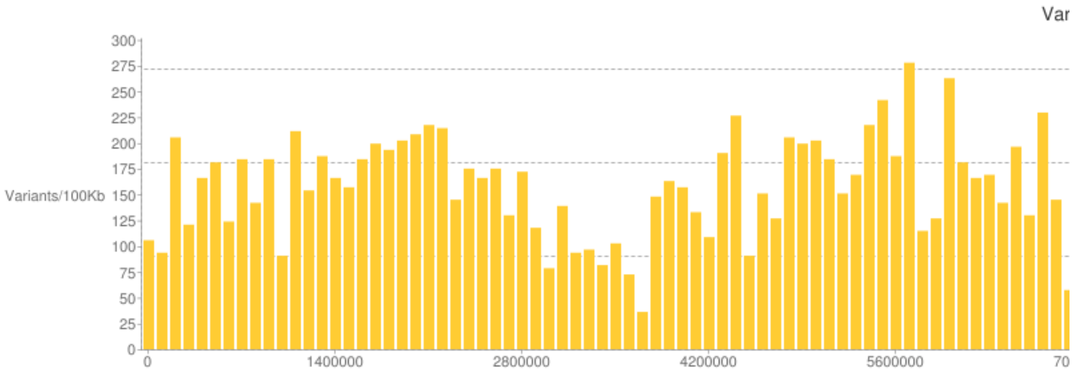

14, Position,0,100000,200000,300000,400000,500000,600000,700000,800000,900000,1000000,1100000,1200000,1300000,1400000
14,Count,108,95,208,123,168,182,125,185,144,185,91,212,157,188,169,160,185,201,195,203,210,218,215,146,177,167,177,130,175,119,80,139

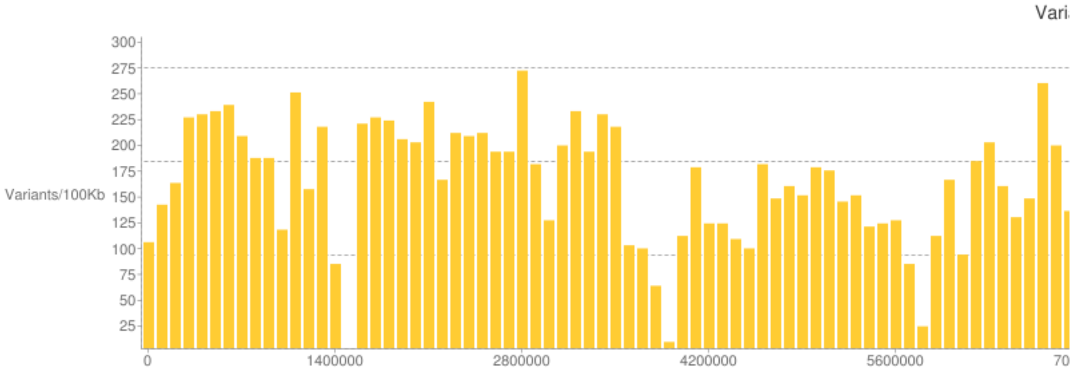

15, Position,0,100000,200000,300000,400000,500000,600000,700000,800000,900000,1000000,1100000,1200000,1300000,1400000
15,Count,106,144,165,227,230,234,241,211,190,188,118,252,159,220,85,3,223,227,225,207,204,243,168,212,209,213,196,194,273,183,129,201

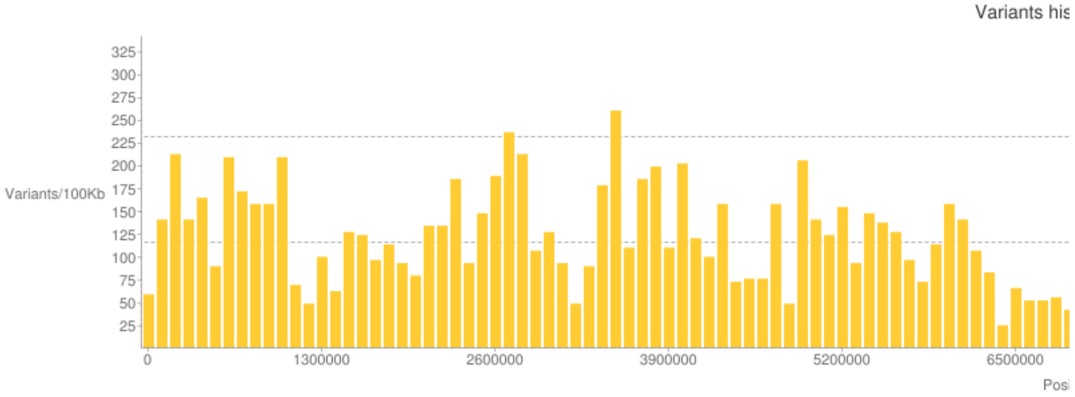

16, Position,0,100000,200000,300000,400000,500000,600000,700000,800000,900000,1000000,1100000,1200000,1300000,1400000,
16,Count,61,144,215,144,166,90,211,174,161,159,211,70,49,103,63,128,124,99,115,96,81,134,135,188,96,151,190,237,214,108,128,94,52,92,

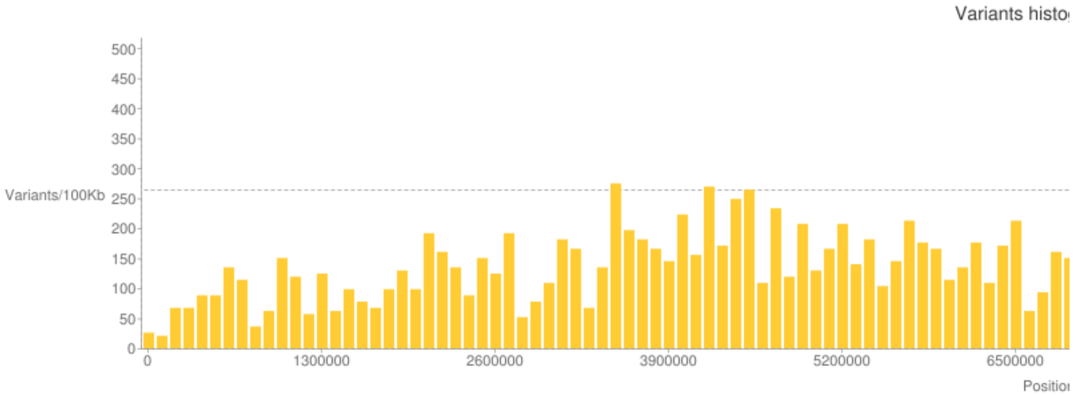

17, Position,0,100000,200000,300000,400000,500000,600000,700000,800000,900000,1000000,1100000,1200000,1300000,1400000,
17,Count,29,25,72,71,93,93,137,116,41,67,154,122,57,129,64,102,81,72,102,133,102,196,162,135,90,151,126,192,55,78,113,184,166,71,137,

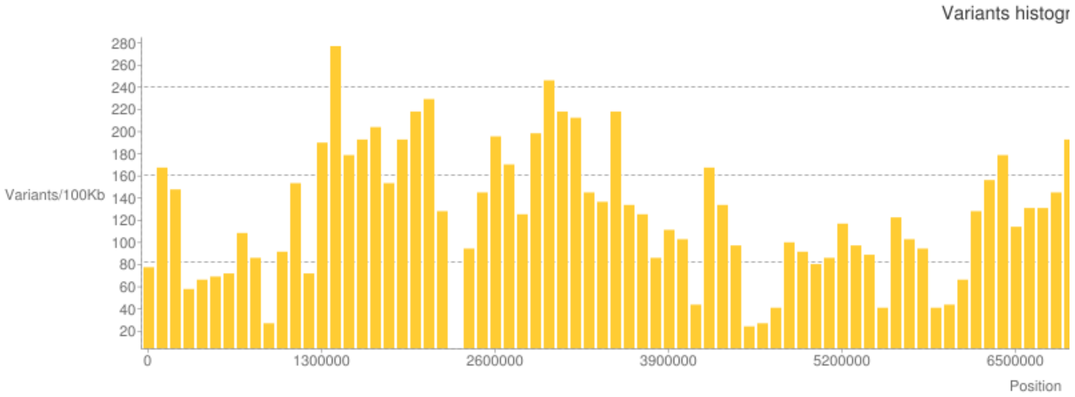

18, Position,0,100000,200000,300000,400000,500000,600000,700000,800000,900000,1000000,1100000,1200000,1300000,1400000,
18,Count,78,168,148,60,67,69,74,108,86,28,92,154,74,190,277,179,195,206,155,195,220,229,128,4,95,146,196,171,127,198,247,218,213,146,

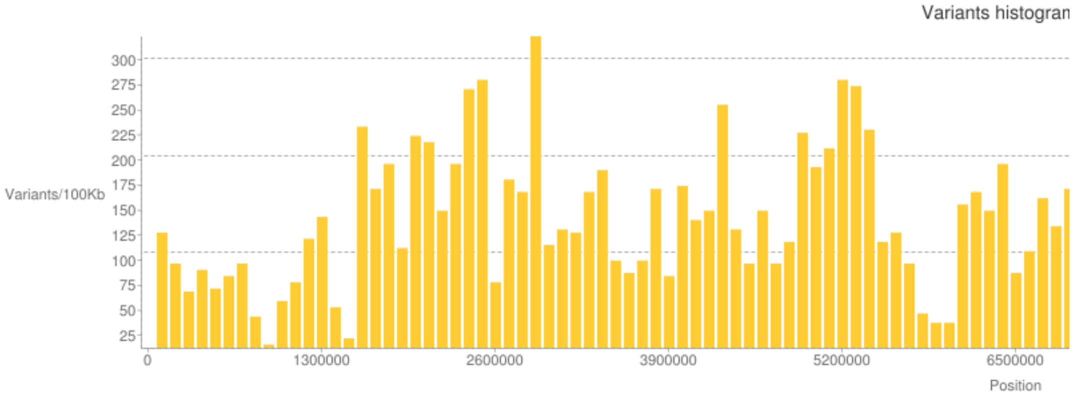

19, Position,0,100000,200000,300000,400000,500000,600000,700000,800000,900000,1000000,1100000,1200000,1300000,1400000,
19,Count,12,129,97,68,91,73,85,99,46,18,60,78,123,144,53,23,234,171,198,112,225,218,151,197,272,282,78,181,170,323,117,131,129,169,19

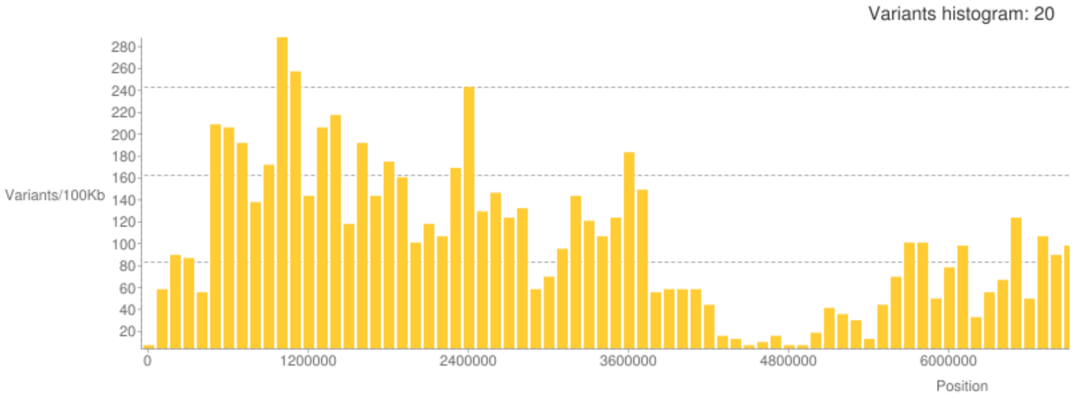


```
20, Position,0,100000,200000,300000,400000,500000,600000,700000,800000,900000,1000000,1100000,1200000,1300000,1400000,
20,Count,7,59,91,88,57,210,207,194,138,174,288,259,144,206,217,118,193,145,176,161,101,119,108,169,245,130,147,126,134,60,70,95,145,1[illegible]
```

Details by gene

Here you can find a tab-separated table.

SnpEff: Variant analysis

Contents

Summary
Variant rate by chromosome
Variants by type
Number of variants by impact
Number of variants by functional class
Number of variants by effect
Quality histogram
InDel length histogram
Base variant table
Transition vs transversions (ts/tv)
Allele frequency
Allele Count
Codon change table
Amino acid change table
Chromosome variants plots
Details by gene

Summary

**Genome**	manindi
**Date**	2021-05-22 14:28
**SnpEff version**	`SnpEff 5.0e (build 2021-03-09 06:01), by Pablo Cingolani`
**Command line arguments**	`SnpEff manindi /home/cocogenomics/mango_genome/mango_ref_genome/M.indica_ref_SNP_final.vcf`
**Warnings**	237,840
**Errors**	0
**Number of lines (input file)**	2,350,709
**Number of variants (before filter)**	2,355,481
**Number of not variants (i.e. reference equals alternative)**	0
**Number of variants processed (i.e. after filter and non-variants)**	2,355,481
**Number of known variants (i.e. non-empty ID)**	0 (0%)
**Number of multi-allelic VCF entries (i.e. more than two alleles)**	4,772
**Number of effects**	4,704,662
**Genome total length**	391,108,416
**Genome effective length**	357,440,369
**Variant rate**	1 variant every 151 bases

Variants rate details

Chromosome	Length	Variants	Variants rate
1	29,456,600	200,394	146
2	24,397,897	165,087	147
3	23,139,393	134,558	171
4	21,507,500	129,543	166
5	21,083,371	122,880	171
6	18,811,960	155,807	120
7	20,623,120	136,536	151
8	18,243,469	96,963	188
9	18,233,274	132,316	137
10	17,652,500	126,684	139
11	17,144,574	96,388	177
12	16,029,966	100,545	159
13	15,457,994	104,188	148
14	14,810,209	87,184	169
15	14,765,960	118,748	124
16	13,817,227	92,430	149
17	13,506,273	91,460	147
18	13,371,754	102,104	130
19	13,093,066	87,184	150
20	12,294,262	74,482	165
**Total**	**357,440,369**	**2,355,481**	**151**

Number variants by type

Type	Total
**SNP**	2,355,481
**MNP**	0
**INS**	0
**DEL**	0
**MIXED**	0
**INV**	0
**DUP**	0
**BND**	0
**Total**	**2,355,481**

Type	Total
INTERVAL	0
Total	2,355,481

Number of effects by impact

Type (alphabetical order)	Count	Percent
HIGH	2,918	0.062%
LOW	64,387	1.369%
MODERATE	79,309	1.686%
MODIFIER	4,558,048	96.884%

Number of effects by functional class

Type (alphabetical order)	Count	Percent
MISSENSE	79,825	58.5%
NONSENSE	1,651	1.21%
SILENT	54,977	40.29%

Missense / Silent ratio: 1.452

Number of effects by type and region

Type

Type (alphabetical order)	Count	Percent
3_prime_UTR_variant	21,424	0.454%
5_prime_UTR_premature_start_codon_gain_variant	1,821	0.039%
5_prime_UTR_variant	11,386	0.241%
downstream_gene_variant	1,139,595	24.168%
initiator_codon_variant	18	0%
intergenic_region	1,862,224	39.493%
intron_variant	327,832	6.953%
missense_variant	79,309	1.682%
splice_acceptor_variant	411	0.009%
splice_donor_variant	345	0.007%
splice_region_variant	10,269	0.218%
start_lost	207	0.004%
start_retained_variant	8	0%
stop_gained	1,651	0.035%
stop_lost	304	0.006%
stop_retained_variant	140	0.003%
synonymous_variant	54,816	1.163%
upstream_gene_variant	1,203,524	25.524%

Region

Type (alphabetical order)	Count	Percent
DOWNSTREAM	1,139,595	24.223%
EXON	135,379	2.878%
INTERGENIC	1,862,224	39.583%
INTRON	319,895	6.8%
SPLICE_SITE_ACCEPTOR	411	0.009%
SPLICE_SITE_DONOR	345	0.007%
SPLICE_SITE_REGION	8,658	0.184%
UPSTREAM	1,203,524	25.582%
UTR_3_PRIME	21,424	0.455%
UTR_5_PRIME	13,207	0.281%

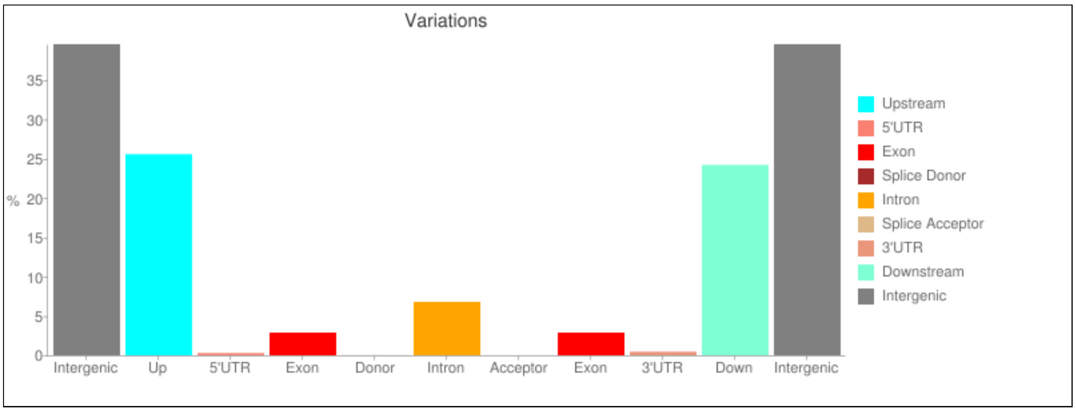

Quality:

Min	10
Max	80,558
Mean	136.731
Median	105
Standard deviation	262.871
Values	10,11,12,13,14,15,16,17,18,19,20,21,22,23,24,25,26,27,28,29,30,31,32,33,34,35,36,37,38,39,40,41,42,43,44,45,46,47,48,49,50,51,52,53,54,55,56,57
Count	2309,1953,10755,3749,3408,2769,4643,6235,10150,10400,12956,12848,16288,15241,17943,22738,17984,16873,19099,10587,9014,14746,8717,9[illegible]

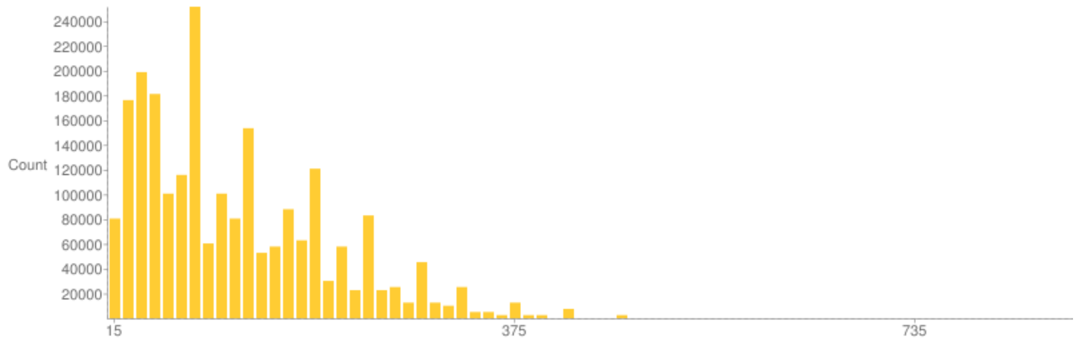

Insertions and deletions length:

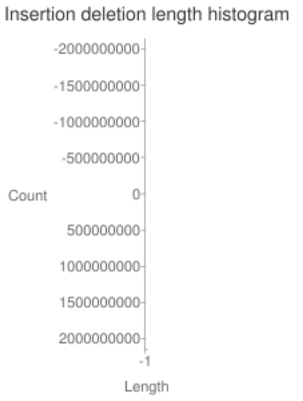

Base changes (SNPs)

	A	C	G	T
A	0	80,838	398,567	119,242
C	87,251	0	54,833	435,503
G	436,295	54,579	0	87,219
T	120,493	399,115	81,546	0

Ts/Tv (transitions / transversions)

Note: Only SNPs are used for this statistic.
Note: This Ts/Tv ratio is a 'raw' ratio (ratio of observed events).

Transitions	2,272,105
Transversions	925,261
Ts/Tv ratio	2.4556

All variants:

```
Sample ,readname,Total
Transitions ,2272105,2272105
Transversions ,925261,925261
Ts/Tv ,2.456,2.456
```

Only known variants (i.e. the ones having a non-empty ID field):

```
No results available (empty input?)
```

Allele frequency

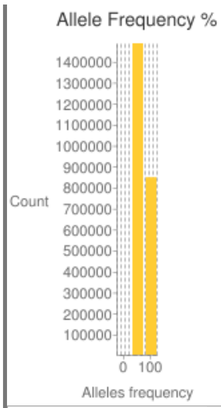

**Min**	0
**Max**	100
**Mean**	68.212
**Median**	50
**Standard deviation**	24.219
**Values**	0,50,100
**Count**	3594,1487320,859795

Allele Count

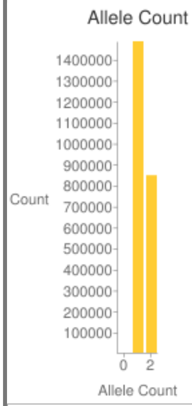

**Min**	0
**Max**	2
**Mean**	1.364
**Median**	1
**Standard deviation**	0.484
**Values**	0,1,2
**Count**	3594,1487320,859795

Hom/Het per sample

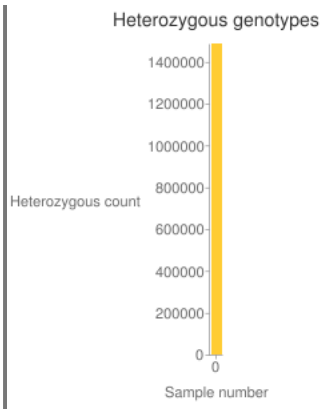

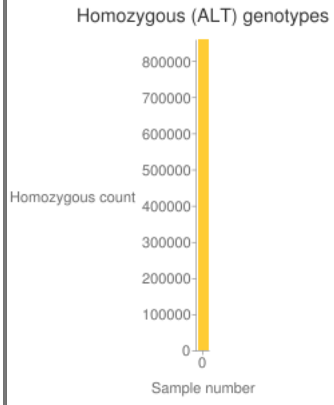

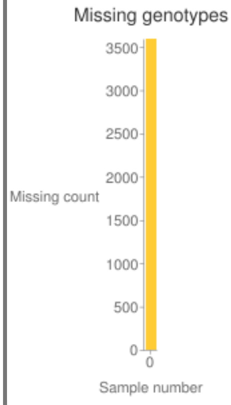


```
Sample_names , readname
Reference , 0
Het , 1487320
Hom , 859795
Missing , 3594
```

Codon changes

How to read this table:
- Rows are reference codons and columns are changed codons. E.g. Row 'AAA' column 'TAA' indicates how many 'AAA' codons have been replaced by 'TAA' codons.
- Red background colors indicate that more changes happened (heat-map).
- Diagonals are indicated using grey background color
- WARNING: This table may include different translation codon tables (e.g. mamalian DNA and mitochondrial DNA).

	AAA	AAC	AAG	AAT	ACA	ACC	ACG	ACT	AGA	AGC	AGG	AGT	ATA	ATC	ATG	ATT	CAA	CAC	CAG	CAT	CCA	CCC	C[illegible]
**AAA**		153	951	288	183				572				144				244						
**AAC**	195		184	994		100				376				60				69					
**AAG**	1,102	156		232			91				544				194				191				
**AAT**	233	785	218					196				527				162				169			
**ACA**	190					166	595	299	145				440								84		
**ACC**		131			162		101	743		122				199								44	
**ACG**			126		759	113		166			69				439								
**ACT**				208	297	648	134					215				464							
**AGA**	568				126					89	476	92	108										
**AGC**		441				114			87		84	600		72									
**AGG**			575				58		526	73		108			101								
**AGT**				602				246	80	519	98					141							
**ATA**	120				375				86					195	482	296							
**ATC**		83				167				56			195		123	845							
**ATG**			153				385				104		652	113		229							
**ATT**				176				367				120	289	689	180								
**CAA**	264																	105	767	121	117		

	AAA	AAC	AAG	AAT	ACA	ACC	ACG	ACT	AGA	AGC	AGG	AGT	ATA	ATC	ATG	ATT	CAA	CAC	CAG	CAT	CCA	CCC	C[illegible]
**CAC**		79															91		87	497		42	
**CAG**			164														858	101		117			
**CAT**				181													173	371	119				
**CCA**					99												128					128	[illegible]
**CCC**						53												40			161		
**CCG**							32												80		929	100	
**CCT**								93												95	281	548	[illegible]
**CGA**									159								559				74		
**CGC**										56								261				42	
**CGG**											135								429				
**CGT**												110								582			
**CTA**													100				100				250		
**CTC**														72				40				98	
**CTG**															137				131				[illegible]
**CTT**																223				123			
**GAA**	966																284						
**GAC**		435																52					
**GAG**			849																241				
**GAT**				915																181			
**GCA**					739																134		
**GCC**						357																56	
**GCG**							161																
**GCT**								683															
**GGA**									486														
**GGC**										306													
**GGG**											304												
**GGT**												615											
**GTA**													543										
**GTC**														425									
**GTG**															552								
**GTT**																970							
**TAA**	20																38						
**TAC**		80																166					
**TAG**			8																33				
**TAT**				159																405			
**TCA**					160																382		
**TCC**						80																146	
**TCG**							45																[illegible]
**TCT**								169															
**TGA**									8														
**TGC**										67													
**TGG**											69												
**TGT**												109											
**TTA**													129										
**TTC**														76									
**TTG**															223								
**TTT**																229							

Amino acid changes

How to read this table:
- Rows are reference amino acids and columns are changed amino acids. E.g. Row 'A' column 'E' indicates how many 'A' amino acids have been replaced by 'E' amino acids.
- Red background colors indicate that more changes happened (heat-map).
- Diagonals are indicated using grey background color
- WARNING: This table may include different translation codon tables (e.g. mamalian DNA and mitochondrial DNA).

	*	A	C	D	E	F	G	H	I	K	L	M	N	P	Q	R	S	T	V	W	Y
*	**140**		13		17		5			28	20				71	43	23			49	35
A		**5,001**		208	280		502							345			660	1,940	1,886		
C	30		**898**			201	144									461	414			91	506
D		158		**2,020**	1,043		617	233					1,350						145		278
E	145	262		961	**1,757**		697			1,815					525				221		
F			184			**1,794**			305		1,266						548		284		319
G	51	483	173	696	759		**3,676**									1,030	921		362	58	
H				227				**868**			157		260	139	470	569					674
I						288			**2,509**	120	495	785	259			86	176	909	1,405		
K	111				1,439				144	**2,053**		194	829		435	1,116		274			
L	133					1,397		163	524		**8,472**	360		933	231	205	826		804	108	
M									994	153	370					104		385	455		
N				1,055				238	222	830			**1,779**				903	296			237
P		372						135			1,136			**4,068**	208	178	1,072	277			
Q	394				492			444		428	217			184	**1,625**	839					
R	210		576				925	843	108	1,143	256	101		241	988	**2,939**	528	184		295	

	*	A	C	D	E	F	G	H	I	K	L	M	N	P	Q	R	S	T	V	W	Y
**S**	115	546	420			656	779		213		932		1,043	979		467	**5,982**	814		66	219
**T**		1,617							1,103	316		439	339	265		214	721	**4,183**			
**V**		1,613		171	206	377	330		1,938		892	552							**3,940**		
**W**	323		112				53				85					256	41				
**Y**	139		433	240		327		571					239				193				**1,273**

Variants by chromosome

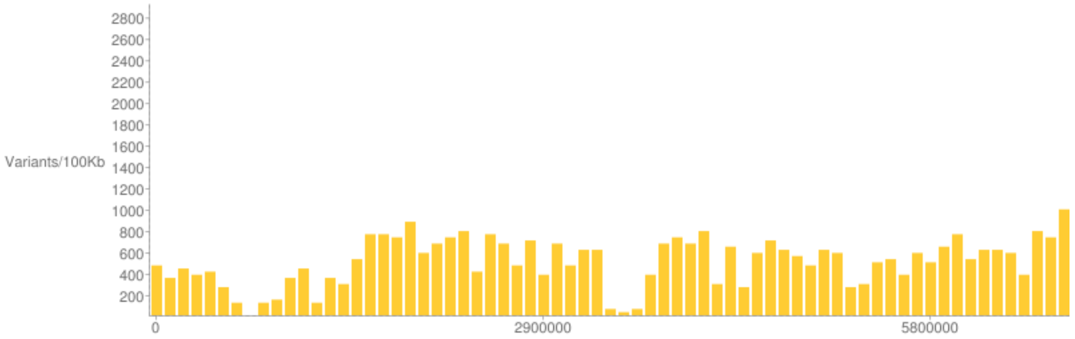

1, Position,0,100000,200000,300000,400000,500000,600000,700000,800000,900000,1000000,1100000,1200000,1300000,1400000,
1,Count,503,389,468,417,434,287,150,41,159,167,388,454,157,391,330,551,782,794,753,892,598,685,766,812,446,786,686,506,730,421,711,50

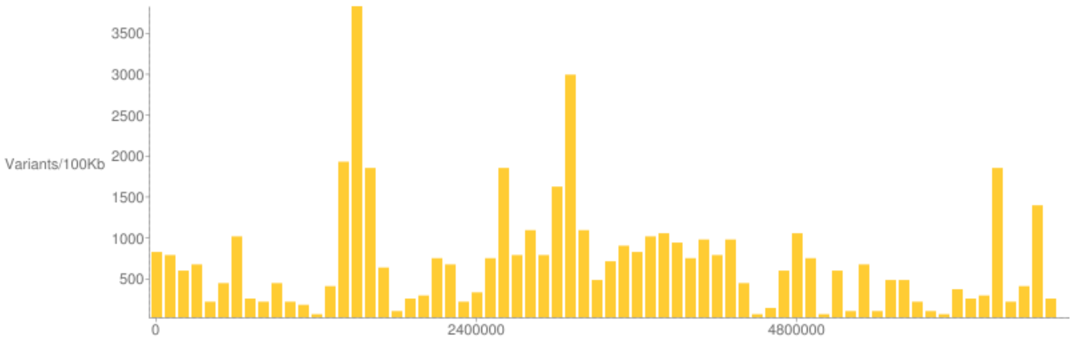

2, Position,0,100000,200000,300000,400000,500000,600000,700000,800000,900000,1000000,1100000,1200000,1300000,1400000,
2,Count,845,798,609,695,227,469,1032,262,238,465,217,210,65,412,1935,3823,1878,667,132,274,316,751,677,224,337,759,1849,801,1097,817,

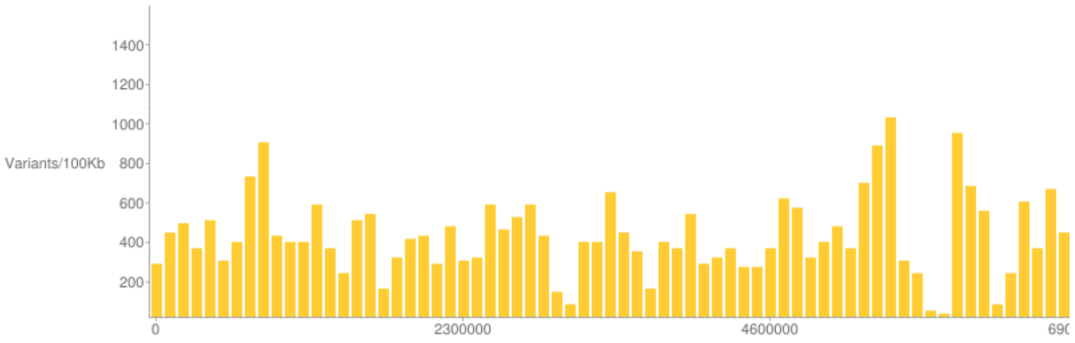

3, Position,0,100000,200000,300000,400000,500000,600000,700000,800000,900000,1000000,1100000,1200000,1300000,1400000,
3,Count,295,453,505,374,514,318,400,732,905,443,403,409,591,370,254,515,542,173,336,424,435,300,486,312,325,603,469,531,601,444,161,8

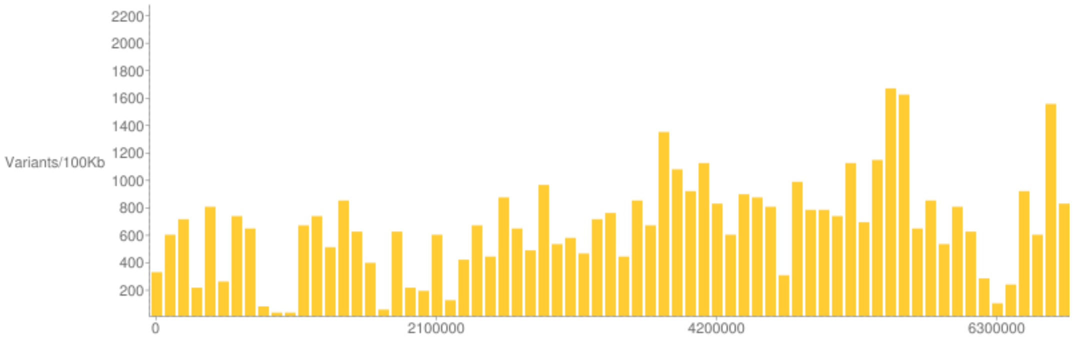

4, Position,0,100000,200000,300000,400000,500000,600000,700000,800000,900000,1000000,1100000,1200000,1300000,1400000,
4,Count,343,612,722,217,820,275,758,653,82,44,49,674,738,528,862,626,397,58,629,219,198,614,132,437,689,447,882,668,499,963,533,593,4

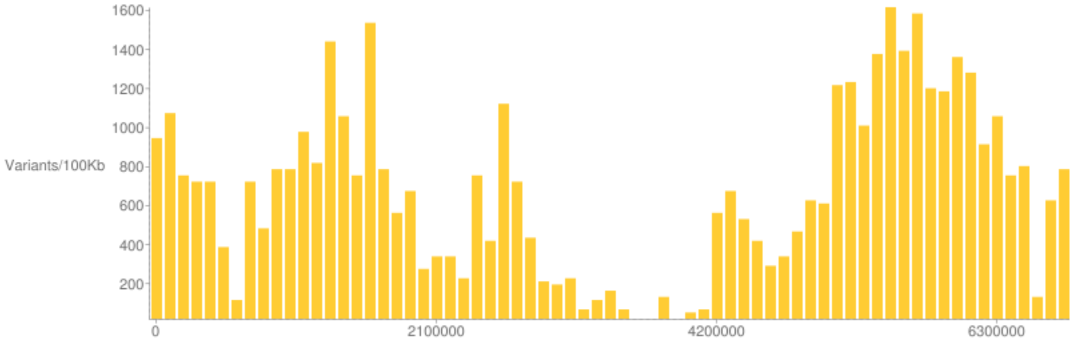

5, Position,0,100000,200000,300000,400000,500000,600000,700000,800000,900000,1000000,1100000,1200000,1300000,1400000,
5,Count,955,1072,762,722,722,399,127,734,492,784,790,979,816,1440,1058,765,1547,789,561,682,285,339,347,231,758,426,1124,721,439,218,

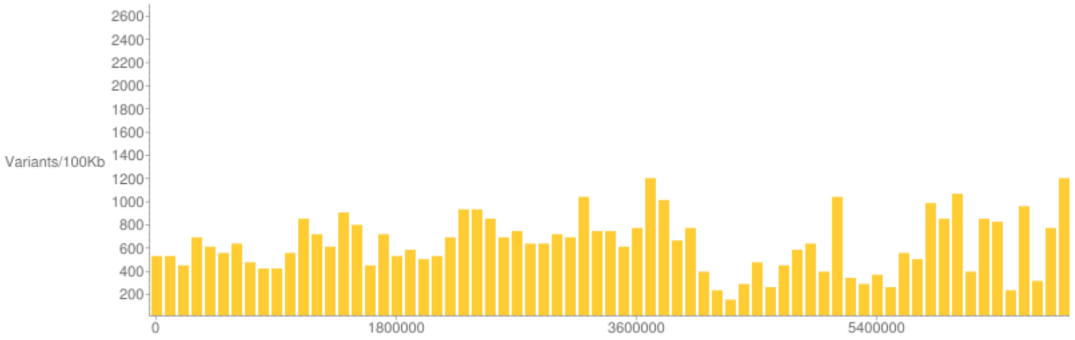

6, Position,0,100000,200000,300000,400000,500000,600000,700000,800000,900000,1000000,1100000,1200000,1300000,1400000,
6,Count,527,550,457,689,608,567,646,490,425,445,574,868,717,630,923,809,455,719,543,601,508,537,692,940,932,860,706,744,659,643,730,7

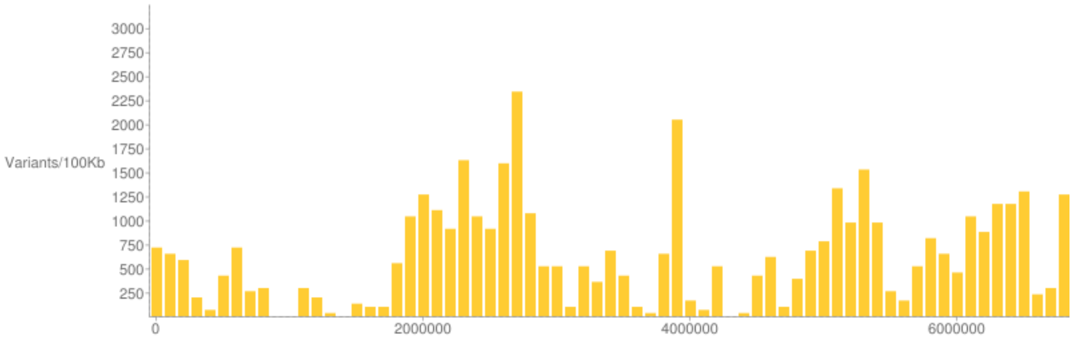

7, Position,0,100000,200000,300000,400000,500000,600000,700000,800000,900000,1000000,1100000,1200000,1300000,1400000,
7,Count,738,679,603,207,86,433,740,269,318,12,17,304,218,60,32,160,134,133,576,1064,1289,1130,931,1641,1054,917,1612,2355,1100,540,52

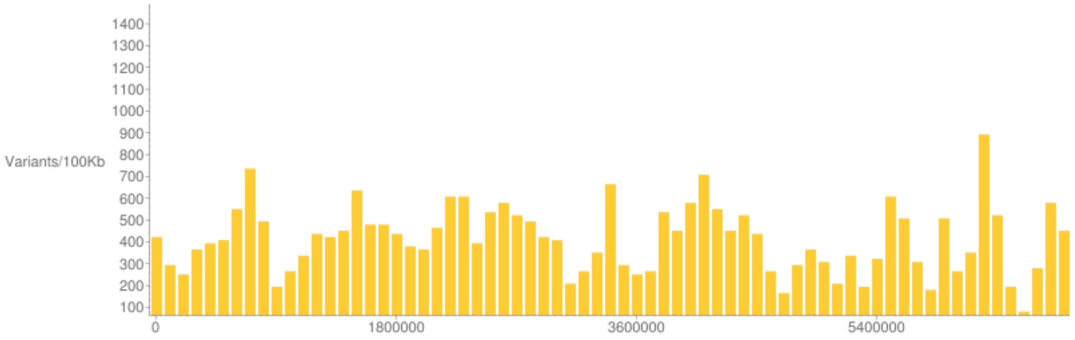

8, Position,0,100000,200000,300000,400000,500000,600000,700000,800000,900000,1000000,1100000,1200000,1300000,1400000,
8,Count,427,299,262,366,403,415,562,745,504,198,273,337,448,422,460,645,486,482,441,381,375,476,613,614,391,534,582,528,491,432,416,2

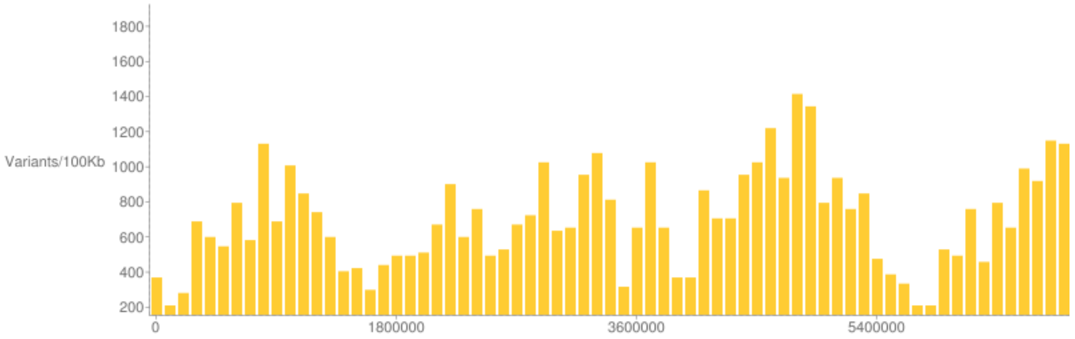

9, Position,0,100000,200000,300000,400000,500000,600000,700000,800000,900000,1000000,1100000,1200000,1300000,1400000,
9,Count,368,209,292,695,608,550,795,591,1144,696,1006,846,738,607,418,427,301,441,496,495,517,683,904,611,764,490,530,672,734,1028,63

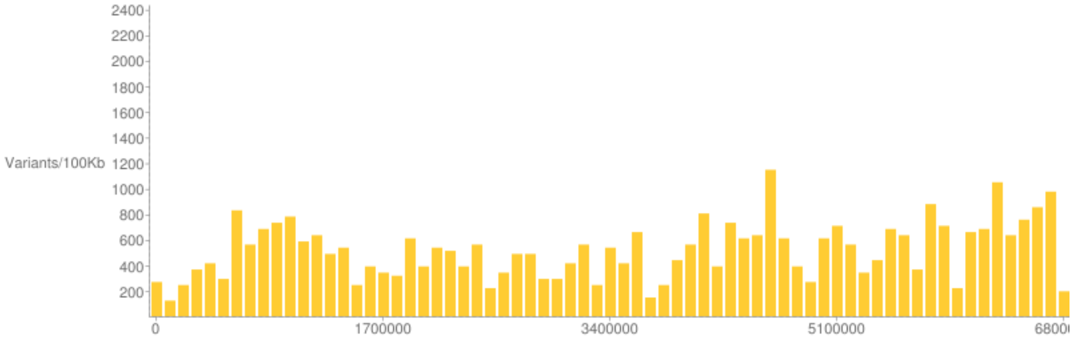

10, Position,0,100000,200000,300000,400000,500000,600000,700000,800000,900000,1000000,1100000,1200000,1300000,1400000,
10,Count,285,127,256,372,431,314,853,575,707,738,801,593,637,514,544,270,394,357,325,633,395,544,522,417,570,225,347,503,511,308,319,

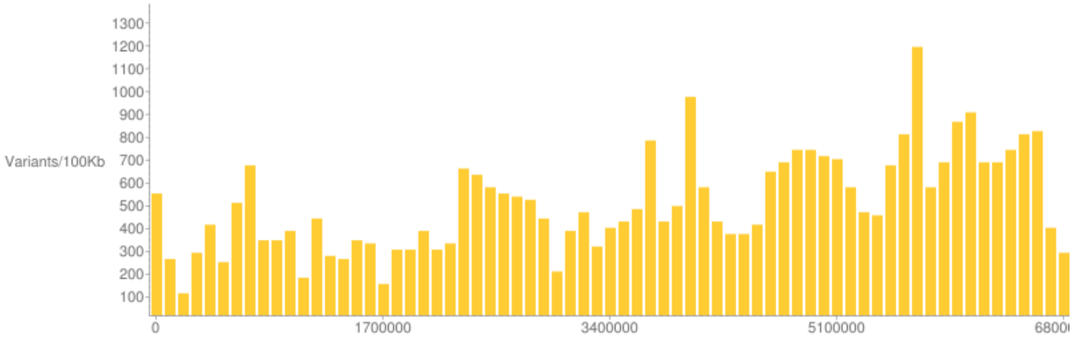

11, Position,0,100000,200000,300000,400000,500000,600000,700000,800000,900000,1000000,1100000,1200000,1300000,1400000,
11,Count,554,276,123,298,419,256,522,684,356,346,400,183,445,284,273,347,344,160,318,310,396,318,340,673,637,588,561,547,533,448,217,

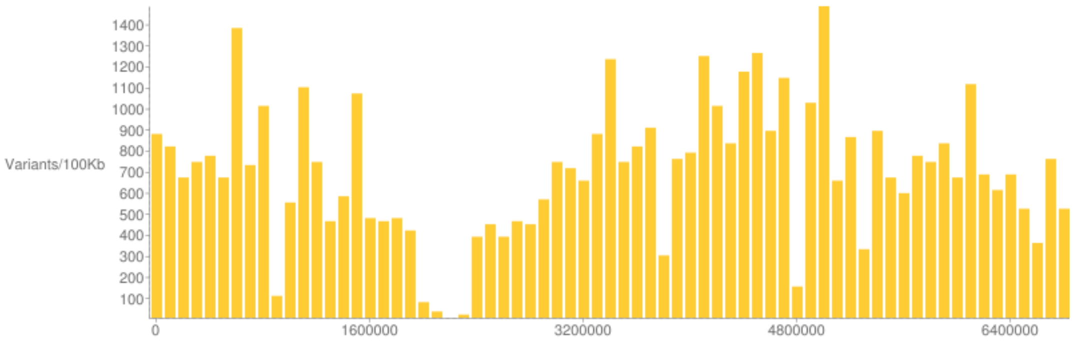

12, Position,0,100000,200000,300000,400000,500000,600000,700000,800000,900000,1000000,1100000,1200000,1300000,1400000,[illegible]
12,Count,883,821,680,750,786,685,1391,738,1014,110,554,1109,747,474,584,1078,483,471,487,424,81,37,6,32,391,456,404,470,464,578,746,7[illegible]

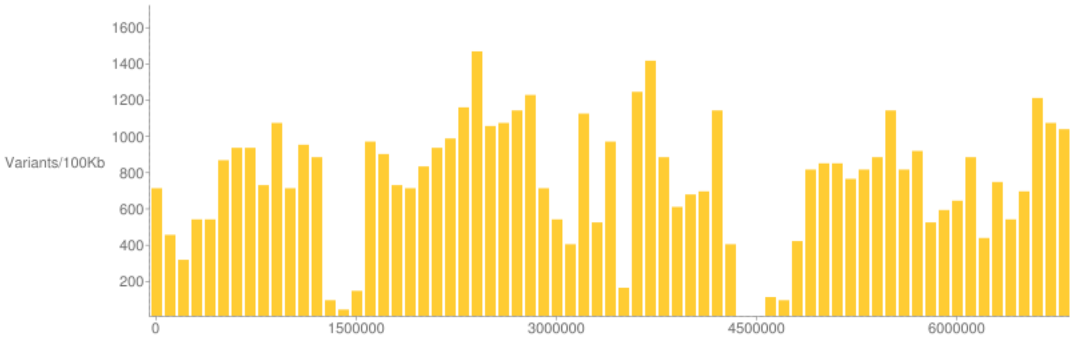

13, Position,0,100000,200000,300000,400000,500000,600000,700000,800000,900000,1000000,1100000,1200000,1300000,1400000,[illegible]
13,Count,724,466,317,554,554,867,946,948,742,1078,713,963,894,109,45,158,985,906,739,720,843,940,994,1169,1469,1054,1080,1145,1238,71[illegible]

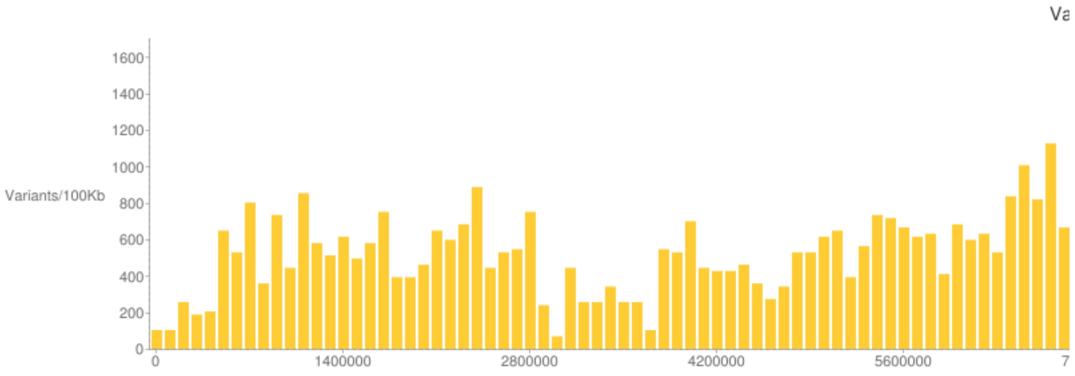

14, Position,0,100000,200000,300000,400000,500000,600000,700000,800000,900000,1000000,1100000,1200000,1300000,1400000,[illegible]
14,Count,111,117,257,199,216,660,532,811,364,734,454,866,588,520,626,509,584,760,408,398,466,658,605,690,887,455,543,548,751,253,79,4[illegible]

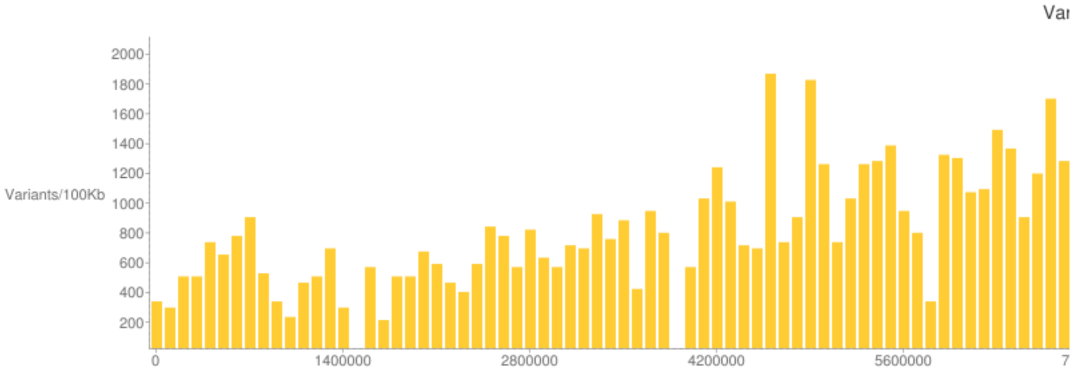

15, Position,0,100000,200000,300000,400000,500000,600000,700000,800000,900000,1000000,1100000,1200000,1300000,1400000,[illegible]
15,Count,349,312,506,506,754,654,781,922,534,347,234,475,514,705,302,23,571,231,508,520,678,603,476,402,607,848,788,582,826,638,583,7[illegible]

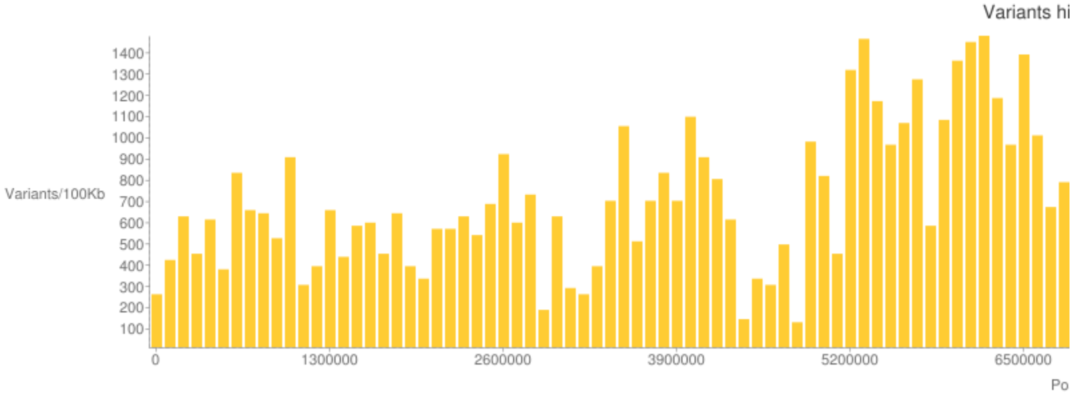

16, Position,0,100000,200000,300000,400000,500000,600000,700000,800000,900000,1000000,1100000,1200000,1300000,1400000,
16,Count,267,426,632,458,623,385,836,661,656,532,918,309,407,669,450,591,611,457,650,400,345,570,581,634,543,700,929,598,742,197,628,

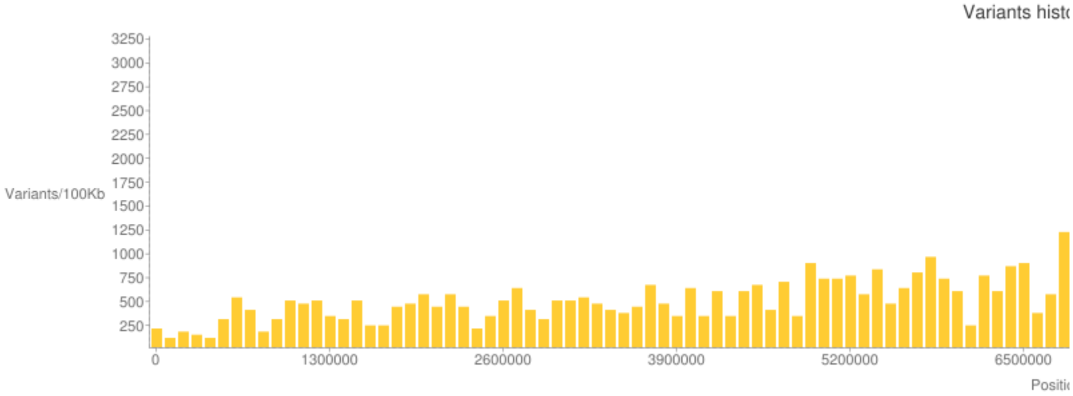

17, Position,0,100000,200000,300000,400000,500000,600000,700000,800000,900000,1000000,1100000,1200000,1300000,1400000,
17,Count,218,114,202,158,123,322,540,417,210,324,534,491,508,351,339,514,266,269,445,491,594,453,583,453,241,342,511,655,411,321,524,

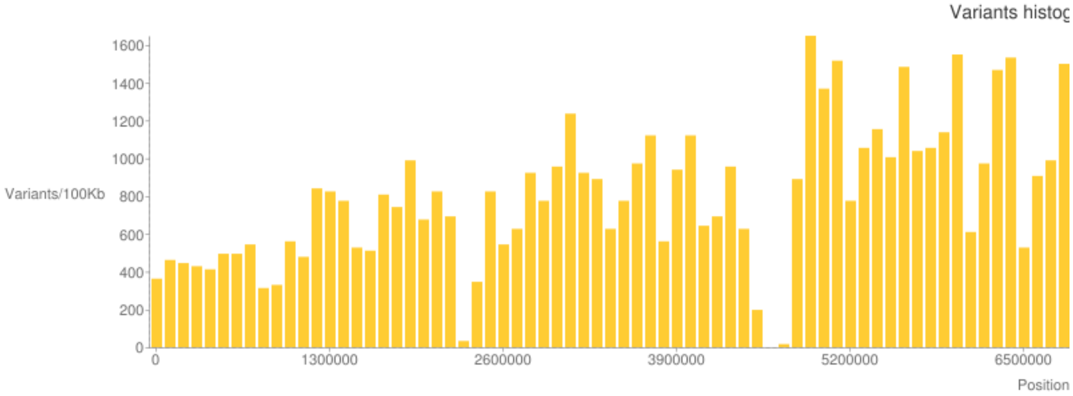

18, Position,0,100000,200000,300000,400000,500000,600000,700000,800000,900000,1000000,1100000,1200000,1300000,1400000,
18,Count,370,462,447,441,422,506,500,556,329,333,573,485,842,825,778,534,518,815,749,992,682,831,700,49,354,827,549,627,923,777,962,

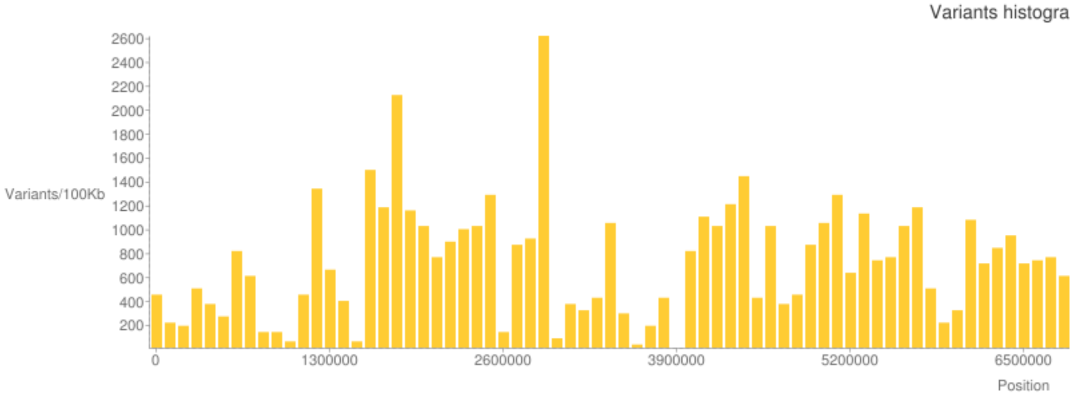

19, Position,0,100000,200000,300000,400000,500000,600000,700000,800000,900000,1000000,1100000,1200000,1300000,1400000,
19,Count,462,226,215,514,398,287,836,611,142,150,64,454,1343,687,413,76,1510,1203,2140,1161,1053,774,911,1025,1050,1305,160,889,947,

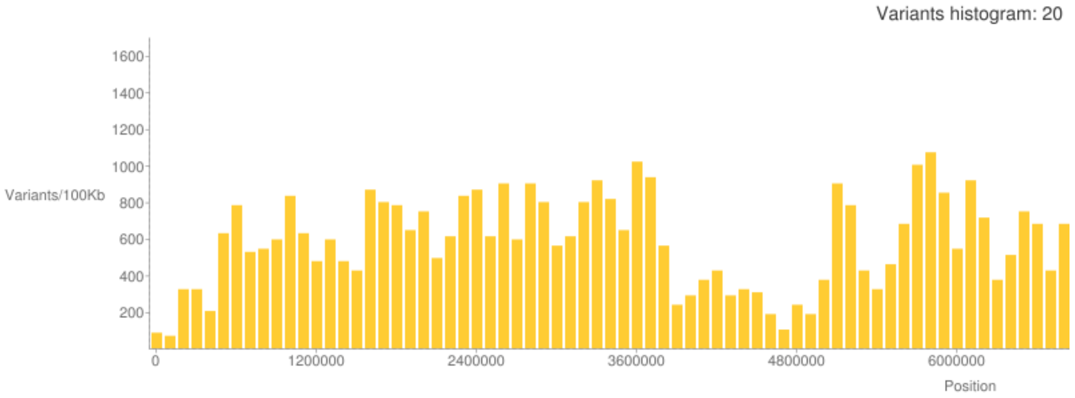

20, Position,0,100000,200000,300000,400000,500000,600000,700000,800000,900000,1000000,1100000,1200000,1300000,1400000,
20,Count,95,76,336,334,221,637,796,539,557,605,835,647,480,599,480,437,884,815,786,652,755,510,621,843,878,617,911,609,908,807,564,62

Details by gene

Here you can find a tab-separated table.

SnpEff: Variant analysis

Contents

Summary
Variant rate by chromosome
Variants by type
Number of variants by impact
Number of variants by functional class
Number of variants by effect
Quality histogram
InDel length histogram
Base variant table
Transition vs transversions (ts/tv)
Allele frequency
Allele Count
Codon change table
Amino acid change table
Chromosome variants plots
Details by gene

Summary

**Genome**	manindi
**Date**	2021-05-22 14:42
**SnpEff version**	`SnpEff 5.0e (build 2021-03-09 06:01), by Pablo Cingolani`
**Command line arguments**	`SnpEff manindi /home/cocogenomics/mango_genome/mango_ref_genome/M.indica_ref_INDEL_final.vcf`
**Warnings**	52,230
**Errors**	0
**Number of lines (input file)**	399,041
**Number of variants (before filter)**	399,786
**Number of not variants (i.e. reference equals alternative)**	0
**Number of variants processed (i.e. after filter and non-variants)**	399,786
**Number of known variants (i.e. non-empty ID)**	0 (0%)
**Number of multi-allelic VCF entries (i.e. more than two alleles)**	745
**Number of effects**	875,658
**Genome total length**	391,108,416
**Genome effective length**	357,440,369
**Variant rate**	1 variant every 894 bases

Variants rate details

Chromosome	Length	Variants	Variants rate
1	29,456,600	34,099	863
2	24,397,897	28,332	861
3	23,139,393	22,887	1,011
4	21,507,500	22,147	971
5	21,083,371	22,208	949
6	18,811,960	25,999	723
7	20,623,120	23,591	874
8	18,243,469	16,785	1,086
9	18,233,274	22,830	798
10	17,652,500	20,349	867
11	17,144,574	16,625	1,031
12	16,029,966	18,109	885
13	15,457,994	17,059	906
14	14,810,209	15,698	943
15	14,765,960	18,724	788
16	13,817,227	15,396	897
17	13,506,273	15,743	857
18	13,371,754	15,814	845
19	13,093,066	15,251	858
20	12,294,262	12,140	1,012
**Total**	**357,440,369**	**399,786**	**894**

Number variants by type

Type	Total
**SNP**	0
**MNP**	0
**INS**	199,857
**DEL**	199,929
**MIXED**	0
**INV**	0
**DUP**	0
**BND**	0
**Total**	**399,786**

Type	Total
INTERVAL	0
Total	399,786

Number of effects by impact

Type (alphabetical order)	Count	Percent
HIGH	6,364	0.727%
LOW	1,493	0.171%
MODERATE	2,812	0.321%
MODIFIER	864,989	98.782%

Number of effects by functional class

Type (alphabetical order)	Count	Percent

Missense / Silent ratio: 0

Number of effects by type and region

Type

Type (alphabetical order)	Count	Percent
3_prime_UTR_variant	4,744	0.54%
5_prime_UTR_truncation	3	0%
5_prime_UTR_variant	2,987	0.34%
bidirectional_gene_fusion	1	0%
conservative_inframe_deletion	550	0.063%
conservative_inframe_insertion	643	0.073%
disruptive_inframe_deletion	1,026	0.117%
disruptive_inframe_insertion	682	0.078%
downstream_gene_variant	225,389	25.666%
exon_loss_variant	3	0%
frameshift_variant	6,054	0.689%
intergenic_region	318,804	36.304%
intragenic_variant	4	0%
intron_variant	65,064	7.409%
non_coding_transcript_variant	74	0.008%
splice_acceptor_variant	129	0.015%
splice_donor_variant	178	0.02%
splice_region_variant	1,851	0.211%
start_lost	90	0.01%
start_retained_variant	7	0.001%
stop_gained	188	0.021%
stop_lost	88	0.01%
stop_retained_variant	8	0.001%
upstream_gene_variant	249,591	28.422%

Region

Type (alphabetical order)	Count	Percent
DOWNSTREAM	225,381	25.738%
EXON	8,950	1.022%
GENE	1	0%
INTERGENIC	318,804	36.407%
INTRON	63,392	7.239%
SPLICE_SITE_ACCEPTOR	96	0.011%
SPLICE_SITE_DONOR	141	0.016%
SPLICE_SITE_REGION	1,493	0.171%
TRANSCRIPT	78	0.009%
UPSTREAM	249,591	28.503%
UTR_3_PRIME	4,741	0.541%
UTR_5_PRIME	2,990	0.341%

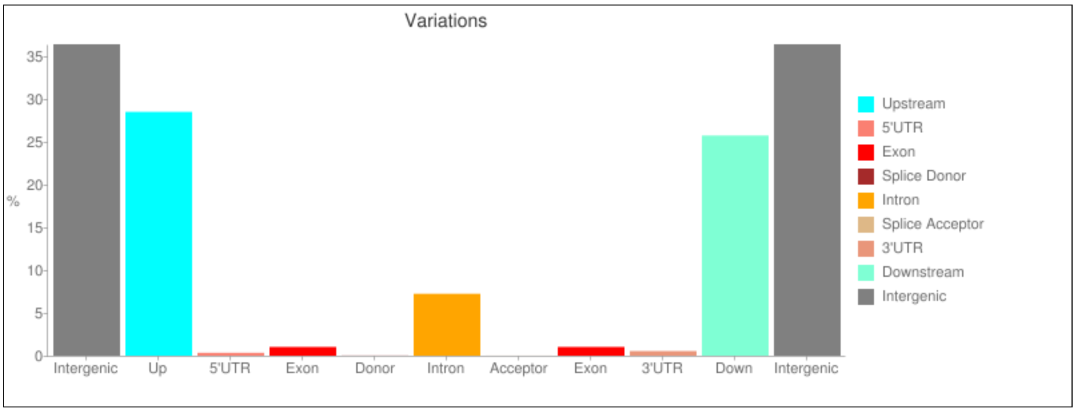

Quality:

**Min**	10
**Max**	85,145
**Mean**	112.927
**Median**	85
**Standard deviation**	402.288
**Values**	10,11,12,13,14,15,16,17,18,19,20,21,22,23,24,25,26,27,28,29,30,31,32,33,34,35,36,37,38,39,40,41,42,43,44,45,46,47,48,49,50,51,52,53,54,55,56,57
**Count**	1393,1986,2514,1877,1541,1508,1873,1611,1482,2104,1527,1595,2499,1624,1526,2979,1461,1464,4099,1496,1522,5952,1888,1627,7646,1374,11[illegible]

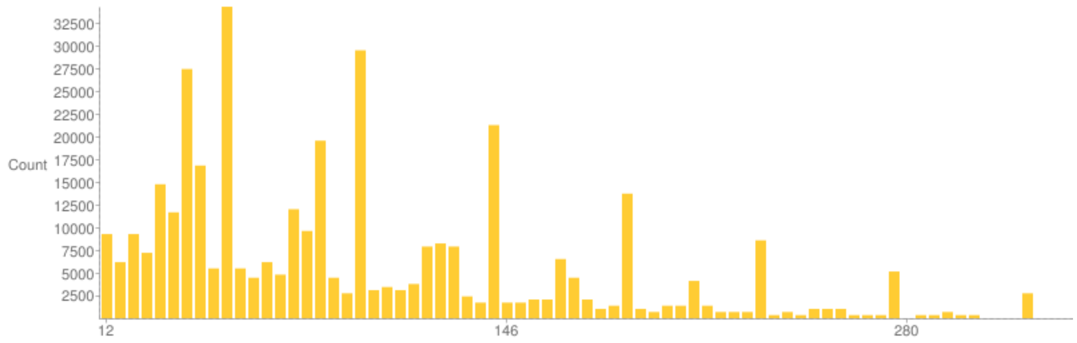

Insertions and deletions length:

**Min**	0
**Max**	214
**Mean**	2.118
**Median**	1
**Standard deviation**	5.67
**Values**	0,1,2,3,4,5,6,7,8,9,10,11,12,13,14,15,16,17,18,19,20,21,22,23,24,25,26,27,28,29,30,31,32,33,34,35,36,37,38,39,40,41,42,43,44,45,46,47,48,49,50,51
**Count**	100879,233788,14256,9656,5484,4564,2974,2921,2624,2531,2041,2198,1562,1329,1173,1063,833,893,719,687,647,522,454,449,351,345,381,321,[illegible]

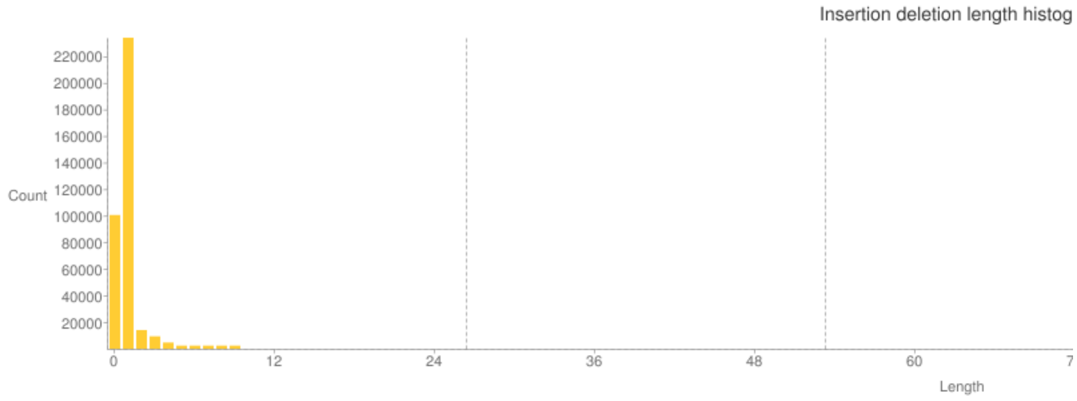

Base changes (SNPs)

	A	C	G	T
**A**	0	0	0	0
**C**	0	0	0	0
**G**	0	0	0	0
**T**	0	0	0	0

Ts/Tv (transitions / transversions)

Note: Only SNPs are used for this statistic.
Note: This Ts/Tv ratio is a 'raw' ratio (ratio of observed events).

**Transitions**	0
**Transversions**	0
**Ts/Tv ratio**	0

All variants:

```
No results available (empty input?)
```

Only known variants (i.e. the ones having a non-empty ID field):

```
No results available (empty input?)
```

Allele frequency

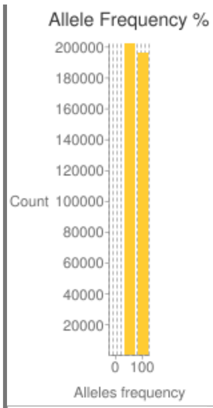

**Min**	0
**Max**	100
**Mean**	74.611
**Median**	50
**Standard deviation**	25.068
**Values**	0,50,100
**Count**	283,202056,196702

Allele Count

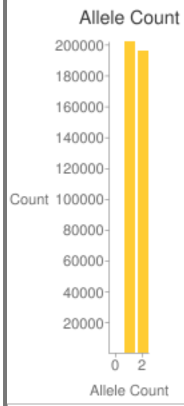

**Min**	0
**Max**	2
**Mean**	1.492
**Median**	1
**Standard deviation**	0.501
**Values**	0,1,2
**Count**	283,202056,196702

Hom/Het per sample

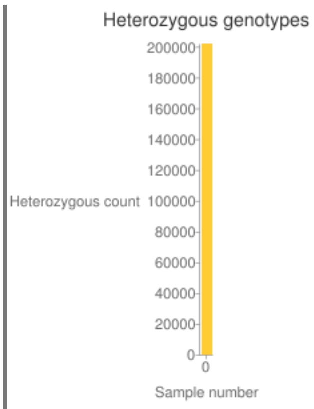

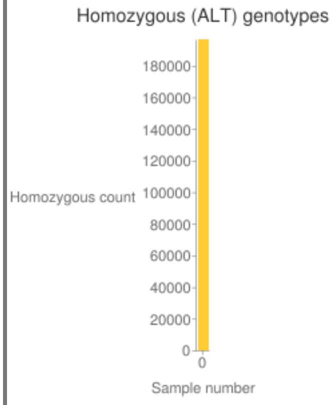

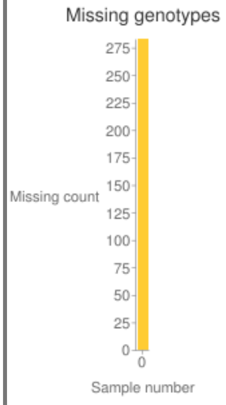


```
Sample_names , readname
Reference , 0
Het , 202056
Hom , 196702
Missing , 283
```

Codon changes

How to read this table:
- Rows are reference codons and columns are changed codons. E.g. Row 'AAA' column 'TAA' indicates how many 'AAA' codons have been replaced by 'TAA' codons.
- Red background colors indicate that more changes happened (heat-map).
- Diagonals are indicated using grey background color
- WARNING: This table may include different translation codon tables (e.g. mamalian DNA and mitochondrial DNA).

	-	AAA	AAC	AAG	AAT	ACA	ACC	ACG	ACT	AGA	AGC	AGG	AGT	ATA	ATC	ATG	ATT	CAA	CAC	CAG	CAT	CCA	CC
**-**		191	85	156	174	111	57	24	105	79	75	67	86	84	70	97	108	135	43	105	129	162	4
**AAA**	307	**37**	10	36	14	2		1	2	11	4	2	1	3	4	6	1	3		2		1	
**AAC**	175	14	**7**	14	18	3		1	2	2	4		1	2	1	5		1		1			
**AAG**	329	56	15	**18**	12	4	2	4		13	3	6	1	1		6	3	6		1			
**AAT**	280	40	15	10	**24**	5	1	1	4	10	5	1	3	7	1	9	3	1		1	1		
**ACA**	177	2	9	2	1	**14**	9	5	15	1	6	1	1	3	2	2	4	1	1				
**ACC**	113	3	9	2		8	**8**	3	11	1	2		1	1	4	2	1		3				
**ACG**	60	2	5			4	3	**10**	8			1	2				1						
**ACT**	175	3	12	5	7	15	9	2	**6**	3	3		5	4	2				1				
**AGA**	201	8	1	13		3	1	1		**8**	7	18	8	1	2	6	1	1					
**AGC**	105	1	4	10	3	1		2		7	**4**	5	7	1		2	1			2			
**AGG**	124	1	4	10	2	1	1	4	4	18	4	**14**	6	2	1	3				1			
**AGT**	179	4	1	10	6	3			5	8	6	9	**8**	2		3	1			1			
**ATA**	136	1	1	1	13	3	1		2	3		1	3	**7**	4	14	9	2					
**ATC**	94	2		3	7	3	3		3	2	3		1	3	**9**	5	8				3		
**ATG**	250	6	2	5	26	5	1	2	3	4			2	18	8	**17**	13				1		
**ATT**	170	1	3	2	14		6	1	8	1	1	1	4	7	14	12	**12**				1		

	-	AAA	AAC	AAG	AAT	ACA	ACC	ACG	ACT	AGA	AGC	AGG	AGT	ATA	ATC	ATG	ATT	CAA	CAC	CAG	CAT	CCA	CC
**CAA**	227	1	1	2		7	1			1							2	**13**	9	23	12	13	
**CAC**	98																	4	**1**	7	11	6	
**CAG**	147			1													1	22	6	**15**	9	6	
**CAT**	173					1												13	13	8	**10**	9	
**CCA**	199					1	3											3	2	2	5	**15**	1
**CCC**	79		2		1													2	1		1	5	**15**
**CCG**	76																		2	2		19	1
**CCT**	221			1			2			1			1					3	3	4	3	23	2
**CGA**	49							1										3		5	1	2	
**CGC**	38																		1	1	1		
**CGG**	48		1		1												1	2		3	1		
**CGT**	74			1															1		4	1	
**CTA**	94								1									1	1	1	3	1	
**CTC**	106								1									1	2	1	2	2	
**CTG**	122								3									2	1	1	5		
**CTT**	208		1						1			1						6		1	5		
**GAA**	418	2		1	1					6		1				1							
**GAC**	141		2	1						5				1									
**GAG**	312			8	1		1			3		1						1					
**GAT**	360			4	2					6		3				1					2		
**GCA**	207	1						1			2											2	
**GCC**	107		1				3				2					1	1					1	
**GCG**	54			2							1												
**GCT**	268			2					1		3			1						1			
**GGA**	215	1								2		3								1			
**GGC**	110			2	1						1	1	1							1			
**GGG**	141											3	1								1		
**GGT**	199			1					1		1	1		1		2					1		
**GTA**	123												1										
**GTC**	92			2																			
**GTG**	172											1				3							
**GTT**	256			1				1		1			3		1	1				1		1	
**TAA**	21																						
**TAC**	88			1																			
**TAG**	16																						
**TAT**	161													3			1	1					
**TCA**	216	1				1									1		1					1	
**TCC**	114				1										1				1	1		1	
**TCG**	66																						
**TCT**	267	1		1	1		1		1						2			1			1		
**TGA**	27			1												1							
**TGC**	75	1	1												1	2							
**TGG**	103												1			1							
**TGT**	124	1			2														1				
**TTA**	169	1	1	1													3						
**TTC**	146													1				1				1	
**TTG**	219								1					1			3						
**TTT**	257		1		2								1				4	1					

Amino acid changes

How to read this table:
- Rows are reference amino acids and columns are changed amino acids. E.g. Row 'A' column 'E' indicates how many 'A' amino acids have been replaced by 'E' amino acids.
- Red background colors indicate that more changes happened (heat-map).
- Diagonals are indicated using grey background color
- WARNING: This table may include different translation codon tables (e.g. mamalian DNA and mitochondrial DNA).

	*	-	?	A	C	D	E	F	G	H	I	K	L	M	N	P	Q	R	S	T	V	W	Y
*	**13**	62	2					1				1	16	1				1	7			2	7
-	123		2,899	298	127	286	363	226	356	172	262	347	433	97	259	360	240	239	579	297	271	64	132
?																							
A		636		**121**	4	24	21	1	45		2	5		1	1	4	1	4	11	5	34		
C	6	199		2	**20**			9	1	1	1	2	17	2	3			2	18		4	11	5
D	5	501		24	1	**68**	72		31	2	1	5	1	1	4			16			24		1
E	8	730		17		84	**137**	1	67			11	3	1	2		1	15	3	1	27	2	1
F	4	403		1	11			**89**			5		38		3	2	2	1	35		8	3	17
G	1	665		23	3	24	47		**209**	2	1	4	3	2	1		2	14	4	1	33	5	
H		271		2		2		1	3	**35**			10			25	32	12	1	1			5
I	3	400		1		3	1	2	2	4	**73**	10	5	31	38		2	8	16	30	4	2	5
K	8	636		1		2	14	3	5		12	**147**	4	12	51	1	12	32	10	15			
L	6	918		6	10	1	3	77	2	19	7	2	**179**		2	52	14	18	45	7	8	2	9
M	2	250			1	4	1		1	1	39	11	2	**17**	28			5	3	11	2	1	3
N	1	453	2	3		1	5	1	5	1	14	78	1	14	**64**	1	4	14	16	17			

	*	-	?	A	C	D	E	F	G	H	I	K	L	M	N	P	Q	R	S	T	V	W	Y
**P**		574	1	8		1	1	3	1	17		1	37		3	**196**	16	16	14	6	2		2
**Q**		374		7	1		3		2	36	3	4	16		1	32	**73**	15	6	8	2		
**R**	6	534		5		1	4	1	6	9	8	33	18	9	9	16	16	**101**	30	16			1
**S**	14	947		3	12	2	2	66	1	2	10	28	24	5	16	6	5	29	**241**	14	5	1	15
**T**		525		5		2	3	1	2	5	22	19	3	4	43	3	1	7	20	**130**	2		3
**V**	1	643		25	6	22	24	2	47		1	3	2	4		1	1	6	5	1	**117**	1	
**W**	8	103			11		2	1					9	1					4		2	**7**	2
**Y**	23	249			12		1	5	2		4	1	23				1	1	10		4	3	**35**

Variants by chromosome

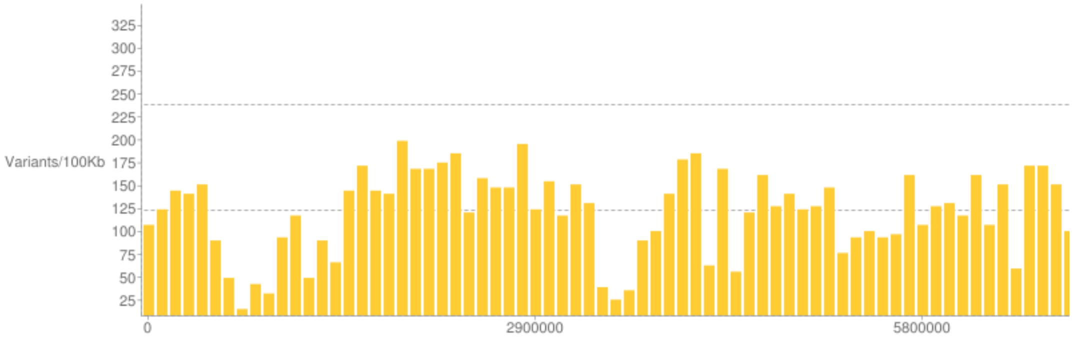

1, Position,0,100000,200000,300000,400000,500000,600000,700000,800000,900000,1000000,1100000,1200000,1300000,1400000,
1,Count,107,124,145,142,151,90,52,17,42,34,93,119,49,92,68,145,173,147,142,200,170,169,177,185,122,160,149,150,196,126,156,120,153,13

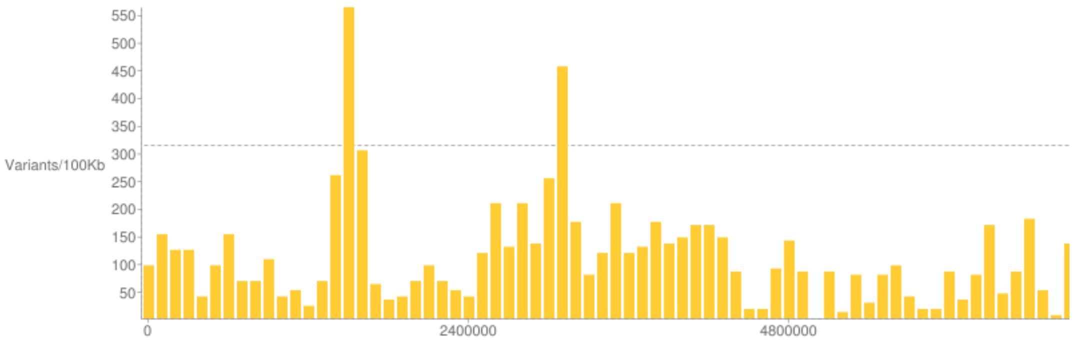

2, Position,0,100000,200000,300000,400000,500000,600000,700000,800000,900000,1000000,1100000,1200000,1300000,1400000,
2,Count,98,154,128,127,45,102,159,70,70,111,42,55,29,74,266,564,310,69,38,43,72,102,75,57,42,124,214,132,212,137,255,458,178,84,123,2

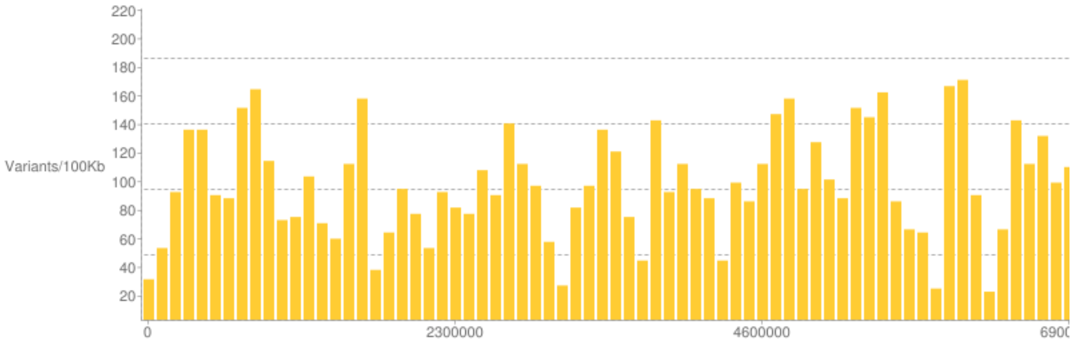

3, Position,0,100000,200000,300000,400000,500000,600000,700000,800000,900000,1000000,1100000,1200000,1300000,1400000,
3,Count,33,54,93,138,138,91,89,153,165,115,74,76,105,71,60,113,158,38,66,95,78,55,94,83,79,108,92,142,114,97,59,28,83,98,138,121,75,4

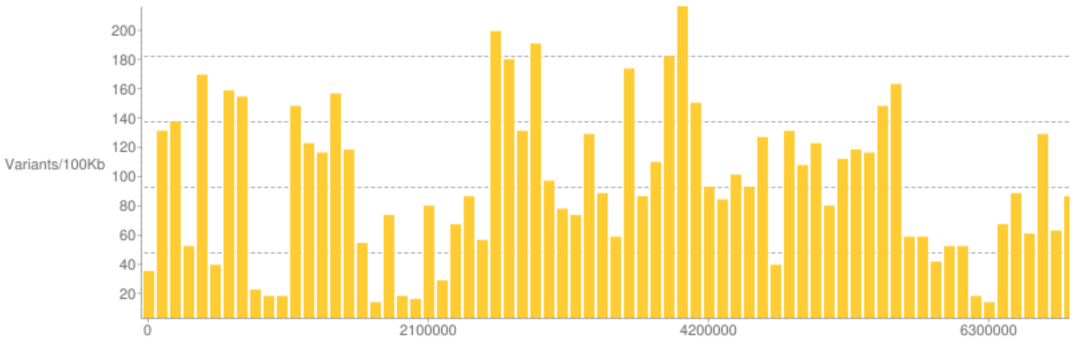

4, Position,0,100000,200000,300000,400000,500000,600000,700000,800000,900000,1000000,1100000,1200000,1300000,1400000,[illegible]
4,Count,37,131,138,53,171,41,160,156,24,19,20,149,124,116,158,120,55,15,74,19,17,81,30,67,88,58,199,181,131,191,97,79,75,129,90,60,17[illegible]

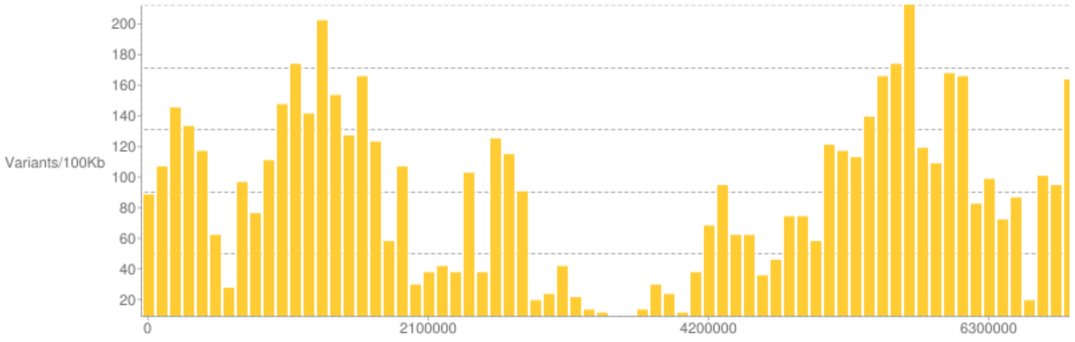

5, Position,0,100000,200000,300000,400000,500000,600000,700000,800000,900000,1000000,1100000,1200000,1300000,1400000,[illegible]
5,Count,89,108,146,133,118,62,28,97,77,112,148,174,141,202,155,127,166,124,58,107,31,38,42,39,104,38,125,116,91,21,24,42,23,15,13,9,1[illegible]

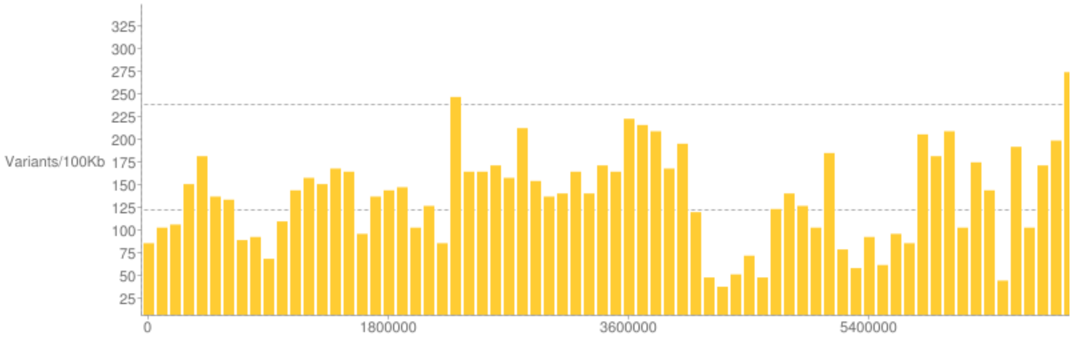

6, Position,0,100000,200000,300000,400000,500000,600000,700000,800000,900000,1000000,1100000,1200000,1300000,1400000,[illegible]
6,Count,85,103,108,151,181,138,133,91,95,69,112,146,160,152,169,165,96,138,144,150,104,127,87,247,165,167,174,160,215,155,137,140,165,[illegible]

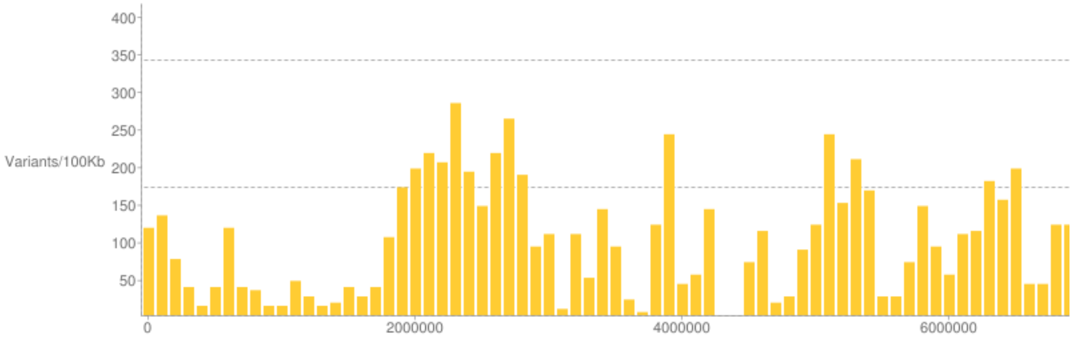

7, Position,0,100000,200000,300000,400000,500000,600000,700000,800000,900000,1000000,1100000,1200000,1300000,1400000,[illegible]
7,Count,120,136,81,41,17,43,121,44,40,19,17,49,32,16,22,41,29,43,108,176,199,221,209,287,195,151,219,266,192,97,113,14,112,53,145,96,[illegible]

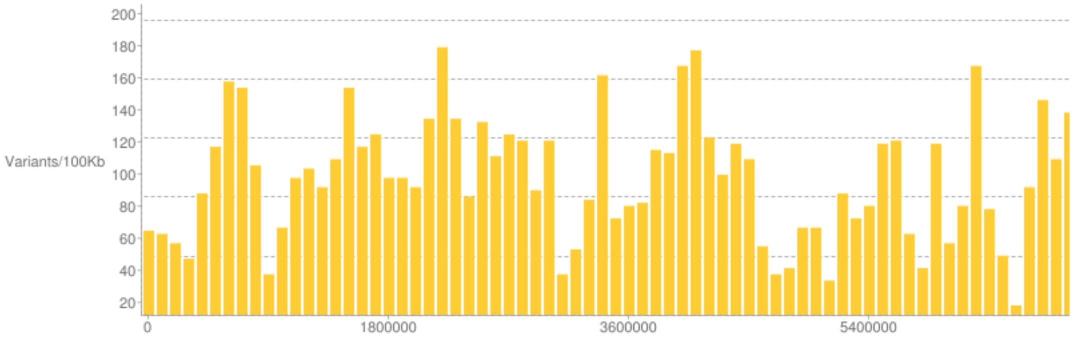

8, Position,0,100000,200000,300000,400000,500000,600000,700000,800000,900000,1000000,1100000,1200000,1300000,1400000,
8,Count,65,64,58,48,88,117,158,155,106,39,67,99,105,92,110,155,117,126,99,98,92,136,180,135,86,134,111,126,121,91,122,39,53,84,163,74,

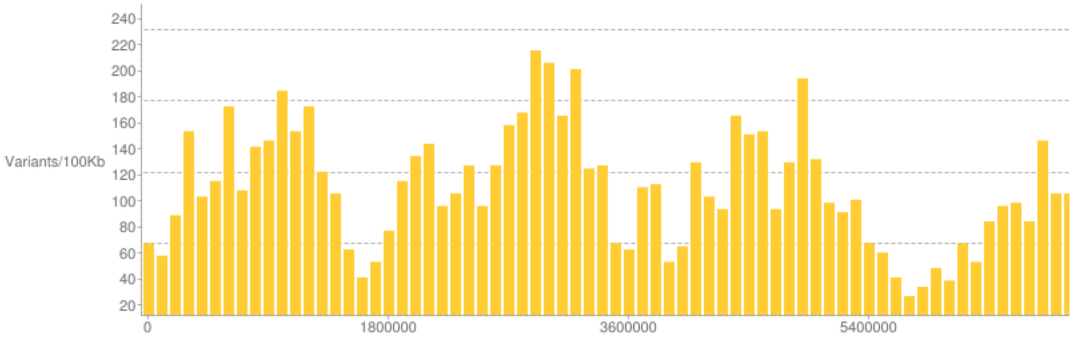

9, Position,0,100000,200000,300000,400000,500000,600000,700000,800000,900000,1000000,1100000,1200000,1300000,1400000,
9,Count,68,58,90,154,105,117,174,108,142,148,186,155,174,124,106,63,43,55,78,115,134,144,98,107,129,98,128,160,168,216,207,167,202,12

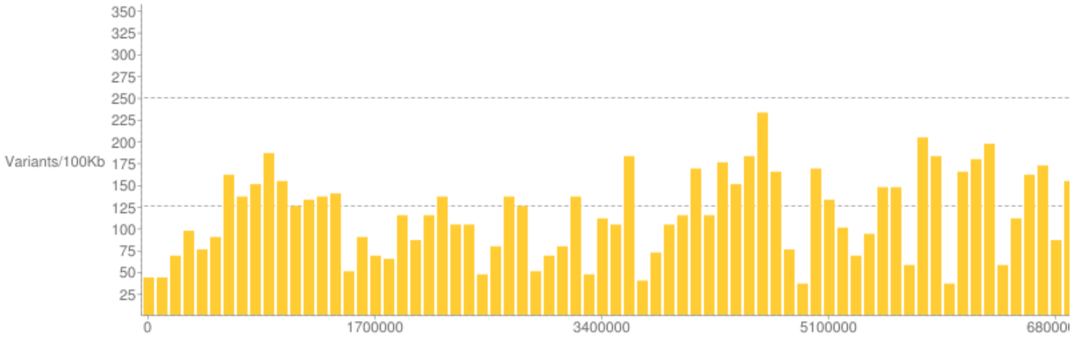

10, Position,0,100000,200000,300000,400000,500000,600000,700000,800000,900000,1000000,1100000,1200000,1300000,1400000,
10,Count,45,46,72,99,78,93,162,140,152,187,157,126,135,139,142,54,91,70,67,117,90,116,138,107,105,50,82,138,126,51,70,80,137,48,115,1

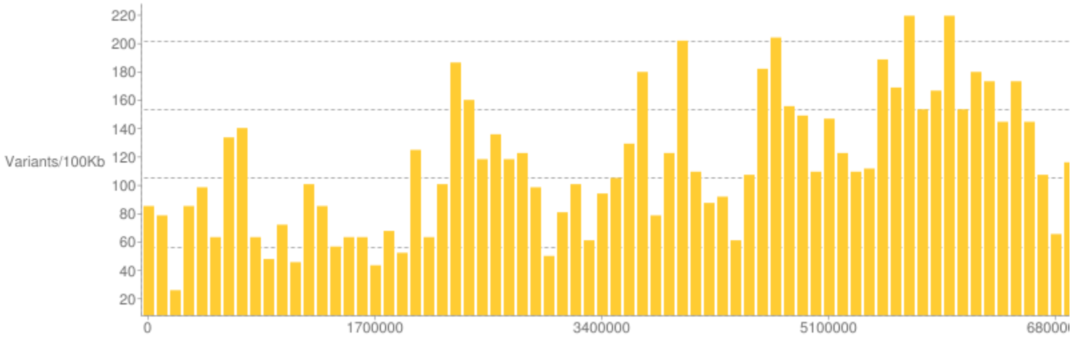

11, Position,0,100000,200000,300000,400000,500000,600000,700000,800000,900000,1000000,1100000,1200000,1300000,1400000,
11,Count,86,80,26,86,100,63,134,140,65,49,73,46,101,87,57,64,65,44,68,52,126,65,101,188,160,120,136,120,123,99,51,81,102,61,95,106,12

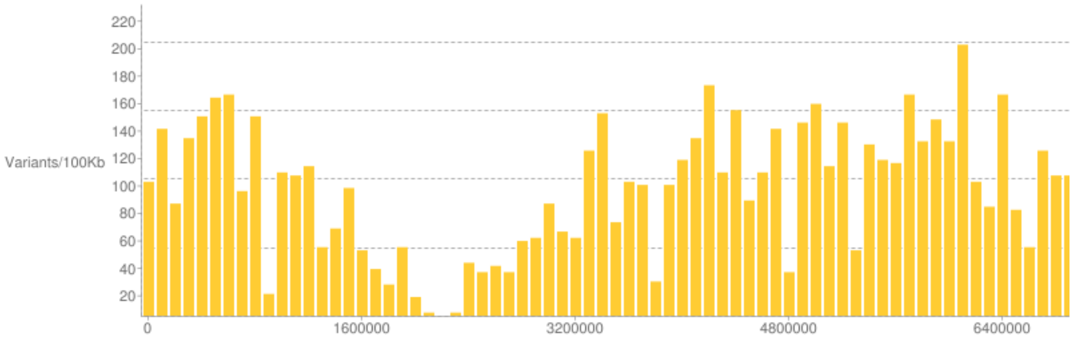

12, Position,0,100000,200000,300000,400000,500000,600000,700000,800000,900000,1000000,1100000,1200000,1300000,1400000,
12,Count,103,142,87,135,152,166,168,97,151,23,111,108,114,55,70,100,54,40,29,55,19,8,5,8,44,38,43,37,60,62,88,67,64,127,153,74,103,10

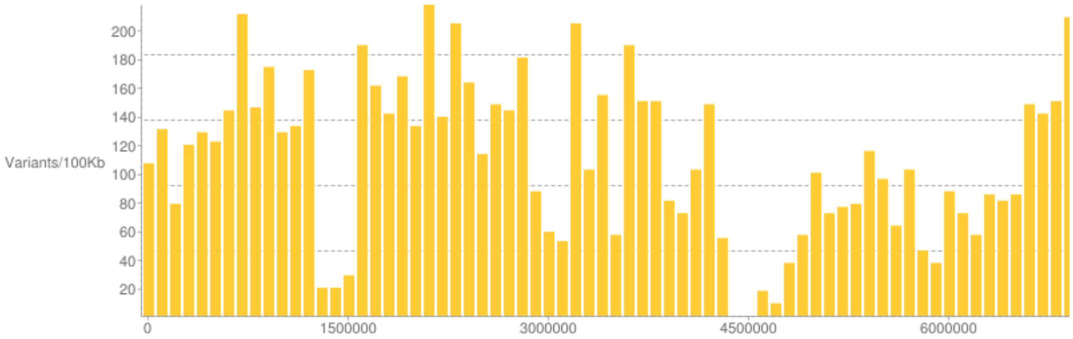

13, Position,0,100000,200000,300000,400000,500000,600000,700000,800000,900000,1000000,1100000,1200000,1300000,1400000,
13,Count,109,132,80,122,131,124,145,212,148,175,131,135,174,21,21,30,191,162,143,169,135,218,140,205,165,116,150,146,182,88,61,54,205,

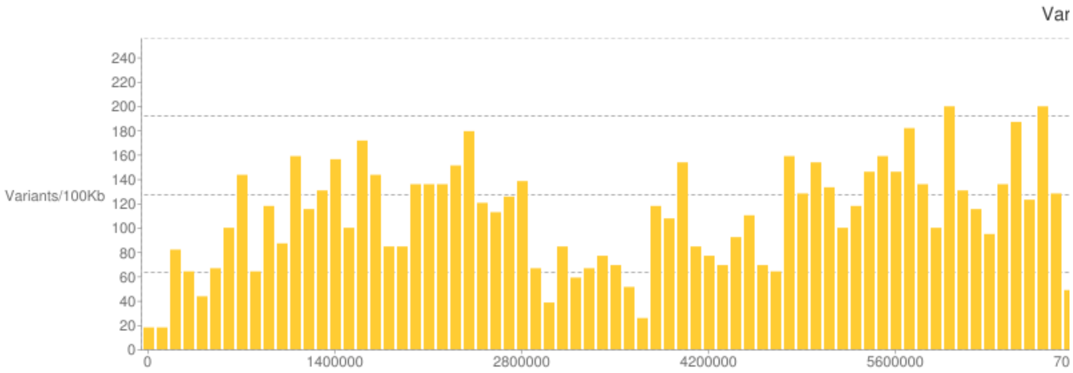

14, Position,0,100000,200000,300000,400000,500000,600000,700000,800000,900000,1000000,1100000,1200000,1300000,1400000,
14,Count,19,18,84,65,44,68,102,144,64,119,89,159,116,133,157,102,173,145,85,85,137,136,137,153,180,122,113,126,140,69,39,86,61,68,77,

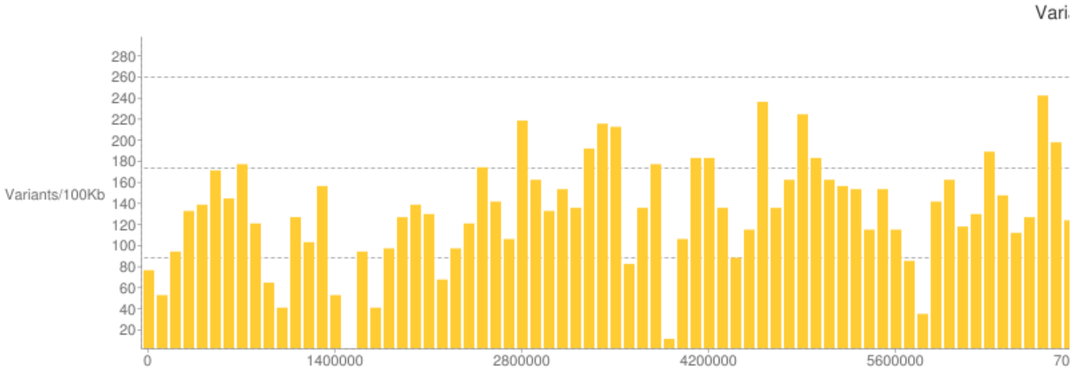

15, Position,0,100000,200000,300000,400000,500000,600000,700000,800000,900000,1000000,1100000,1200000,1300000,1400000,
15,Count,76,54,95,133,141,173,146,179,121,67,41,127,104,158,54,2,94,43,98,128,139,131,69,97,123,174,142,108,220,164,134,154,138,193,2

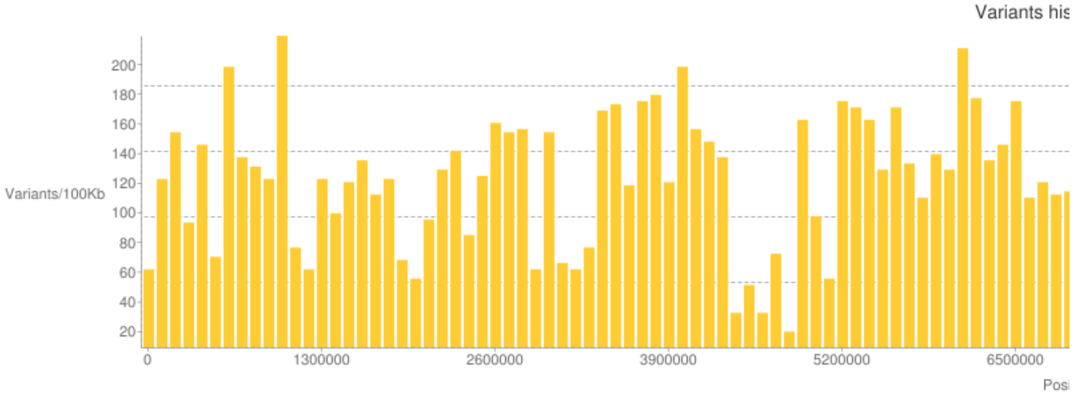

16, Position,0,100000,200000,300000,400000,500000,600000,700000,800000,900000,1000000,1100000,1200000,1300000,1400000,
16,Count,62,123,155,94,147,71,198,138,132,124,219,77,63,123,101,121,136,112,123,69,56,97,130,143,85,125,162,155,157,63,154,66,62,78,

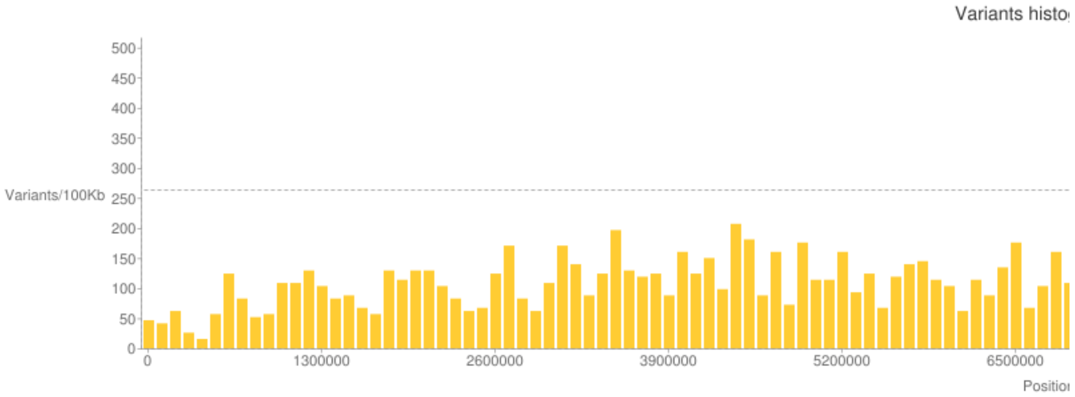

17, Position,0,100000,200000,300000,400000,500000,600000,700000,800000,900000,1000000,1100000,1200000,1300000,1400000,
17,Count,49,45,67,30,20,60,125,84,55,60,111,111,133,104,87,89,71,62,134,114,133,131,107,87,64,69,127,175,83,67,113,173,144,92,127,198,

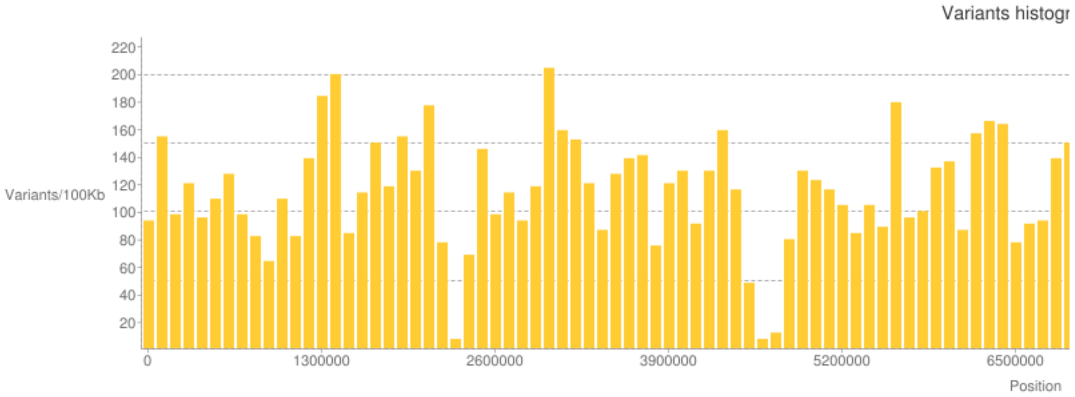

18, Position,0,100000,200000,300000,400000,500000,600000,700000,800000,900000,1000000,1100000,1200000,1300000,1400000,
18,Count,94,155,100,122,97,111,128,99,83,65,111,84,141,185,200,86,115,151,119,155,132,179,78,8,69,146,100,115,95,119,205,161,154,123,

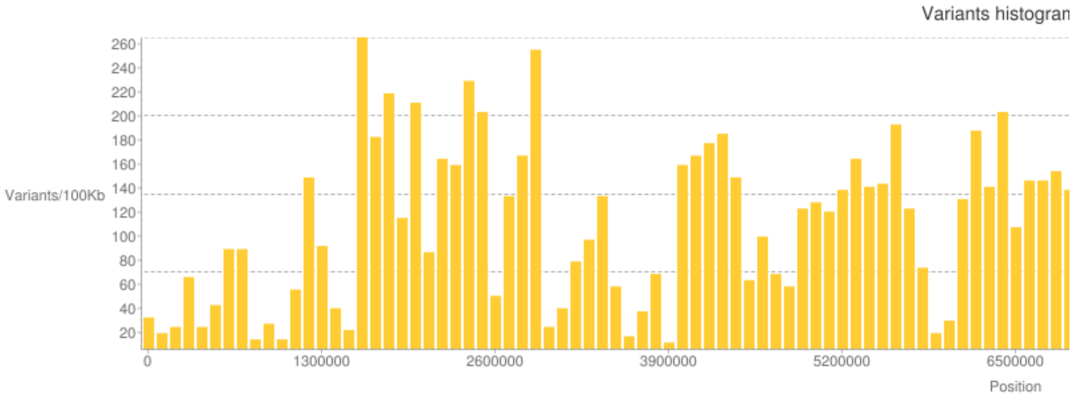

19, Position,0,100000,200000,300000,400000,500000,600000,700000,800000,900000,1000000,1100000,1200000,1300000,1400000,
19,Count,32,21,25,66,25,43,89,90,16,27,15,56,150,93,41,23,265,184,220,115,211,87,165,161,230,203,51,133,169,255,25,40,81,98,134,59,17,

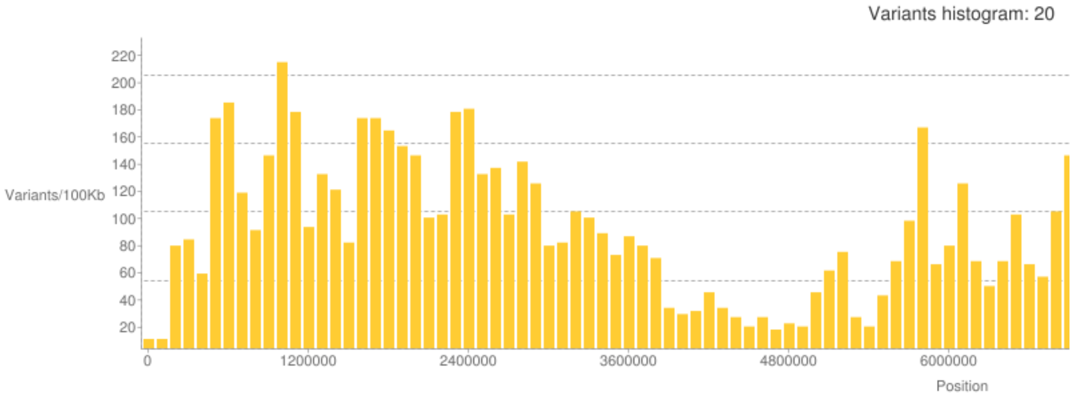

20, Position,0,100000,200000,300000,400000,500000,600000,700000,800000,900000,1000000,1100000,1200000,1300000,1400000,[illegible]
20,Count,11,11,81,85,59,174,187,120,93,148,215,179,94,133,123,83,174,174,166,154,146,102,103,179,181,134,139,104,142,127,80,82,107,10[illegible]

Details by gene

Here you can find a tab-separated table.
