# Supplementary material for: Genome-wide SNP and InDel analysis of three Philippine mango species inferred from whole-genome sequencing
Source: J Genet Eng Biotechnol. 2022 Mar 11;20:46. doi: 10.1186/s43141-022-00326-3 (PMC8917249; doi:10.1186/s43141-022-00326-3)
Supplement: Supplementary file 3 — Additional file 3: Supplemental File 1. A_Alphonso SnpEff. B_Tommy Atkins SnpEff. [file 43141_2022_326_MOESM3_ESM.zip › Supplemental File 1B_Tommy Atkins SnpEffR2.pdf]

SnpEff: Variant analysis

Contents

- Summary
- Variant rate by chromosome
- Variants by type
- Number of variants by impact
- Number of variants by functional class
- Number of variants by effect
- Quality histogram
- InDel length histogram
- Base variant table
- Transition vs transversions (ts/tv)
- Allele frequency
- Allele Count
- Codon change table
- Amino acid change table
- Chromosome variants plots
- Details by gene

Summary

**Genome**	manindi_TA
**Date**	2021-05-22 15:03
**SnpEff version**	`SnpEff 5.0e (build 2021-03-09 06:01), by Pablo Cingolani`
**Command line arguments**	`SnpEff  manindi_TA /home/cocogenomics/mango_genome/mango_TA_genome/M.odorata_TA_SNP_final.vcf`
**Warnings**	280,326
**Errors**	0
**Number of lines (input file)**	3,766,404
**Number of variants (before filter)**	3,777,813
**Number of not variants (i.e. reference equals alternative)**	0
**Number of variants processed (i.e. after filter and non-variants)**	3,777,813
**Number of known variants (i.e. non-empty ID)**	0 (0%)
**Number of multi-allelic VCF entries (i.e. more than two alleles)**	11,409
**Number of effects**	7,191,596
**Genome total length**	377,290,333
**Genome effective length**	377,290,333
**Variant rate**	1 variant every 99 bases

Variants rate details

Chromosome	Length	Variants	Variants rate
1	17,320,008	219,486	78
2	17,063,873	180,956	94
3	21,566,805	199,114	108
4	22,357,487	241,393	92
5	14,540,018	148,603	97
6	10,680,009	95,531	111
7	13,133,232	153,954	85
8	14,750,018	167,749	87
9	21,055,410	215,911	97
10	11,063,414	133,091	83
11	17,675,019	185,635	95
12	14,336,529	146,136	98
13	15,099,493	166,459	90
14	13,335,999	131,074	101
15	16,178,320	160,990	100
16	21,434,198	204,868	104
17	11,746,059	138,795	84
18	16,863,820	206,406	81
19	22,398,858	254,860	87
20	16,105,987	188,608	85
10000001	48,585,777	238,194	203
**Total**	**377,290,333**	**3,777,813**	**99**

Number variants by type

Type	Total
**SNP**	3,777,813
**MNP**	0
**INS**	0
**DEL**	0
**MIXED**	0
**INV**	0
**DUP**	0
**Total**	**3,777,813**

Type	Total
BND	0
INTERVAL	0
Total	3,777,813

Number of effects by impact

Type (alphabetical order)	Count	Percent
HIGH	3,564	0.05%
LOW	105,214	1.463%
MODERATE	111,102	1.545%
MODIFIER	6,971,716	96.943%

Number of effects by functional class

Type (alphabetical order)	Count	Percent
MISSENSE	111,626	55.944%
NONSENSE	1,630	0.817%
SILENT	86,276	43.239%

Missense / Silent ratio: 1.2938

Number of effects by type and region

Type

Type (alphabetical order)	Count	Percent
3_prime_UTR_variant	58,036	0.805%
5_prime_UTR_premature_start_codon_gain_variant	4,850	0.067%
5_prime_UTR_variant	30,836	0.428%
downstream_gene_variant	1,570,171	21.776%
initiator_codon_variant	32	0%
intergenic_region	2,801,150	38.849%
intragenic_variant	104	0.001%
intron_variant	823,307	11.418%
missense_variant	111,102	1.541%
splice_acceptor_variant	731	0.01%
splice_donor_variant	711	0.01%
splice_region_variant	18,521	0.257%
start_lost	183	0.003%
stop_gained	1,630	0.023%
stop_lost	309	0.004%
stop_retained_variant	143	0.002%
synonymous_variant	86,133	1.195%
upstream_gene_variant	1,702,492	23.611%

Region

Type (alphabetical order)	Count	Percent
DOWNSTREAM	1,570,171	21.833%
EXON	197,700	2.749%
INTERGENIC	2,801,150	38.95%
INTRON	808,927	11.248%
SPLICE_SITE_ACCEPTOR	731	0.01%
SPLICE_SITE_DONOR	711	0.01%
SPLICE_SITE_REGION	15,888	0.221%
TRANSCRIPT	104	0.001%
UPSTREAM	1,702,492	23.673%
UTR_3_PRIME	58,036	0.807%
UTR_5_PRIME	35,686	0.496%

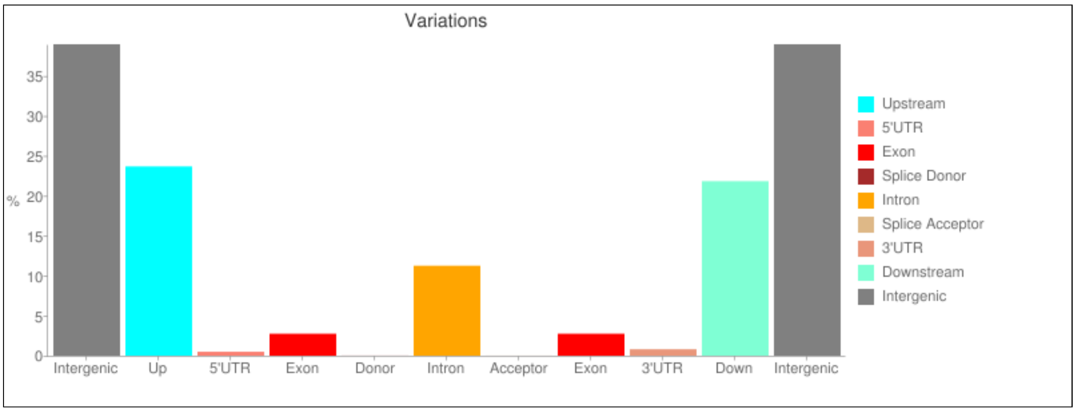

Quality:

**Min**	10
**Max**	21,860
**Mean**	147.722
**Median**	118
**Standard deviation**	197.192
**Values**	10,11,12,13,14,15,16,17,18,19,20,21,22,23,24,25,26,27,28,29,30,31,32,33,34,35,36,37,38,39,40,41,42,43,44,45,46,47,48,49,50,51,52,53,54,55,56,57
**Count**	1455,1884,7633,3094,2255,2170,6121,9191,14697,15344,18198,19449,22292,23733,25533,29871,26220,23947,23990,11568,9648,17653,8239,960

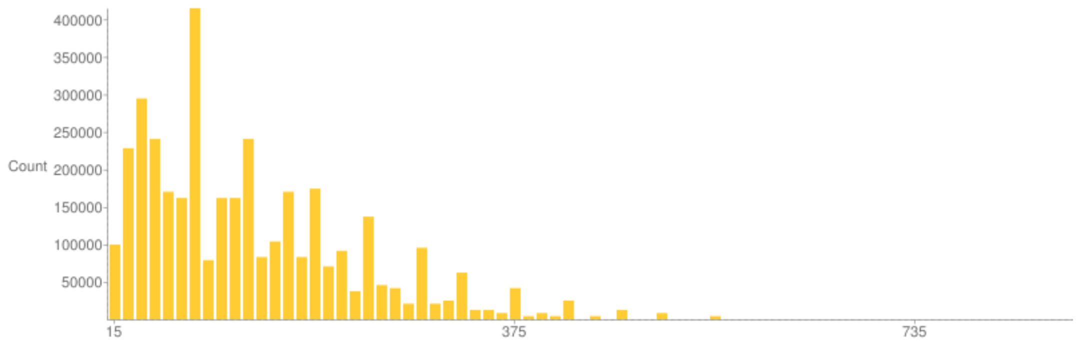

Insertions and deletions length:

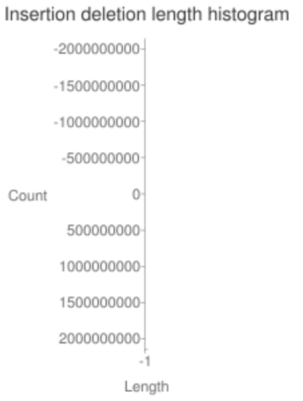

Base changes (SNPs)

	A	C	G	T
A	0	132,294	637,091	192,541
C	140,323	0	90,385	696,814
G	696,625	90,045	0	140,132
T	192,503	637,196	131,864	0

Ts/Tv (transitions / transversions)

Note: Only SNPs are used for this statistic.
Note: This Ts/Tv ratio is a 'raw' ratio (ratio of observed events).

Transitions	3,570,390
Transversions	1,479,013
Ts/Tv ratio	2.414

All variants:

```
Sample ,readname,Total
Transitions ,3570390,3570390
Transversions ,1479013,1479013
Ts/Tv ,2.414,2.414
```

Only known variants (i.e. the ones having a non-empty ID field):

```
No results available (empty input?)
```

Allele frequency

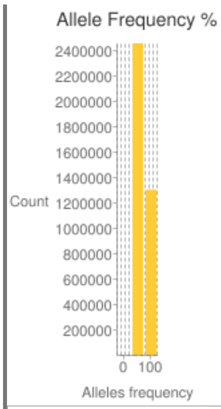

**Min**	0
**Max**	100
**Mean**	67.335
**Median**	50
**Standard deviation**	23.878
**Values**	0,50,100
**Count**	2938,2454711,1308755

Allele Count

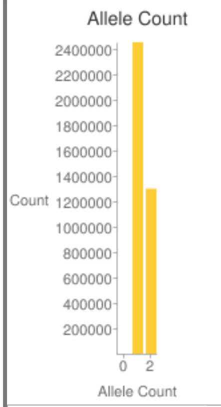

**Min**	0
**Max**	2
**Mean**	1.347
**Median**	1
**Standard deviation**	0.478
**Values**	0,1,2
**Count**	2938,2454711,1308755

Hom/Het per sample

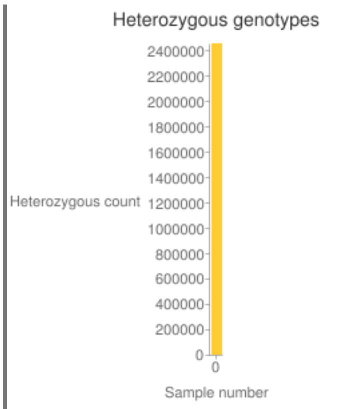

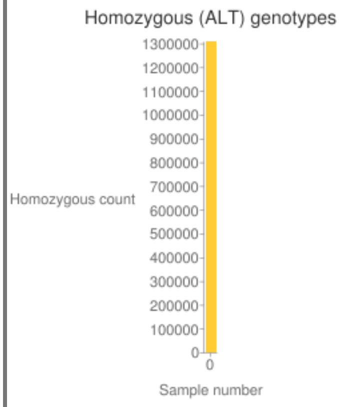

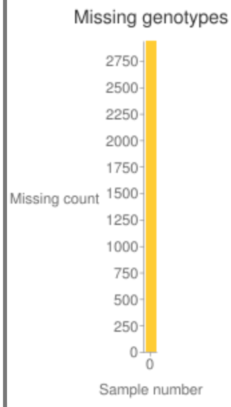


```
Sample_names , readname
Reference , 0
Het , 2454711
Hom , 1308755
Missing , 2938
```

Codon changes

How to read this table:
- Rows are reference codons and columns are changed codons. E.g. Row 'AAA' column 'TAA' indicates how many 'AAA' codons have been replaced by 'TAA' codons.
- Red background colors indicate that more changes happened (heat-map).
- Diagonals are indicated using grey background color
- WARNING: This table may include different translation codon tables (e.g. mamalian DNA and mitochondrial DNA).

	AAA	AAC	AAG	AAT	ACA	ACC	ACG	ACT	AGA	AGC	AGG	AGT	ATA	ATC	ATG	ATT	CAA	CAC	CAG	CAT	CCA
**AAA**		259	1,388	385	275				724				201				360				
**AAC**	293		289	1,471		157				570				100				112			
**AAG**	1,502	266		343			127				740				228				249		
**AAT**	372	1,294	307					281				845				240				274	
**ACA**	271					255	975	477	231				557								132
**ACC**		179			237		165	1,245		202				299							
**ACG**			182		1,212	178		300			113				588						
**ACT**				328	485	1,064	235					370				632					
**AGA**	742				208					106	703	135	147								
**AGC**		590				159			111		126	976		100							
**AGG**			679				80		762	111		187			121						
**AGT**				861				352	120	779	147					222					
**ATA**	173				484				139					285	616	450					
**ATC**		73				228				86			339		209	1,271					
**ATG**			242				550				163		757	203		296					
**ATT**				254				543				200	429	1,123	286						
**CAA**	398																	155	1,095	221	188

	AAA	AAC	AAG	AAT	ACA	ACC	ACG	ACT	AGA	AGC	AGG	AGT	ATA	ATC	ATG	ATT	CAA	CAC	CAG	CAT	CCA
**CAC**		109															158		190	820	
**CAG**			269														1,158	175		187	
**CAT**				282													216	703	160		
**CCA**					144												186				
**CCC**						78												60			243
**CCG**							37												122		1,583
**CCT**								163												127	433
**CGA**									298								800				101
**CGC**										87								381			
**CGG**											203								731		
**CGT**												122								792	
**CTA**													178				142				265
**CTC**														130				61			
**CTG**															245				170		
**CTT**																326				178	
**GAA**	1,070																402				
**GAC**		517																104			
**GAG**			982																355		
**GAT**				1,142																276	
**GCA**					971																228
**GCC**						513															
**GCG**							202														
**GCT**								969													
**GGA**									558												
**GGC**										430											
**GGG**											364										
**GGT**												747									
**GTA**													669								
**GTC**														594							
**GTG**															716						
**GTT**																1,281					
**TAA**	18																39				
**TAC**		114																282			
**TAG**			9																32		
**TAT**				219																569	
**TCA**					219																450
**TCC**						118															
**TCG**							40														
**TCT**								275													
**TGA**									14												
**TGC**										84											
**TGG**											105										
**TGT**												162									
**TTA**													183								
**TTC**														133							
**TTG**															282						
**TTT**																243					

Amino acid changes

How to read this table:
- Rows are reference amino acids and columns are changed amino acids. E.g. Row 'A' column 'E' indicates how many 'A' amino acids have been replaced by 'E' amino acids.
- Red background colors indicate that more changes happened (heat-map).
- Diagonals are indicated using grey background color
- WARNING: This table may include different translation codon tables (e.g. mamalian DNA and mitochondrial DNA).

	*	A	C	D	E	F	G	H	I	K	L	M	N	P	Q	R	S	T	V	W	Y
*	**143**		6		15		13			27	34				71	54	23			35	31
A		**8,218**		310	447		769							586			1,061	2,655	2,451		
C	42		**1,332**			265	164									638	604			138	625
D		279		**3,327**	1,597		858	380					1,659						221		345
E	163	397		1,522	**2,814**		953			2,052					757				323		
F			278			**2,601**			376		1,714						681		390		490
G	46	753	253	976	1,045		**6,193**									1,251	1,177		568	93	
H				332				**1,523**			227		391	172	724	966					848
I						390			**3,897**	173	760	1,111	327			139	286	1,255	2,249		
K	119				1,679				201	**2,890**		228	1,253		609	1,464		402			
L	122					1,924		239	817		**12,620**	527		1,097	312	321	1,088		1,336	116	
M									1,256	242	546					163		550	614		
N				1,400				386	340	1,261			**2,765**				1,415	438			333
P		535						187			1,377			**6,704**	308	305	1,493	422			
Q	344				742			738		667	319			279	**2,253**	1,113					
R	238		869				1,151	1,173	147	1,421	348	121		316	1,531	**4,557**	748	288		428	

	*	A	C	D	E	F	G	H	I	K	L	M	N	P	Q	R	S	T	V	W	Y
**S**	143	923	703			795	1,147		322		1,337		1,451	1,271		662	**9,449**	1,163		82	329
**T**		2,187							1,488	453		588	507	395		344	1,235	**6,828**			
**V**		2,106		199	261	490	470		2,544		1,407	716							**6,180**		
**W**	239		126				77				146					384	53				
**Y**	174		650	313		495		851					333				280				**1,982**

Variants by chromosome

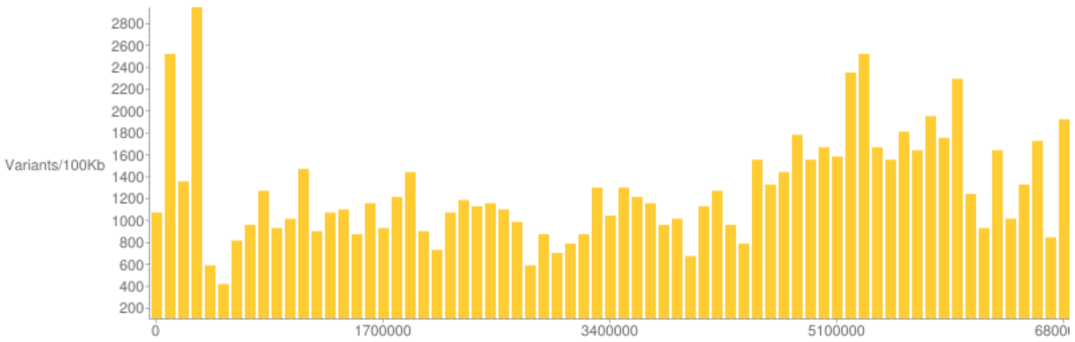

1, Position,0,100000,200000,300000,400000,500000,600000,700000,800000,900000,1000000,1100000,1200000,1300000,1400000,[illegible]
1,Count,1081,2524,1362,2943,585,429,817,956,1295,944,1036,1474,904,1070,1098,872,1155,950,1236,1462,914,734,1083,1194,1139,1160,1115,[illegible]

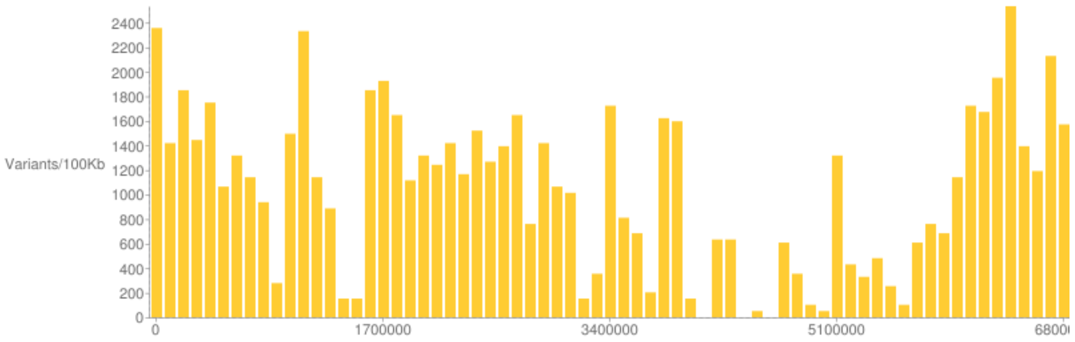

2, Position,0,100000,200000,300000,400000,500000,600000,700000,800000,900000,1000000,1100000,1200000,1300000,1400000,[illegible]
2,Count,2365,1432,1855,1452,1769,1068,1323,1164,947,295,1497,2332,1151,910,175,174,1868,1928,1663,1134,1325,1249,1436,1189,1538,1267,[illegible]

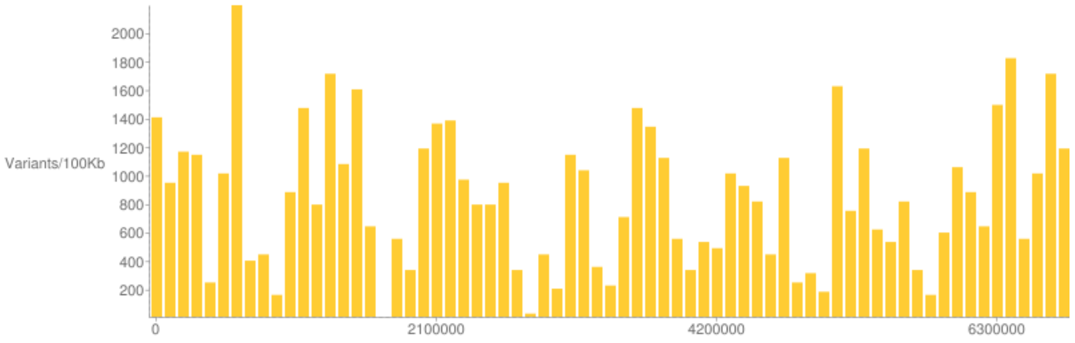

3, Position,0,100000,200000,300000,400000,500000,600000,700000,800000,900000,1000000,1100000,1200000,1300000,1400000,[illegible]
3,Count,1423,959,1181,1153,263,1030,2196,423,458,182,900,1483,815,1725,1097,1608,663,9,561,350,1200,1372,1401,976,800,797,961,347,45,[illegible]

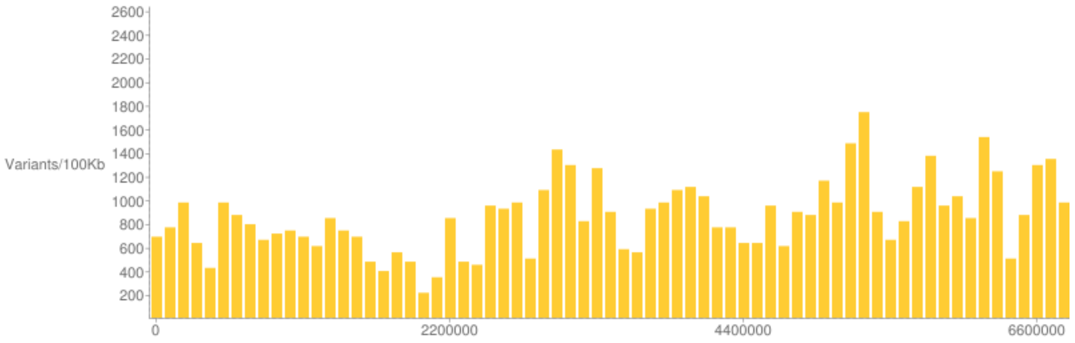

4, Position,0,100000,200000,300000,400000,500000,600000,700000,800000,900000,1000000,1100000,1200000,1300000,1400000,
4,Count,708,779,995,654,448,1007,879,802,691,745,751,698,631,869,746,708,495,412,568,489,236,371,863,501,481,971,945,999,510,1092,143

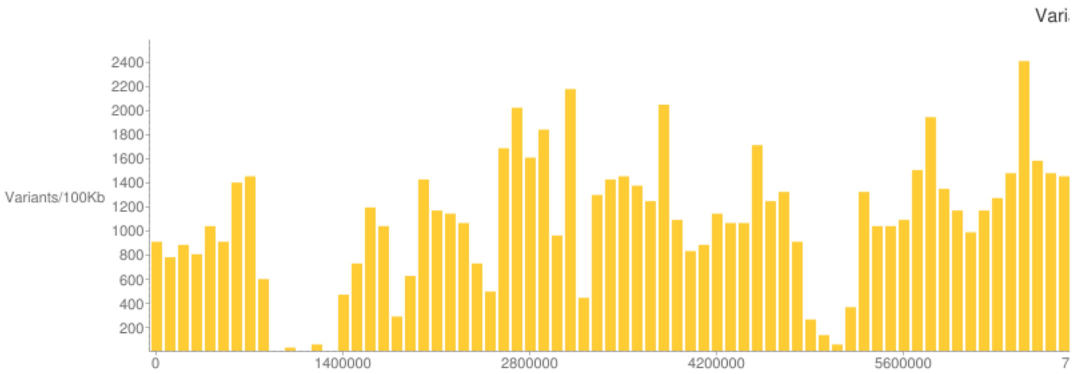

5, Position,0,100000,200000,300000,400000,500000,600000,700000,800000,900000,1000000,1100000,1200000,1300000,1400000,
5,Count,925,793,906,819,1044,929,1417,1455,604,11,45,2,68,13,486,749,1211,1037,309,636,1440,1181,1147,1066,749,511,1699,2041,1608,184

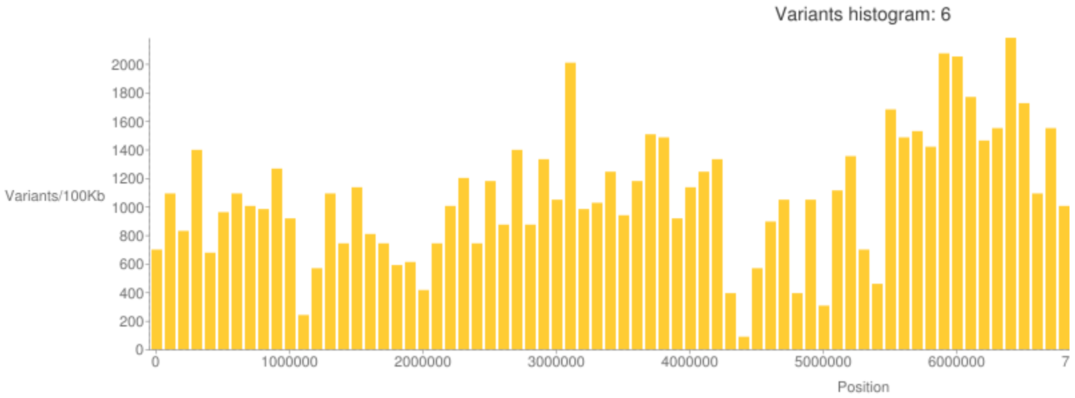

6, Position,0,100000,200000,300000,400000,500000,600000,700000,800000,900000,1000000,1100000,1200000,1300000,1400000,
6,Count,706,1100,835,1411,680,964,1102,1010,993,1267,921,253,582,1097,745,1137,815,761,600,611,431,754,1012,1212,751,1187,881,1408,88

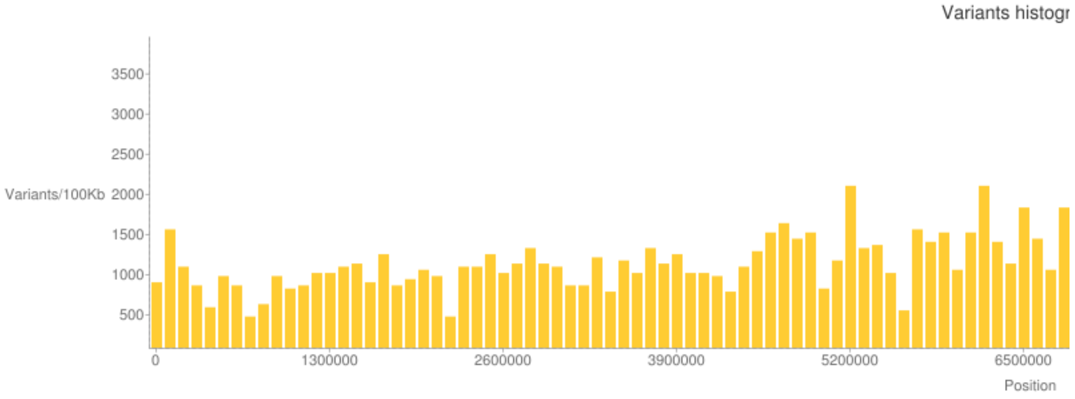

7, Position,0,100000,200000,300000,400000,500000,600000,700000,800000,900000,1000000,1100000,1200000,1300000,1400000,
7,Count,931,1555,1124,886,589,995,870,472,639,993,837,878,1017,1032,1125,1149,931,1264,888,950,1087,1005,469,1101,1109,1277,1031,1132,

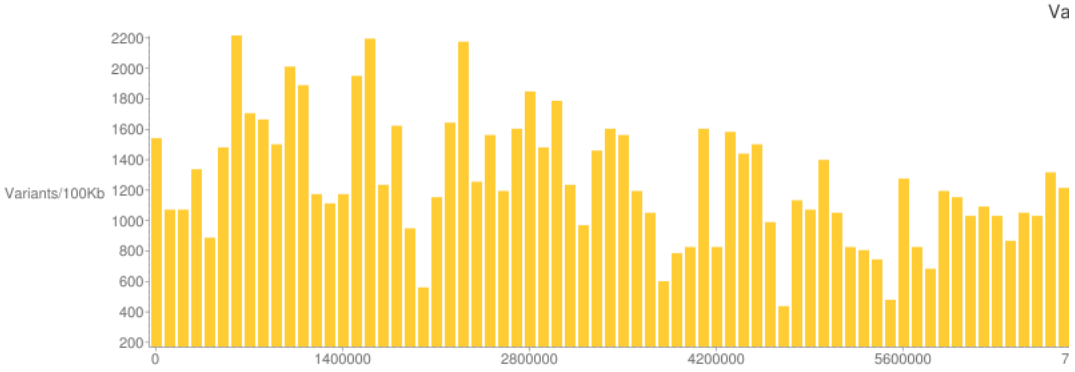

8, Position,0,100000,200000,300000,400000,500000,600000,700000,800000,900000,1000000,1100000,1200000,1300000,1400000,
8,Count,1551,1069,1085,1352,888,1482,2212,1703,1674,1504,2019,1895,1179,1113,1171,1958,2200,1241,1635,965,564,1164,1659,2181,1255,157

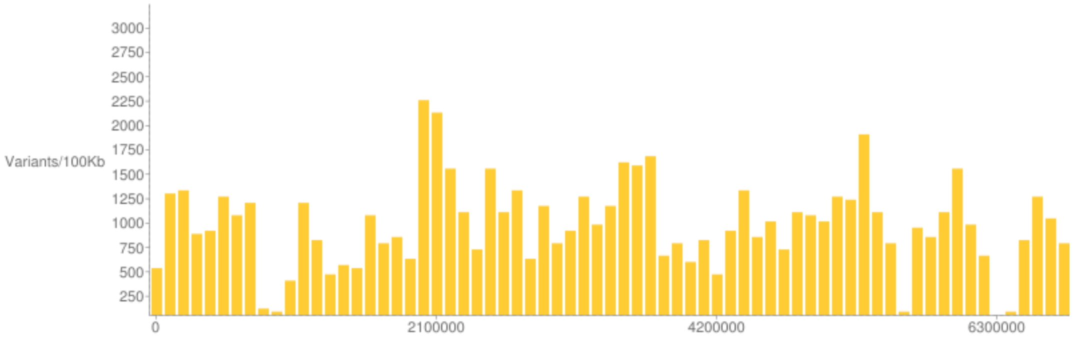

9, Position,0,100000,200000,300000,400000,500000,600000,700000,800000,900000,1000000,1100000,1200000,1300000,1400000,
9,Count,535,1309,1332,886,932,1274,1101,1222,125,113,421,1209,847,498,586,549,1103,799,870,651,2258,2148,1560,1110,753,1575,1114,1346,

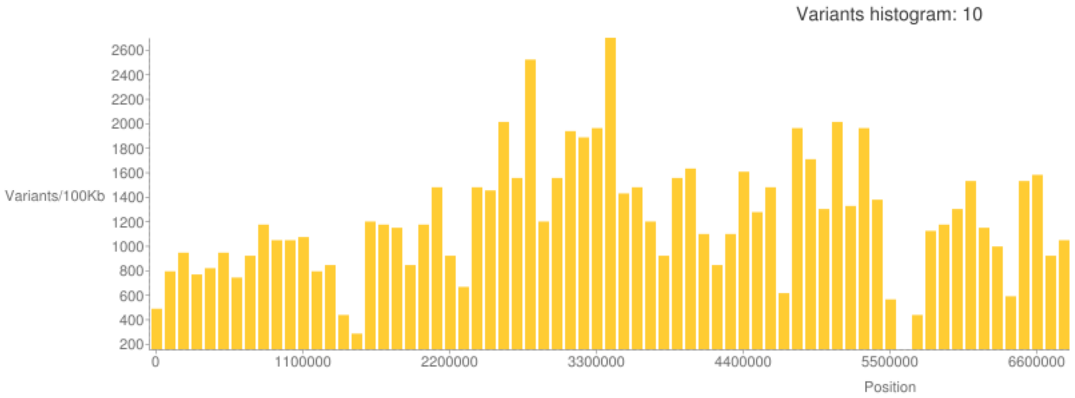

10, Position,0,100000,200000,300000,400000,500000,600000,700000,800000,900000,1000000,1100000,1200000,1300000,1400000,
10,Count,510,810,965,782,833,965,750,922,1193,1060,1054,1083,791,845,436,284,1199,1182,1154,850,1191,1495,934,684,1492,1459,2022,1568,

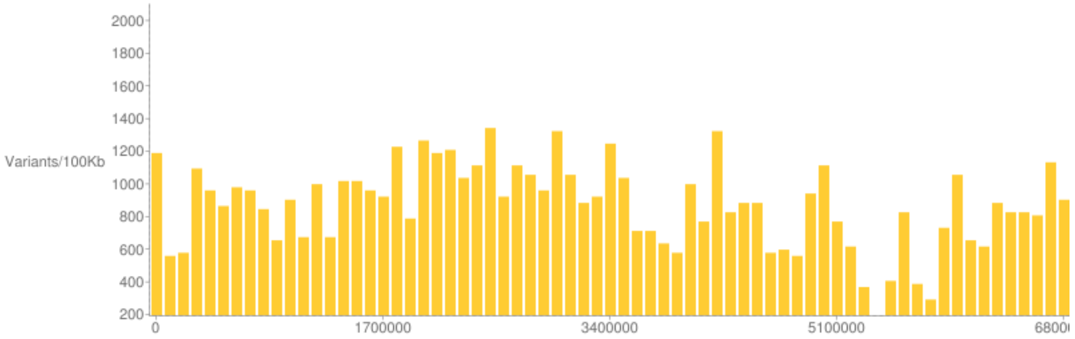

11, Position,0,100000,200000,300000,400000,500000,600000,700000,800000,900000,1000000,1100000,1200000,1300000,1400000,
11,Count,1200,558,588,1104,974,865,981,971,859,657,915,672,1011,682,1017,1031,962,924,1228,792,1262,1204,1217,1038,1111,1339,922,1119,

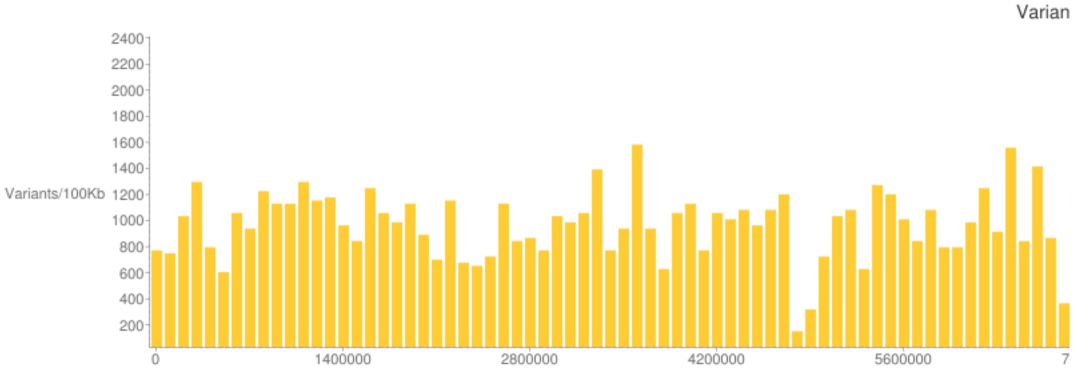

12, Position,0,100000,200000,300000,400000,500000,600000,700000,800000,900000,1000000,1100000,1200000,1300000,1400000,
12,Count,789,757,1048,1315,813,614,1060,954,1242,1141,1133,1309,1171,1175,961,853,1256,1075,988,1135,903,719,1172,680,659,741,1145,84[illegible]

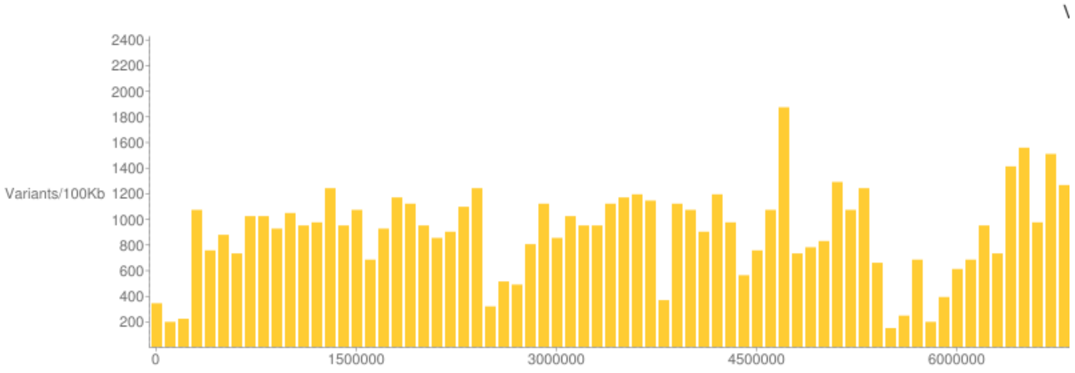

13, Position,0,100000,200000,300000,400000,500000,600000,700000,800000,900000,1000000,1100000,1200000,1300000,1400000,
13,Count,348,197,226,1072,762,884,743,1023,1033,939,1045,955,985,1257,953,1081,696,938,1182,1126,955,851,907,1097,1249,337,520,510,81[illegible]

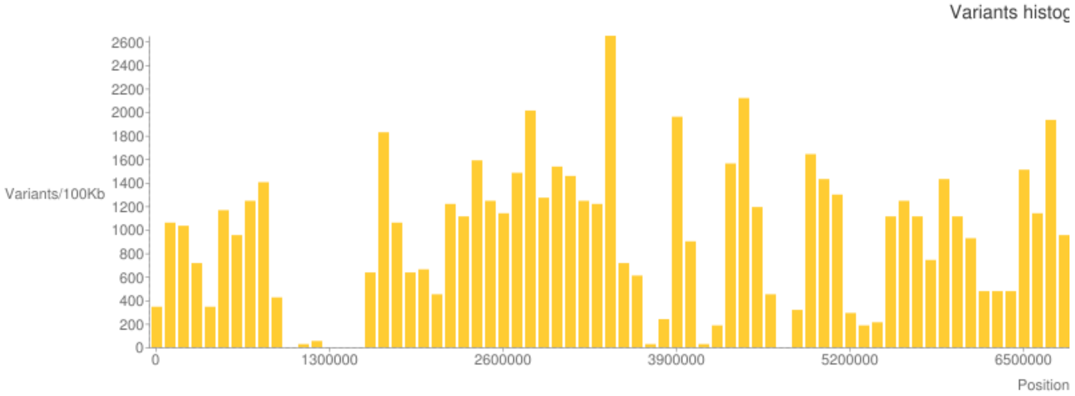

14, Position,0,100000,200000,300000,400000,500000,600000,700000,800000,900000,1000000,1100000,1200000,1300000,1400000,
14,Count,354,1081,1058,721,354,1189,963,1270,1414,441,8,48,65,18,19,0,658,1836,1085,661,662,467,1241,1121,1598,1247,1160,1506,2020,12[illegible]

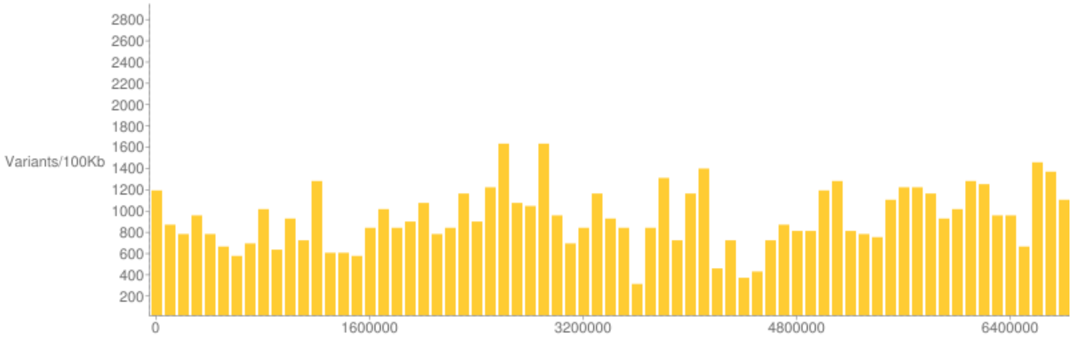

15, Position,0,100000,200000,300000,400000,500000,600000,700000,800000,900000,1000000,1100000,1200000,1300000,1400000,
15,Count,1214,889,784,960,800,685,597,693,1012,654,930,729,1282,606,622,588,836,1020,854,920,1085,798,844,1169,897,1224,1650,1088,106[illegible]

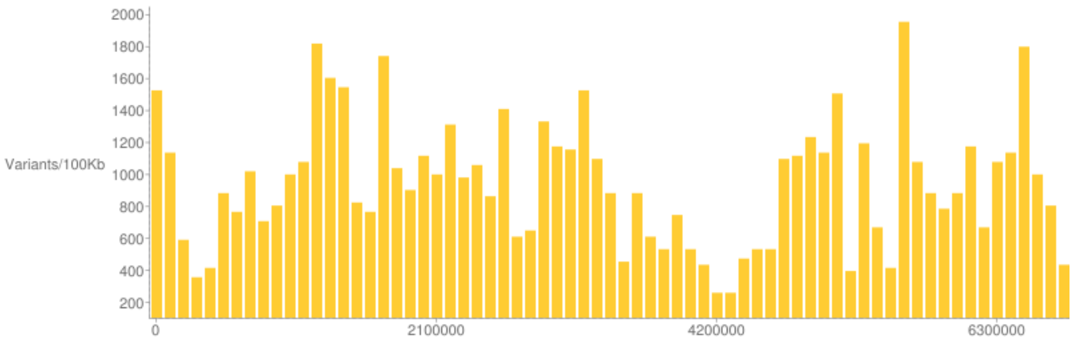

16, Position,0,100000,200000,300000,400000,500000,600000,700000,800000,900000,1000000,1100000,1200000,1300000,1400000,
16,Count,1525,1141,602,367,425,899,777,1031,716,819,1011,1075,1829,1617,1550,825,780,1737,1038,907,1130,1001,1319,978,1069,879,1408,6

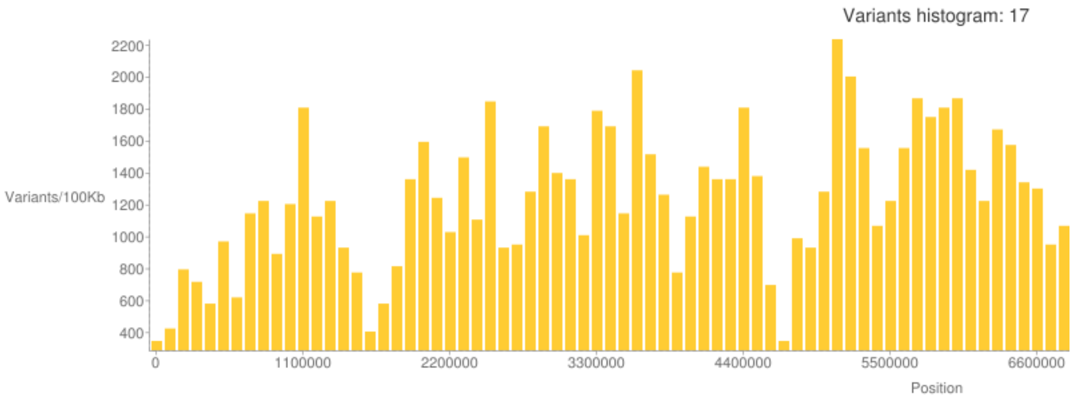

17, Position,0,100000,200000,300000,400000,500000,600000,700000,800000,900000,1000000,1100000,1200000,1300000,1400000,
17,Count,359,424,799,723,580,968,621,1151,1239,899,1220,1818,1130,1226,933,783,411,583,818,1358,1607,1249,1040,1511,1110,1848,929,967,

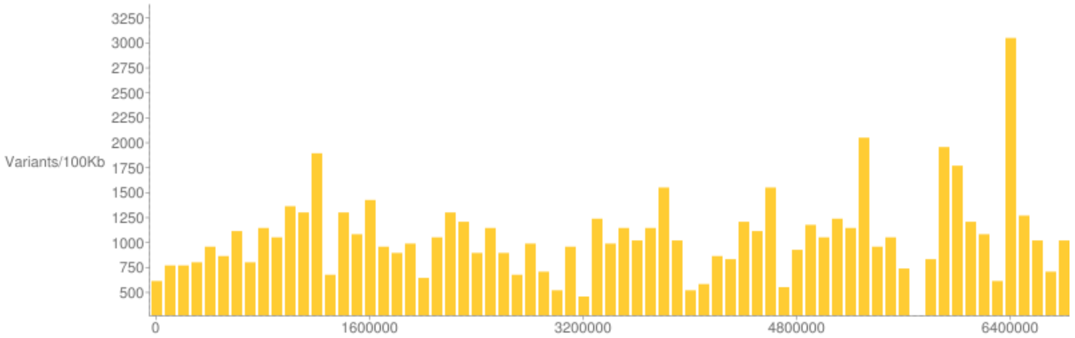

18, Position,0,100000,200000,300000,400000,500000,600000,700000,800000,900000,1000000,1100000,1200000,1300000,1400000,
18,Count,615,779,783,819,953,871,1111,815,1163,1074,1358,1298,1911,692,1314,1104,1430,961,920,1000,645,1046,1296,1216,912,1141,891,70

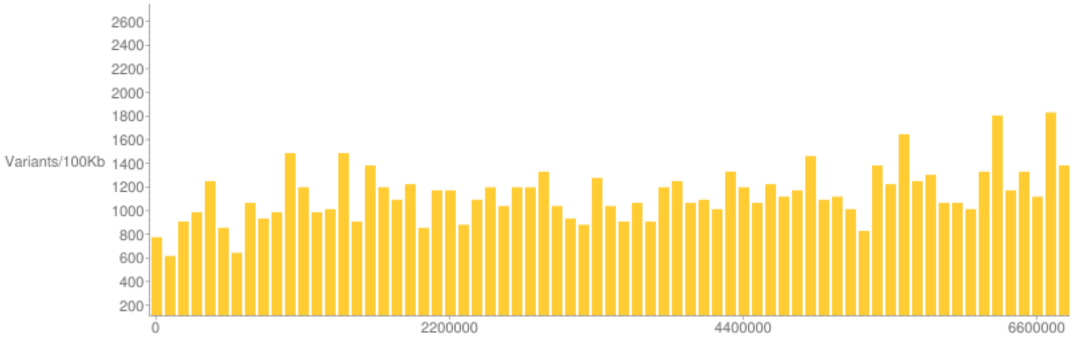

19, Position,0,100000,200000,300000,400000,500000,600000,700000,800000,900000,1000000,1100000,1200000,1300000,1400000,
19,Count,777,625,915,987,1259,850,643,1068,950,1004,1492,1210,1001,1024,1486,910,1379,1209,1087,1243,875,1170,1186,889,1093,1217,1046,

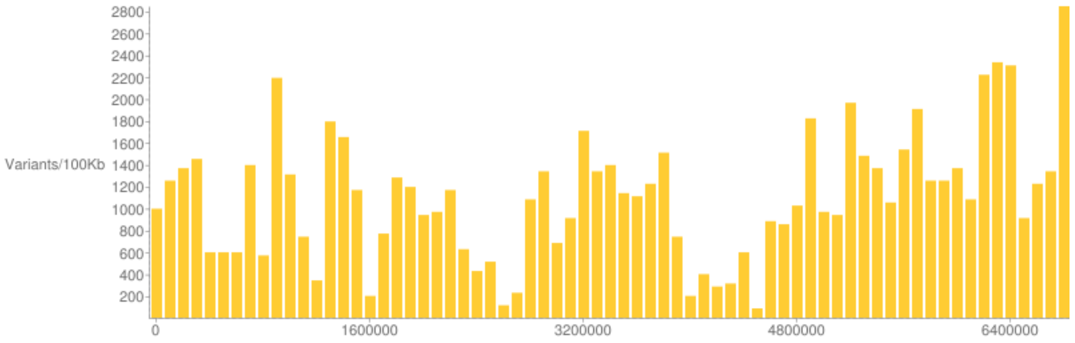

20, Position,0,100000,200000,300000,400000,500000,600000,700000,800000,900000,1000000,1100000,1200000,1300000,1400000,
20,Count,1018,1261,1370,1473,606,607,604,1411,595,2207,1329,765,360,1810,1666,1180,219,773,1295,1226,943,998,1183,652,431,529,122,247,

Variants histogram: 10000001

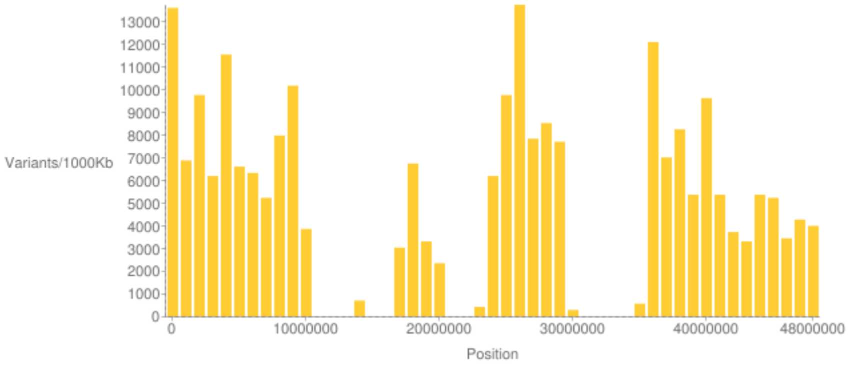

10000001, Position,0,1000000,2000000,3000000,4000000,5000000,6000000,7000000,8000000,9000000,10000000,11000000,120000[illegible]
10000001,Count,13664,6925,9856,6294,11599,6692,6427,5255,8091,10168,3917,0,0,0,719,0,0,3042,6764,3297,2361,0,0,498,6213,9807,13722,78[illegible]

Details by gene

[Here](#) you can find a tab-separated table.

SnpEff: Variant analysis

Contents

Summary
Variant rate by chromosome
Variants by type
Number of variants by impact
Number of variants by functional class
Number of variants by effect
Quality histogram
InDel length histogram
Base variant table
Transition vs transversions (ts/tv)
Allele frequency
Allele Count
Codon change table
Amino acid change table
Chromosome variants plots
Details by gene

Summary

**Genome**	manindi_TA
**Date**	2021-05-22 15:20
**SnpEff version**	`SnpEff 5.0e (build 2021-03-09 06:01), by Pablo Cingolani`
**Command line arguments**	`SnpEff manindi_TA /home/cocogenomics/mango_genome/mango_TA_genome/M.odorata_TA_INDEL_final.vcf`
**Warnings**	50,471
**Errors**	0
**Number of lines (input file)**	497,200
**Number of variants (before filter)**	499,474
**Number of not variants (i.e. reference equals alternative)**	0
**Number of variants processed (i.e. after filter and non-variants)**	499,474
**Number of known variants (i.e. non-empty ID)**	0 (0%)
**Number of multi-allelic VCF entries (i.e. more than two alleles)**	2,274
**Number of effects**	1,038,342
**Genome total length**	377,290,333
**Genome effective length**	377,290,333
**Variant rate**	1 variant every 755 bases

Variants rate details

Chromosome	Length	Variants	Variants rate
1	17,320,008	29,717	582
2	17,063,873	25,866	659
3	21,566,805	27,844	774
4	22,357,487	32,325	691
5	14,540,018	19,936	729
6	10,680,009	12,325	866
7	13,133,232	20,257	648
8	14,750,018	23,203	635
9	21,055,410	28,283	744
10	11,063,414	16,916	654
11	17,675,019	23,544	750
12	14,336,529	19,499	735
13	15,099,493	22,307	676
14	13,335,999	17,992	741
15	16,178,320	21,019	769
16	21,434,198	27,286	785
17	11,746,059	16,824	698
18	16,863,820	27,686	609
19	22,398,858	33,261	673
20	16,105,987	23,292	691
10000001	48,585,777	30,092	1,614
**Total**	**377,290,333**	**499,474**	**755**

Number variants by type

Type	Total
**SNP**	0
**MNP**	0
**INS**	242,643
**DEL**	256,831
**MIXED**	0
**INV**	0
**DUP**	0
**Total**	**499,474**

Type	Total
BND	0
INTERVAL	0
Total	499,474

Number of effects by impact

Type (alphabetical order)	Count	Percent
HIGH	5,812	0.56%
LOW	2,281	0.22%
MODERATE	3,011	0.29%
MODIFIER	1,027,238	98.931%

Number of effects by functional class

Type (alphabetical order)	Count	Percent

Missense / Silent ratio: 0

Number of effects by type and region

Type

Type (alphabetical order)	Count	Percent
3_prime_UTR_variant	10,397	0.998%
5_prime_UTR_truncation	2	0%
5_prime_UTR_variant	6,417	0.616%
bidirectional_gene_fusion	5	0%
conservative_inframe_deletion	587	0.056%
conservative_inframe_insertion	664	0.064%
disruptive_inframe_deletion	1,114	0.107%
disruptive_inframe_insertion	743	0.071%
downstream_gene_variant	244,707	23.486%
exon_loss_variant	5	0%
frameshift_variant	5,307	0.509%
intergenic_region	369,574	35.47%
intragenic_variant	21	0.002%
intron_variant	123,064	11.811%
non_coding_transcript_variant	267	0.026%
splice_acceptor_variant	229	0.022%
splice_donor_variant	287	0.028%
splice_region_variant	2,815	0.27%
start_lost	104	0.01%
start_retained_variant	9	0.001%
stop_gained	198	0.019%
stop_lost	75	0.007%
stop_retained_variant	11	0.001%
upstream_gene_variant	275,331	26.425%

Region

Type (alphabetical order)	Count	Percent
DOWNSTREAM	244,697	23.566%
EXON	8,408	0.81%
GENE	5	0%
INTERGENIC	369,574	35.593%
INTRON	120,611	11.616%
SPLICE_SITE_ACCEPTOR	194	0.019%
SPLICE_SITE_DONOR	234	0.023%
SPLICE_SITE_REGION	2,281	0.22%
TRANSCRIPT	288	0.028%
UPSTREAM	275,331	26.516%
UTR_3_PRIME	10,331	0.995%
UTR_5_PRIME	6,388	0.615%

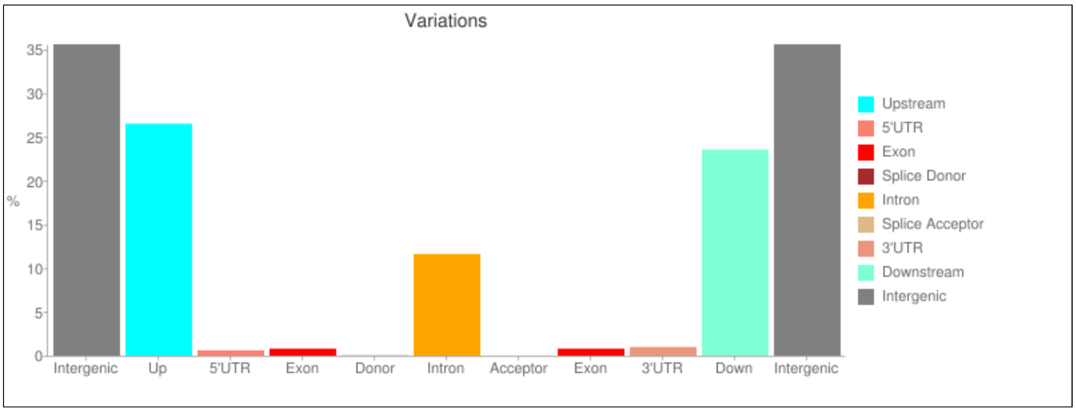

Quality:

Min	10
Max	24,711
Mean	130.314
Median	98
Standard deviation	178.204
Values	10,11,12,13,14,15,16,17,18,19,20,21,22,23,24,25,26,27,28,29,30,31,32,33,34,35,36,37,38,39,40,41,42,43,44,45,46,47,48,49,50,51,52,53,54,55,56,57
Count	813,1075,1654,1183,1277,1201,1358,1391,1403,1501,1371,1353,2650,1367,1346,3205,1358,1332,4524,1309,1451,6866,1838,1615,9418,1337,141[illegible]

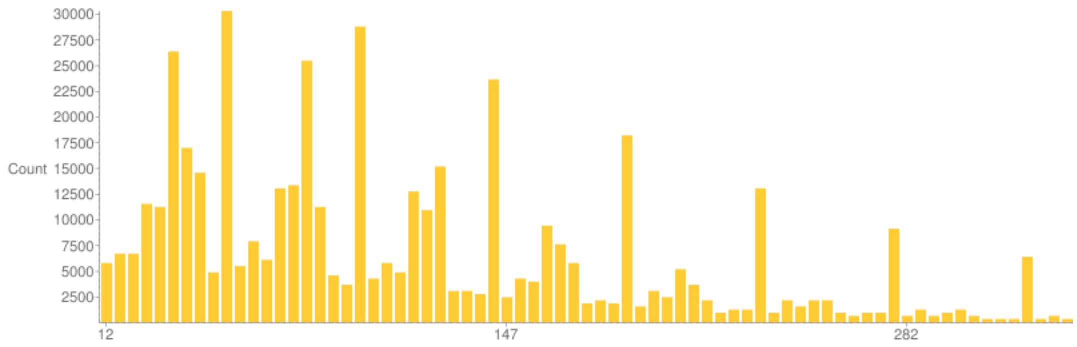

Insertions and deletions length:

**Min**	0
**Max**	258
**Mean**	2.194
**Median**	1
**Standard deviation**	5.837
**Values**	0,1,2,3,4,5,6,7,8,9,10,11,12,13,14,15,16,17,18,19,20,21,22,23,24,25,26,27,28,29,30,31,32,33,34,35,36,37,38,39,40,41,42,43,44,45,46,47,48,49,50,51
**Count**	115292,299992,18609,12720,7099,5811,3856,3727,3369,3235,2632,2727,1996,1677,1475,1308,1094,1058,870,856,844,677,577,550,519,446,478,4

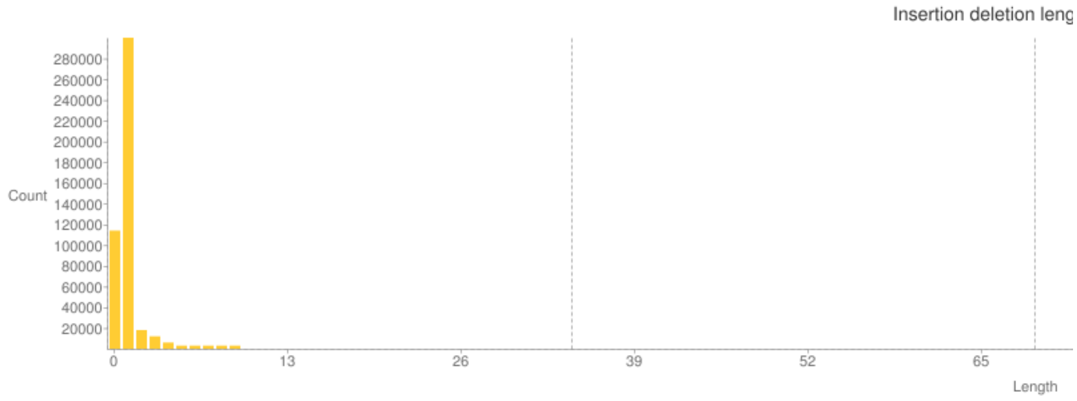

Base changes (SNPs)

	A	C	G	T
**A**	0	0	0	0
**C**	0	0	0	0
**G**	0	0	0	0
**T**	0	0	0	0

Ts/Tv (transitions / transversions)

Note: Only SNPs are used for this statistic.
Note: This Ts/Tv ratio is a 'raw' ratio (ratio of observed events).

**Transitions**	0
**Transversions**	0
**Ts/Tv ratio**	0

All variants:

```
No results available (empty input?)
```

Only known variants (i.e. the ones having a non-empty ID field):

```
No results available (empty input?)
```

Allele frequency

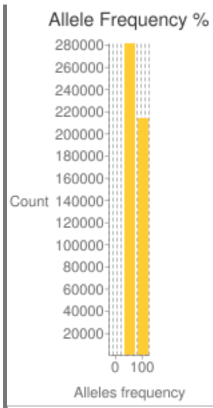

**Min**	0
**Max**	100
**Mean**	71.662
**Median**	50
**Standard deviation**	24.83
**Values**	0,50,100
**Count**	265,281264,215671

Allele Count

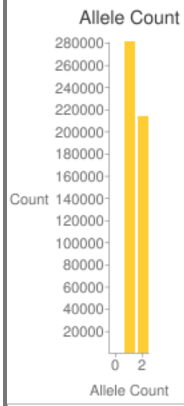

**Min**	0
**Max**	2
**Mean**	1.433
**Median**	1
**Standard deviation**	0.497
**Values**	0,1,2
**Count**	265,281264,215671

Hom/Het per sample

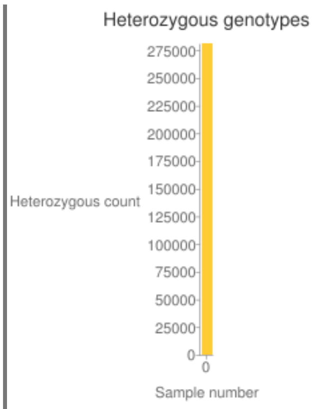

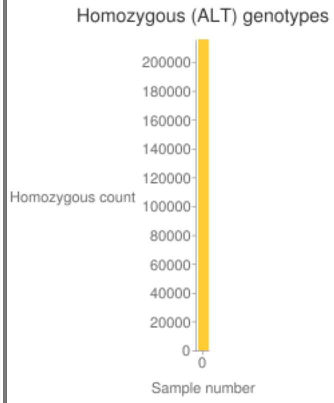

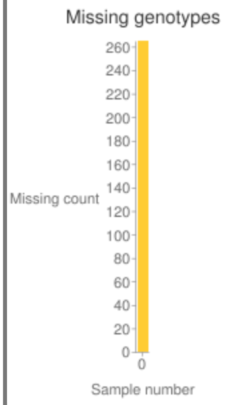


```
Sample_names , readname
Reference , 0
Het , 281264
Hom , 215671
Missing , 265
```

Codon changes

How to read this table:
- Rows are reference codons and columns are changed codons. E.g. Row 'AAA' column 'TAA' indicates how many 'AAA' codons have been replaced by 'TAA' codons.
- Red background colors indicate that more changes happened (heat-map).
- Diagonals are indicated using grey background color
- WARNING: This table may include different translation codon tables (e.g. mamalian DNA and mitochondrial DNA).

	-	AAA	AAC	AAG	AAT	ACA	ACC	ACG	ACT	AGA	AGC	AGG	AGT	ATA	ATC	ATG	ATT	CAA	CAC	CAG	CAT	CCA	CC[illegible]
**-**		194	104	149	171	100	55	45	117	95	64	54	89	65	62	112	123	158	77	100	127	163	5
**AAA**	263	**33**	6	37	21	6	2		3	9	3	3	3	9	1		2	6	1	2	1	1	
**AAC**	182	26	**7**	15	23	4	3	1	7	3	5	3	3	2		3	1	2	1	1			
**AAG**	312	39	11	**24**	10	5	2	1	2	7	1	3	5	2		5	5	2			1		
**AAT**	252	40	16	12	**14**	5	2		9	7	2	1	2	4	5	4	4	7			2	2	
**ACA**	144	5	11	4	4	**21**	9	7	14	3	5	1	1	11		2	4	2					
**ACC**	100	1	3	3		11	**2**	4	8		4	1		2	4	1	1		2	1			
**ACG**	53		2			9	7	**3**	7	1	1	2		3		2							
**ACT**	168	4	12	4	1	11	13	11	**11**	4	3	2	5	1	1	1	4		3	5		1	
**AGA**	191	3	1	14	1	5	1	1	1	**8**	5	10	5			4	3	1			1		
**AGC**	86	1	5	8	2	2	3	1		8	**6**	5	4	2		1							
**AGG**	122	7	3	9	4	1	3			20	1	**3**	6	2		2	1						
**AGT**	171	5	2	7	2	4	3		8	6	4	10	**10**	1	1	1	4	1					
**ATA**	117	6		3	9	7	1	2	2	2	1	3	4	**14**	5	7	19			1	2		
**ATC**	93	2	3	5	9	4	2						3	3	**2**	2	5						
**ATG**	236	7	3	4	34	3	2	2	7	6	2	4	4	11	4	**15**	19	1		1	3		
**ATT**	189	2	1	2	13	1	5	1	8	6		1	3	12	6	10	**13**						

	-	AAA	AAC	AAG	AAT	ACA	ACC	ACG	ACT	AGA	AGC	AGG	AGT	ATA	ATC	ATG	ATT	CAA	CAC	CAG	CAT	CCA	CC(C)
**CAA**	226		3	1	1	3					1	1		1			2	**18**	8	20	17	6	
**CAC**	88	1				3											1	7	**2**	7	19	5	
**CAG**	184				1	1	1			2								28	3	**7**	8	1	
**CAT**	164		2		3					2			2	1		1		13	10	11	**24**	2	
**CCA**	188		1	2			1											6	6	4	2	**10**	1
**CCC**	95				1													2				7	**9**
**CCG**	82			1		2	1											1	1	2	1	9	
**CCT**	192		1				1			2						1		4		5	6	18	2
**CGA**	38				2	1				2								3	1			2	
**CGC**	42							1											3		1		
**CGG**	32			1											1			1		1			
**CGT**	58							1					1	2				1	1		4	1	
**CTA**	84				1	1								3				1		2	2	3	
**CTC**	100					1	1								2			1	1	1	2		
**CTG**	88		1						3		2		1					2		1	2	1	
**CTT**	185	1				3			2			1	1		1			4	3	3	6	1	
**GAA**	372	1		1	3	1				4			3		1					1		2	
**GAC**	160		3	5						1									1				
**GAG**	289	1		4			1	2		6	2		2		1	3					1	1	
**GAT**	353	1	1		4	2				1			1			4	2		2	4	2	2	
**GCA**	218	1	2	1	2	1			1		2	1	2		1			3		1	1		
**GCC**	111		2	2			1				1					2	1				1		
**GCG**	69			2						1													
**GCT**	230	2			1	1			3	1	3		1	1		1		1		1	1		
**GGA**	257					1			1		1	5					1						
**GGC**	137			1	1					1	2	4				1							
**GGG**	132	1	1								1				1			1				1	
**GGT**	212			1	1	3									1							1	
**GTA**	93									1		1						1	1				
**GTC**	72			1		1			1				3			1							
**GTG**	123					2		1			1	1	1		1		2			1			
**GTT**	199	1		1	1		1			2	2		4	1							3		
**TAA**	23												1										
**TAC**	101	1	1	1	2	2							2	1	1				1		1		
**TAG**	15																						
**TAT**	160	3				1	1			1		1		4			1				1		
**TCA**	192		1								1		1	1	4					1	1		
**TCC**	104				2												1			1	2		
**TCG**	53																						
**TCT**	292	3	3		3	1	2		3						4		2			2		1	
**TGA**	22					1																	
**TGC**	64	1				1				2			1			3	1		1		1	1	
**TGG**	86				2							1				2	2						
**TGT**	102	2			6					3	1										1		
**TTA**	147	1	1	2	2				2						1		2	1		1	1		
**TTC**	148				1	1				2	2				1		3	1			1		
**TTG**	202			2							1		1	2			6	1					
**TTT**	267			2		1	2		1				2				4	2		1	2		

Amino acid changes

How to read this table:
- Rows are reference amino acids and columns are changed amino acids. E.g. Row 'A' column 'E' indicates how many 'A' amino acids have been replaced by 'E' amino acids.
- Red background colors indicate that more changes happened (heat-map).
- Diagonals are indicated using grey background color
- WARNING: This table may include different translation codon tables (e.g. mamalian DNA and mitochondrial DNA).

	*	-	?	A	C	D	E	F	G	H	I	K	L	M	N	P	Q	R	S	T	V	W	Y
*****	**13**	59	1			1	1	1					5						5	1	1		9
**-**	173		2,388	290	128	224	342	247	308	204	250	343	463	112	275	366	258	233	628	317	284	63	131
**?**																							
**A**	8	628		**137**	9	25	17	5	41	3	3	8	16	3	7	4	6	5	21	7	33		3
**C**	5	166		2	**22**		1	12	2	3	1	3	17	3	6	2		6	18	1	5	8	6
**D**	10	513		25	1	**53**	100	1	46	5	2	6	9	4	8	4	4	4	12	2	14	1	6
**E**	7	661		21	4	66	**125**	3	53	1	2	7	6	3	3	3	1	14	8	4	18	5	5
**F**	13	415		4	5	3	1	**82**	4	3	8	2	37		1	1	4	4	36	5	6	5	8
**G**	2	738		17	4	29	51	4	**183**		3	3	4	1	3	9	1	13	16	5	31	10	3
**H**	3	251	1	2		3	4	1	5	**55**	2	1	20	1	5	18	38	7	7	3	4		7
**I**	4	399		1	9	6	2	4	7	2	**79**	20	2	19	35	2	1	13	15	33	4	4	5
**K**	17	575		6	2	4	15	3	5	3	19	**133**	10	5	48	1	10	24	17	21	5		1
**L**	12	806		11	18	2	5	74	11	17	17	6	**156**		5	30	18	8	28	13	9	2	23
**M**	5	236		1		2	3		2	3	34	11	4	**15**	37		2	10	10	14	1	3	6
**N**	7	434		6	1	1	6	2	4	3	16	93	7	7	**60**	2	10	15	17	31	2	1	

	*	-	?	A	C	D	E	F	G	H	I	K	L	M	N	P	Q	R	S	T	V	W	Y
**P**	5	557		10	7	3	1	5		16		3	35	1	3	**171**	24	10	15	5	3	5	2
**Q**	3	410		6	1	6	6	4	5	36	3	1	26		5	18	**73**	17	4	5	2		2
**R**	7	483		6	2		9	3	11	11	9	34	24	6	11	11	7	**84**	25	15	3	1	3
**S**	20	898		10	14	3	6	68	11	3	20	24	28	2	20	7	5	32	**272**	27	11	6	17
**T**	3	465		4	4	7	8	4	4	5	31	21	9	6	33	3	8	15	25	**148**		1	3
**V**	5	487		22	6	28	27	5	42	4	4	3	8	1	1	1	2	8	21	6	**125**	1	2
**W**	7	86			11	2	1	2	2		2		15	2	2			2	14			**8**	1
**Y**	23	261			10		4	8	3	3	7	5	25		3	3		4	27	4	3	1	**45**

Variants by chromosome

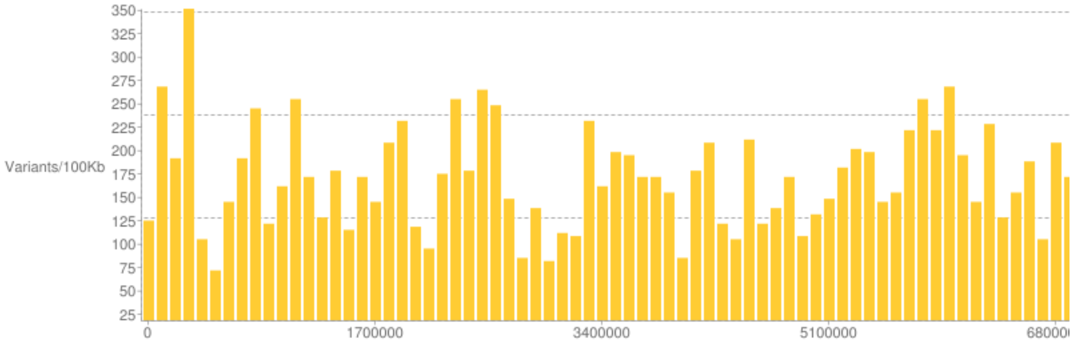


```
1, Position,0,100000,200000,300000,400000,500000,600000,700000,800000,900000,1000000,1100000,1200000,1300000,1400000,
1,Count,127,271,194,351,105,74,147,192,247,123,162,257,173,131,178,116,172,146,208,232,118,96,175,255,179,265,249,149,87,138,83,112,
```

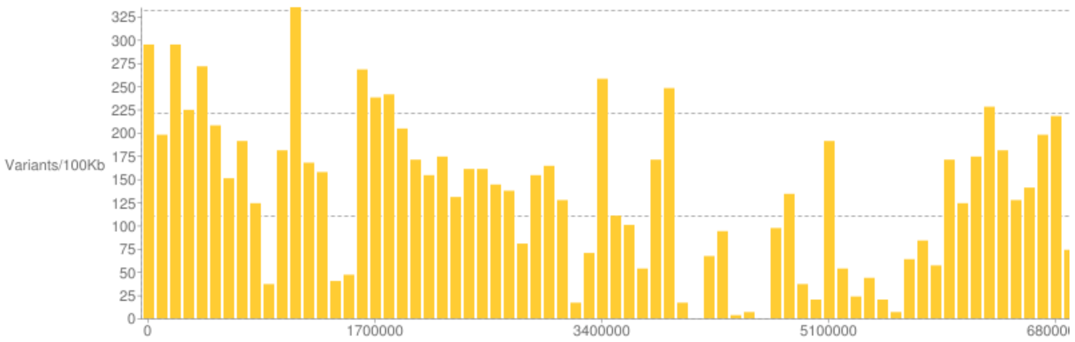


```
2, Position,0,100000,200000,300000,400000,500000,600000,700000,800000,900000,1000000,1100000,1200000,1300000,1400000,
2,Count,298,200,295,226,274,208,152,194,124,40,181,335,168,159,43,49,268,241,244,206,174,156,175,131,163,164,145,138,82,155,166,129,
```

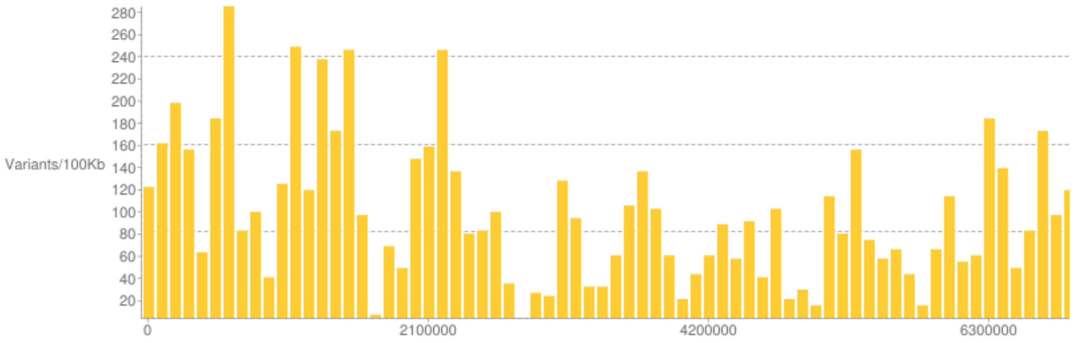


```
3, Position,0,100000,200000,300000,400000,500000,600000,700000,800000,900000,1000000,1100000,1200000,1300000,1400000,
3,Count,124,162,199,158,65,186,285,85,100,41,125,250,120,239,175,248,99,7,69,50,148,159,247,138,80,83,102,35,5,28,26,129,96,34,34,62,
```

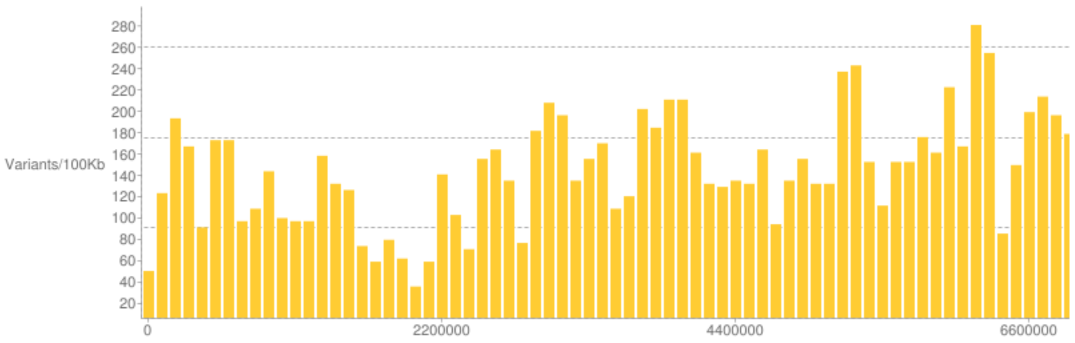

4, Position,0,100000,200000,300000,400000,500000,600000,700000,800000,900000,1000000,1100000,1200000,1300000,1400000,

4,Count,52,125,195,168,91,173,175,97,111,146,102,99,99,159,133,127,74,60,80,62,36,60,143,104,71,156,165,137,77,184,209,198,136,155,17

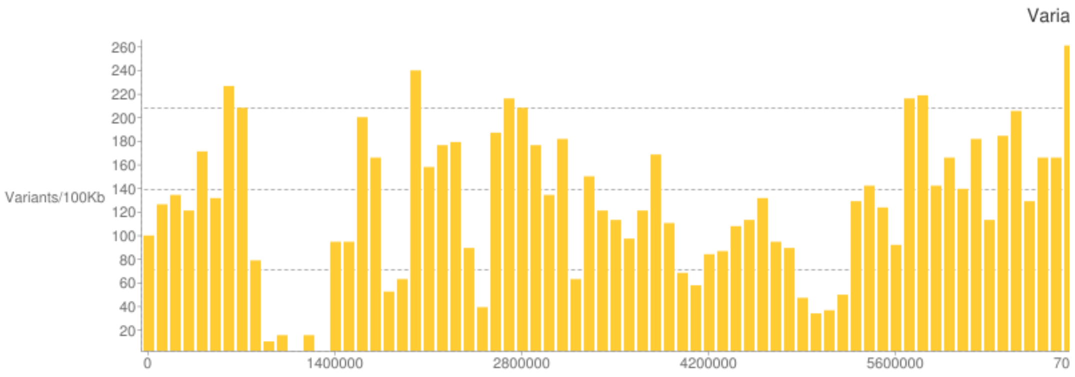

5, Position,0,100000,200000,300000,400000,500000,600000,700000,800000,900000,1000000,1100000,1200000,1300000,1400000,

5,Count,102,128,135,123,172,132,228,210,81,11,17,4,17,2,97,97,200,167,53,65,241,160,177,180,90,41,188,216,209,177,136,183,65,152,122,

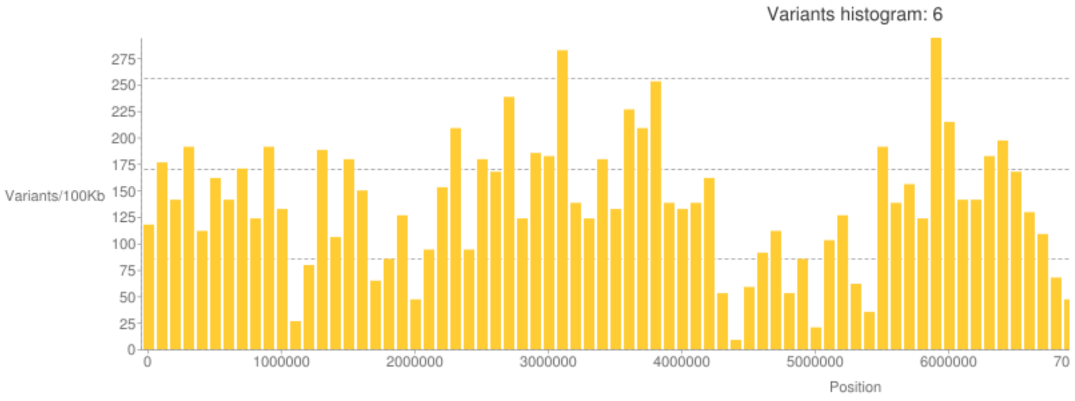

6, Position,0,100000,200000,300000,400000,500000,600000,700000,800000,900000,1000000,1100000,1200000,1300000,1400000,

6,Count,120,178,144,192,113,163,144,171,124,194,135,29,81,190,107,180,151,65,86,127,48,95,153,210,96,180,168,241,125,186,184,284,140,

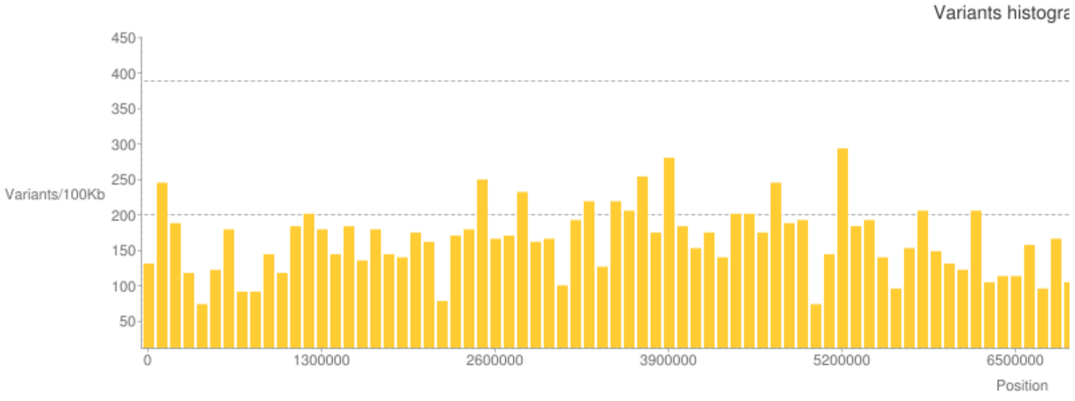

7, Position,0,100000,200000,300000,400000,500000,600000,700000,800000,900000,1000000,1100000,1200000,1300000,1400000,

7,Count,131,248,189,121,77,126,180,93,94,144,120,185,202,180,144,184,138,183,145,143,177,163,78,174,183,252,169,172,235,165,168,104,1

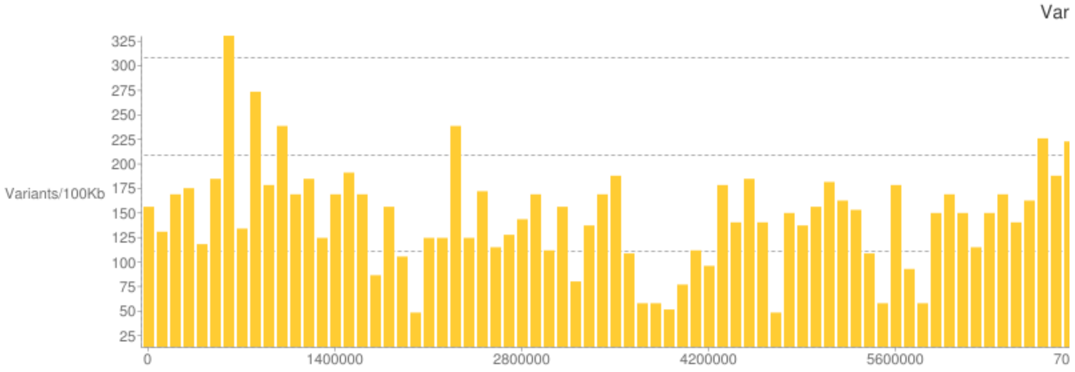

8, Position,0,100000,200000,300000,400000,500000,600000,700000,800000,900000,1000000,1100000,1200000,1300000,1400000,
8,Count,157,133,170,176,120,187,330,134,273,178,241,170,185,126,171,191,171,88,156,108,48,124,125,240,125,172,117,128,143,169,113,158,

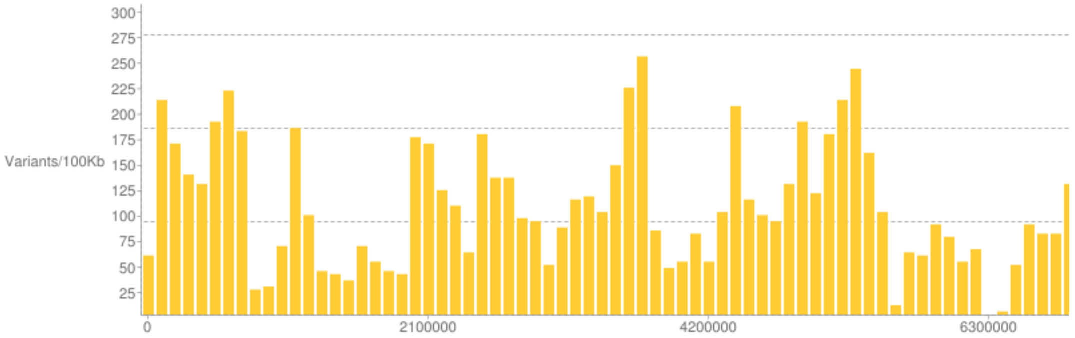

9, Position,0,100000,200000,300000,400000,500000,600000,700000,800000,900000,1000000,1100000,1200000,1300000,1400000,
9,Count,61,215,173,142,133,195,225,185,29,31,72,189,103,47,44,37,71,55,48,45,178,171,128,111,65,181,139,139,98,96,52,91,116,120,106,1

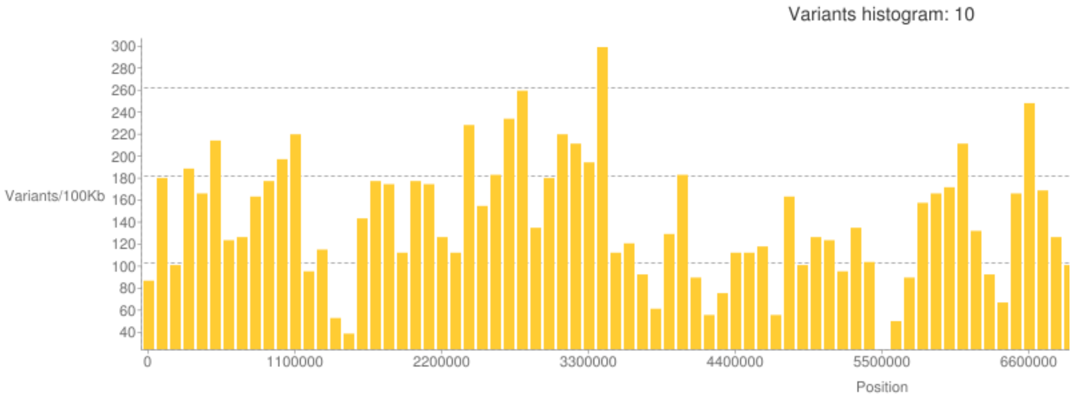

10, Position,0,100000,200000,300000,400000,500000,600000,700000,800000,900000,1000000,1100000,1200000,1300000,1400000,
10,Count,88,181,103,190,168,215,124,126,164,177,197,220,96,116,53,39,145,177,174,113,179,175,127,113,229,157,184,236,260,137,182,220,

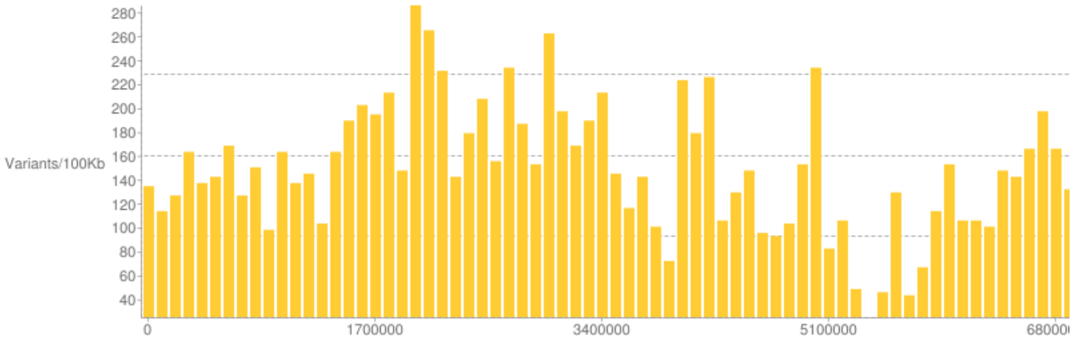

11, Position,0,100000,200000,300000,400000,500000,600000,700000,800000,900000,1000000,1100000,1200000,1300000,1400000,
11,Count,135,115,128,165,139,145,170,129,151,100,165,138,146,105,165,191,205,196,215,149,286,266,232,144,181,210,158,236,188,153,264,

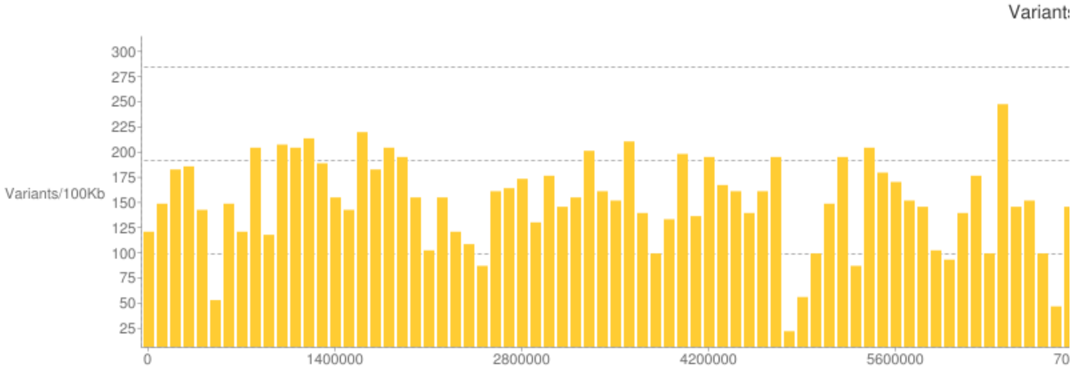


```
              12, Position,0,100000,200000,300000,400000,500000,600000,700000,800000,900000,1000000,1100000,1200000,1300000,1400000,
12,Count,121,150,185,188,144,53,150,123,205,120,207,204,216,189,157,145,221,183,204,197,156,102,156,121,108,89,163,164,173,130,177,14
```

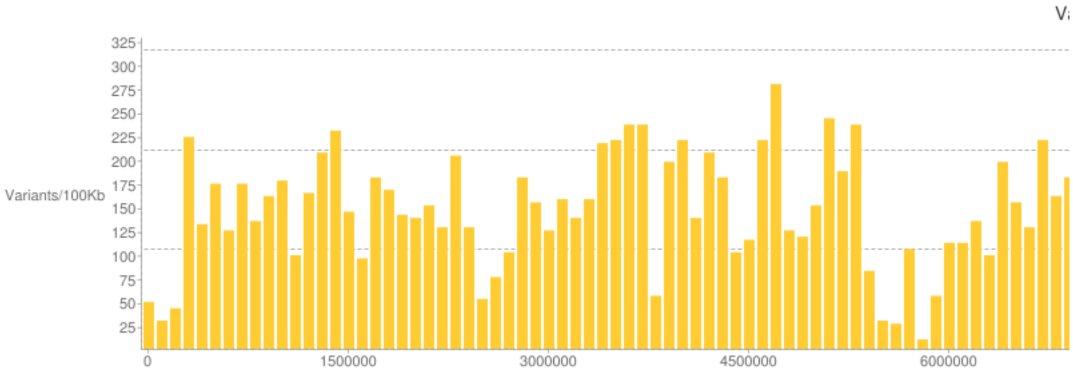


```
              13, Position,0,100000,200000,300000,400000,500000,600000,700000,800000,900000,1000000,1100000,1200000,1300000,1400000,
13,Count,53,33,46,228,136,178,129,176,138,165,182,102,167,210,232,147,99,183,170,144,141,153,130,208,132,57,79,105,185,159,127,162,14
```

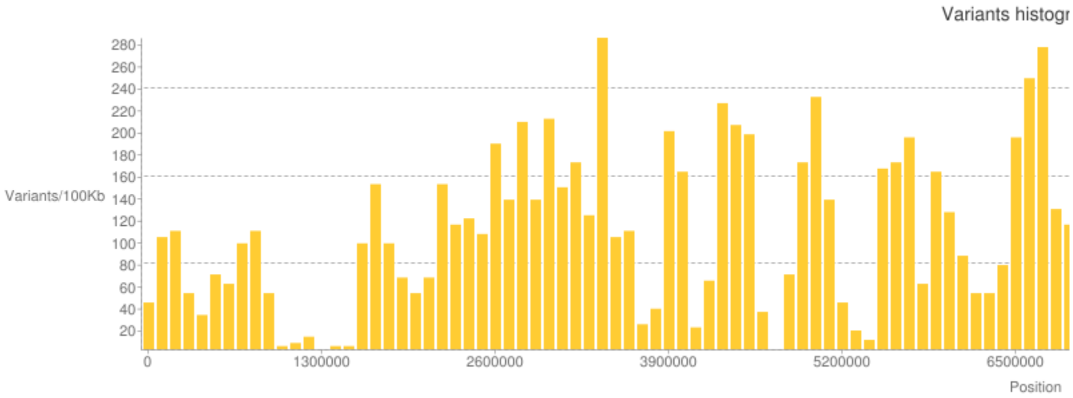


```
              14, Position,0,100000,200000,300000,400000,500000,600000,700000,800000,900000,1000000,1100000,1200000,1300000,1400000,
14,Count,47,105,112,54,35,72,65,102,113,54,6,10,17,4,6,7,102,153,101,70,56,69,154,117,122,110,192,139,211,141,213,151,173,127,286,106,
```

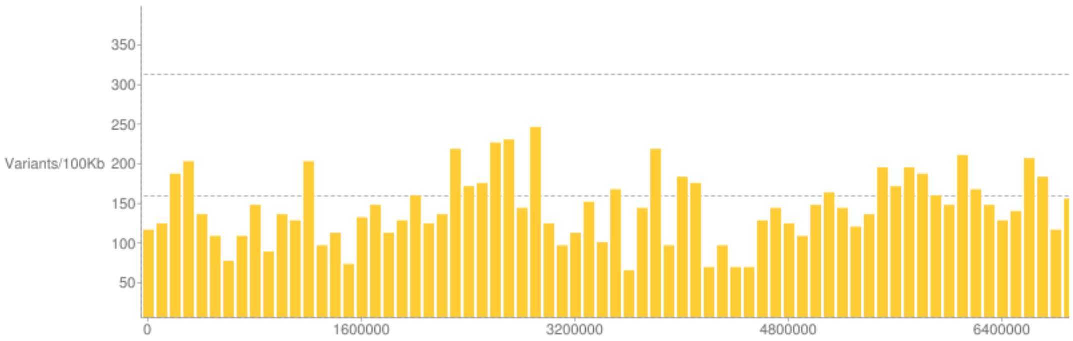


```
              15, Position,0,100000,200000,300000,400000,500000,600000,700000,800000,900000,1000000,1100000,1200000,1300000,1400000,
15,Count,118,125,189,206,139,110,78,111,150,91,139,130,205,100,113,76,134,148,115,128,162,127,138,222,173,176,227,231,144,248,124,98,
```

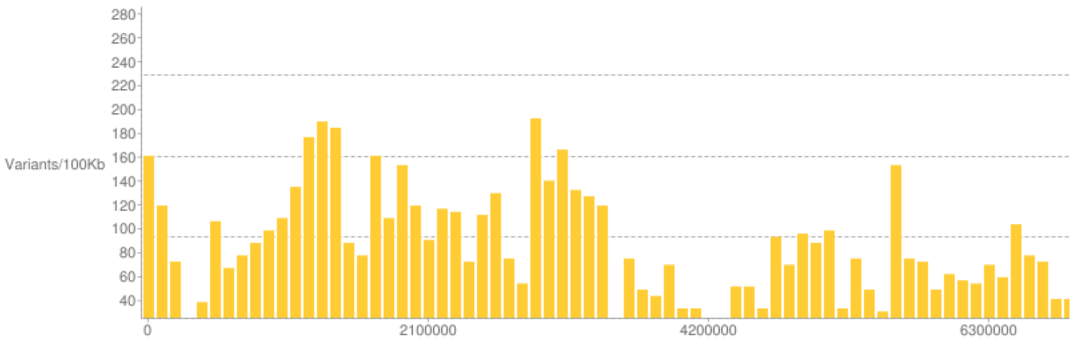

16, Position,0,100000,200000,300000,400000,500000,600000,700000,800000,900000,1000000,1100000,1200000,1300000,1400000,
16,Count,162,121,73,27,39,107,67,78,90,100,111,135,177,192,186,89,79,163,111,154,119,91,117,116,73,112,131,77,55,194,142,168,134,128,

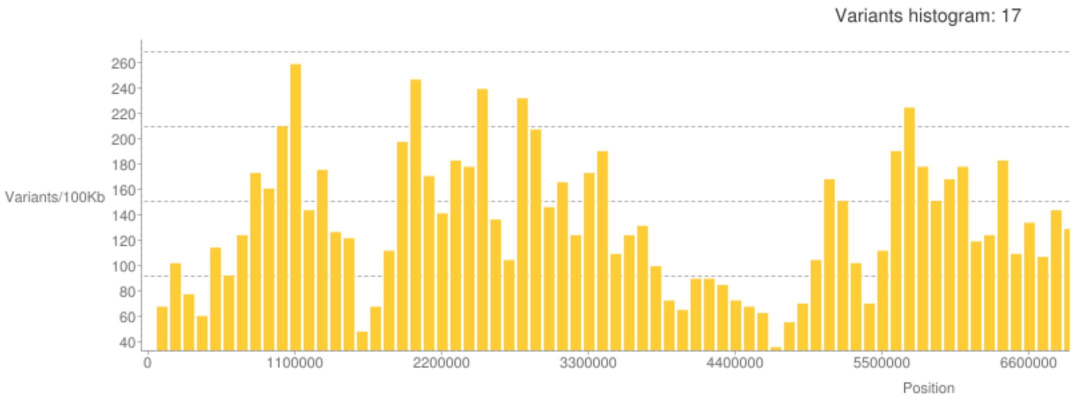

17, Position,0,100000,200000,300000,400000,500000,600000,700000,800000,900000,1000000,1100000,1200000,1300000,1400000,
17,Count,33,69,104,78,62,114,92,125,173,161,211,260,145,176,128,122,48,68,113,199,247,171,141,184,179,241,137,106,232,207,146,167,124,

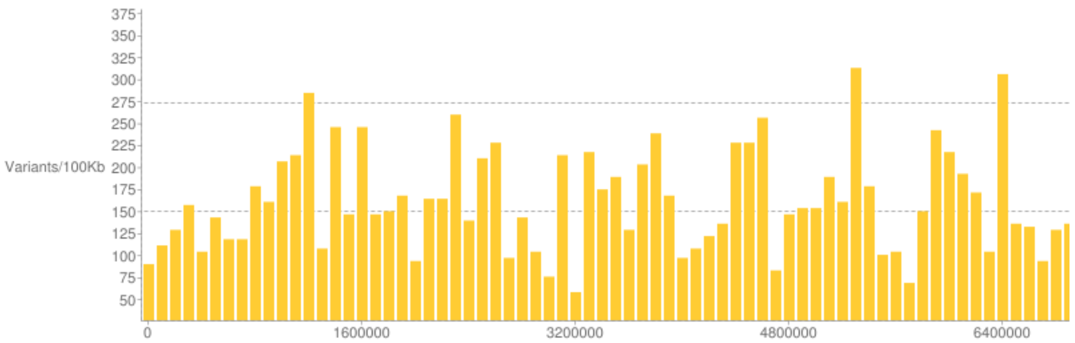

18, Position,0,100000,200000,300000,400000,500000,600000,700000,800000,900000,1000000,1100000,1200000,1300000,1400000,
18,Count,91,113,131,157,104,145,121,121,179,163,208,217,286,109,247,147,247,147,153,169,95,167,167,260,140,211,230,99,143,105,77,214,

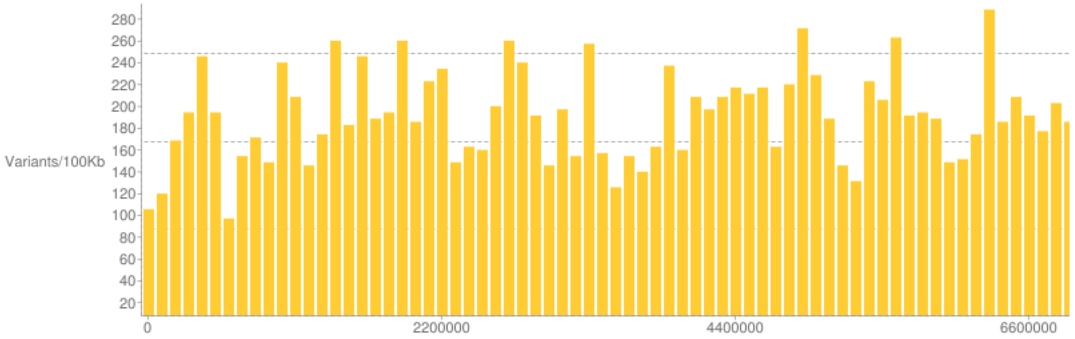

19, Position,0,100000,200000,300000,400000,500000,600000,700000,800000,900000,1000000,1100000,1200000,1300000,1400000,
19,Count,108,120,170,194,246,194,98,156,173,149,241,209,146,174,260,185,246,190,194,260,186,223,234,150,165,161,202,262,242,192,148,

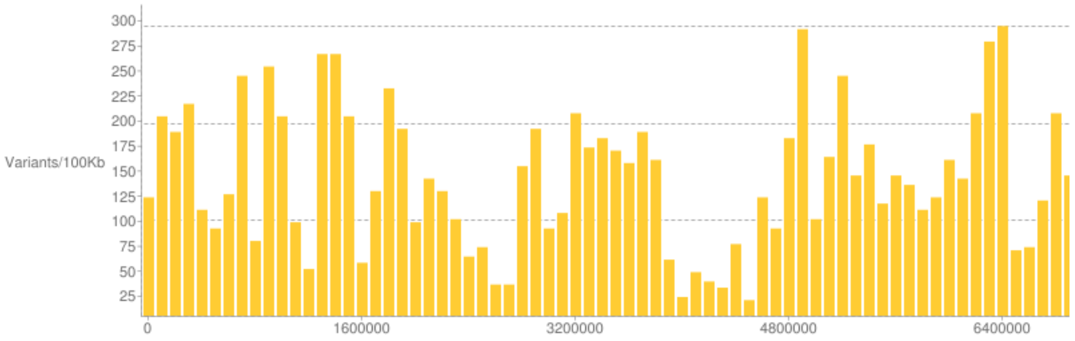

20, Position,0,100000,200000,300000,400000,500000,600000,700000,800000,900000,1000000,1100000,1200000,1300000,1400000,
20,Count,124,206,191,217,113,94,129,247,81,256,205,100,52,268,267,206,60,131,233,194,101,142,130,102,67,75,39,39,157,194,95,110,210,1[illegible]

Variants histogram: 10000001

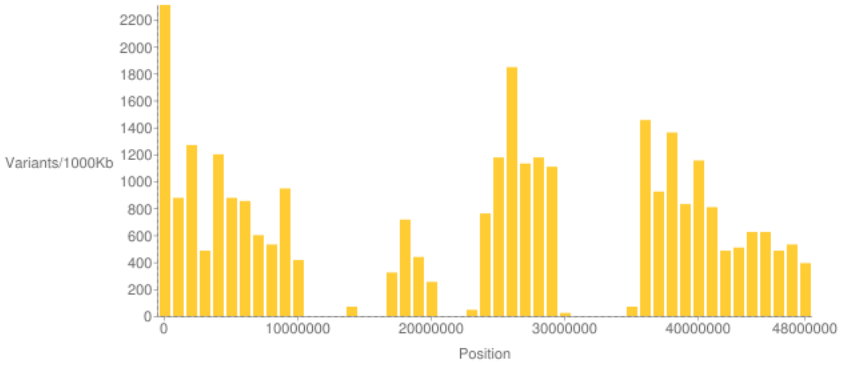

10000001, Position,0,1000000,2000000,3000000,4000000,5000000,6000000,7000000,8000000,9000000,10000000,11000000,1200000[illegible]
10000001,Count,2308,885,1287,494,1201,878,856,610,535,957,418,0,0,0,88,0,0,341,735,446,276,0,0,51,770,1184,1867,1149,1185,1121,45,0,0,

Details by gene

Here you can find a tab-separated table.

SnpEff: Variant analysis

Contents

Summary
Variant rate by chromosome
Variants by type
Number of variants by impact
Number of variants by functional class
Number of variants by effect
Quality histogram
InDel length histogram
Base variant table
Transition vs transversions (ts/tv)
Allele frequency
Allele Count
Codon change table
Amino acid change table
Chromosome variants plots
Details by gene

Summary

**Genome**	manindi_TA
**Date**	2021-05-22 14:53
**SnpEff version**	`SnpEff 5.0e (build 2021-03-09 06:01), by Pablo Cingolani`
**Command line arguments**	`SnpEff manindi_TA /home/cocogenomics/mango_genome/mango_TA_genome/M.altissima_TA_SNP_final.vcf`
**Warnings**	231,471
**Errors**	0
**Number of lines (input file)**	2,982,132
**Number of variants (before filter)**	2,990,377
**Number of not variants (i.e. reference equals alternative)**	0
**Number of variants processed (i.e. after filter and non-variants)**	2,990,377
**Number of known variants (i.e. non-empty ID)**	0 (0%)
**Number of multi-allelic VCF entries (i.e. more than two alleles)**	8,245
**Number of effects**	5,825,502
**Genome total length**	377,290,333
**Genome effective length**	377,290,333
**Variant rate**	1 variant every 126 bases

Variants rate details

Chromosome	Length	Variants	Variants rate
1	17,320,008	166,961	103
2	17,063,873	157,284	108
3	21,566,805	178,183	121
4	22,357,487	197,281	113
5	14,540,018	121,572	119
6	10,680,009	72,837	146
7	13,133,232	126,586	103
8	14,750,018	126,318	116
9	21,055,410	174,889	120
10	11,063,414	106,600	103
11	17,675,019	138,160	127
12	14,336,529	109,904	130
13	15,099,493	111,030	135
14	13,335,999	117,473	113
15	16,178,320	127,499	126
16	21,434,198	158,392	135
17	11,746,059	95,197	123
18	16,863,820	164,748	102
19	22,398,858	190,704	117
20	16,105,987	144,587	111
10000001	48,585,777	204,172	237
**Total**	**377,290,333**	**2,990,377**	**126**

Number variants by type

Type	Total
**SNP**	2,990,377
**MNP**	0
**INS**	0
**DEL**	0
**MIXED**	0
**INV**	0
**DUP**	0
**Total**	**2,990,377**

Type	Total
BND	0
INTERVAL	0
Total	2,990,377

Number of effects by impact

Type (alphabetical order)	Count	Percent
HIGH	2,935	0.05%
LOW	82,091	1.409%
MODERATE	88,532	1.52%
MODIFIER	5,651,944	97.021%

Number of effects by functional class

Type (alphabetical order)	Count	Percent
MISSENSE	88,943	56.55%
NONSENSE	1,393	0.886%
SILENT	66,946	42.564%

Missense / Silent ratio: 1.3286

Number of effects by type and region

Type

Type (alphabetical order)	Count	Percent
3_prime_UTR_variant	46,735	0.8%
5_prime_UTR_premature_start_codon_gain_variant	3,751	0.064%
5_prime_UTR_variant	24,082	0.412%
downstream_gene_variant	1,300,734	22.27%
initiator_codon_variant	33	0.001%
intergenic_region	2,196,966	37.615%
intragenic_variant	80	0.001%
intron_variant	675,164	11.56%
missense_variant	88,532	1.516%
splice_acceptor_variant	580	0.01%
splice_donor_variant	584	0.01%
splice_region_variant	14,905	0.255%
start_lost	147	0.003%
stop_gained	1,393	0.024%
stop_lost	231	0.004%
stop_retained_variant	133	0.002%
synonymous_variant	66,813	1.144%
upstream_gene_variant	1,419,809	24.309%

Region

Type (alphabetical order)	Count	Percent
DOWNSTREAM	1,300,734	22.328%
EXON	155,843	2.675%
INTERGENIC	2,196,966	37.713%
INTRON	663,538	11.39%
SPLICE_SITE_ACCEPTOR	580	0.01%
SPLICE_SITE_DONOR	584	0.01%
SPLICE_SITE_REGION	12,800	0.22%
TRANSCRIPT	80	0.001%
UPSTREAM	1,419,809	24.372%
UTR_3_PRIME	46,735	0.802%
UTR_5_PRIME	27,833	0.478%

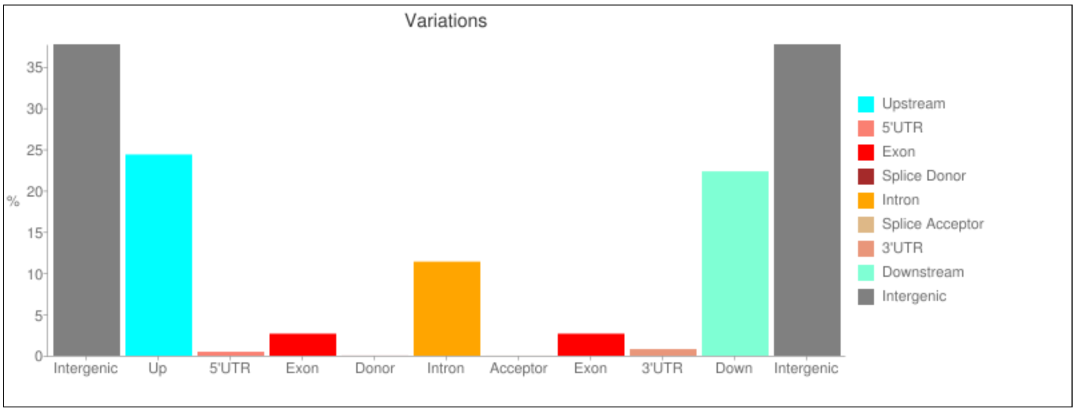

Quality:

Min	10
Max	34,778
Mean	146.166
Median	113
Standard deviation	359.051
Values	10,11,12,13,14,15,16,17,18,19,20,21,22,23,24,25,26,27,28,29,30,31,32,33,34,35,36,37,38,39,40,41,42,43,44,45,46,47,48,49,50,51,52,53,54,55,56,57
Count	2215,1911,12484,4136,3771,3476,5491,6871,11867,12221,15222,15080,19239,18231,20842,25625,20514,19249,21333,12067,10122,14993,10414,

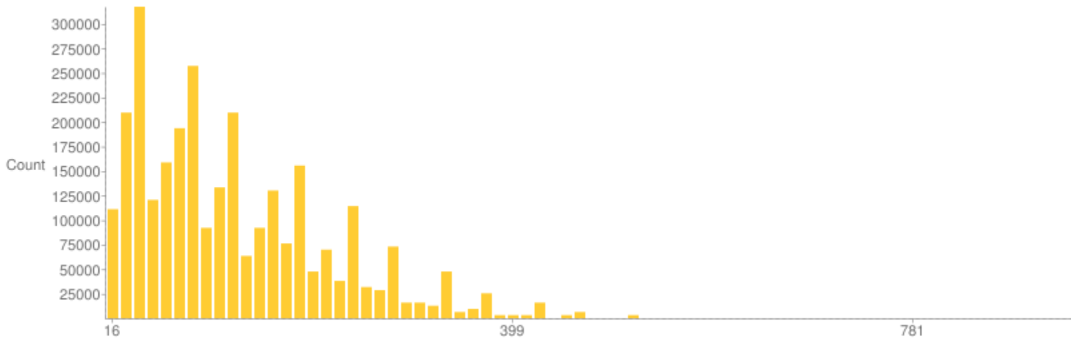

Insertions and deletions length:

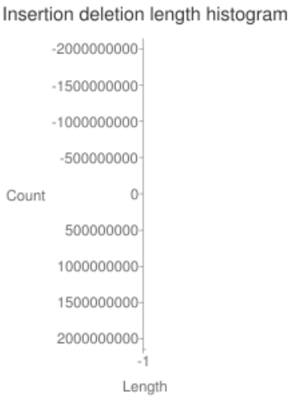

Base changes (SNPs)

	A	C	G	T
**A**	0	104,098	495,278	160,022
**C**	113,798	0	70,773	551,320
**G**	553,629	70,728	0	113,039
**T**	159,351	494,450	103,891	0

Ts/Tv (transitions / transversions)

Note: Only SNPs are used for this statistic.
Note: This Ts/Tv ratio is a 'raw' ratio (ratio of observed events).

**Transitions**	2,901,190
**Transversions**	1,234,945
**Ts/Tv ratio**	2.3492

All variants:

```
Sample ,readname,Total
Transitions ,2901190,2901190
Transversions ,1234945,1234945
Ts/Tv ,2.349,2.349
```

Only known variants (i.e. the ones having a non-empty ID field):

```
No results available (empty input?)
```

Allele frequency

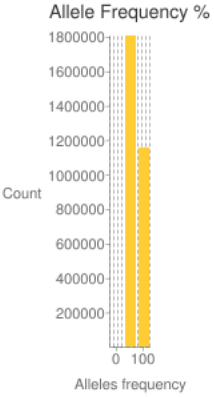

**Min**	0
**Max**	100
**Mean**	69.625
**Median**	50
**Standard deviation**	24.486
**Values**	0,50,100
**Count**	2053,1807533,1172546

Allele Count

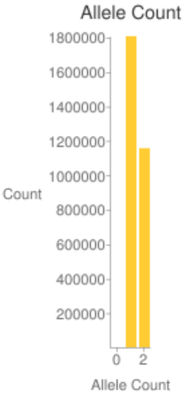

**Min**	0
**Max**	2
**Mean**	1.393
**Median**	1
**Standard deviation**	0.49
**Values**	0,1,2
**Count**	2053,1807533,1172546

Hom/Het per sample

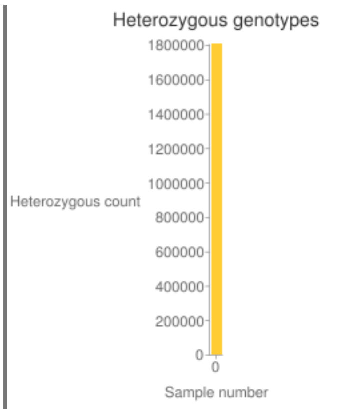

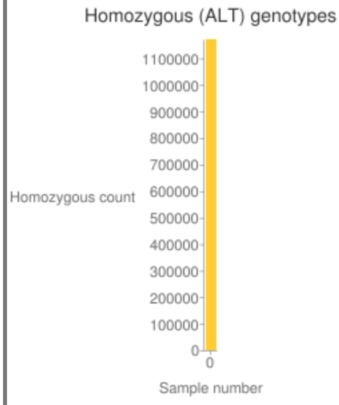

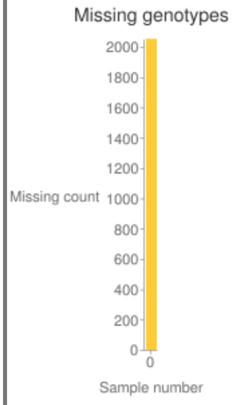


```
Sample_names , readname
Reference , 0
Het , 1807533
Hom , 1172546
Missing , 2053
```

Codon changes

How to read this table:
- Rows are reference codons and columns are changed codons. E.g. Row 'AAA' column 'TAA' indicates how many 'AAA' codons have been replaced by 'TAA' codons.
- Red background colors indicate that more changes happened (heat-map).
- Diagonals are indicated using grey background color
- WARNING: This table may include different translation codon tables (e.g. mamalian DNA and mitochondrial DNA).

	AAA	AAC	AAG	AAT	ACA	ACC	ACG	ACT	AGA	AGC	AGG	AGT	ATA	ATC	ATG	ATT	CAA	CAC	CAG	CAT	CCA	CCC
AAA		195	1,074	294	202				564				147				282					
AAC	214		217	1,152		109				474				78				81				
AAG	1,190	183		286			100				585				205				196			
AAT	332	896	216					201				615				214				222		
ACA	241					220	740	366	225				427								91	
ACC		162			182		106	1,027		132				229								51
ACG			149		967	149		220			89				514							
ACT				249	381	764	165					270				501						
AGA	665				144					93	548	105	119									
AGC		519				132			97		101	730		63								
AGG			577				64		593	115		137			95							
AGT				715				275	109	600	120					212						
ATA	148				348				106					229	509	360						
ATC		79				197				74			295		165	984						
ATG			182				391				118		651	184		262						
ATT				197				407				157	338	845	247							
CAA	280																	95	794	190	143	

	AAA	AAC	AAG	AAT	ACA	ACC	ACG	ACT	AGA	AGC	AGG	AGT	ATA	ATC	ATG	ATT	CAA	CAC	CAG	CAT	CCA	CCC
**CAC**		80															110		123	636		40
**CAG**			213														843	111		171		
**CAT**				244													184	469	131			
**CCA**					130												136					175
**CCC**						49												38			176	
**CCG**							33												86		1,195	135
**CCT**								142												108	361	579
**CGA**									222								622				74	
**CGC**										64								308				34
**CGG**											172								553			
**CGT**												85								705		
**CTA**													142				149				239	
**CTC**														107				53				98
**CTG**															163				135			
**CTT**																269				149		
**GAA**	903																305					
**GAC**		425																71				
**GAG**			772																292			
**GAT**				876																223		
**GCA**					749																169	
**GCC**						383																80
**GCG**							165															
**GCT**								788														
**GGA**									439													
**GGC**										363												
**GGG**											315											
**GGT**												613										
**GTA**													544									
**GTC**														461								
**GTG**															511							
**GTT**																1,045						
**TAA**	16																25					
**TAC**		107																190				
**TAG**			8																21			
**TAT**				186																427		
**TCA**					175																319	
**TCC**						78																163
**TCG**							37															
**TCT**								231														
**TGA**									5													
**TGC**										87												
**TGG**											70											
**TGT**												129										
**TTA**													147									
**TTC**														96								
**TTG**															268							
**TTT**																218						

Amino acid changes

How to read this table:
- Rows are reference amino acids and columns are changed amino acids. E.g. Row 'A' column 'E' indicates how many 'A' amino acids have been replaced by 'E' amino acids.
- Red background colors indicate that more changes happened (heat-map).
- Diagonals are indicated using grey background color
- WARNING: This table may include different translation codon tables (e.g. mamalian DNA and mitochondrial DNA).

	*	A	C	D	E	F	G	H	I	K	L	M	N	P	Q	R	S	T	V	W	Y
*****	**133**		6		18		5			24	30				46	35	20			24	23
**A**		**6,208**		232	363		602							422			796	2,085	1,947		
**C**	51		**1,077**			211	131									442	520			101	511
**D**		213		**2,566**	1,345		713	294					1,301						170		310
**E**	149	310		1,160	**2,182**		789			1,675					597				267		
**F**			184			**2,186**			314		1,315						583		305		397
**G**	48	606	212	812	872		**4,654**									1,018	976		456	92	
**H**				274				**1,105**			182		324	131	548	657					680
**I**						344			**3,051**	148	596	921	276			106	231	952	1,635		
**K**	103				1,418				147	**2,264**		205	958		478	1,149		302			
**L**	113					1,595		202	665		**10,120**	431		888	284	241	868		928	84	
**M**									1,097	182	467					118		391	442		
**N**				1,136				303	292	979			**2,048**				1,089	310			287
**P**		414						146			1,113			**5,149**	222	222	1,202	354			
**Q**	274				592			567		493	279			234	**1,637**	970					
**R**	200		695				909	1,013	119	1,242	292	95		253	1,175	**3,560**	599	208		354	

	*	A	C	D	E	F	G	H	I	K	L	M	N	P	Q	R	S	T	V	W	Y
**S**	130	683	579			659	920		275		1,107		1,234	969		563	**7,309**	928		50	266
**T**		1,715							1,157	390		514	411	300		314	954	**5,287**			
**V**		1,668		174	252	407	351		2,050		1,097	511							**4,921**		
**W**	195		126				57				128					270	52				
**Y**	130		476	223		401		617					293				219				**1,489**

Variants by chromosome

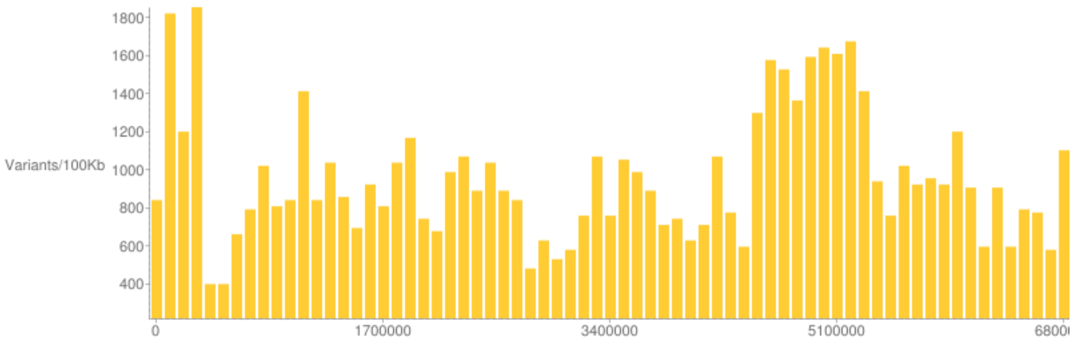

1, Position,0,100000,200000,300000,400000,500000,600000,700000,800000,900000,1000000,1100000,1200000,1300000,1400000,
1,Count,839,1829,1206,1850,407,411,660,803,1027,815,851,1422,838,1037,870,696,920,813,1047,1167,742,682,996,1067,887,1040,891,848,485,

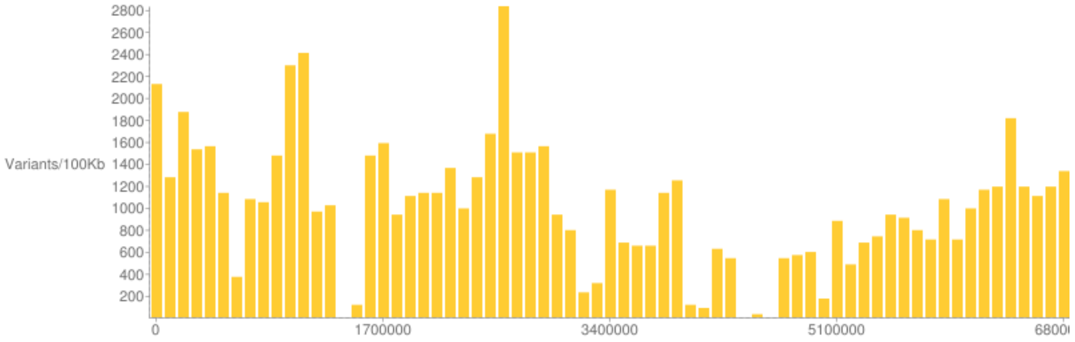

2, Position,0,100000,200000,300000,400000,500000,600000,700000,800000,900000,1000000,1100000,1200000,1300000,1400000,
2,Count,2153,1282,1883,1544,1583,1139,386,1082,1068,1477,2306,2415,976,1030,18,122,1490,1599,956,1134,1163,1161,1365,1018,1286,1678,2[illegible]

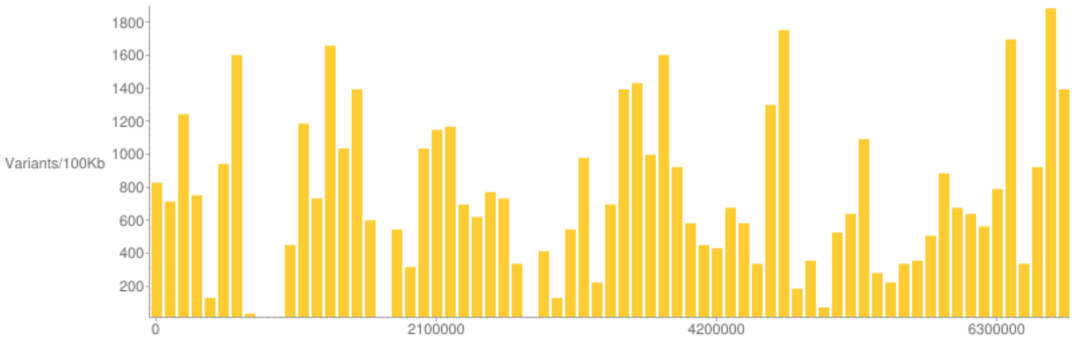

3, Position,0,100000,200000,300000,400000,500000,600000,700000,800000,900000,1000000,1100000,1200000,1300000,1400000,
3,Count,836,724,1241,763,139,950,1614,40,25,24,448,1198,733,1668,1034,1394,606,16,543,317,1049,1149,1174,707,628,774,741,344,11,415,1[illegible]

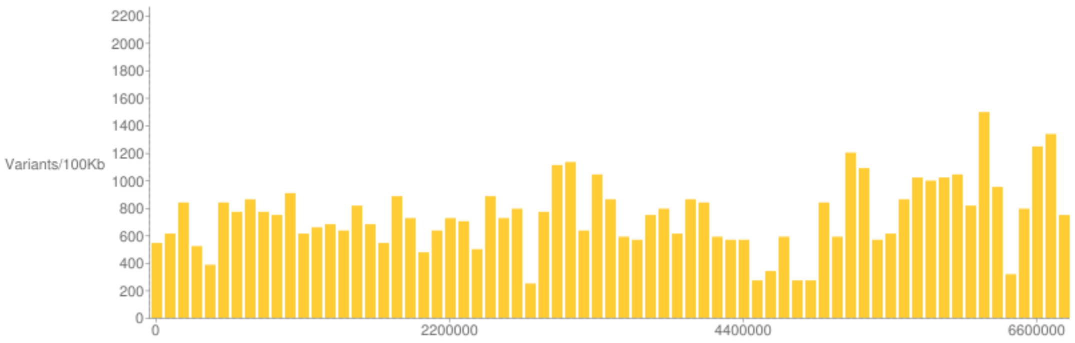

4, Position,0,100000,200000,300000,400000,500000,600000,700000,800000,900000,1000000,1100000,1200000,1300000,1400000,
4,Count,557,617,843,543,398,856,780,876,785,761,912,612,668,690,643,817,699,554,886,747,487,642,738,725,515,906,741,814,262,783,1126,

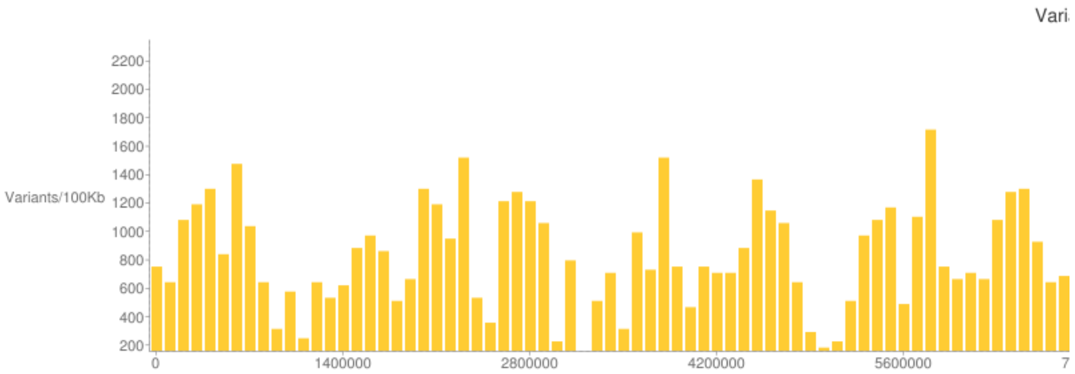

5, Position,0,100000,200000,300000,400000,500000,600000,700000,800000,900000,1000000,1100000,1200000,1300000,1400000,
5,Count,764,657,1080,1207,1309,842,1487,1053,649,325,584,253,639,538,636,888,977,875,521,678,1317,1194,953,1521,547,364,1226,1278,120

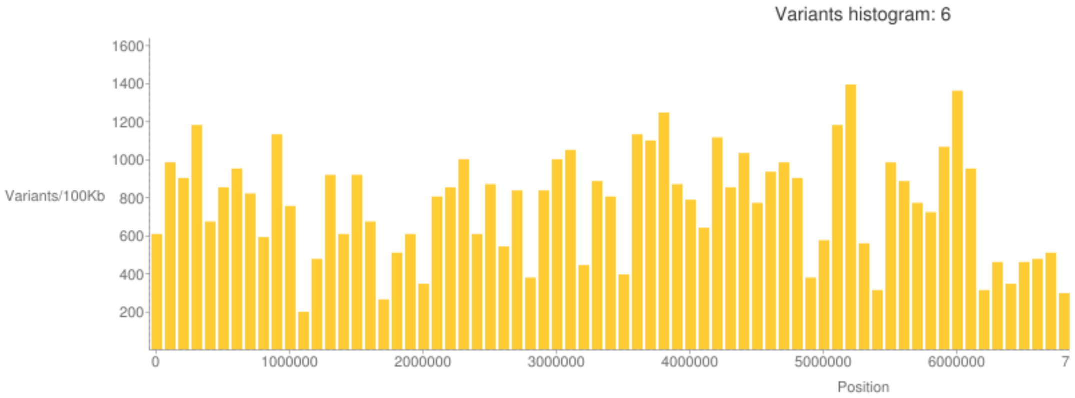

6, Position,0,100000,200000,300000,400000,500000,600000,700000,800000,900000,1000000,1100000,1200000,1300000,1400000,
6,Count,620,994,916,1184,686,856,951,833,602,1138,770,209,478,919,607,924,676,265,516,607,345,813,855,1005,609,875,556,842,393,848,10

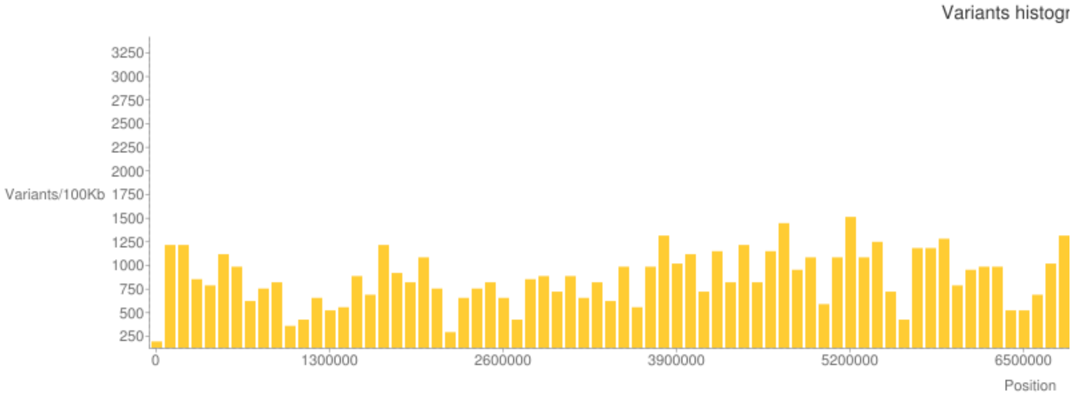

7, Position,0,100000,200000,300000,400000,500000,600000,700000,800000,900000,1000000,1100000,1200000,1300000,1400000,
7,Count,217,1217,1228,848,787,1113,989,638,754,823,363,428,665,550,574,892,711,1233,928,841,1099,767,318,676,776,834,682,439,863,885,

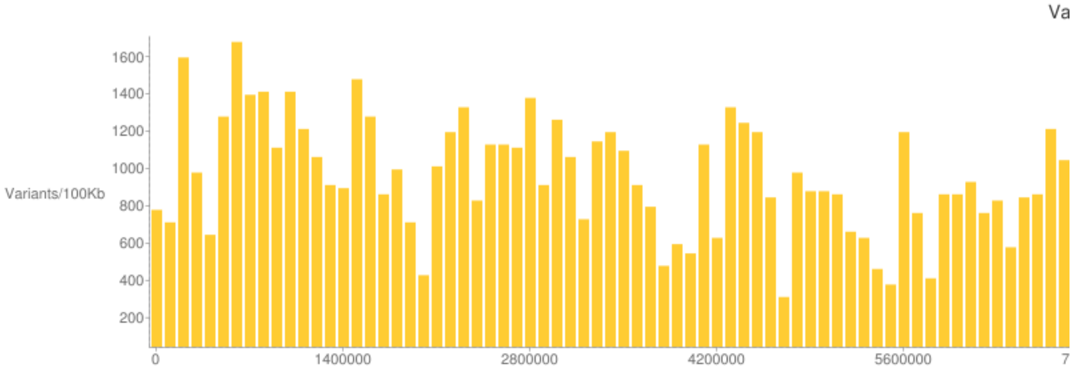

8, Position,0,100000,200000,300000,400000,500000,600000,700000,800000,900000,1000000,1100000,1200000,1300000,1400000,
8,Count,779,712,1602,979,655,1290,1691,1398,1411,1113,1409,1212,1072,921,903,1490,1277,868,1003,709,424,1021,1205,1335,830,1129,1132,

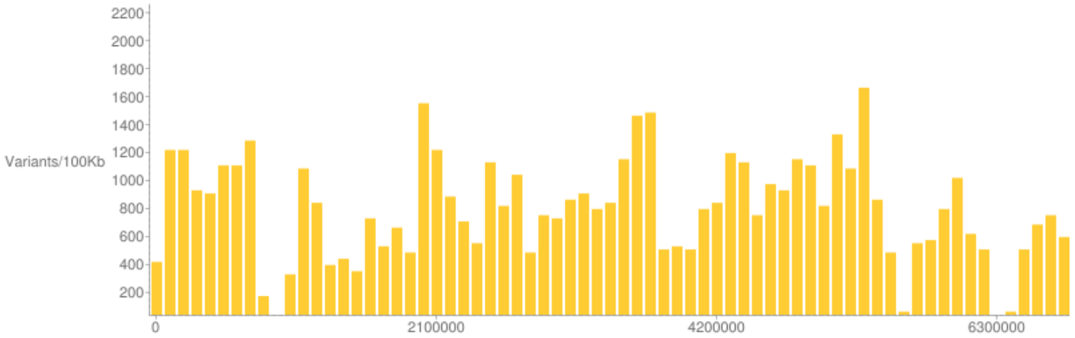

9, Position,0,100000,200000,300000,400000,500000,600000,700000,800000,900000,1000000,1100000,1200000,1300000,1400000,
9,Count,434,1227,1225,943,910,1106,1120,1295,169,39,341,1093,845,403,447,351,731,531,663,482,1563,1222,902,709,565,1145,816,1039,480,

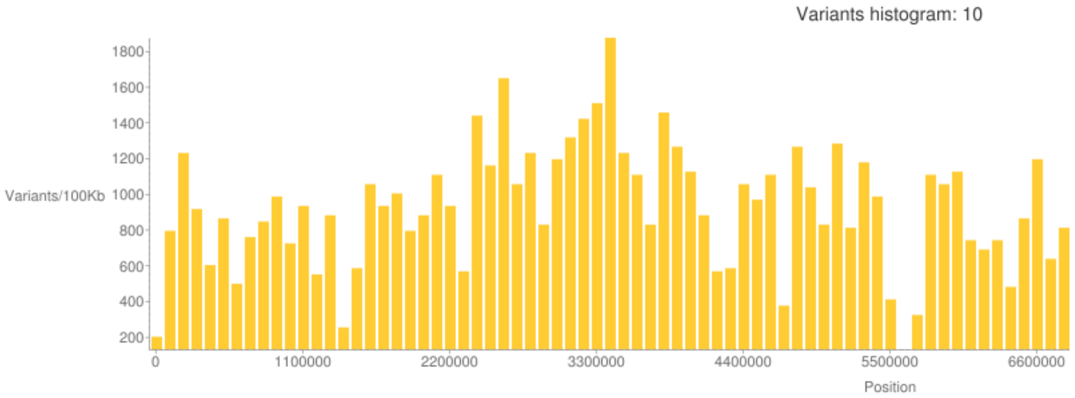

10, Position,0,100000,200000,300000,400000,500000,600000,700000,800000,900000,1000000,1100000,1200000,1300000,1400000,
10,Count,210,809,1232,922,611,873,511,772,859,993,740,941,553,884,266,591,1067,943,1018,802,891,1111,946,568,1445,1172,1653,1057,1240,

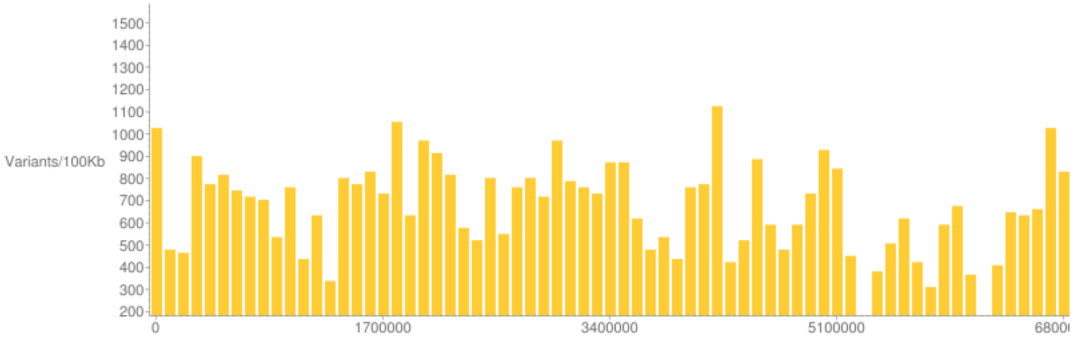

11, Position,0,100000,200000,300000,400000,500000,600000,700000,800000,900000,1000000,1100000,1200000,1300000,1400000,
11,Count,1031,480,475,908,780,826,753,721,706,537,760,443,641,348,811,784,831,731,1052,638,976,925,815,586,523,810,550,770,806,719,97

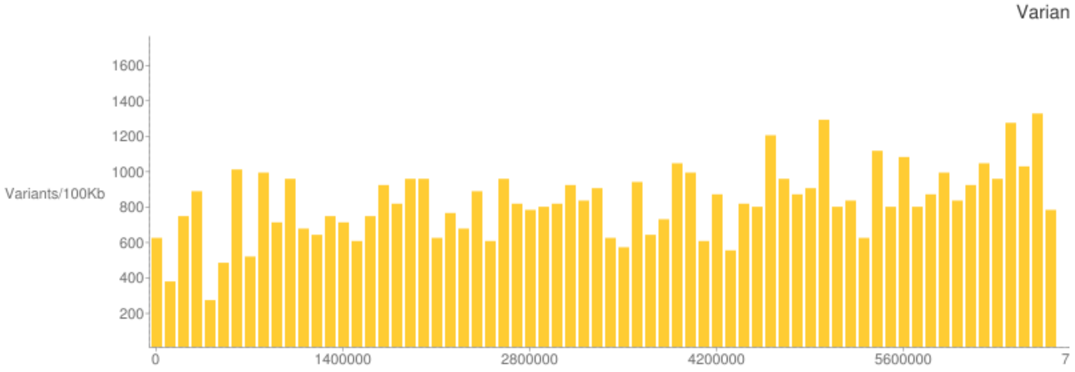

12, Position,0,100000,200000,300000,400000,500000,600000,700000,800000,900000,1000000,1100000,1200000,1300000,1400000,
12,Count,624,390,748,886,274,490,1014,522,1007,727,971,682,645,755,724,614,759,925,825,966,957,626,776,682,901,618,960,829,781,809,82[illegible]

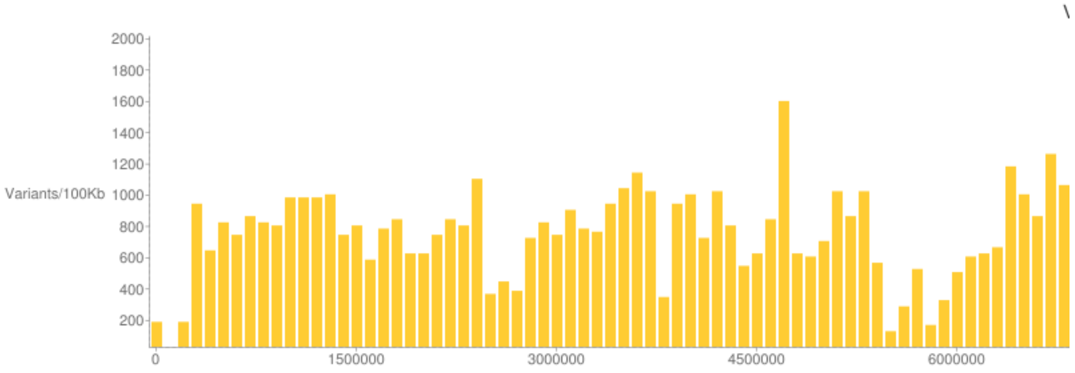

13, Position,0,100000,200000,300000,400000,500000,600000,700000,800000,900000,1000000,1100000,1200000,1300000,1400000,
13,Count,192,27,197,958,660,826,757,865,827,807,996,991,995,1007,762,815,590,788,855,643,635,746,851,803,1113,375,448,391,738,832,748,

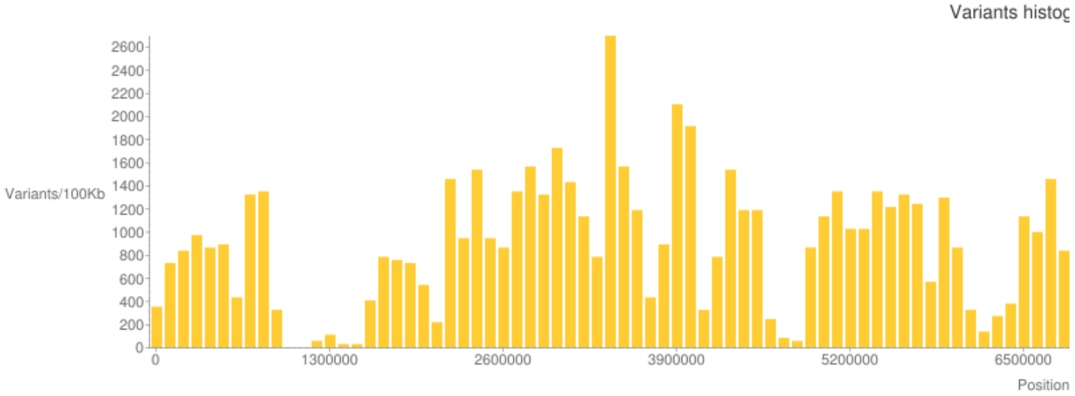

14, Position,0,100000,200000,300000,400000,500000,600000,700000,800000,900000,1000000,1100000,1200000,1300000,1400000,
14,Count,356,738,855,992,877,890,456,1323,1369,338,0,11,77,121,40,50,408,782,774,740,552,217,1464,957,1550,953,883,1371,1566,1335,173[illegible]

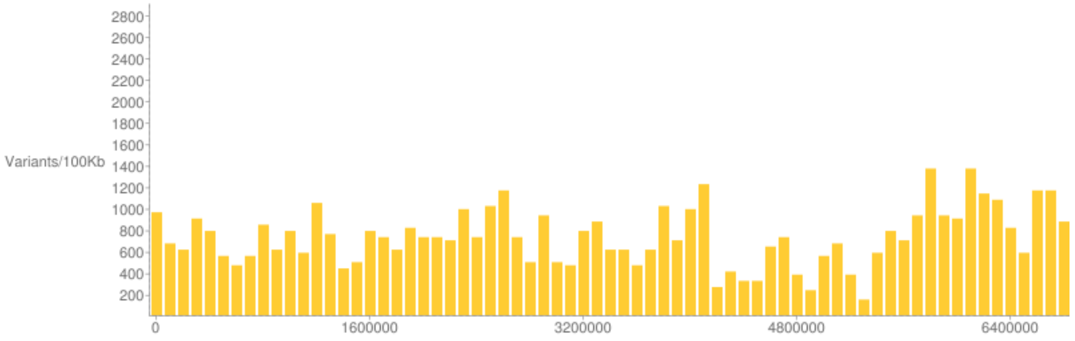

15, Position,0,100000,200000,300000,400000,500000,600000,700000,800000,900000,1000000,1100000,1200000,1300000,1400000,
15,Count,985,680,636,915,813,573,481,583,864,620,813,608,1077,777,459,504,807,760,621,834,750,752,711,1012,763,1026,1190,743,527,966,[illegible]

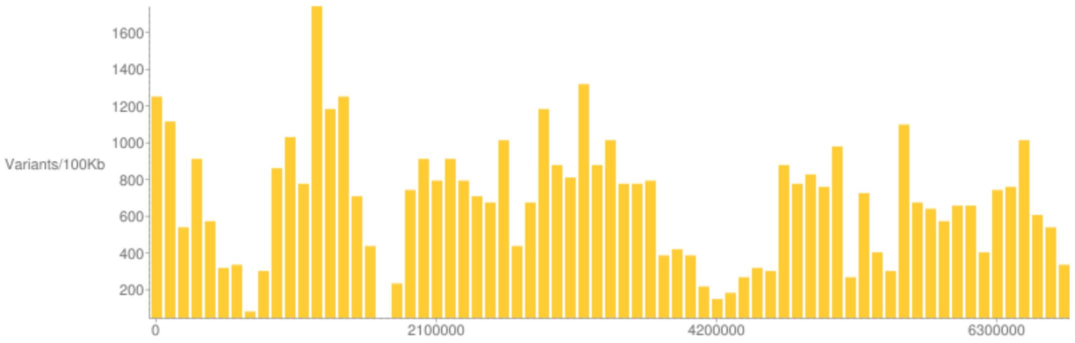


```
16, Position,0,100000,200000,300000,400000,500000,600000,700000,800000,900000,1000000,1100000,1200000,1300000,1400000,
16,Count,1256,1128,540,920,586,332,336,89,305,860,1032,784,1740,1195,1265,711,436,44,241,750,922,804,923,801,713,678,1021,449,678,118[illegible]
```

Variants histogram: 17

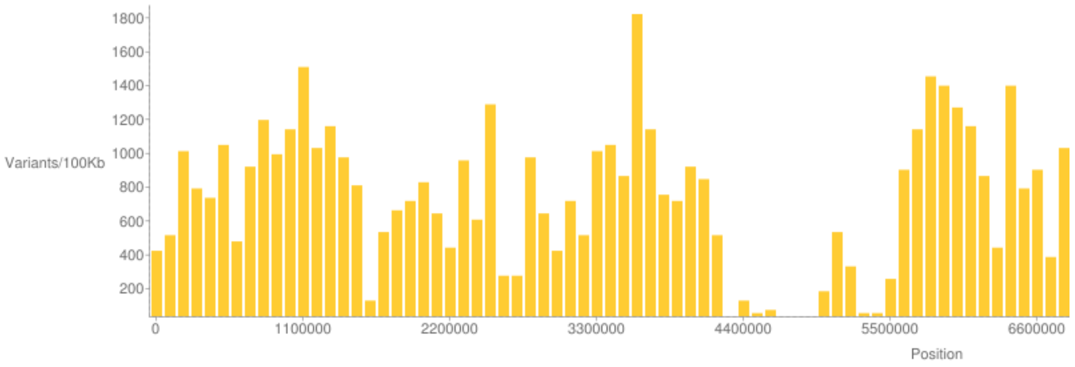


```
17, Position,0,100000,200000,300000,400000,500000,600000,700000,800000,900000,1000000,1100000,1200000,1300000,1400000,
17,Count,438,516,1017,789,739,1059,493,918,1205,993,1156,1513,1031,1171,988,817,130,540,661,727,827,657,449,954,618,1303,275,286,987,[illegible]
```

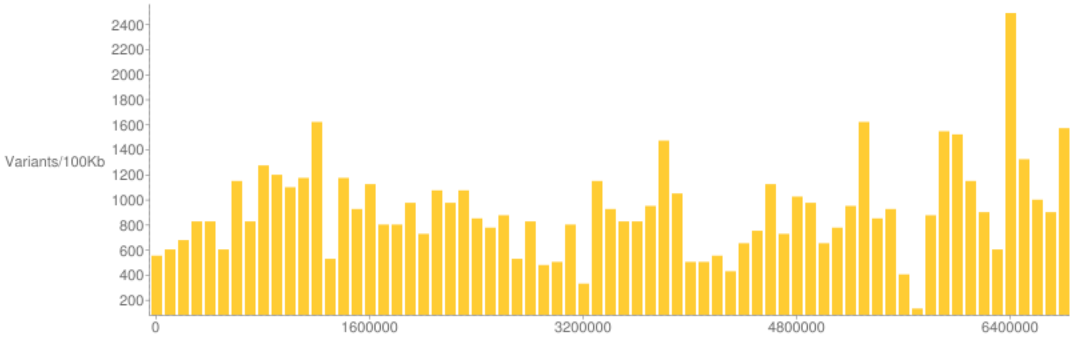


```
18, Position,0,100000,200000,300000,400000,500000,600000,700000,800000,900000,1000000,1100000,1200000,1300000,1400000,
18,Count,558,609,694,838,831,601,1165,840,1277,1203,1103,1174,1620,545,1188,942,1146,806,810,990,748,1074,982,1090,862,792,879,530,83[illegible]
```

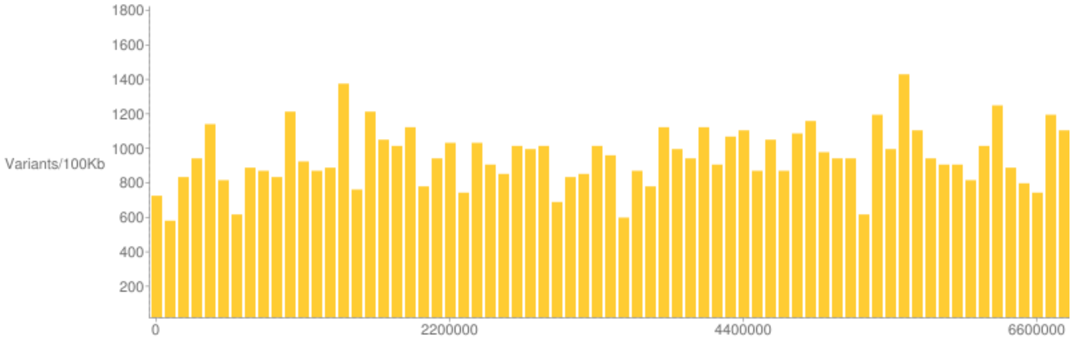


```
19, Position,0,100000,200000,300000,400000,500000,600000,700000,800000,900000,1000000,1100000,1200000,1300000,1400000,
19,Count,726,583,830,954,1152,820,617,895,866,836,1216,936,875,888,1374,773,1217,1059,1017,1134,778,948,1038,745,1046,918,862,1015,10[illegible]
```

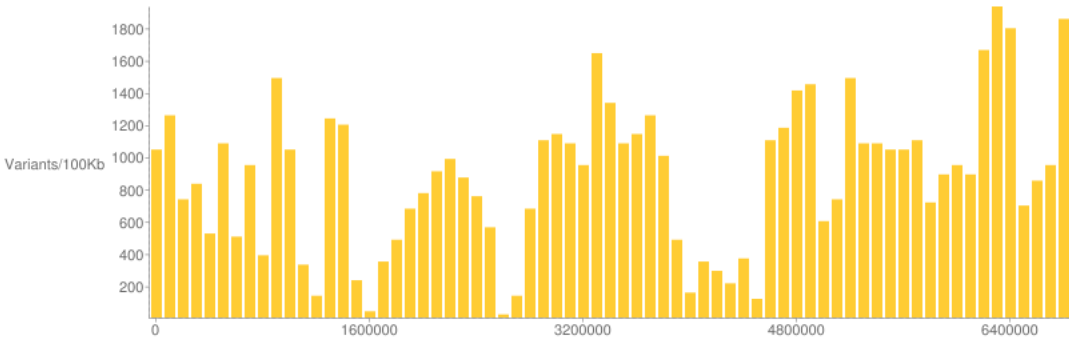


```
20, Position,0,100000,200000,300000,400000,500000,600000,700000,800000,900000,1000000,1100000,1200000,1300000,1400000,
20,Count,1049,1266,753,843,535,1100,510,966,393,1506,1066,350,150,1259,1203,248,63,357,498,687,797,930,992,884,767,583,44,143,696,111
```

Variants histogram: 10000001

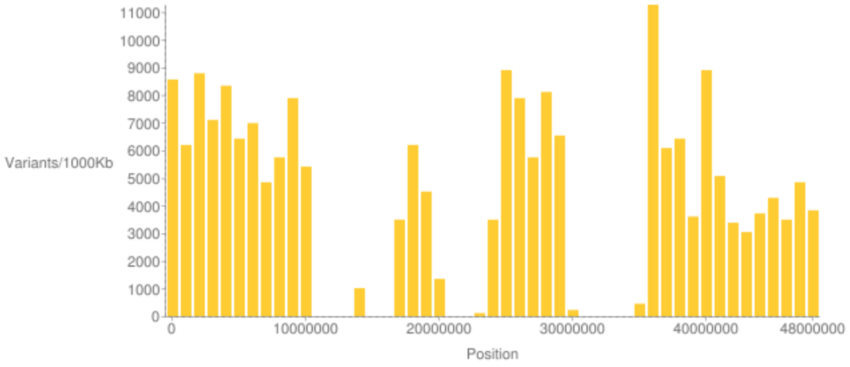


```
10000001, Position,0,1000000,2000000,3000000,4000000,5000000,6000000,7000000,8000000,9000000,10000000,11000000,120000
10000001,Count,8603,6286,8894,7147,8341,6484,7056,4860,5808,7898,5453,0,0,0,1039,0,0,3562,6201,4580,1411,0,0,150,3523,8923,7910,5785,
```

Details by gene

Here you can find a tab-separated table.

SnpEff: Variant analysis

Contents

Summary
Variant rate by chromosome
Variants by type
Number of variants by impact
Number of variants by functional class
Number of variants by effect
Quality histogram
InDel length histogram
Base variant table
Transition vs transversions (ts/tv)
Allele frequency
Allele Count
Codon change table
Amino acid change table
Chromosome variants plots
Details by gene

Summary

**Genome**	manindi_TA
**Date**	2021-05-22 15:16
**SnpEff version**	`SnpEff 5.0e (build 2021-03-09 06:01), by Pablo Cingolani`
**Command line arguments**	`SnpEff manindi_TA /home/cocogenomics/mango_genome/mango_TA_genome/M.altissima_TA_INDEL_final.vcf`
**Warnings**	47,128
**Errors**	0
**Number of lines (input file)**	457,943
**Number of variants (before filter)**	459,540
**Number of not variants (i.e. reference equals alternative)**	0
**Number of variants processed (i.e. after filter and non-variants)**	459,540
**Number of known variants (i.e. non-empty ID)**	0 (0%)
**Number of multi-allelic VCF entries (i.e. more than two alleles)**	1,597
**Number of effects**	964,442
**Genome total length**	377,290,333
**Genome effective length**	377,290,333
**Variant rate**	1 variant every 821 bases

Variants rate details

Chromosome	Length	Variants	Variants rate
1	17,320,008	25,567	677
2	17,063,873	25,106	679
3	21,566,805	29,060	742
4	22,357,487	31,861	701
5	14,540,018	19,059	762
6	10,680,009	11,014	969
7	13,133,232	19,424	676
8	14,750,018	19,459	758
9	21,055,410	26,993	780
10	11,063,414	15,570	710
11	17,675,019	21,265	831
12	14,336,529	17,907	800
13	15,099,493	16,271	928
14	13,335,999	18,239	731
15	16,178,320	19,425	832
16	21,434,198	25,101	853
17	11,746,059	14,089	833
18	16,863,820	25,704	656
19	22,398,858	29,637	755
20	16,105,987	21,020	766
10000001	48,585,777	27,769	1,749
**Total**	**377,290,333**	**459,540**	**821**

Number variants by type

Type	Total
**SNP**	0
**MNP**	0
**INS**	223,650
**DEL**	235,890
**MIXED**	0
**INV**	0
**DUP**	0
**Total**	**459,540**

Type	Total
BND	0
INTERVAL	0
Total	459,540

Number of effects by impact

Type (alphabetical order)	Count	Percent
HIGH	5,301	0.55%
LOW	2,138	0.222%
MODERATE	2,668	0.277%
MODIFIER	954,335	98.952%

Number of effects by functional class

Type (alphabetical order)	Count	Percent

Missense / Silent ratio: 0

Number of effects by type and region

Type

Type (alphabetical order)	Count	Percent
3_prime_UTR_truncation	1	0%
3_prime_UTR_variant	9,645	0.997%
5_prime_UTR_variant	5,935	0.613%
bidirectional_gene_fusion	2	0%
conservative_inframe_deletion	489	0.051%
conservative_inframe_insertion	637	0.066%
disruptive_inframe_deletion	947	0.098%
disruptive_inframe_insertion	692	0.072%
downstream_gene_variant	228,871	23.648%
exon_loss_variant	3	0%
frameshift_variant	4,857	0.502%
intergenic_region	338,484	34.974%
intragenic_variant	21	0.002%
intron_variant	114,750	11.857%
non_coding_transcript_variant	209	0.022%
splice_acceptor_variant	221	0.023%
splice_donor_variant	244	0.025%
splice_region_variant	2,647	0.274%
start_lost	94	0.01%
start_retained_variant	8	0.001%
stop_gained	192	0.02%
stop_lost	76	0.008%
stop_retained_variant	12	0.001%
upstream_gene_variant	258,779	26.738%

Region

Type (alphabetical order)	Count	Percent
DOWNSTREAM	228,865	23.73%
EXON	7,618	0.79%
GENE	2	0%
INTERGENIC	338,484	35.096%
INTRON	112,469	11.662%
SPLICE_SITE_ACCEPTOR	181	0.019%
SPLICE_SITE_DONOR	188	0.019%
SPLICE_SITE_REGION	2,138	0.222%
TRANSCRIPT	230	0.024%
UPSTREAM	258,779	26.832%
UTR_3_PRIME	9,579	0.993%
UTR_5_PRIME	5,909	0.613%

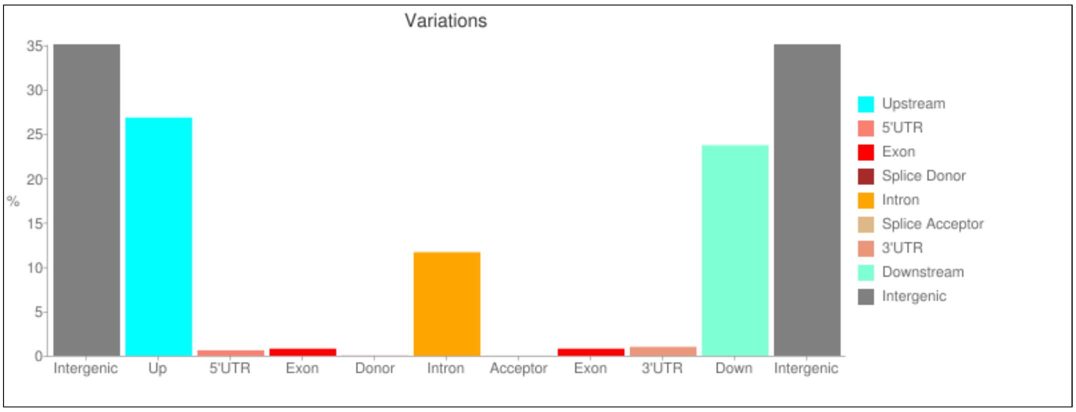

Quality:

**Min**	10
**Max**	39,799
**Mean**	125.413
**Median**	98
**Standard deviation**	304.402
**Values**	10,11,12,13,14,15,16,17,18,19,20,21,22,23,24,25,26,27,28,29,30,31,32,33,34,35,36,37,38,39,40,41,42,43,44,45,46,47,48,49,50,51,52,53,54,55,56,57
**Count**	928,1285,1824,1325,1249,1310,1358,1372,1465,1493,1485,1542,2332,1489,1457,2885,1420,1465,3759,1423,1623,6009,1848,1630,8409,1352,133[illegible]

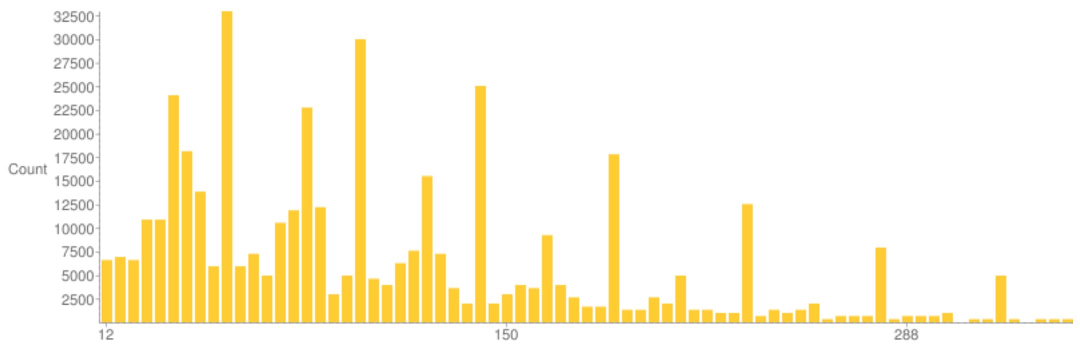

Insertions and deletions length:

**Min**	0
**Max**	152
**Mean**	2.189
**Median**	1
**Standard deviation**	5.784
**Values**	0,1,2,3,4,5,6,7,8,9,10,11,12,13,14,15,16,17,18,19,20,21,22,23,24,25,26,27,28,29,30,31,32,33,34,35,36,37,38,39,40,41,42,43,44,45,46,47,48,49,50,51
**Count**	106564,275312,16970,11768,6581,5419,3478,3542,3075,3074,2509,2570,1804,1610,1333,1232,983,1009,852,757,751,663,520,509,431,442,394,36

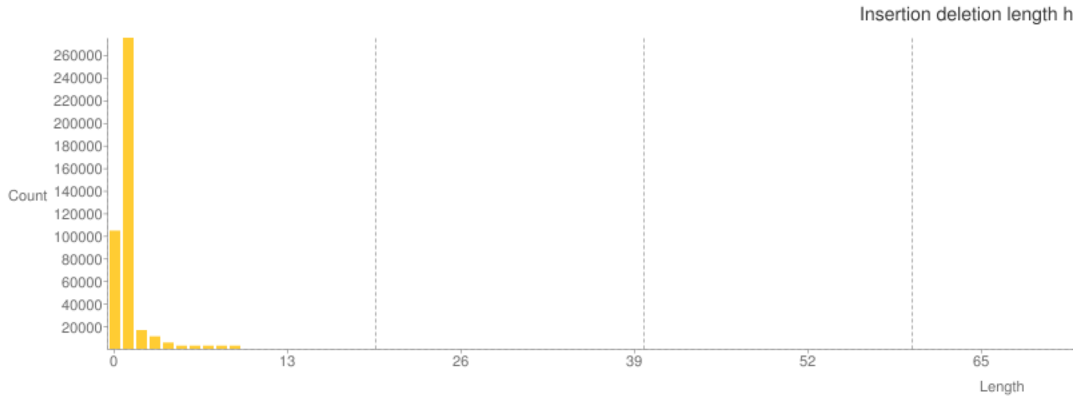

Base changes (SNPs)

	A	C	G	T
**A**	0	0	0	0
**C**	0	0	0	0
**G**	0	0	0	0
**T**	0	0	0	0

Ts/Tv (transitions / transversions)

Note: Only SNPs are used for this statistic.
Note: This Ts/Tv ratio is a 'raw' ratio (ratio of observed events).

**Transitions**	0
**Transversions**	0
**Ts/Tv ratio**	0

All variants:

```
No results available (empty input?)
```

Only known variants (i.e. the ones having a non-empty ID field):

```
No results available (empty input?)
```

Allele frequency

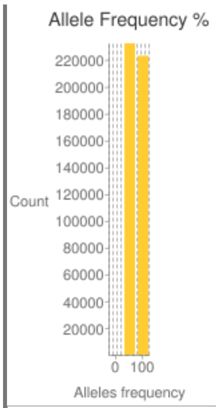

**Min**	0
**Max**	100
**Mean**	74.578
**Median**	50
**Standard deviation**	25.042
**Values**	0,50,100
**Count**	209,232417,225317

Allele Count

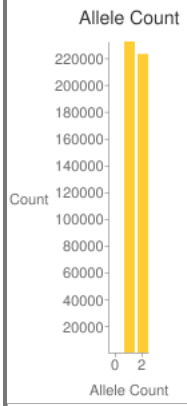

**Min**	0
**Max**	2
**Mean**	1.492
**Median**	1
**Standard deviation**	0.501
**Values**	0,1,2
**Count**	209,232417,225317

Hom/Het per sample

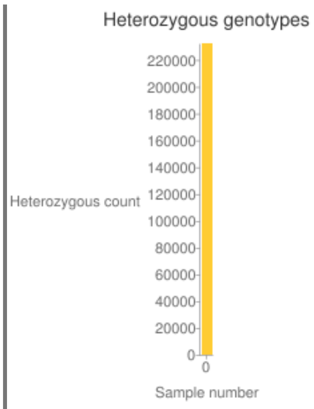

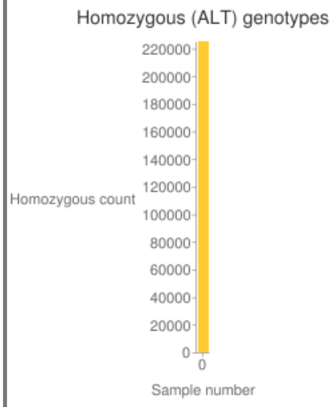

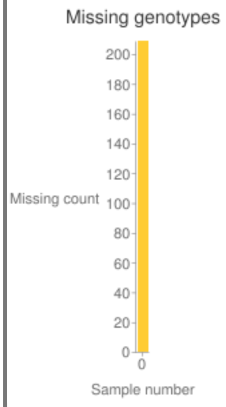


```
Sample_names , readname
Reference , 0
Het , 232417
Hom , 225317
Missing , 209
```

Codon changes

How to read this table:
- Rows are reference codons and columns are changed codons. E.g. Row 'AAA' column 'TAA' indicates how many 'AAA' codons have been replaced by 'TAA' codons.
- Red background colors indicate that more changes happened (heat-map).
- Diagonals are indicated using grey background color
- WARNING: This table may include different translation codon tables (e.g. mamalian DNA and mitochondrial DNA).

	-	AAA	AAC	AAG	AAT	ACA	ACC	ACG	ACT	AGA	AGC	AGG	AGT	ATA	ATC	ATG	ATT	CAA	CAC	CAG	CAT	CCA	CC
**-**		149	85	150	147	113	54	43	114	95	61	51	88	75	54	85	101	148	50	106	98	145	4
**AAA**	250	**44**	4	35	16	4	1		5	11	3	2		3	3	3	2	4		2		2	
**AAC**	147	22	**4**	21	21	6		3	2	6	2	4		1	1	4	1	4	1				
**AAG**	287	27	10	**26**	15	5	1	1	1	3		1	1	2		4	1	1					
**AAT**	269	35	11	10	**14**	3	2	1	5	9	4	2	2	4	3	3	3	1			1		
**ACA**	141	3	9	2	1	**10**	11	7	13	1	4	2	2	1		2	3						
**ACC**	87		3	3	2	12	**4**	2	9		2	2	3	1	3		2		1				
**ACG**	44			2		7	3	**2**	7			1		2		1							
**ACT**	136	3	10	2	2	13	9	6	**10**	2	3	2	5		3	3	5	1	1	3		1	
**AGA**	153	4		9	2	2	1	1	1	**7**	3	16	7	2	1	2				1			
**AGC**	68		5	8	2	1	1			9	**4**	4	4			2				3			
**AGG**	106	5	1	5	6	2	2		1	18		**7**	5	2		3					1		
**AGT**	130	5	2	7	5	3	1	2	4	9	8	14	**11**	1		1	4	1					
**ATA**	105			4	7	3		2	1	2		1	3	**8**	5	4	11					1	
**ATC**	68	2	2	2	8	3	4	1	1				1	4		2	4						
**ATG**	228	6		2	32	4		2	6	3	2	2	1	6	4	**19**	10			1	1		
**ATT**	171	1	2	1	10			1	5	4	1		2	14	11	5	**15**		1				

	-	AAA	AAC	AAG	AAT	ACA	ACC	ACG	ACT	AGA	AGC	AGG	AGT	ATA	ATC	ATG	ATT	CAA	CAC	CAG	CAT	CCA	CC[illegible]
**CAA**	183																	**18**		18	17	6	
**CAC**	80					1												5	**3**	4	16	4	
**CAG**	151		1		1	1									1			20	3	**5**	7	2	
**CAT**	138				1	2									1			17	8	9	**13**	3	
**CCA**	177		1	2			3											2	3	3	2	**10**	1
**CCC**	86						1															10	**17**
**CCG**	58			1	1		1					1						1		4		11	
**CCT**	154		1				2			1						1		1		4	3	22	1
**CGA**	35							1			1							3	1	1	1		
**CGC**	30																	1	1	1	1		
**CGG**	31																	1		1			
**CGT**	47																	3	1	1	1	1	
**CTA**	61				1	1			1									1				3	
**CTC**	90				1		1								2			1	3	1	2		
**CTG**	88								1									2	1		2		
**CTT**	164		1						3									5	1		3	1	
**GAA**	324	1		3		5				1			2		1			1		1		1	
**GAC**	147		2	4								4		1		1			1				
**GAG**	266			2				1		2													
**GAT**	330		1	1	2				1	1										2	1		
**GCA**	176			1		2					2									1			
**GCC**	101		2				1				1					1							
**GCG**	59			2																			
**GCT**	202								1		3												
**GGA**	199											4				1							
**GGC**	114	1	1	1	1		1					2											
**GGG**	106	1																					
**GGT**	192			1																1			
**GTA**	85												1										
**GTC**	69		2													1				1			
**GTG**	120			1				1					1			1							
**GTT**	163									1			3								1		
**TAA**	25													1									
**TAC**	91	1			1			1				1		1							1		
**TAG**	8																						
**TAT**	141	1										1		3									
**TCA**	177	1			1										5					1			
**TCC**	97					1			1								1					2	
**TCG**	49														3								
**TCT**	261						1		2						2		1				2		
**TGA**	27																						
**TGC**	59					1							1			1							
**TGG**	89				2											3	1						
**TGT**	95	1			1					1							1	1			1		
**TTA**	115		1		1				1								3				1		
**TTC**	129				1					1				1			2				3		
**TTG**	164				1												2	1					
**TTT**	246				1	1							3				4	1					

Amino acid changes

How to read this table:
- Rows are reference amino acids and columns are changed amino acids. E.g. Row 'A' column 'E' indicates how many 'A' amino acids have been replaced by 'E' amino acids.
- Red background colors indicate that more changes happened (heat-map).
- Diagonals are indicated using grey background color
- WARNING: This table may include different translation codon tables (e.g. mamalian DNA and mitochondrial DNA).

	*	-	?	A	C	D	E	F	G	H	I	K	L	M	N	P	Q	R	S	T	V	W	Y
*****	**12**	58	2		2	1	1	1			1		7						3		1		7
**-**	138		2,146	268	103	215	330	235	297	148	230	299	426	85	232	326	254	234	598	324	252	63	146
**?**																							
**A**	1	538		**105**	11	20	23	1	26			3	7	1	2	2	1	1	7	4	18		
**C**	5	154			**20**		1	5		1	1	1	9	1	1		1	1	14	1	4	6	1
**D**	5	477		19		**54**	60	1	26	2	1	5		1	5		2	8	1	1	14		2
**E**	5	590		18		58	**111**	3	47		1	6	1			1	2	5	5	6	19	1	3
**F**	7	375		1	6	1		**73**		3	7		33		2	1	1	3	15	1	2	5	10
**G**	1	611		20		29	42	1	**150**			4	6	1	2	1	1	12	3	1	35	3	
**H**		218		2		3			2	**40**	1		16		1	15	35	8	3	3	1		1
**I**	3	344			1	4	1	1	3	1	**72**	10	4	11	29	1		9	9	21	3	1	3
**K**	10	537		1		4	7		3		11	**132**	3	7	45	3	7	18	4	18	2		
**L**	6	682		9	5		2	65	2	13	7		**148**		6	26	11	8	27	8	5	3	5
**M**	2	228		1						1	20	8	1	**19**	32		1	5	4	12	1		2
**N**	4	416		4		1	2	1	5	2	13	88	3	7	**50**		5	23	11	22	1		

	*	-	?	A	C	D	E	F	G	H	I	K	L	M	N	P	Q	R	S	T	V	W	Y
**P**	1	475		3	1	1			2	8		3	23	1	3	**172**	15	11	5	7	2		
**Q**		334		4	1	2	5	2	1	27	1		19		2	18	**61**	5	4	1			
**R**	5	402		3	2	1	3		3	7	5	23	21	5	9	5	13	**78**	21	11	1		2
**S**	12	782		3	9	1	6	58	3	2	17	21	20	3	15	9	5	38	**268**	17	6	4	10
**T**	2	408		4	1	3	6	3		2	20	15	5	6	27	3	4	12	22	**125**	1	1	2
**V**		437		21	2	25	20	3	31	1		1	3	2	2		1	3	8	1	**93**	2	1
**W**	5	89			6		1	1	1		1		8	3	2			1	10		1	**2**	1
**Y**	23	232			2		1	6		1	4	2	23		1			3	20	1	2	1	**31**

Variants by chromosome

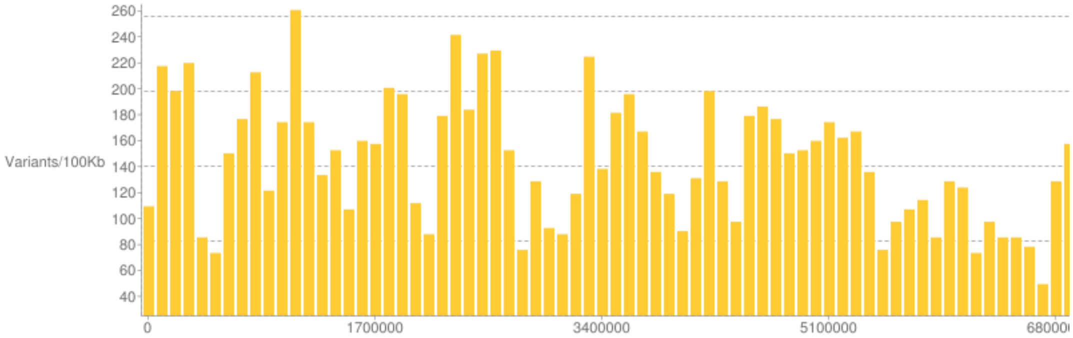

1, Position,0,100000,200000,300000,400000,500000,600000,700000,800000,900000,1000000,1100000,1200000,1300000,1400000,
1,Count,109,219,200,220,85,73,150,178,214,122,174,262,175,134,154,108,160,158,201,196,112,88,179,243,184,228,231,153,77,130,93,89,120,

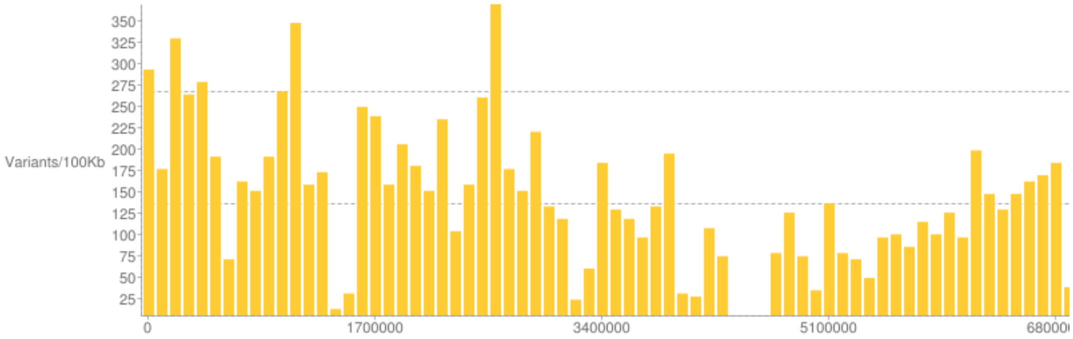

2, Position,0,100000,200000,300000,400000,500000,600000,700000,800000,900000,1000000,1100000,1200000,1300000,1400000,
2,Count,296,177,329,266,279,191,74,163,154,193,270,350,159,176,13,32,249,238,159,206,181,154,237,104,161,262,369,179,151,220,133,118,

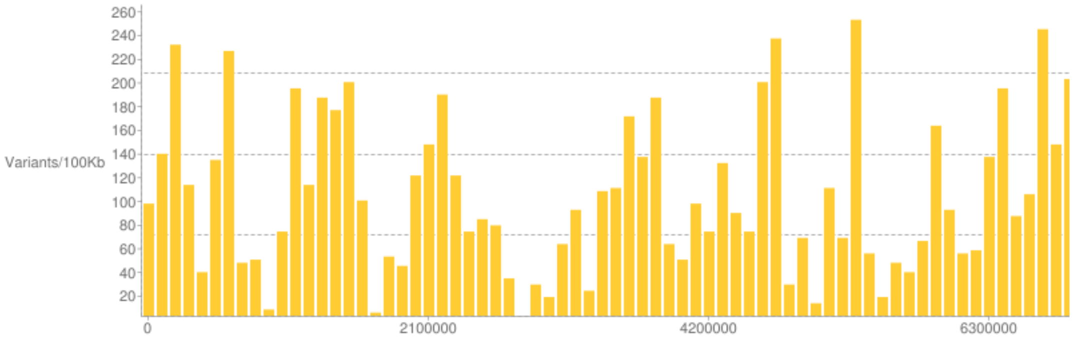

3, Position,0,100000,200000,300000,400000,500000,600000,700000,800000,900000,1000000,1100000,1200000,1300000,1400000,
3,Count,100,141,234,114,42,137,229,49,51,9,75,195,116,188,178,201,102,7,54,47,123,150,192,122,76,87,81,35,3,31,20,64,94,26,110,111,

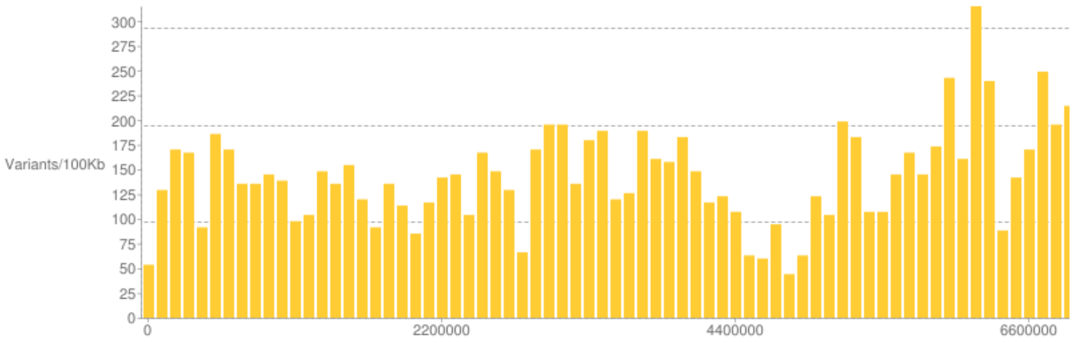

4, Position,0,100000,200000,300000,400000,500000,600000,700000,800000,900000,1000000,1100000,1200000,1300000,1400000,
4,Count,56,131,173,168,92,187,172,137,137,147,141,100,105,150,137,156,121,92,137,116,87,118,142,147,105,169,149,132,69,173,197,196,

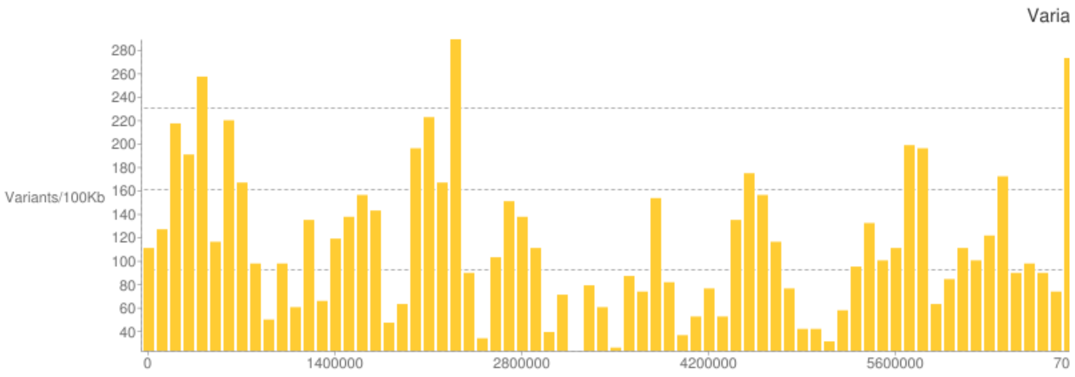

5, Position,0,100000,200000,300000,400000,500000,600000,700000,800000,900000,1000000,1100000,1200000,1300000,1400000,
5,Count,112,127,219,192,258,118,222,168,100,51,100,61,137,68,119,138,156,145,49,63,198,225,168,289,92,36,104,151,140,112,39,72,23,80,

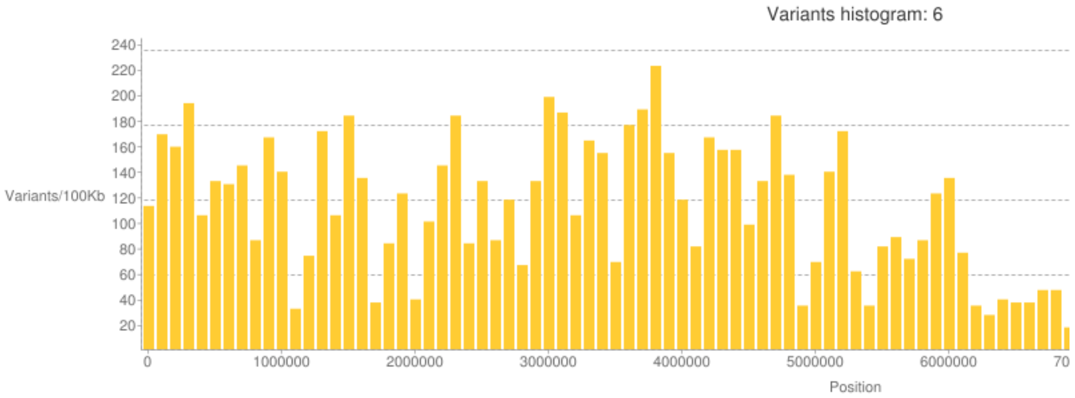

6, Position,0,100000,200000,300000,400000,500000,600000,700000,800000,900000,1000000,1100000,1200000,1300000,1400000,
6,Count,114,171,162,194,106,134,132,147,88,169,142,34,76,174,108,184,136,40,86,124,42,103,147,186,85,135,88,120,67,135,199,187,106,

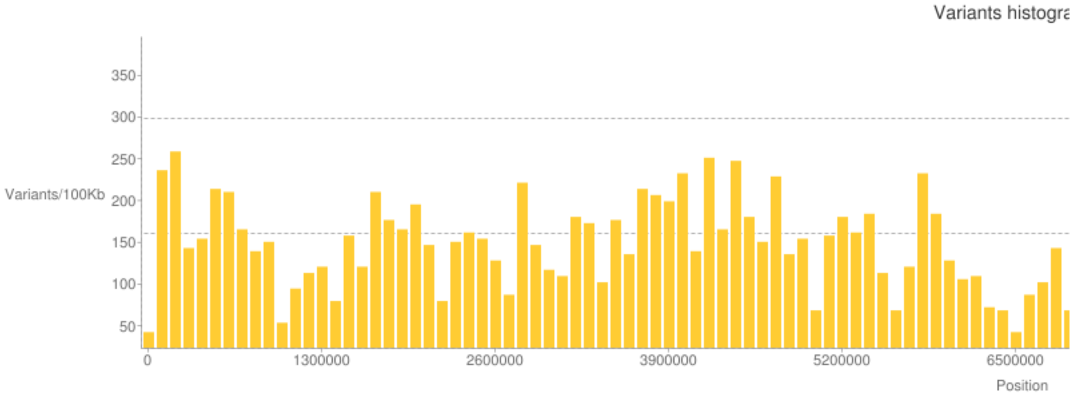

7, Position,0,100000,200000,300000,400000,500000,600000,700000,800000,900000,1000000,1100000,1200000,1300000,1400000,
7,Count,43,239,260,144,156,215,211,166,139,151,54,97,116,120,80,158,122,211,177,168,195,149,80,152,163,155,129,87,222,149,118,109,182,

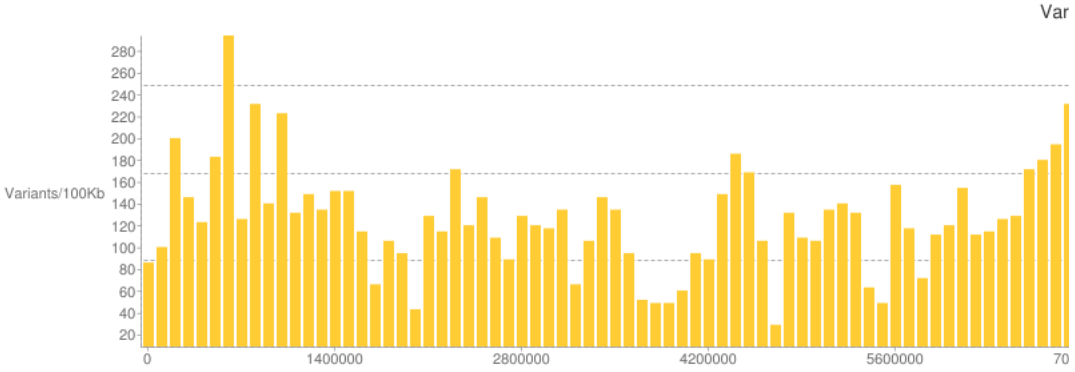

8, Position,0,100000,200000,300000,400000,500000,600000,700000,800000,900000,1000000,1100000,1200000,1300000,1400000,
8,Count,86,102,201,148,123,183,294,127,233,141,223,133,150,136,154,153,117,68,108,96,46,130,116,174,121,148,111,91,130,121,120,135,66,

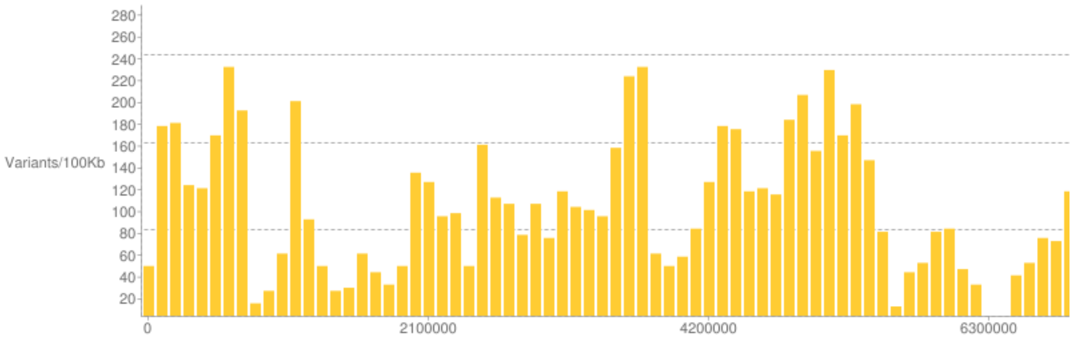

9, Position,0,100000,200000,300000,400000,500000,600000,700000,800000,900000,1000000,1100000,1200000,1300000,1400000,
9,Count,51,179,182,124,121,170,233,193,18,29,62,203,94,52,28,32,61,45,35,52,136,129,98,99,51,162,115,109,79,109,77,120,106,102,96,160,

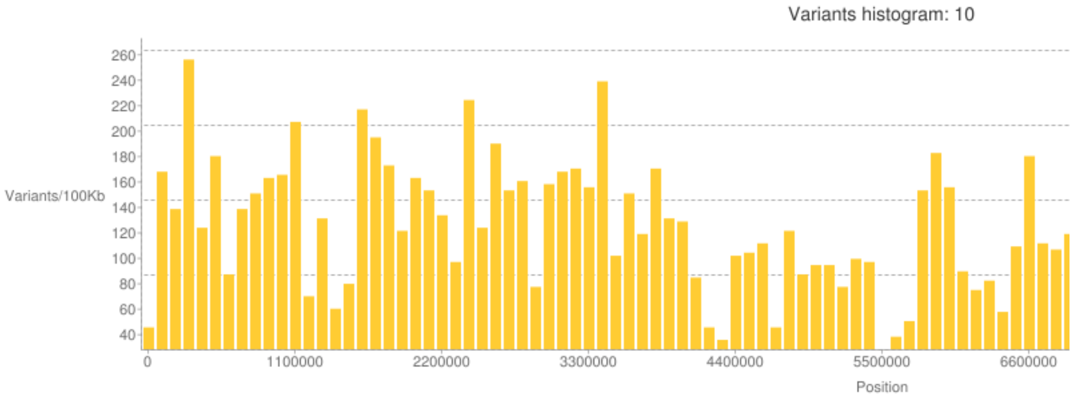

10, Position,0,100000,200000,300000,400000,500000,600000,700000,800000,900000,1000000,1100000,1200000,1300000,1400000,
10,Count,47,168,139,257,124,182,87,139,152,163,166,209,72,132,60,81,218,195,174,122,163,155,134,98,224,125,190,155,161,79,160,170,172,

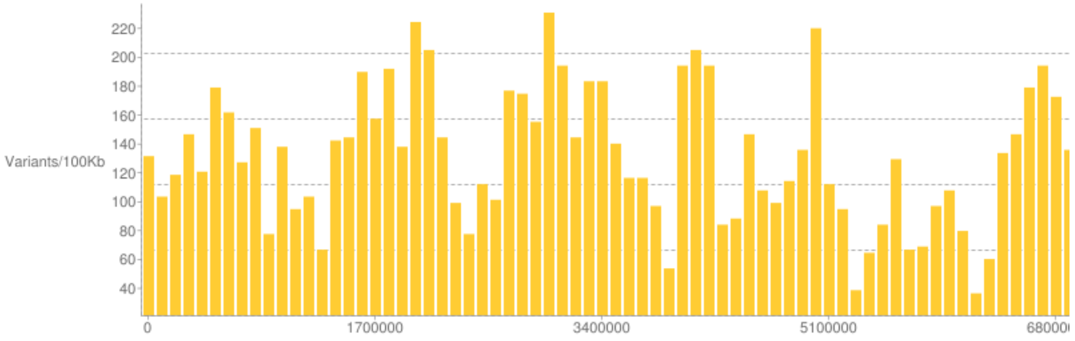

11, Position,0,100000,200000,300000,400000,500000,600000,700000,800000,900000,1000000,1100000,1200000,1300000,1400000,
11,Count,132,104,119,147,121,179,163,128,152,79,139,95,105,67,142,146,191,159,193,138,226,206,146,99,79,113,103,177,176,157,231,195,

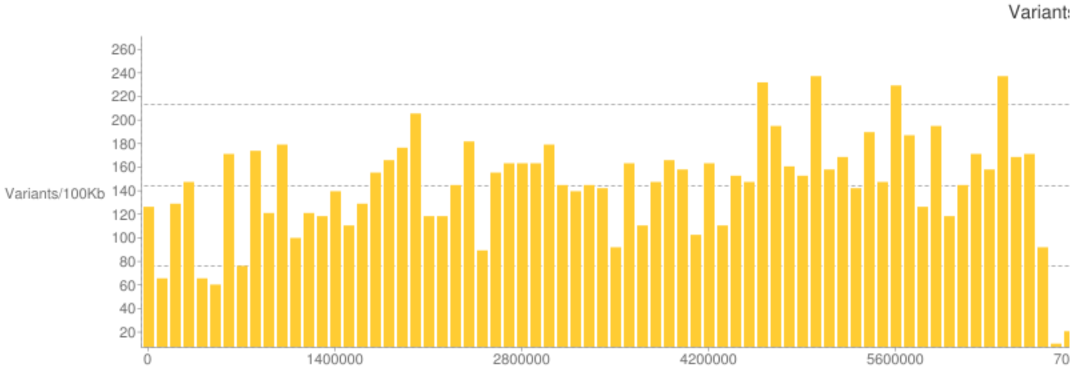

12, Position,0,100000,200000,300000,400000,500000,600000,700000,800000,900000,1000000,1100000,1200000,1300000,1400000,
12,Count,126,66,130,149,67,60,173,76,174,121,180,102,122,118,140,111,129,155,168,176,206,118,120,145,183,91,155,164,164,165,179,146,1

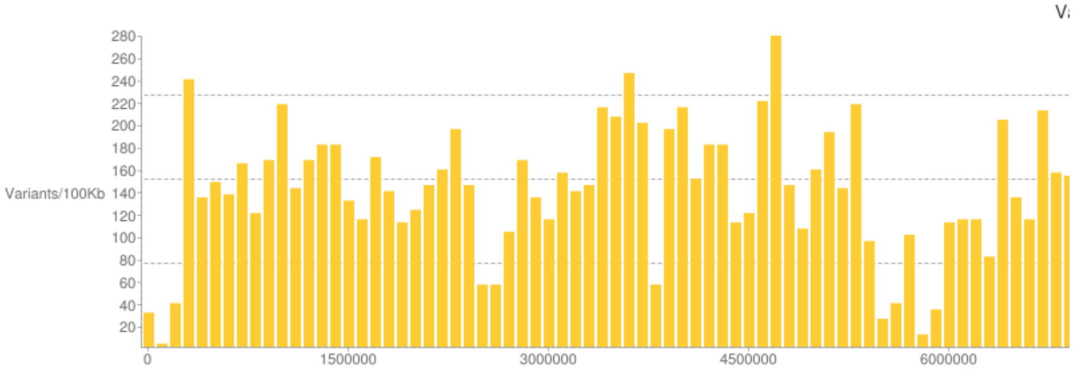

13, Position,0,100000,200000,300000,400000,500000,600000,700000,800000,900000,1000000,1100000,1200000,1300000,1400000,
13,Count,33,6,42,242,136,151,139,167,123,169,221,144,170,184,183,133,118,172,143,115,126,149,161,198,149,60,60,105,171,138,118,158,14

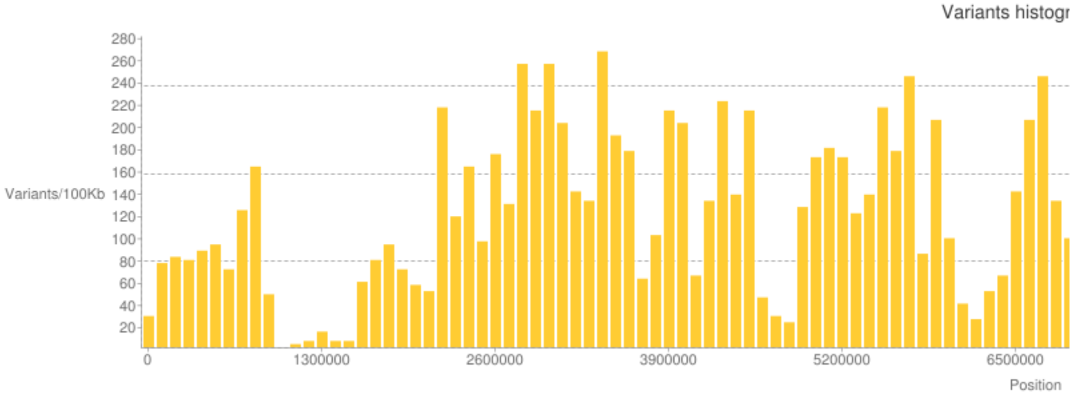

14, Position,0,100000,200000,300000,400000,500000,600000,700000,800000,900000,1000000,1100000,1200000,1300000,1400000,
14,Count,30,78,85,82,91,95,73,127,166,51,2,5,9,16,10,9,63,83,97,72,59,55,220,122,166,99,177,133,257,215,257,206,142,136,270,195,181,6

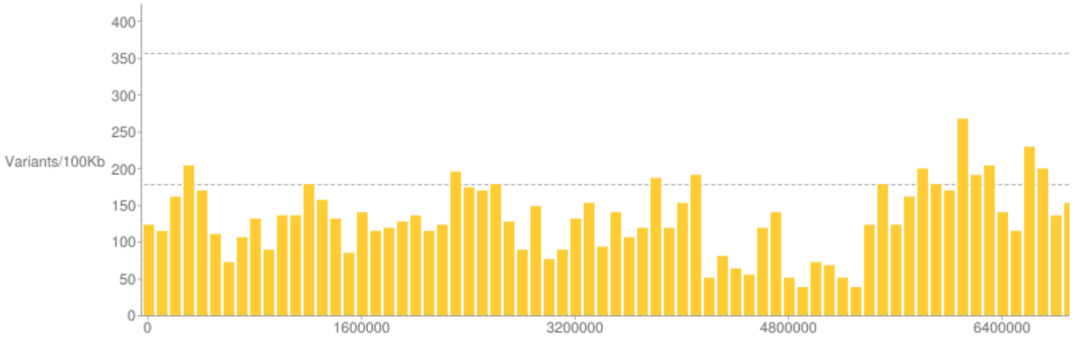

15, Position,0,100000,200000,300000,400000,500000,600000,700000,800000,900000,1000000,1100000,1200000,1300000,1400000,
15,Count,124,117,163,207,172,113,75,107,133,91,137,137,179,158,134,85,142,116,121,129,137,118,127,196,175,173,180,131,93,152,77,91,13

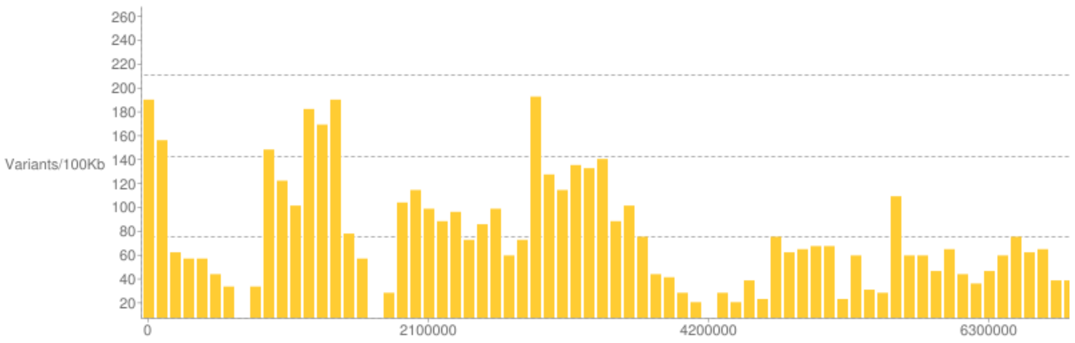

16, Position,0,100000,200000,300000,400000,500000,600000,700000,800000,900000,1000000,1100000,1200000,1300000,1400000,
16,Count,192,156,64,58,58,45,34,7,34,148,123,103,182,169,191,78,58,9,30,104,116,99,89,96,73,87,99,61,73,194,128,115,136,133,142,88,10

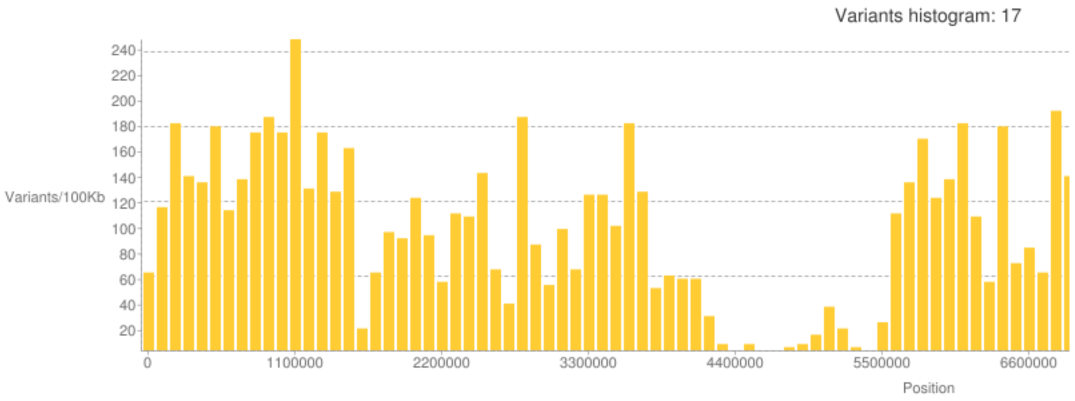

17, Position,0,100000,200000,300000,400000,500000,600000,700000,800000,900000,1000000,1100000,1200000,1300000,1400000,
17,Count,66,118,183,143,137,182,114,140,175,188,175,248,133,175,129,164,23,65,97,94,124,95,60,112,111,144,68,41,188,88,57,101,68,128,

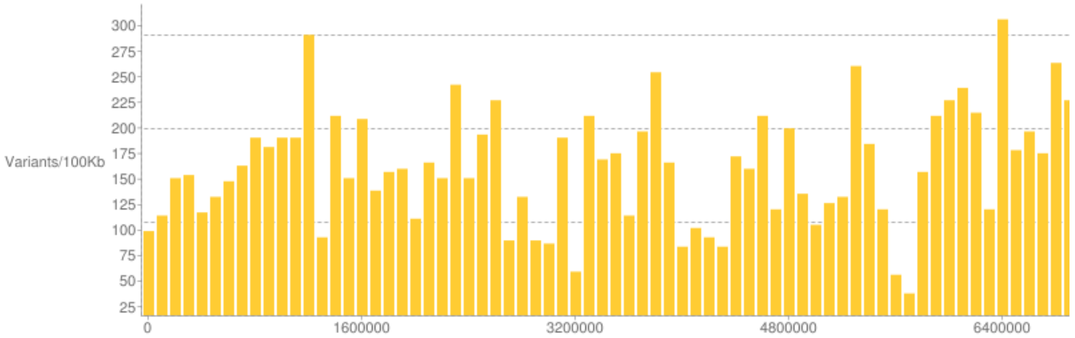

18, Position,0,100000,200000,300000,400000,500000,600000,700000,800000,900000,1000000,1100000,1200000,1300000,1400000,
18,Count,99,116,153,156,118,133,148,163,192,182,191,190,292,93,213,152,211,140,159,161,111,168,153,242,153,195,229,90,134,90,87,192,5

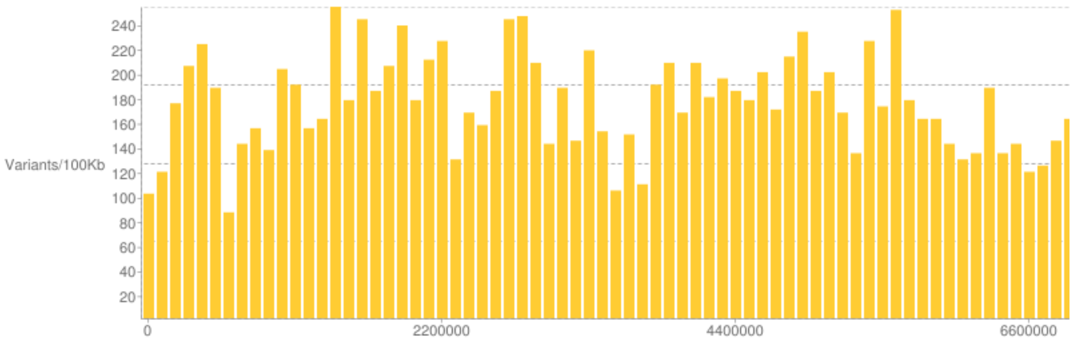

19, Position,0,100000,200000,300000,400000,500000,600000,700000,800000,900000,1000000,1100000,1200000,1300000,1400000,
19,Count,104,121,179,207,226,190,90,146,158,139,206,192,158,165,255,180,247,187,207,240,181,214,229,132,169,161,187,246,249,210,146,1

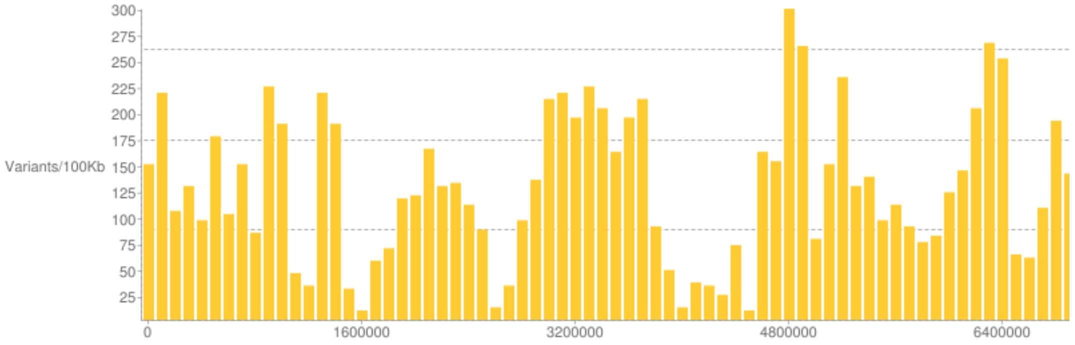

20, Position,0,100000,200000,300000,400000,500000,600000,700000,800000,900000,1000000,1100000,1200000,1300000,1400000,
20,Count,154,223,109,132,100,180,106,153,87,229,192,49,38,223,192,33,13,61,74,120,124,168,134,135,116,90,16,36,99,139,216,223,198,229,

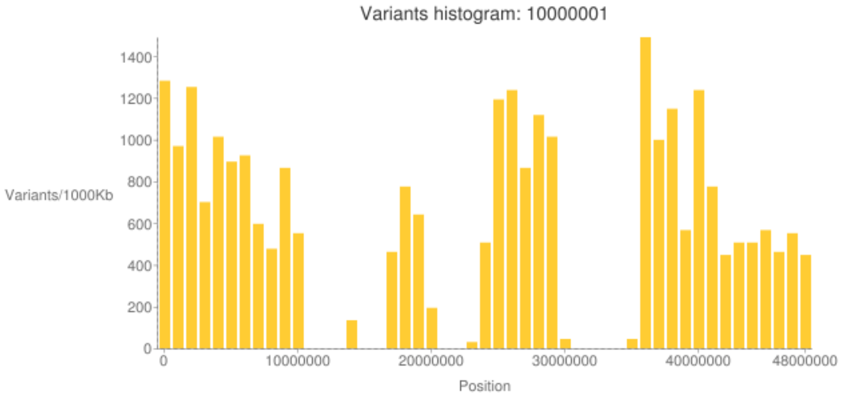

10000001, Position,0,1000000,2000000,3000000,4000000,5000000,6000000,7000000,8000000,9000000,10000000,11000000,1200000
10000001,Count,1296,980,1253,707,1018,894,928,609,491,866,564,0,0,0,142,0,0,471,778,655,206,0,0,43,521,1202,1237,870,1120,1027,46,0,0,

Details by gene

Here you can find a tab-separated table.

SnpEff: Variant analysis

Contents

Summary
Variant rate by chromosome
Variants by type
Number of variants by impact
Number of variants by functional class
Number of variants by effect
Quality histogram
InDel length histogram
Base variant table
Transition vs transversions (ts/tv)
Allele frequency
Allele Count
Codon change table
Amino acid change table
Chromosome variants plots
Details by gene

Summary

**Genome**	manindi_TA
**Date**	2021-05-22 15:11
**SnpEff version**	SnpEff 5.0e (build 2021-03-09 06:01), by Pablo Cingolani
**Command line arguments**	SnpEff manindi_TA /home/cocogenomics/mango_genome/mango_TA_genome/M.indica_TA_SNP_final.vcf
**Warnings**	174,179
**Errors**	0
**Number of lines (input file)**	2,443,689
**Number of variants (before filter)**	2,448,630
**Number of not variants (i.e. reference equals alternative)**	0
**Number of variants processed (i.e. after filter and non-variants)**	2,448,630
**Number of known variants (i.e. non-empty ID)**	0 (0%)
**Number of multi-allelic VCF entries (i.e. more than two alleles)**	4,941
**Number of effects**	4,534,925
**Genome total length**	377,290,333
**Genome effective length**	377,290,333
**Variant rate**	1 variant every 154 bases

Variants rate details

Chromosome	Length	Variants	Variants rate
1	17,320,008	137,855	125
2	17,063,873	119,444	142
3	21,566,805	134,945	159
4	22,357,487	159,826	139
5	14,540,018	92,408	157
6	10,680,009	66,922	159
7	13,133,232	101,191	129
8	14,750,018	106,451	138
9	21,055,410	136,310	154
10	11,063,414	88,246	125
11	17,675,019	127,220	138
12	14,336,529	88,122	162
13	15,099,493	102,326	147
14	13,335,999	89,792	148
15	16,178,320	100,462	161
16	21,434,198	126,181	169
17	11,746,059	78,441	149
18	16,863,820	143,927	117
19	22,398,858	147,290	152
20	16,105,987	128,223	125
10000001	48,585,777	173,048	280
**Total**	**377,290,333**	**2,448,630**	**154**

Number variants by type

Type	Total
**SNP**	2,448,630
**MNP**	0
**INS**	0
**DEL**	0
**MIXED**	0
**INV**	0
**DUP**	0
**Total**	**2,448,630**

Type	Total
BND	0
INTERVAL	0
Total	2,448,630

Number of effects by impact

Type (alphabetical order)	Count	Percent
HIGH	2,335	0.051%
LOW	67,704	1.493%
MODERATE	73,636	1.624%
MODIFIER	4,391,250	96.832%

Number of effects by functional class

Type (alphabetical order)	Count	Percent
MISSENSE	73,964	56.661%
NONSENSE	1,055	0.808%
SILENT	55,519	42.531%

Missense / Silent ratio: 1.3322

Number of effects by type and region

Type

Type (alphabetical order)	Count	Percent
3_prime_UTR_variant	36,749	0.808%
5_prime_UTR_premature_start_codon_gain_variant	2,897	0.064%
5_prime_UTR_variant	18,839	0.414%
downstream_gene_variant	968,689	21.302%
initiator_codon_variant	25	0.001%
intergenic_region	1,811,990	39.846%
intragenic_variant	35	0.001%
intron_variant	538,294	11.837%
missense_variant	73,636	1.619%
splice_acceptor_variant	491	0.011%
splice_donor_variant	486	0.011%
splice_region_variant	12,266	0.27%
start_lost	113	0.002%
stop_gained	1,055	0.023%
stop_lost	190	0.004%
stop_retained_variant	102	0.002%
synonymous_variant	55,417	1.219%
upstream_gene_variant	1,026,224	22.567%

Region

Type (alphabetical order)	Count	Percent
DOWNSTREAM	968,689	21.361%
EXON	129,297	2.851%
INTERGENIC	1,811,990	39.956%
INTRON	528,724	11.659%
SPLICE_SITE_ACCEPTOR	491	0.011%
SPLICE_SITE_DONOR	486	0.011%
SPLICE_SITE_REGION	10,504	0.232%
TRANSCRIPT	35	0.001%
UPSTREAM	1,026,224	22.629%
UTR_3_PRIME	36,749	0.81%
UTR_5_PRIME	21,736	0.479%

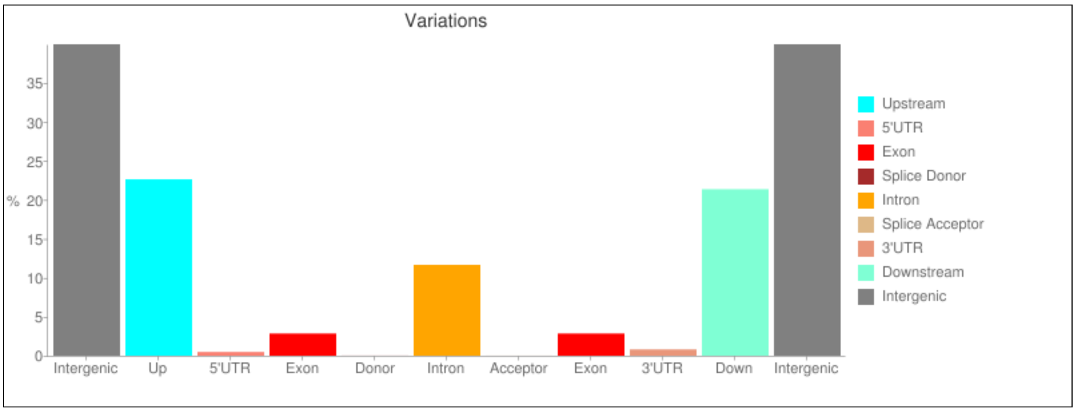

Quality:

**Min**	10
**Max**	26,279
**Mean**	140.176
**Median**	110
**Standard deviation**	252.376
**Values**	10,11,12,13,14,15,16,17,18,19,20,21,22,23,24,25,26,27,28,29,30,31,32,33,34,35,36,37,38,39,40,41,42,43,44,45,46,47,48,49,50,51,52,53,54,55,56,57
**Count**	3221,1955,11616,2134,3069,3635,6029,7133,13455,11202,13685,15815,18977,12847,20984,23166,14003,12115,15985,8317,7443,13688,10278,86[illegible]

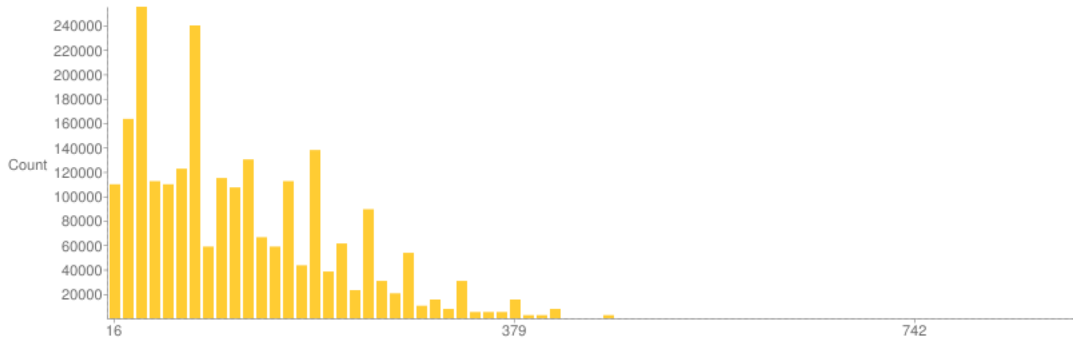

Insertions and deletions length:

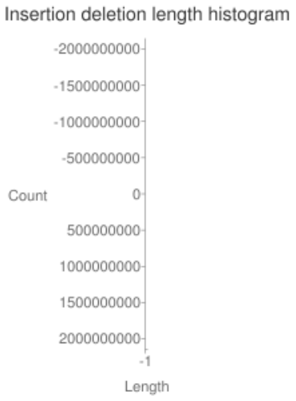

Base changes (SNPs)

	A	C	G	T
**A**	0	84,097	410,796	125,158
**C**	91,820	0	57,180	454,086
**G**	455,425	57,138	0	91,956
**T**	125,412	411,349	84,213	0

Ts/Tv (transitions / transversions)

Note: Only SNPs are used for this statistic.
Note: This Ts/Tv ratio is a 'raw' ratio (ratio of observed events).

**Transitions**	2,408,525
**Transversions**	991,782
**Ts/Tv ratio**	2.4285

All variants:

```
Sample ,readname,Total
Transitions ,2408525,2408525
Transversions ,991782,991782
Ts/Tv ,2.428,2.428
```

Only known variants (i.e. the ones having a non-empty ID field):

```
No results available (empty input?)
```

Allele frequency

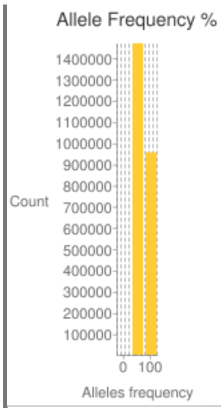

**Min**	0
**Max**	100
**Mean**	69.775
**Median**	50
**Standard deviation**	24.586
**Values**	0,50,100
**Count**	3307,1470575,969807

Allele Count

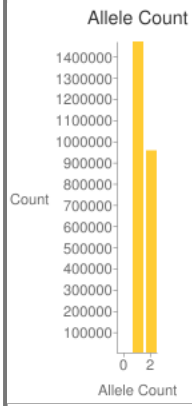

**Min**	0
**Max**	2
**Mean**	1.396
**Median**	1
**Standard deviation**	0.492
**Values**	0,1,2
**Count**	3307,1470575,969807

Hom/Het per sample

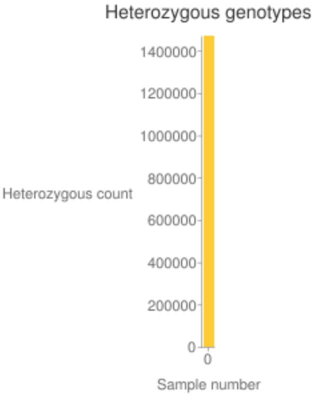

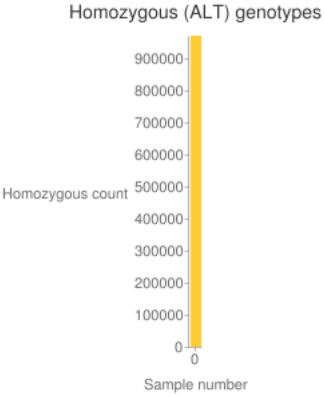

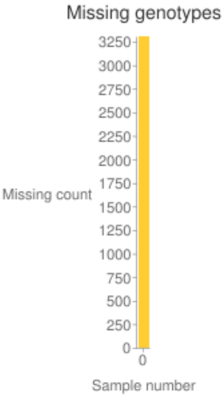


```
Sample_names , readname
Reference , 0
Het , 1470575
Hom , 969807
Missing , 3307
```

Codon changes

How to read this table:
- Rows are reference codons and columns are changed codons. E.g. Row 'AAA' column 'TAA' indicates how many 'AAA' codons have been replaced by 'TAA' codons.
- Red background colors indicate that more changes happened (heat-map).
- Diagonals are indicated using grey background color
- WARNING: This table may include different translation codon tables (e.g. mamalian DNA and mitochondrial DNA).

	AAA	AAC	AAG	AAT	ACA	ACC	ACG	ACT	AGA	AGC	AGG	AGT	ATA	ATC	ATG	ATT	CAA	CAC	CAG	CAT	CCA	CCC	[illegible]
**AAA**		130	872	258	182				514				112				238						
**AAC**	202		177	1,006		112				369				68				72					
**AAG**	1,014	137		203			75				451				160				182				
**AAT**	223	746	217					163				508				162				189			
**ACA**	179					150	567	309	161				394								85		
**ACC**		125			170		93	822		127				172								46	
**ACG**			123		818	119		207			69				448								
**ACT**				215	304	584	129					208				448							
**AGA**	534				127					71	419	91	121										
**AGC**		432				104			83		80	639		78									
**AGG**			500				52		504	82		117			93								
**AGT**				613				237	88	522	95					158							
**ATA**	105				288				92					199	415	307							
**ATC**		57				144				55			230		134	843							
**ATG**			156				335				98		530	134		237							
**ATT**				172				377				111	285	748	186								
**CAA**	249																	84	689	137	96		

	AAA	AAC	AAG	AAT	ACA	ACC	ACG	ACT	AGA	AGC	AGG	AGT	ATA	ATC	ATG	ATT	CAA	CAC	CAG	CAT	CCA	CCC
**CAC**		87															87		126	554		26
**CAG**			159														739	91		142		
**CAT**				200													185	396	122			
**CCA**					94												155					135
**CCC**						41												39			160	
**CCG**							34												94		973	102
**CCT**								115												92	290	450
**CGA**									191								564				68	
**CGC**										41								219				41
**CGG**											149								474			
**CGT**												72								575		
**CTA**													108				105				190	
**CTC**														87				34				85
**CTG**															143				92			
**CTT**																205				105		
**GAA**	784																269					
**GAC**		374																60				
**GAG**			635																245			
**GAT**				785																165		
**GCA**					653																131	
**GCC**						329																48
**GCG**							146															
**GCT**								648														
**GGA**									364													
**GGC**										283												
**GGG**											243											
**GGT**												570										
**GTA**													504									
**GTC**														395								
**GTG**															460							
**GTT**																923						
**TAA**	8																18					
**TAC**		72																158				
**TAG**			7																23			
**TAT**				171																353		
**TCA**					165																308	
**TCC**						68																132
**TCG**							30															
**TCT**								190														
**TGA**									5													
**TGC**										49												
**TGG**											73											
**TGT**												93										
**TTA**													105									
**TTC**														92								
**TTG**															216							
**TTT**																205						

Amino acid changes

How to read this table:
- Rows are reference amino acids and columns are changed amino acids. E.g. Row 'A' column 'E' indicates how many 'A' amino acids have been replaced by 'E' amino acids.
- Red background colors indicate that more changes happened (heat-map).
- Diagonals are indicated using grey background color
- WARNING: This table may include different translation codon tables (e.g. mamalian DNA and mitochondrial DNA).

	*	A	C	D	E	F	G	H	I	K	L	M	N	P	Q	R	S	T	V	W	Y
*****	**102**		9		17		8			15	15				41	25	16			25	19
**A**		**5,205**		203	308		488							340			655	1,776	1,715		
**C**	41		**904**			176	101									449	361			74	420
**D**		171		**2,068**	1,070		540	225					1,159						154		275
**E**	91	240		941	**1,783**		582			1,419					514				228		
**F**			159			**1,785**			297		1,154						481		248		353
**G**	27	473	183	659	707		**3,928**									826	853		354	59	
**H**				235				**950**			156		287	103	520	547					548
**I**						277			**2,612**	105	481	735	229			92	166	809	1,421		
**K**	74				1,203				112	**1,886**		160	728		420	965		257			
**L**	76					1,306		139	505		**8,377**	359		696	197	196	762		833	83	
**M**									901	156	353					98		335	391		
**N**				955				261	230	819			**1,752**				877	275			237
**P**		360						131			924			**4,180**	249	169	973	284			
**Q**	219				474			454		408	209			144	**1,428**	784					
**R**	149		572				716	794	121	1,034	231	93		213	1,038	**2,911**	474	179		274	

	*	A	C	D	E	F	G	H	I	K	L	M	N	P	Q	R	S	T	V	W	Y
**S**	96	561	433			565	677		236		900		1,045	847		475	**6,098**	794		53	216
**T**		1,437							1,014	302		448	340	258		230	757	**4,272**			
**V**		1,434		135	201	336	301		1,822		899	460							**4,033**		
**W**	167		98				43				92					262	44				
**Y**	115		377	209		320		511					243				187				**1,245**

Variants by chromosome

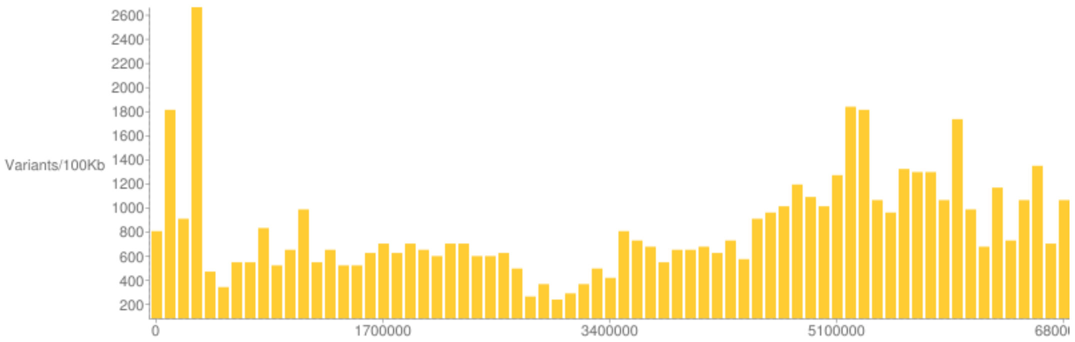

1, Position,0,100000,200000,300000,400000,500000,600000,700000,800000,900000,1000000,1100000,1200000,1300000,1400000,[illegible]
1,Count,811,1822,907,2661,487,349,557,548,829,544,649,997,550,667,541,534,638,706,626,702,660,608,725,700,620,618,644,515,265,366,260,[illegible]

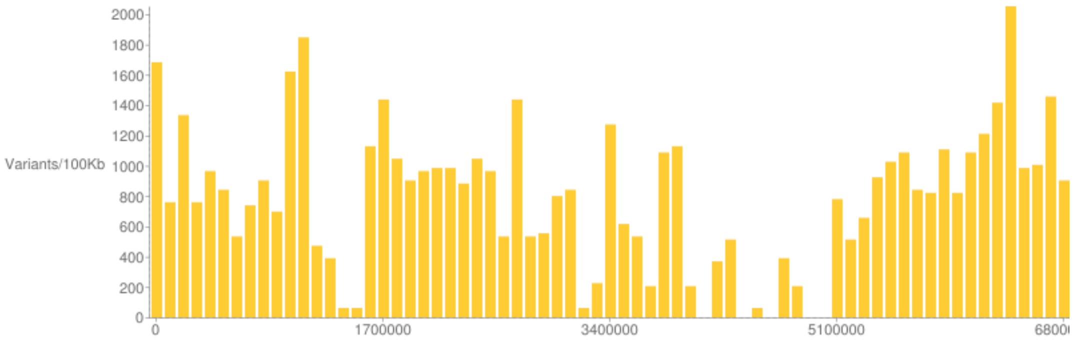

2, Position,0,100000,200000,300000,400000,500000,600000,700000,800000,900000,1000000,1100000,1200000,1300000,1400000,[illegible]
2,Count,1687,765,1336,779,979,852,541,745,914,698,1632,1861,478,408,71,82,1148,1439,1063,906,984,991,1001,890,1050,970,536,1451,538,5[illegible]

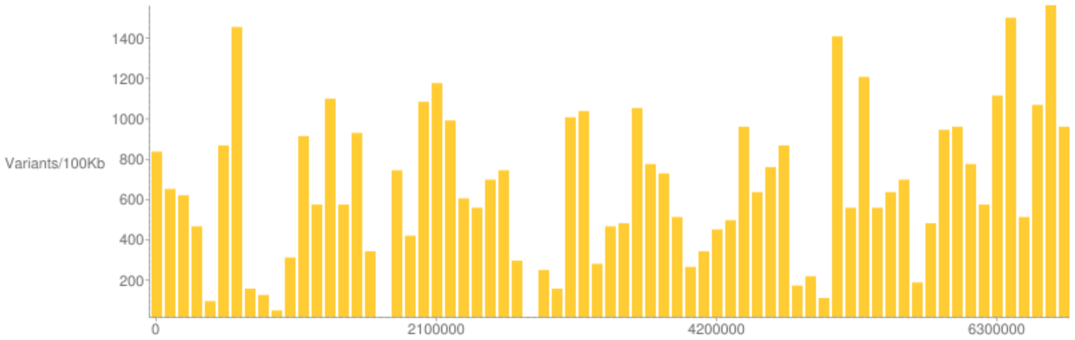

3, Position,0,100000,200000,300000,400000,500000,600000,700000,800000,900000,1000000,1100000,1200000,1300000,1400000,[illegible]
3,Count,844,654,620,474,94,871,1458,161,139,54,316,912,574,1098,587,929,344,16,746,420,1095,1175,1004,614,559,708,752,306,18,256,161,[illegible]

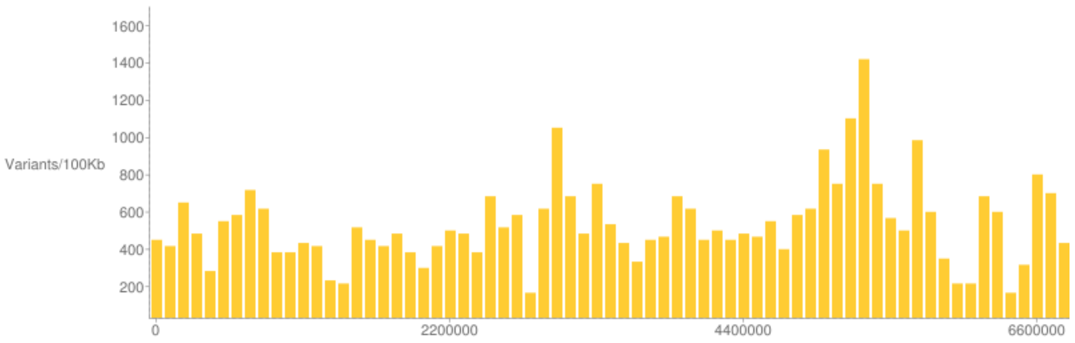

4, Position,0,100000,200000,300000,400000,500000,600000,700000,800000,900000,1000000,1100000,1200000,1300000,1400000,
4,Count,457,424,661,495,294,562,598,717,631,386,394,442,421,245,219,529,459,415,483,387,305,417,500,490,396,691,521,582,178,622,1061,

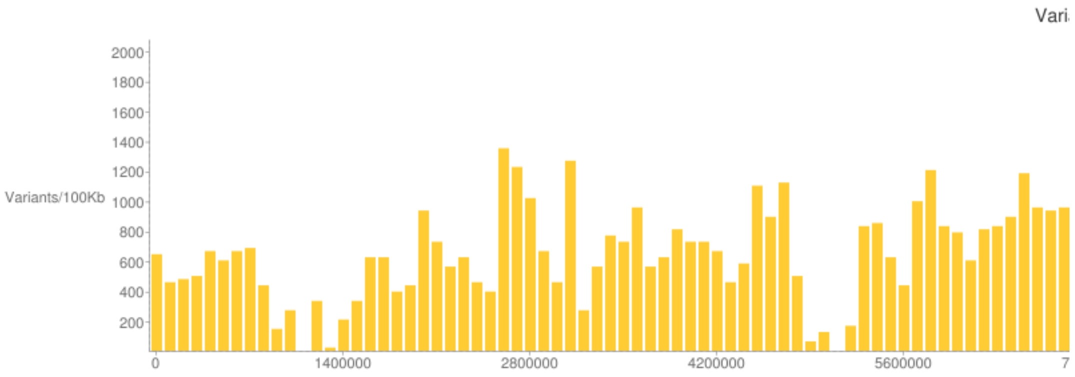

5, Position,0,100000,200000,300000,400000,500000,600000,700000,800000,900000,1000000,1100000,1200000,1300000,1400000,
5,Count,661,476,494,519,689,616,675,700,451,167,276,25,348,44,213,345,633,636,418,454,940,741,584,648,473,419,1375,1243,1026,685,471,

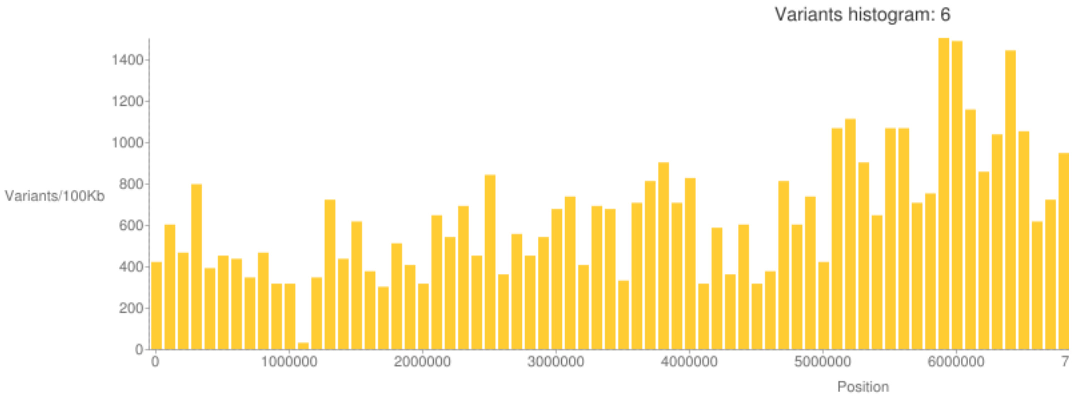

6, Position,0,100000,200000,300000,400000,500000,600000,700000,800000,900000,1000000,1100000,1200000,1300000,1400000,
6,Count,428,610,479,797,405,455,445,356,472,324,324,36,350,730,445,616,390,312,511,417,322,655,552,692,458,852,371,566,454,541,677,74

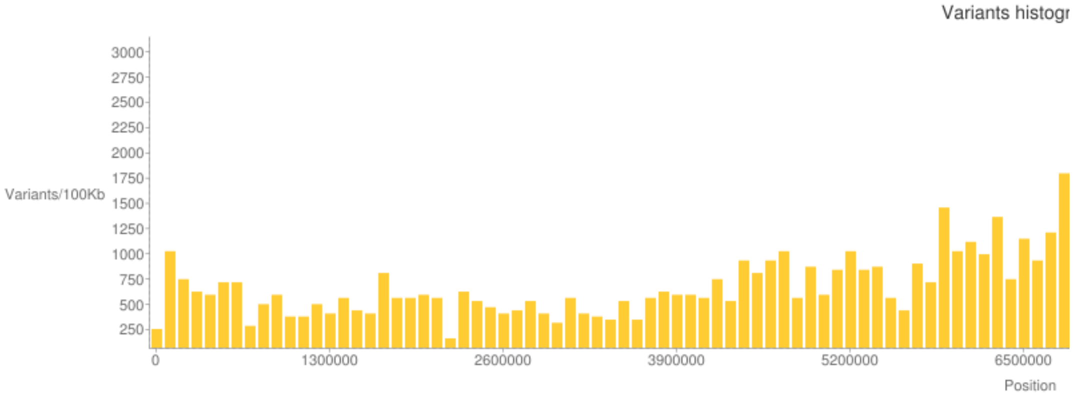

7, Position,0,100000,200000,300000,400000,500000,600000,700000,800000,900000,1000000,1100000,1200000,1300000,1400000,
7,Count,272,1044,767,633,593,726,736,298,504,614,385,398,497,434,561,440,432,817,573,562,599,558,178,623,555,468,433,462,529,425,330,

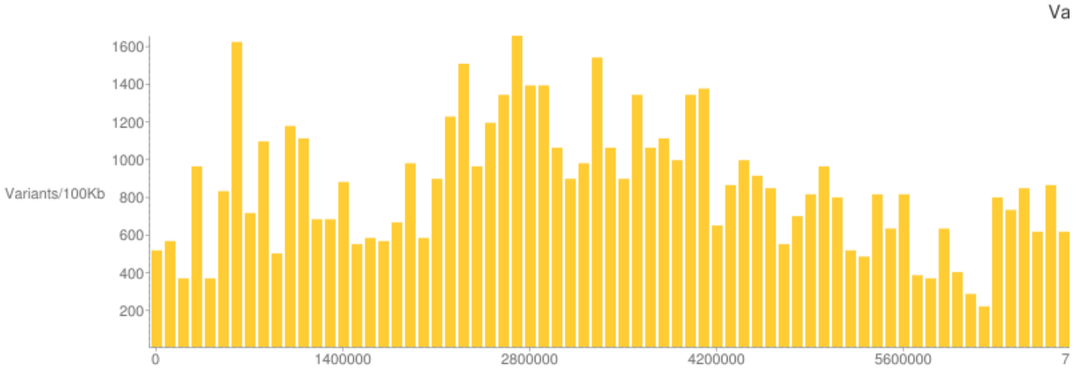

8, Position,0,100000,200000,300000,400000,500000,600000,700000,800000,900000,1000000,1100000,1200000,1300000,1400000,
8,Count,516,567,381,965,370,840,1623,723,1100,512,1191,1119,681,693,891,549,588,565,665,979,586,911,1231,1513,965,1197,1345,1654,1401,

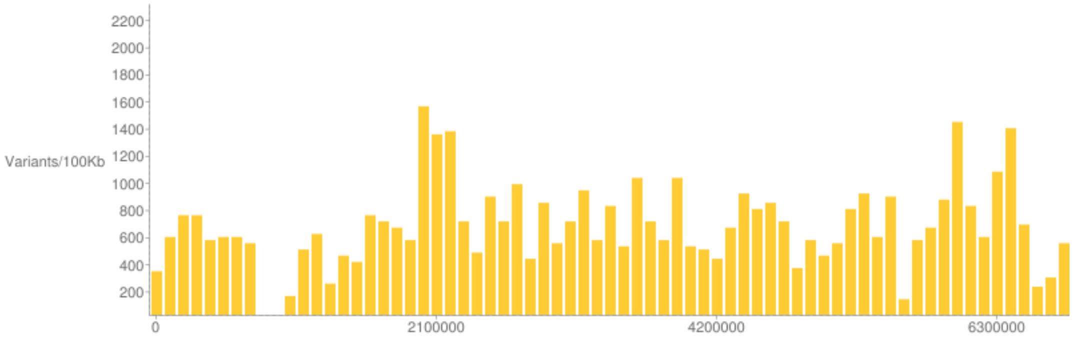

9, Position,0,100000,200000,300000,400000,500000,600000,700000,800000,900000,1000000,1100000,1200000,1300000,1400000,
9,Count,367,622,782,775,591,602,622,573,27,36,185,526,626,267,464,429,778,733,685,600,1572,1378,1399,725,506,908,729,1012,440,857,573,

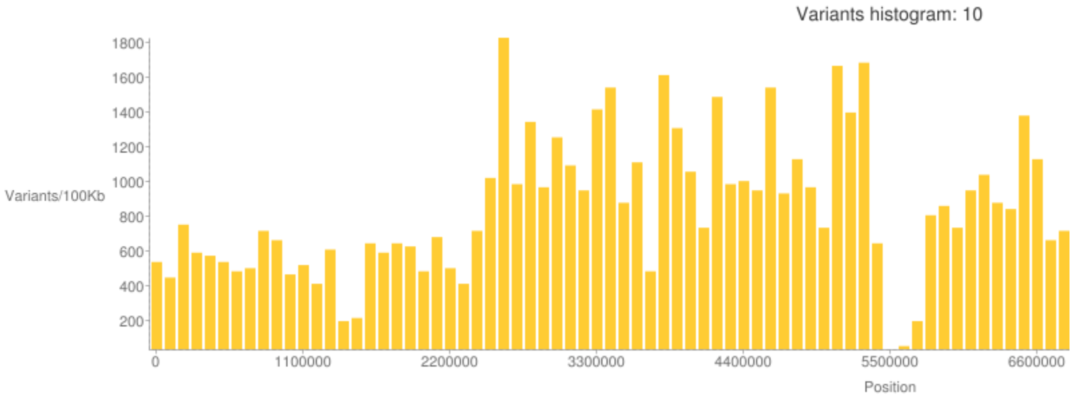

10, Position,0,100000,200000,300000,400000,500000,600000,700000,800000,900000,1000000,1100000,1200000,1300000,1400000,
10,Count,536,461,761,594,582,547,498,515,724,671,478,528,418,619,202,212,653,593,658,639,492,683,513,417,723,1035,1825,999,1351,972,

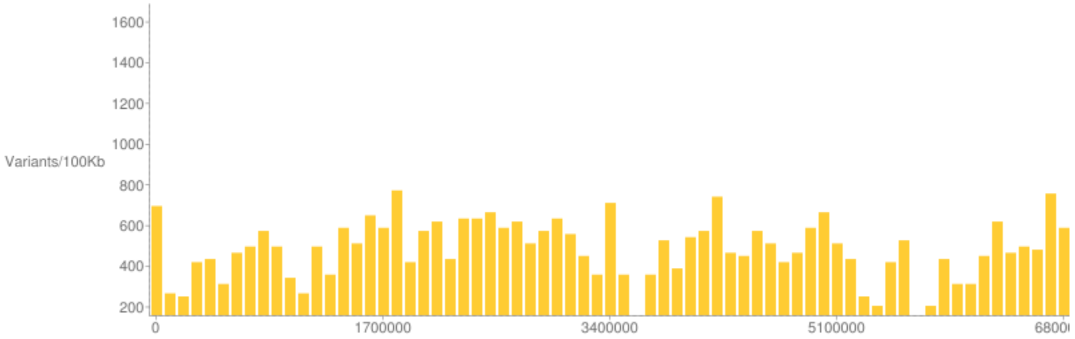

11, Position,0,100000,200000,300000,400000,500000,600000,700000,800000,900000,1000000,1100000,1200000,1300000,1400000,
11,Count,703,271,255,421,440,315,472,505,574,504,354,274,495,368,599,514,656,589,782,430,576,623,440,642,632,665,601,619,519,571,633,

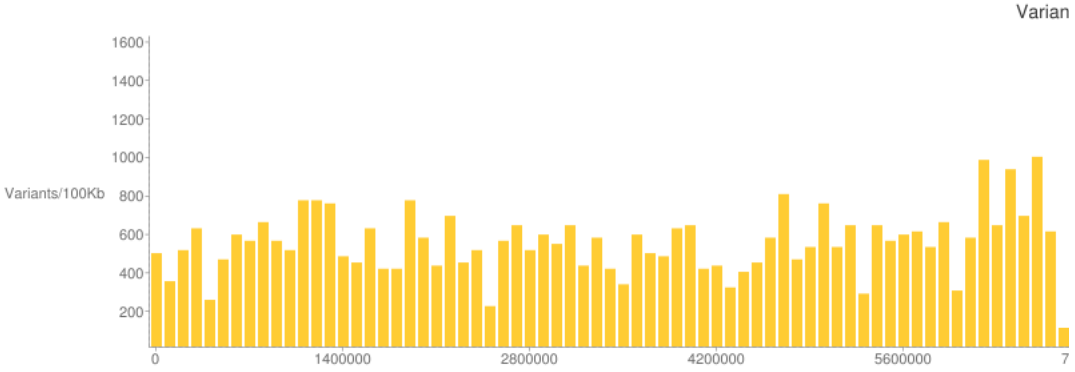

12, Position,0,100000,200000,300000,400000,500000,600000,700000,800000,900000,1000000,1100000,1200000,1300000,1400000,
12,Count,502,359,529,640,264,472,605,569,662,570,530,779,782,761,494,453,637,424,432,777,585,450,698,458,528,239,567,653,519,602,556,

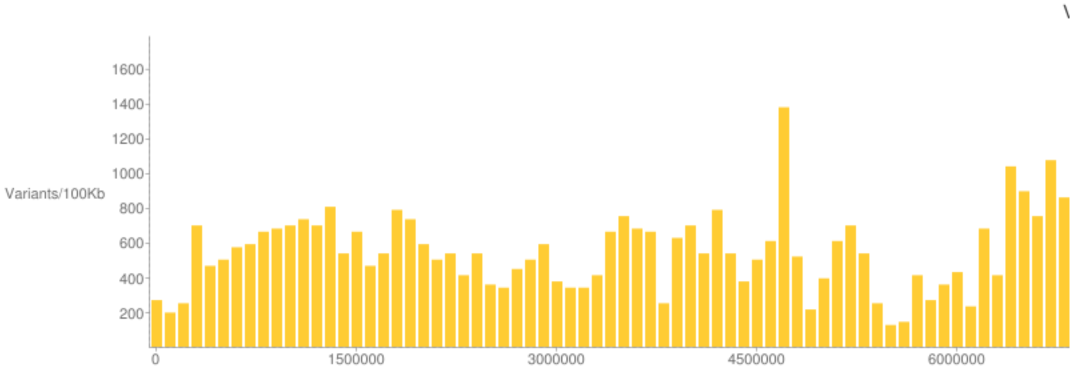

13, Position,0,100000,200000,300000,400000,500000,600000,700000,800000,900000,1000000,1100000,1200000,1300000,1400000,
13,Count,286,199,264,716,474,513,590,609,664,694,716,741,716,809,542,672,474,549,793,746,609,509,546,422,546,363,348,452,511,603,378,

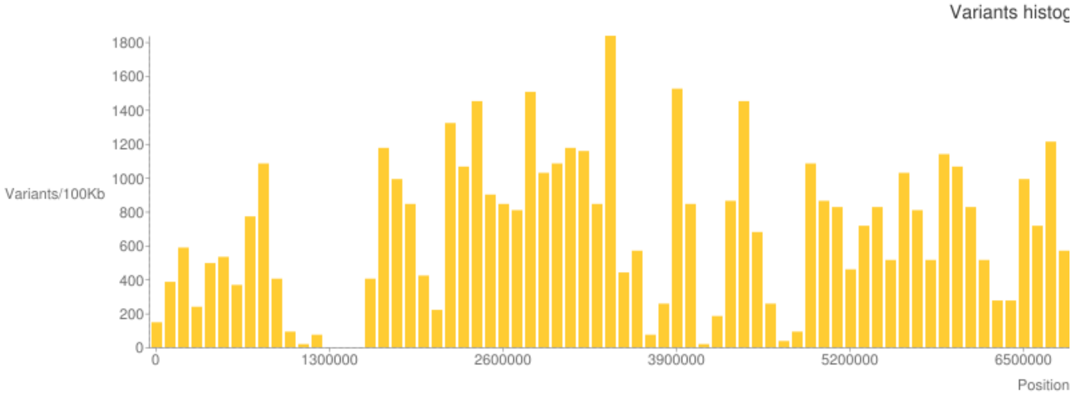

14, Position,0,100000,200000,300000,400000,500000,600000,700000,800000,900000,1000000,1100000,1200000,1300000,1400000,
14,Count,154,400,602,243,501,538,380,772,1093,408,98,34,77,0,6,5,420,1191,1008,850,430,225,1322,1081,1456,901,854,824,1515,1032,1092,

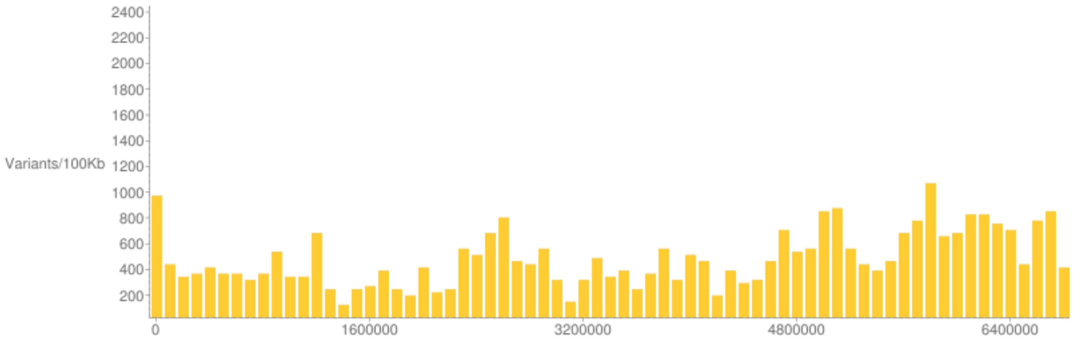

15, Position,0,100000,200000,300000,400000,500000,600000,700000,800000,900000,1000000,1100000,1200000,1300000,1400000,
15,Count,984,450,351,374,436,380,372,336,371,538,351,349,690,248,145,248,272,396,260,211,430,230,246,582,533,690,803,466,438,572,339,

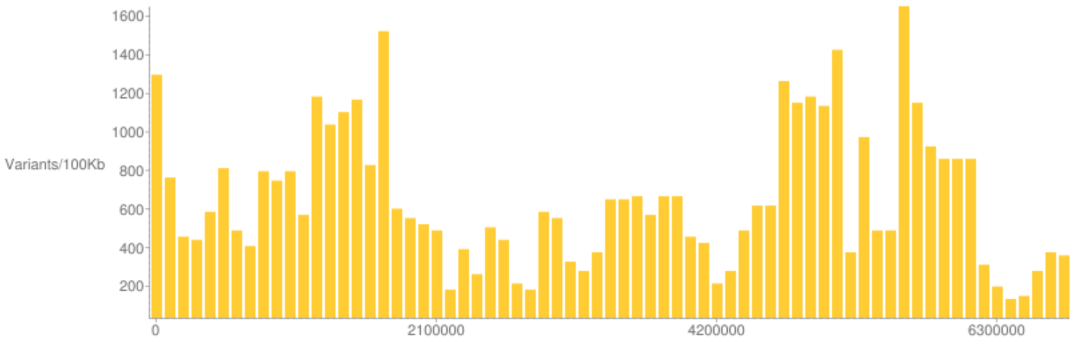

16, Position,0,100000,200000,300000,400000,500000,600000,700000,800000,900000,1000000,1100000,1200000,1300000,1400000,
16,Count,1294,761,458,447,588,811,490,414,799,751,802,577,1182,1042,1106,1168,836,1524,603,566,524,496,187,390,263,514,452,215,180,58[illegible]

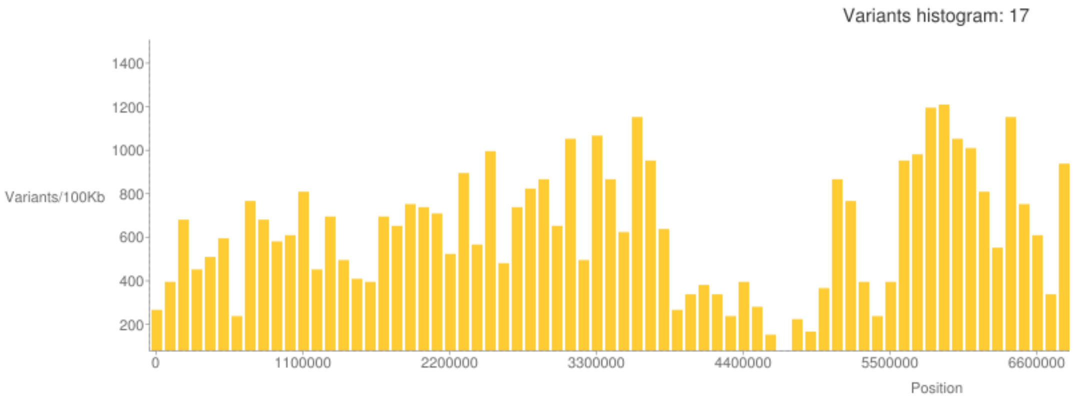

17, Position,0,100000,200000,300000,400000,500000,600000,700000,800000,900000,1000000,1100000,1200000,1300000,1400000,
17,Count,278,399,692,452,514,596,247,778,691,592,617,821,460,699,493,408,406,696,663,755,742,721,526,897,573,1006,484,749,832,868,661,

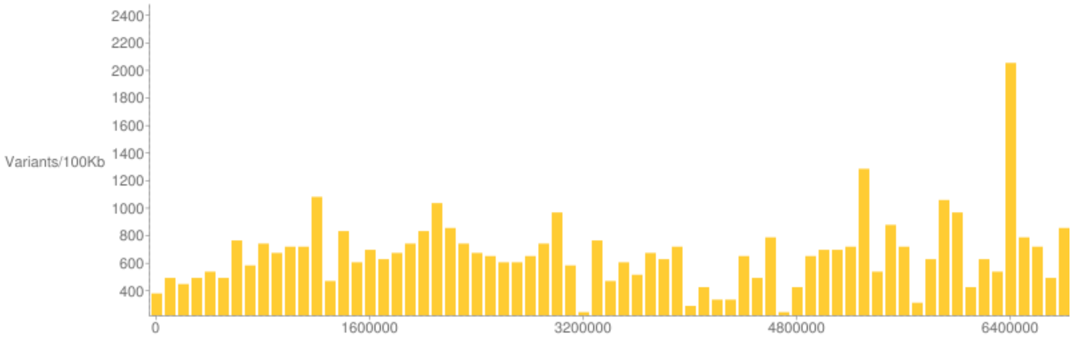

18, Position,0,100000,200000,300000,400000,500000,600000,700000,800000,900000,1000000,1100000,1200000,1300000,1400000,
18,Count,383,498,456,506,537,509,764,592,739,681,719,721,1083,468,842,616,706,641,672,754,841,1038,869,749,674,655,620,605,653,753,97[illegible]

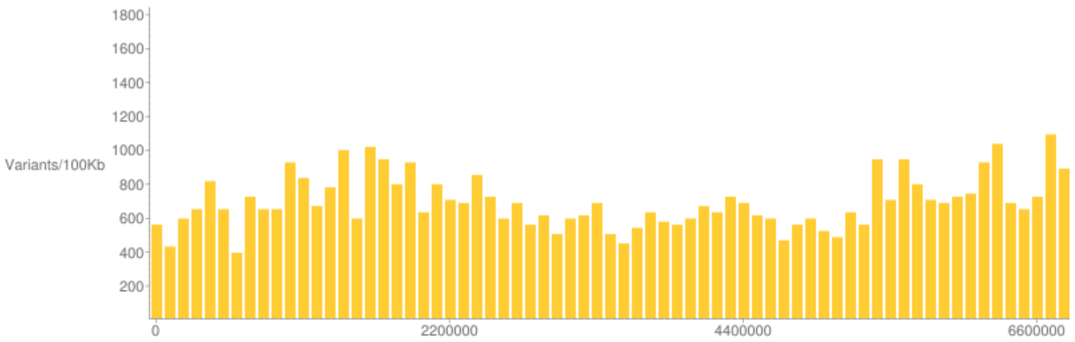

19, Position,0,100000,200000,300000,400000,500000,600000,700000,800000,900000,1000000,1100000,1200000,1300000,1400000,
19,Count,559,430,606,659,820,650,405,730,665,665,936,849,671,786,1012,599,1017,955,801,927,649,808,719,700,866,739,612,690,576,618,51[illegible]

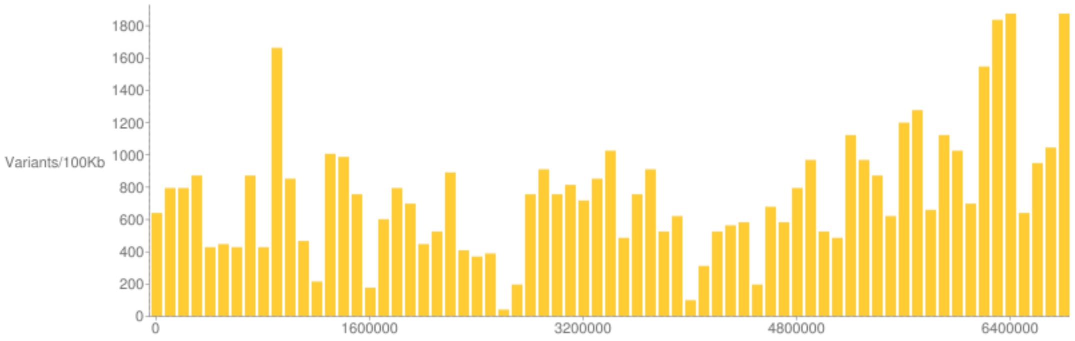

20, Position,0,100000,200000,300000,400000,500000,600000,700000,800000,900000,1000000,1100000,1200000,1300000,1400000,
20,Count,649,796,810,874,426,449,434,883,433,1673,860,470,218,1011,999,763,180,604,799,712,446,533,904,418,381,387,48,200,763,923,768,

Variants histogram: 10000001

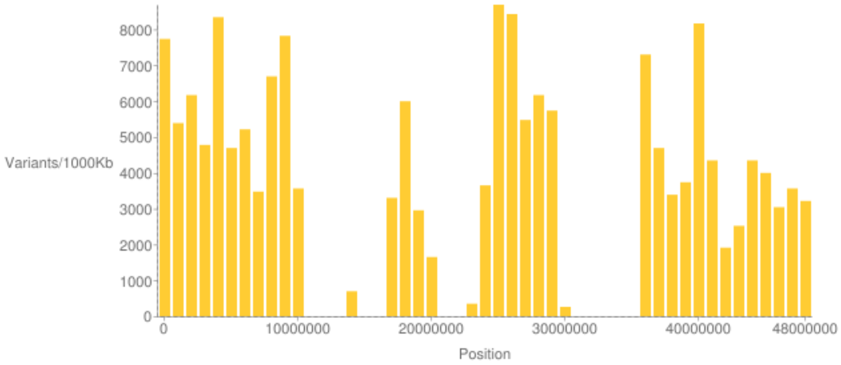

10000001, Position,0,1000000,2000000,3000000,4000000,5000000,6000000,7000000,8000000,9000000,10000000,11000000,120000[illegible]
10000001,Count,7757,5427,6223,4801,8401,4707,5227,3546,6755,7856,3580,0,0,0,737,0,0,3348,5999,2961,1736,0,0,350,3725,8687,8470,5538,6[illegible]

Details by gene

Here you can find a tab-separated table.

SnpEff: Variant analysis

Contents

Summary
Variant rate by chromosome
Variants by type
Number of variants by impact
Number of variants by functional class
Number of variants by effect
Quality histogram
InDel length histogram
Base variant table
Transition vs transversions (ts/tv)
Allele frequency
Allele Count
Codon change table
Amino acid change table
Chromosome variants plots
Details by gene

Summary

**Genome**	manindi_TA
**Date**	2021-05-22 15:23
**SnpEff version**	`SnpEff 5.0e (build 2021-03-09 06:01), by Pablo Cingolani`
**Command line arguments**	`SnpEff manindi_TA /home/cocogenomics/mango_genome/mango_TA_genome/M.indica_TA_INDEL_final.vcf`
**Warnings**	40,633
**Errors**	0
**Number of lines (input file)**	403,126
**Number of variants (before filter)**	403,850
**Number of not variants (i.e. reference equals alternative)**	0
**Number of variants processed (i.e. after filter and non-variants)**	403,850
**Number of known variants (i.e. non-empty ID)**	0 (0%)
**Number of multi-allelic VCF entries (i.e. more than two alleles)**	724
**Number of effects**	829,337
**Genome total length**	377,290,333
**Genome effective length**	377,290,333
**Variant rate**	1 variant every 934 bases

Variants rate details

Chromosome	Length	Variants	Variants rate
1	17,320,008	23,627	733
2	17,063,873	20,680	825
3	21,566,805	23,066	935
4	22,357,487	26,973	828
5	14,540,018	15,911	913
6	10,680,009	9,826	1,086
7	13,133,232	16,447	798
8	14,750,018	18,072	816
9	21,055,410	22,037	955
10	11,063,414	13,733	805
11	17,675,019	20,343	868
12	14,336,529	15,217	942
13	15,099,493	16,827	897
14	13,335,999	15,665	851
15	16,178,320	16,007	1,010
16	21,434,198	22,493	952
17	11,746,059	12,529	937
18	16,863,820	24,019	702
19	22,398,858	25,803	868
20	16,105,987	19,555	823
10000001	48,585,777	25,020	1,941
**Total**	**377,290,333**	**403,850**	**934**

Number variants by type

Type	Total
**SNP**	0
**MNP**	0
**INS**	197,129
**DEL**	206,721
**MIXED**	0
**INV**	0
**DUP**	0
**Total**	**403,850**

Type	Total
BND	0
INTERVAL	0
Total	403,850

Number of effects by impact

Type (alphabetical order)	Count	Percent
HIGH	4,780	0.576%
LOW	1,949	0.235%
MODERATE	2,562	0.309%
MODIFIER	820,046	98.88%

Number of effects by functional class

Type (alphabetical order)	Count	Percent

Missense / Silent ratio: 0

Number of effects by type and region

Type

Type (alphabetical order)	Count	Percent
3_prime_UTR_variant	8,874	1.066%
5_prime_UTR_truncation	3	0%
5_prime_UTR_variant	5,265	0.633%
bidirectional_gene_fusion	4	0%
conservative_inframe_deletion	502	0.06%
conservative_inframe_insertion	564	0.068%
disruptive_inframe_deletion	907	0.109%
disruptive_inframe_insertion	669	0.08%
downstream_gene_variant	194,277	23.34%
exon_loss_variant	4	0%
frameshift_variant	4,371	0.525%
intergenic_region	296,065	35.569%
intragenic_variant	17	0.002%
intron_variant	102,061	12.261%
non_coding_transcript_variant	184	0.022%
splice_acceptor_variant	177	0.021%
splice_donor_variant	240	0.029%
splice_region_variant	2,375	0.285%
start_lost	93	0.011%
start_retained_variant	12	0.001%
stop_gained	170	0.02%
stop_lost	70	0.008%
stop_retained_variant	13	0.002%
upstream_gene_variant	215,457	25.885%

Region

Type (alphabetical order)	Count	Percent
DOWNSTREAM	194,273	23.425%
EXON	7,016	0.846%
GENE	4	0%
INTERGENIC	296,065	35.699%
INTRON	99,963	12.053%
SPLICE_SITE_ACCEPTOR	149	0.018%
SPLICE_SITE_DONOR	195	0.024%
SPLICE_SITE_REGION	1,949	0.235%
TRANSCRIPT	201	0.024%
UPSTREAM	215,457	25.979%
UTR_3_PRIME	8,824	1.064%
UTR_5_PRIME	5,241	0.632%

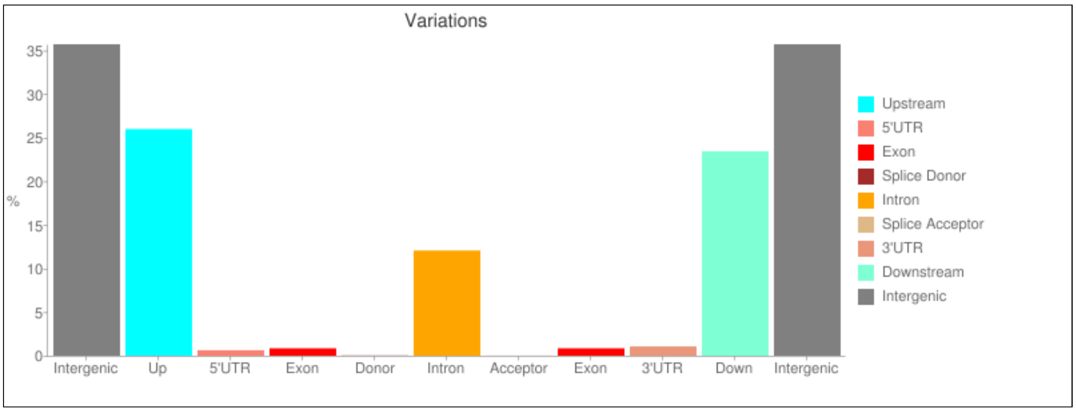

Quality:

**Min**	10
**Max**	28,267
**Mean**	114.261
**Median**	85
**Standard deviation**	203.811
**Values**	10,11,12,13,14,15,16,17,18,19,20,21,22,23,24,25,26,27,28,29,30,31,32,33,34,35,36,37,38,39,40,41,42,43,44,45,46,47,48,49,50,51,52,53,54,55,56,57
**Count**	972,1188,1970,1280,1397,1448,1332,1322,1562,1465,1389,1491,2333,1302,1367,2910,1405,1446,3880,1416,1573,5806,1845,1465,7075,1267,136[illegible]

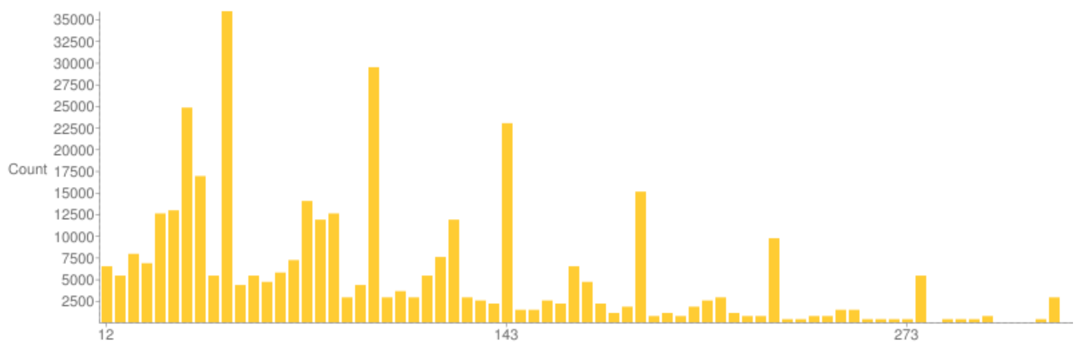

Insertions and deletions length:

**Min**	0
**Max**	214
**Mean**	2.164
**Median**	1
**Standard deviation**	5.704
**Values**	0,1,2,3,4,5,6,7,8,9,10,11,12,13,14,15,16,17,18,19,20,21,22,23,24,25,26,27,28,29,30,31,32,33,34,35,36,37,38,39,40,41,42,43,44,45,46,47,48,49,50,51
**Count**	95070,241369,14790,10042,5779,4636,3150,3046,2794,2556,2148,2233,1597,1354,1198,1043,807,890,728,681,667,551,465,444,401,364,361,326,[illegible]

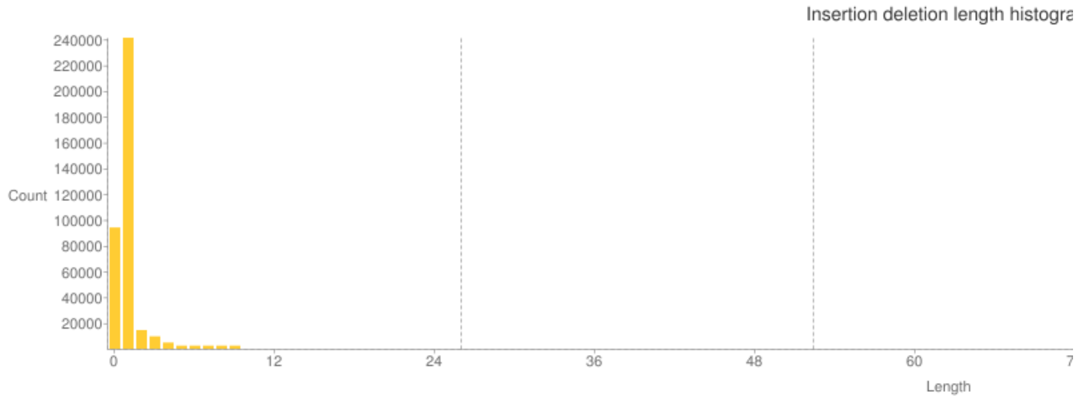

Base changes (SNPs)

	A	C	G	T
**A**	0	0	0	0
**C**	0	0	0	0
**G**	0	0	0	0
**T**	0	0	0	0

Ts/Tv (transitions / transversions)

Note: Only SNPs are used for this statistic.
Note: This Ts/Tv ratio is a 'raw' ratio (ratio of observed events).

**Transitions**	0
**Transversions**	0
**Ts/Tv ratio**	0

All variants:

```
No results available (empty input?)
```

Only known variants (i.e. the ones having a non-empty ID field):

```
No results available (empty input?)
```

Allele frequency

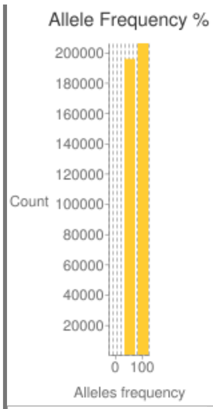

**Min**	0
**Max**	100
**Mean**	75.517
**Median**	100
**Standard deviation**	25.06
**Values**	0,50,100
**Count**	265,196865,205996

Allele Count

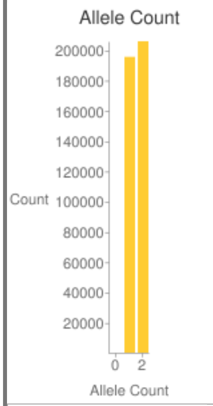

**Min**	0
**Max**	2
**Mean**	1.51
**Median**	2
**Standard deviation**	0.501
**Values**	0,1,2
**Count**	265,196865,205996

Hom/Het per sample

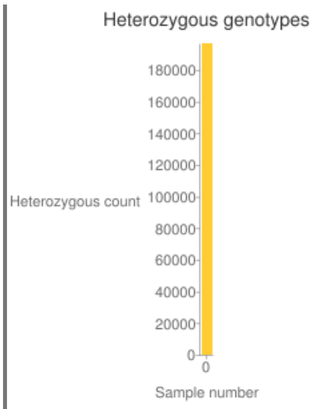

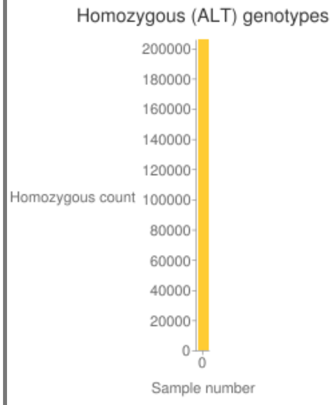

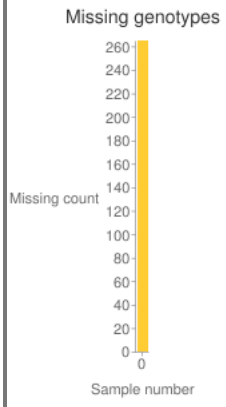


```
Sample_names , readname
Reference , 0
Het , 196865
Hom , 205996
Missing , 265
```

Codon changes

How to read this table:
- Rows are reference codons and columns are changed codons. E.g. Row 'AAA' column 'TAA' indicates how many 'AAA' codons have been replaced by 'TAA' codons.
- Red background colors indicate that more changes happened (heat-map).
- Diagonals are indicated using grey background color
- WARNING: This table may include different translation codon tables (e.g. mamalian DNA and mitochondrial DNA).

	-	AAA	AAC	AAG	AAT	ACA	ACC	ACG	ACT	AGA	AGC	AGG	AGT	ATA	ATC	ATG	ATT	CAA	CAC	CAG	CAT	CCA	CC
**-**		151	73	125	127	98	41	33	103	73	53	52	85	59	49	92	89	130	56	99	91	144	4
**AAA**	222	**24**	5	35	19	3	1	3	3	14	1		1	3	2	4	1			2	1		
**AAC**	141	22	**7**	13	19	3	3	4	4	2	4	2	1	1		1	2	2		1			
**AAG**	264	34	11	**22**	12	4	3	1	1	6	3	6	1	2	1	4	5	1					
**AAT**	231	35	12	11	**11**	3	1		7	9	3		1	3	2	4	4	3					
**ACA**	133	2	9	2	2	**5**	10	5	10	1	3	1	1			1	2	1					
**ACC**	80	1	3	2	3	5	**3**	3	6	1	1		1	2	3		1		1				
**ACG**	41		2	1		4	3	**2**	3			1		1		2							
**ACT**	135	4	7	2	1	10	6	8	**10**	2	1	2	5	1	3	1	2		2				
**AGA**	145	2	1	9	1	2		1	1	**6**	3	12	3	1		1	1	1					
**AGC**	88	1	7	7	1	1	1	1		6	**7**	1	4	1									
**AGG**	97	2	3	6	2	1	2		2	17	1	**8**	3			2				2	1		
**AGT**	114	3	2	3	6	3	2	2	6	5	6	6	**11**	2	1	1	1	1					
**ATA**	91	1		4	6	3		1	1			1	1	**7**	5	3	13				1		
**ATC**	73		1	1	3	3	2				1		2	6	**1**	4	3						
**ATG**	201	7		3	28	1	2		3	3	2	2	1	10	2	**11**	10	1			1		
**ATT**	154		3	2	10	2	3	2	3	4		1	2	14	9	11	**13**					1	

	-	AAA	AAC	AAG	AAT	ACA	ACC	ACG	ACT	AGA	AGC	AGG	AGT	ATA	ATC	ATG	ATT	CAA	CAC	CAG	CAT	CCA	CC[illegible]
**CAA**	169					1	2											**17**	3	21	12	5	
**CAC**	69																	7	**1**	4	17	3	
**CAG**	131		1	1														13	5	**8**	2	2	
**CAT**	124				1	1												12	6	11	**11**	6	
**CCA**	173			2			1											1	2	5	2	**7**	1
**CCC**	71				1							1						1	2			8	**7**
**CCG**	75			1														1			1	9	
**CCT**	184						1			1						1		2		2	3	15	1
**CGA**	28					1		1		1								1	1		2	1	
**CGC**	27																		1	1	1		
**CGG**	30																			2	2		
**CGT**	44																	3	1		2	1	
**CTA**	75								1											2	1	1	
**CTC**	85						1		1						2				2	2	2		
**CTG**	71		1						2									2		1	2	2	
**CTT**	150								3									3	1	2	2	1	
**GAA**	302									2			1									1	
**GAC**	114		2	3						1		1							1				
**GAG**	258	1		3				2		2												1	
**GAT**	284		1		3				1				1							1	2		
**GCA**	189					1					2		1			1				2			
**GCC**	83		4	1			2				1					1	1						
**GCG**	49			3																			
**GCT**	200								1		3					1				1			
**GGA**	176							1				1				1							
**GGC**	91											3								1			
**GGG**	100			1								1											
**GGT**	193			1													1						
**GTA**	95												1										
**GTC**	65			1								1								1			
**GTG**	113			1		2							1			1							
**GTT**	175									1			2								1		
**TAA**	22																						
**TAC**	78				1																		
**TAG**	12																						
**TAT**	118													3									
**TCA**	202	1													3					1			
**TCC**	89																			1	1	1	
**TCG**	53														2								
**TCT**	260	1			1		1				1				4						1	1	
**TGA**	21			1																			
**TGC**	60					1							1			1							
**TGG**	69				1							1				2							
**TGT**	95	1			1					1								1			2		
**TTA**	130		1		2				1								2				1		
**TTC**	136		1		1					1				1			3	1			1		
**TTG**	148				1				1						1		1						
**TTT**	204				1												5						

Amino acid changes

How to read this table:
- Rows are reference amino acids and columns are changed amino acids. E.g. Row 'A' column 'E' indicates how many 'A' amino acids have been replaced by 'E' amino acids.
- Red background colors indicate that more changes happened (heat-map).
- Diagonals are indicated using grey background color
- WARNING: This table may include different translation codon tables (e.g. mamalian DNA and mitochondrial DNA).

	*	-	?	A	C	D	E	F	G	H	I	K	L	M	N	P	Q	R	S	T	V	W	Y
*	**6**	54	1		1	1	1	1				1	7					1	2		1	1	8
-	130		1,970	232	79	204	293	207	258	147	197	276	360	92	200	313	229	209	524	275	242	50	102
?																							
A	2	521		**126**	5	15	15	1	43		1	4	4	3	4	3	3		11	4	12		
C	6	155			**14**	1		5		2		1	13	1	1	1	1	2	11	1		5	4
D	3	398		20	1	**50**	61	1	37	3		3	1		6		1	2	1	1	8		2
E	7	560		17		56	**104**	1	45			4	1			2		7	1	2	24	2	2
F	7	340		3	5	2	2	**61**		1	9		36		3	1	1	2	18		5	4	14
G		560		20	2	21	46		**146**		1	2	5	1		2	1	5	2	1	30	4	2
H		193				2			3	**35**			13		1	14	34	8	4	1			
I	1	318			1	3			3	1	**71**	8	1	18	23	2		6	10	20	1	1	6
K	9	486		2		2	11		3	1	14	**115**	4	8	47		3	28	7	19	2		
L	3	659		9	6		2	70	3	11	6		**135**		5	25	12	7	26	10	5	2	8
M		201		1		2				1	22	10		**11**	28		1	6	4	6			3
N	6	371	1	3		1	3	1	4		12	81	4	5	**49**		6	15	11	25			

	*	-	?	A	C	D	E	F	G	H	I	K	L	M	N	P	Q	R	S	T	V	W	Y
**P**	1	503		8	1	1	1	2		10		3	24	1	1	**146**	12	11	7	2	4		1
**Q**		300		6		1	4	1	3	22		1	14		1	16	**59**	7	2	3			
**R**	6	371		3			3		4	11	2	19	16	3	7	10	10	**85**	13	11	1		1
**S**	10	806		4	15	1	4	48	3	2	14	16	24	1	17	9	3	18	**245**	17	4	2	12
**T**	1	389		2	1	4	3		1	3	15	14	5	4	27	2	1	8	14	**93**			3
**V**		448		18	4	22	15	3	24	1		2	3	1			1	3	7	2	**101**	1	
**W**	6	69			6			1	1				9	2	1			2	4			**6**	
**Y**	15	196			2		1	3	1		3		17		1			1	15		2	1	**24**

Variants by chromosome

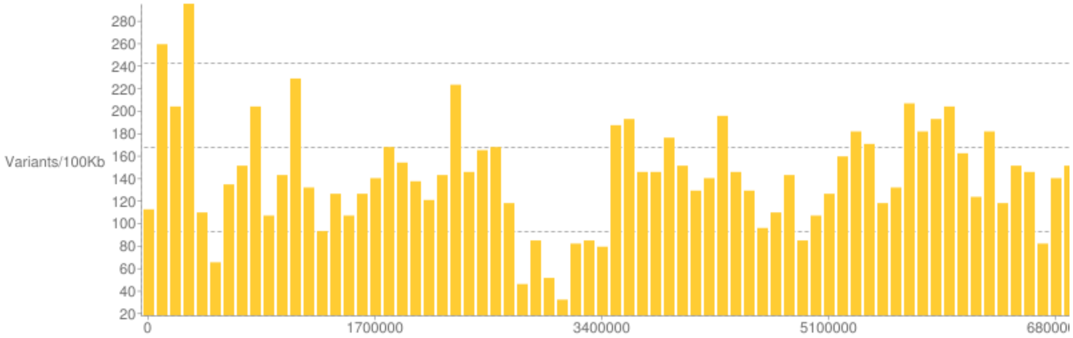

1, Position,0,100000,200000,300000,400000,500000,600000,700000,800000,900000,1000000,1100000,1200000,1300000,1400000,
1,Count,113,260,205,295,111,67,137,151,206,107,144,229,134,95,128,108,128,140,168,155,138,121,145,223,146,165,168,120,48,85,52,34,82,

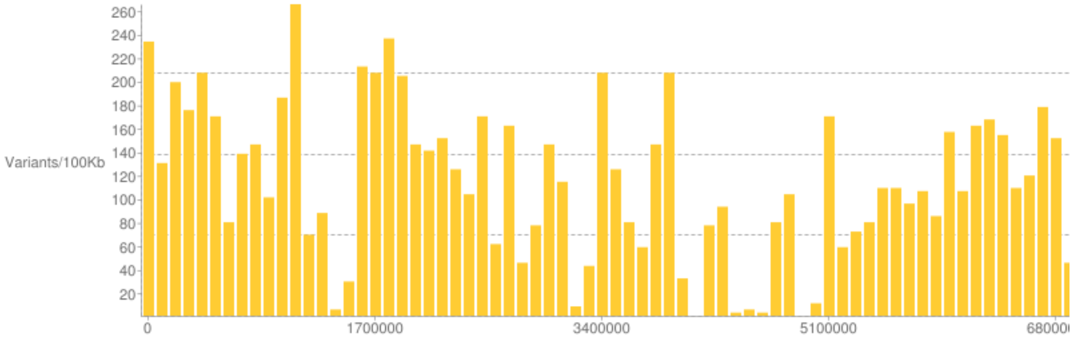

2, Position,0,100000,200000,300000,400000,500000,600000,700000,800000,900000,1000000,1100000,1200000,1300000,1400000,
2,Count,236,133,202,176,208,173,83,141,149,104,188,266,72,89,7,32,215,208,238,207,149,144,153,127,106,172,63,165,48,80,149,115,11,46,

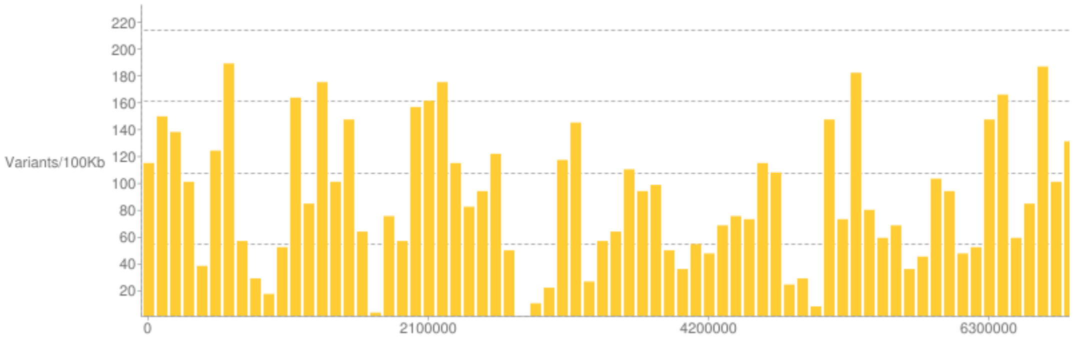

3, Position,0,100000,200000,300000,400000,500000,600000,700000,800000,900000,1000000,1100000,1200000,1300000,1400000,
3,Count,115,150,139,102,39,126,189,57,31,19,54,164,85,176,101,148,65,5,76,58,157,163,175,116,83,94,123,50,3,11,22,117,145,27,58,65,11

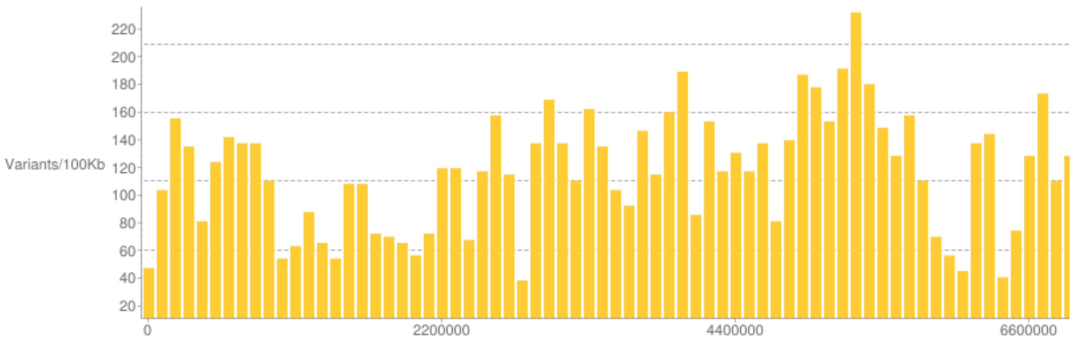

4, Position,0,100000,200000,300000,400000,500000,600000,700000,800000,900000,1000000,1100000,1200000,1300000,1400000,
4,Count,47,104,157,136,81,124,143,137,137,110,54,64,89,65,55,108,109,73,70,65,57,73,119,119,68,117,159,115,40,139,169,138,111,162,136,

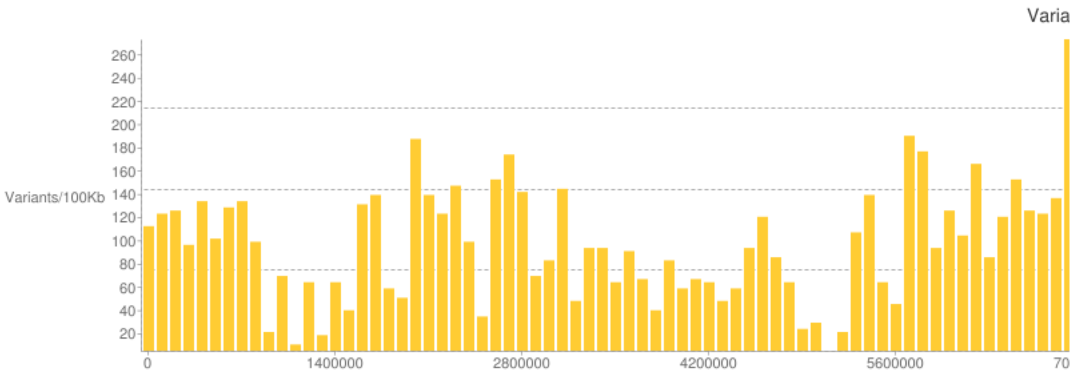

5, Position,0,100000,200000,300000,400000,500000,600000,700000,800000,900000,1000000,1100000,1200000,1300000,1400000,
5,Count,114,124,127,98,135,102,129,136,99,23,70,13,64,19,64,41,132,140,59,51,188,140,123,148,99,37,153,174,143,71,83,145,48,96,94,66,

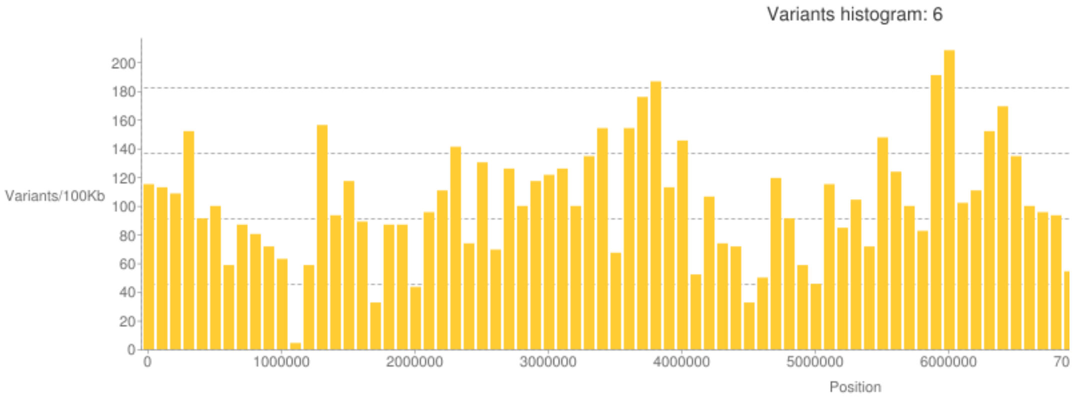

6, Position,0,100000,200000,300000,400000,500000,600000,700000,800000,900000,1000000,1100000,1200000,1300000,1400000,
6,Count,117,115,109,154,92,100,59,88,82,72,65,6,60,158,94,118,90,34,87,88,44,97,111,143,74,132,70,127,100,118,122,126,101,135,156,69,

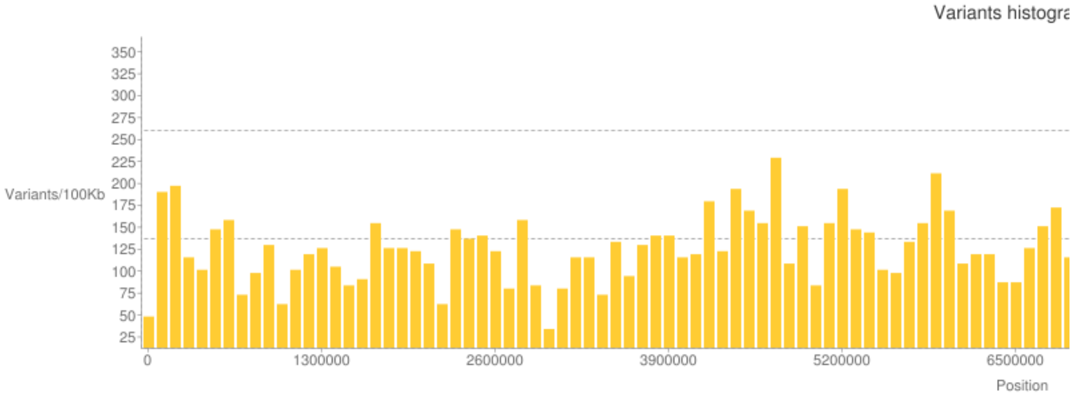

7, Position,0,100000,200000,300000,400000,500000,600000,700000,800000,900000,1000000,1100000,1200000,1300000,1400000,
7,Count,50,192,198,117,103,148,158,75,98,131,62,104,120,126,105,85,93,157,128,126,125,109,63,147,137,141,123,80,159,85,36,81,116,118,

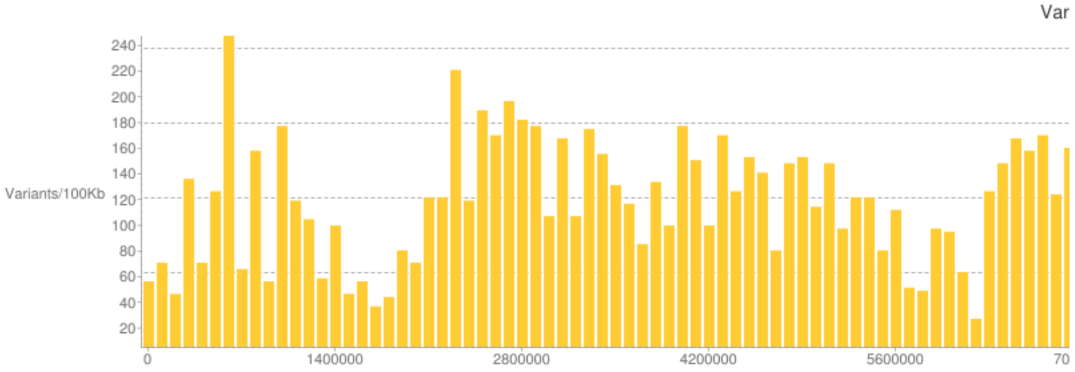

8, Position,0,100000,200000,300000,400000,500000,600000,700000,800000,900000,1000000,1100000,1200000,1300000,1400000,
8,Count,57,71,47,137,71,126,247,66,158,56,179,119,105,59,100,48,56,38,46,82,71,122,122,221,120,189,171,198,184,178,109,168,109,176,

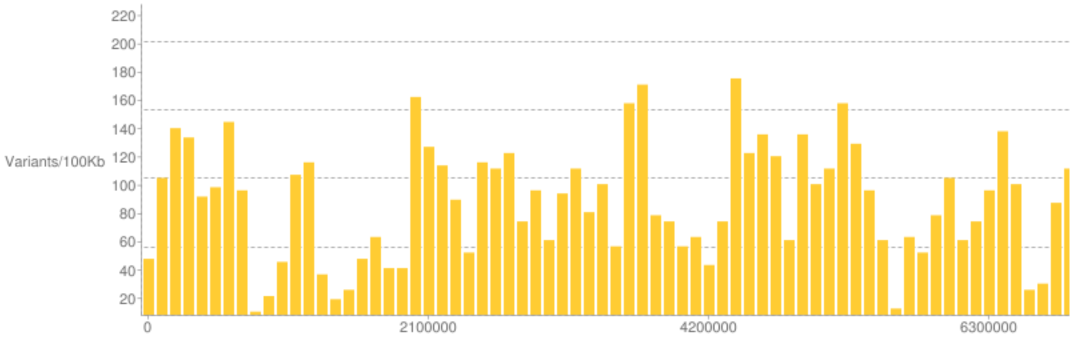

9, Position,0,100000,200000,300000,400000,500000,600000,700000,800000,900000,1000000,1100000,1200000,1300000,1400000,
9,Count,48,106,141,135,92,100,145,97,12,23,47,107,116,38,21,26,48,65,43,41,163,127,114,90,52,116,112,124,76,97,62,95,113,81,101,58,

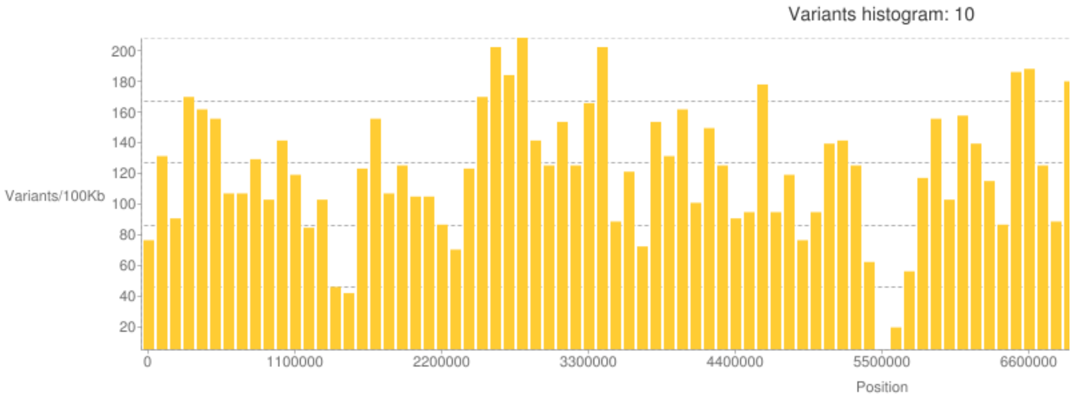

10, Position,0,100000,200000,300000,400000,500000,600000,700000,800000,900000,1000000,1100000,1200000,1300000,1400000,
10,Count,77,132,91,170,163,157,108,107,129,103,142,119,85,104,46,42,124,157,107,125,105,106,88,71,123,170,203,185,208,142,126,154,125,

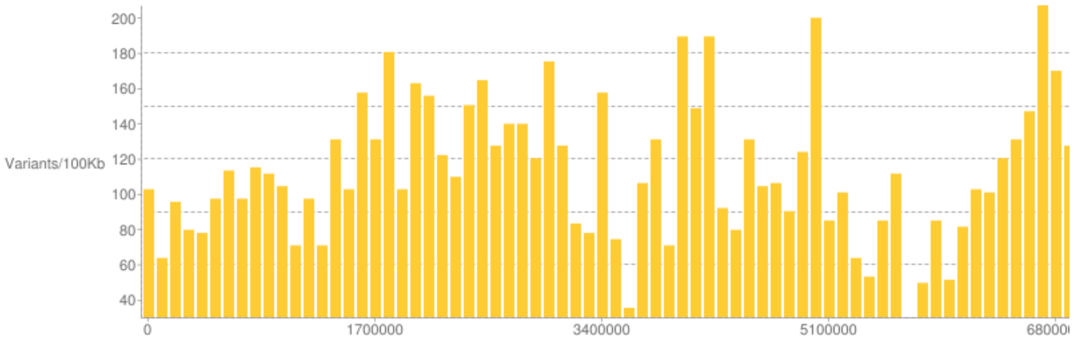

11, Position,0,100000,200000,300000,400000,500000,600000,700000,800000,900000,1000000,1100000,1200000,1300000,1400000,
11,Count,103,65,97,81,78,99,114,99,115,113,106,72,99,72,132,103,158,131,182,104,164,157,123,110,152,166,129,140,140,122,176,129,84,79,

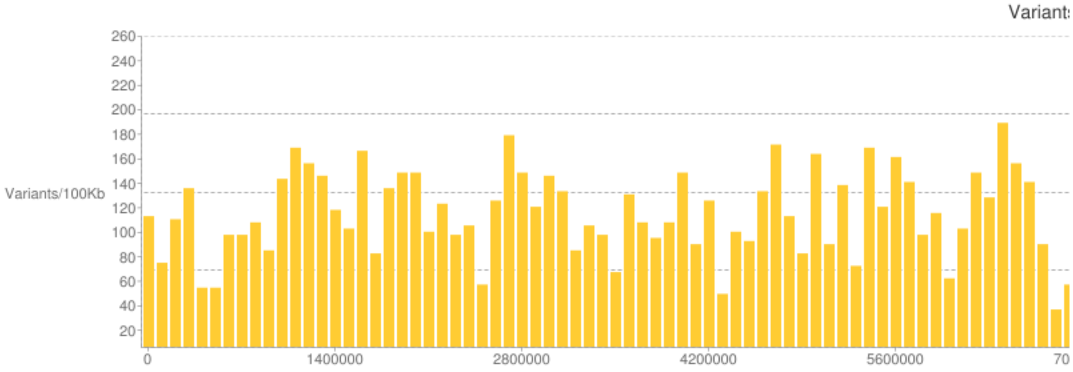

12, Position,0,100000,200000,300000,400000,500000,600000,700000,800000,900000,1000000,1100000,1200000,1300000,1400000,
12,Count,113,76,111,136,56,55,99,99,108,85,144,171,156,147,119,105,167,84,136,150,149,102,124,99,106,57,126,179,150,122,147,133,87,10

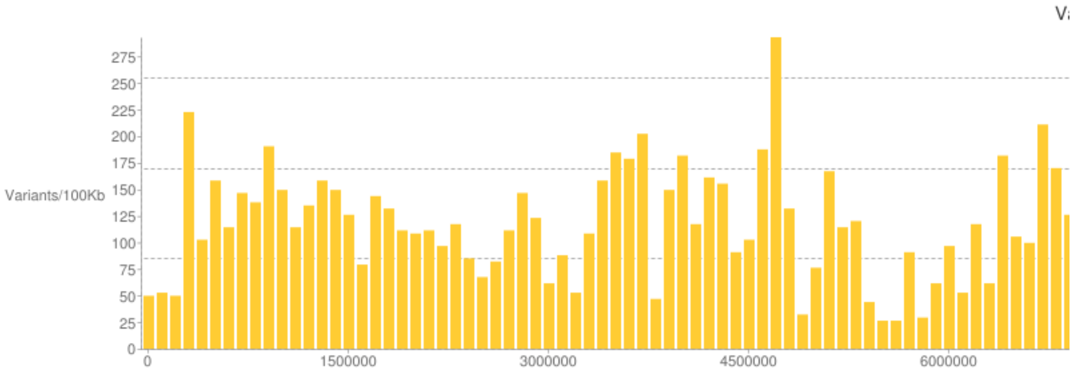

13, Position,0,100000,200000,300000,400000,500000,600000,700000,800000,900000,1000000,1100000,1200000,1300000,1400000,
13,Count,51,55,51,223,104,160,117,147,140,192,151,116,136,161,151,126,80,146,134,113,111,113,97,119,86,69,83,114,147,125,63,89,54,111,

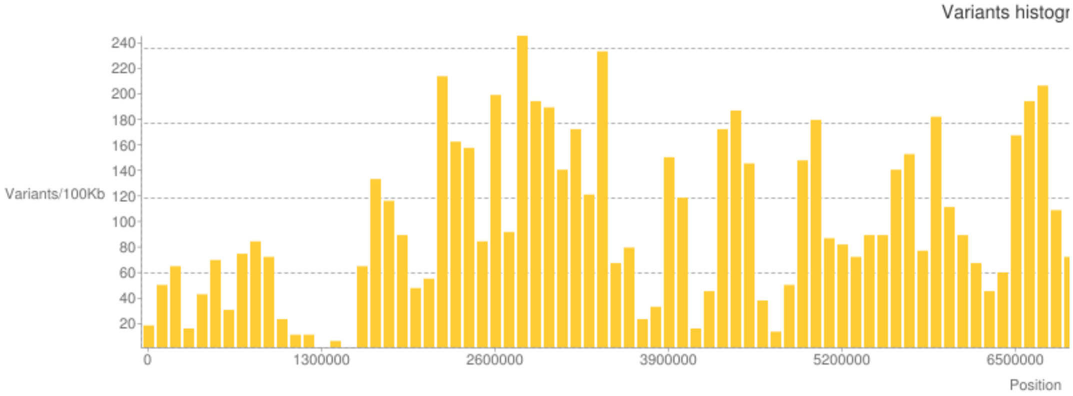

14, Position,0,100000,200000,300000,400000,500000,600000,700000,800000,900000,1000000,1100000,1200000,1300000,1400000,
14,Count,20,50,65,16,43,70,31,75,86,73,25,11,12,3,6,1,65,133,117,91,49,56,214,163,158,85,201,92,245,196,191,141,172,121,233,68,80,25,

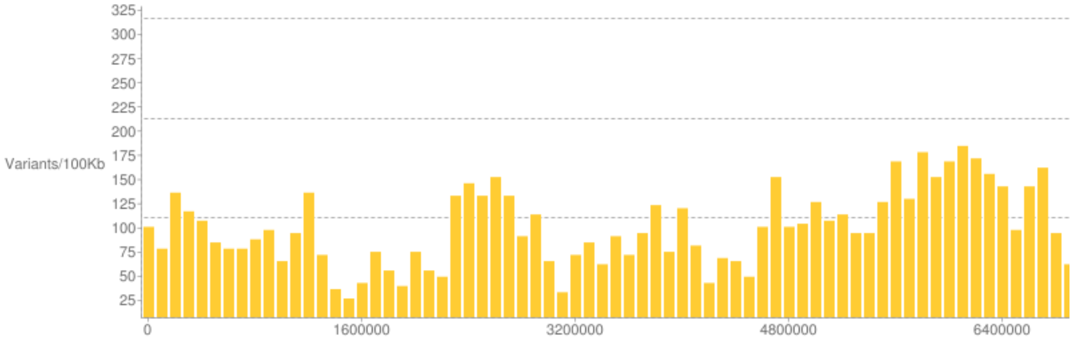

15, Position,0,100000,200000,300000,400000,500000,600000,700000,800000,900000,1000000,1100000,1200000,1300000,1400000,
15,Count,102,78,137,117,109,87,81,79,88,98,68,95,138,73,36,28,45,75,58,40,75,57,51,134,147,135,153,134,92,115,68,33,74,86,62,93,74,97,

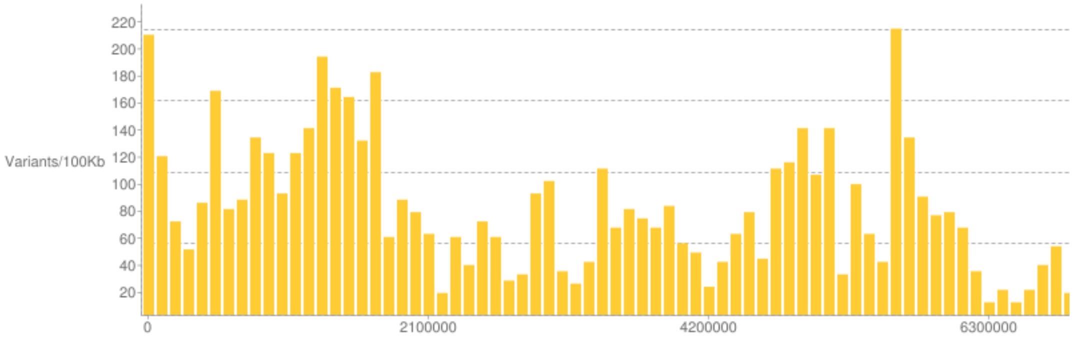

16, Position,0,100000,200000,300000,400000,500000,600000,700000,800000,900000,1000000,1100000,1200000,1300000,1400000,
16,Count,212,122,73,52,86,169,83,90,136,123,93,124,143,195,171,166,134,184,62,89,81,63,20,61,40,74,62,29,33,94,104,37,27,44,112,68,83,

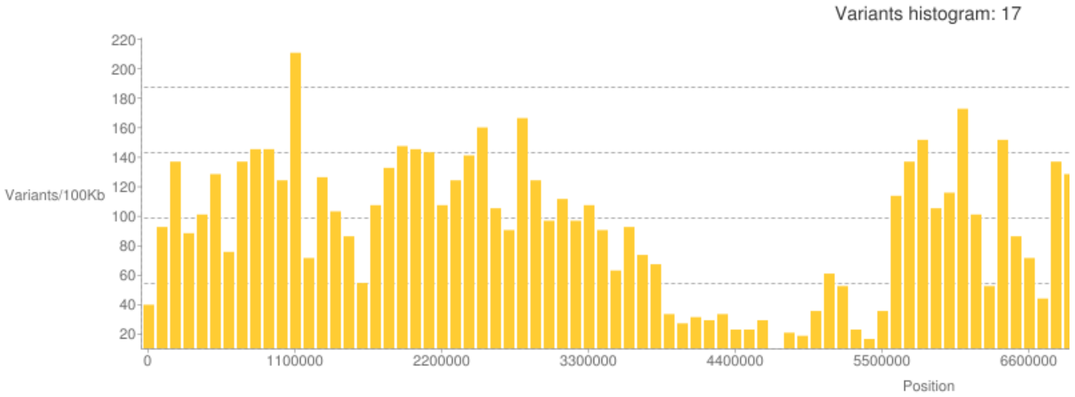

17, Position,0,100000,200000,300000,400000,500000,600000,700000,800000,900000,1000000,1100000,1200000,1300000,1400000,
17,Count,41,94,138,89,102,130,77,137,146,147,124,211,72,128,103,88,55,109,134,148,146,143,108,126,142,160,106,91,168,125,97,113,98,10[illegible]

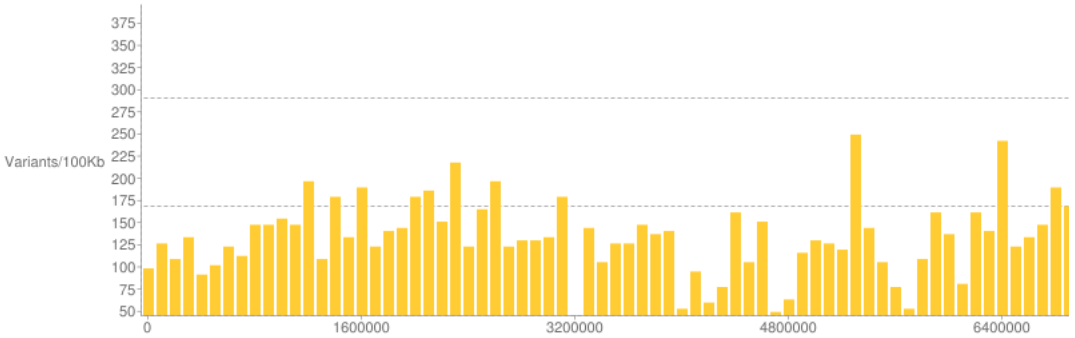

18, Position,0,100000,200000,300000,400000,500000,600000,700000,800000,900000,1000000,1100000,1200000,1300000,1400000,
18,Count,99,128,111,135,93,103,124,114,149,148,155,150,199,109,181,135,190,123,140,145,180,188,153,220,123,165,199,123,132,132,135,17[illegible]

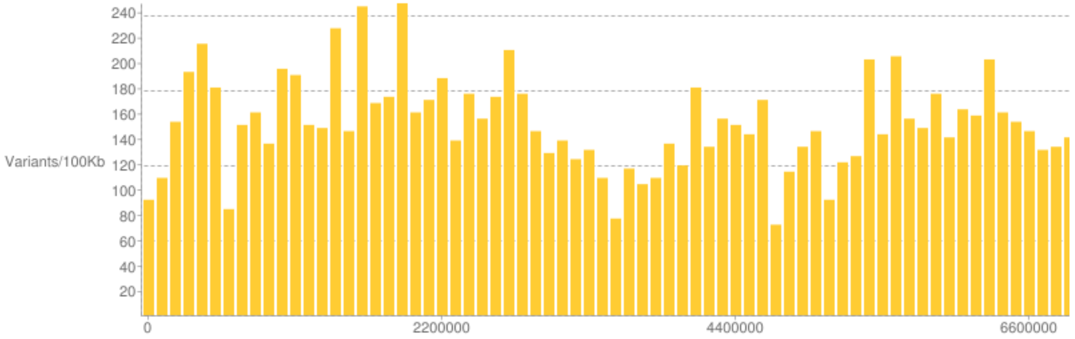

19, Position,0,100000,200000,300000,400000,500000,600000,700000,800000,900000,1000000,1100000,1200000,1300000,1400000,
19,Count,94,111,154,193,217,182,85,153,161,137,196,191,153,150,228,148,245,170,174,247,161,171,189,139,177,157,175,212,177,148,131,14[illegible]

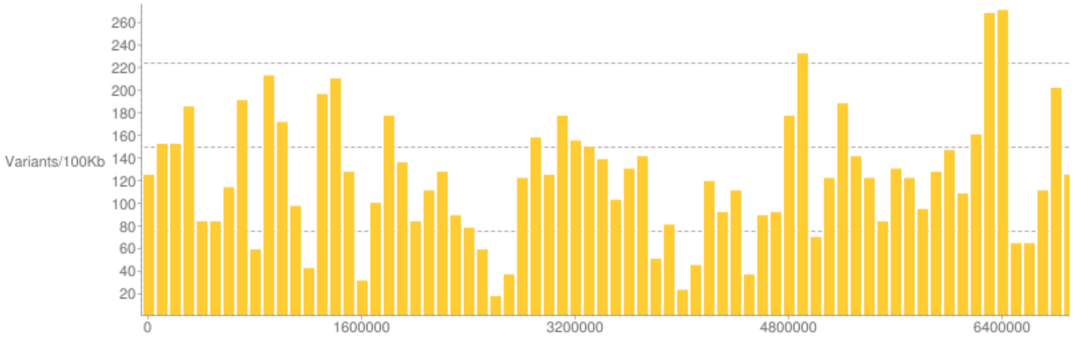

20, Position,0,100000,200000,300000,400000,500000,600000,700000,800000,900000,1000000,1100000,1200000,1300000,1400000,
20,Count,125,154,154,187,85,84,115,192,61,214,172,98,44,197,211,128,32,102,178,136,85,111,128,89,80,61,18,38,122,158,126,177,155,152,

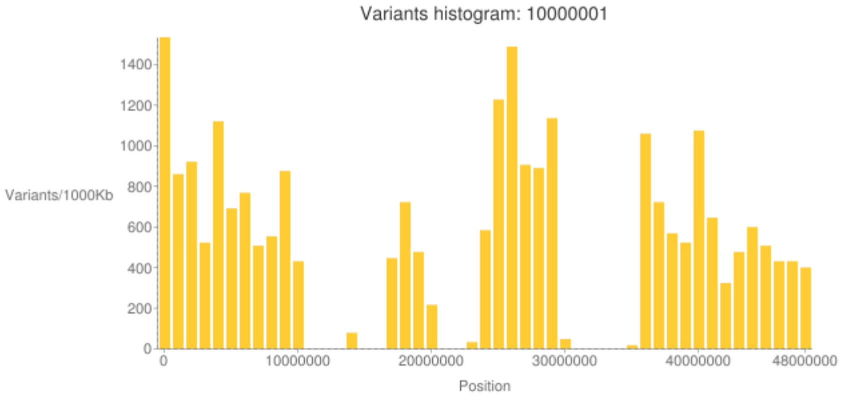

10000001, Position,0,1000000,2000000,3000000,4000000,5000000,6000000,7000000,8000000,9000000,10000000,11000000,1200000
10000001,Count,1531,859,920,532,1130,699,768,511,554,880,431,0,0,0,90,0,0,459,734,487,229,0,0,42,584,1225,1486,917,903,1143,47,0,0,0,

Details by gene

Here you can find a tab-separated table.
