# Supplementary material for: Genome-wide SNP and InDel analysis of three Philippine mango species inferred from whole-genome sequencing
Source: J Genet Eng Biotechnol. 2022 Mar 11;20:46. doi: 10.1186/s43141-022-00326-3 (PMC8917249; doi:10.1186/s43141-022-00326-3)
Supplement: Supplementary file 7 — Additional file 7: Supplemental Table 1. Breakdown of genes with high impact variants. [file 43141_2022_326_MOESM7_ESM.docx]

**Supplemental Table 1. Breakdown of functionally annotated genes with high impact variants.**

| **Reference genome** | **Species** | **Number of high impact SNP/InDel** | **Number of genes with high impact variants** | **With BLAST + Annotation** | **With BLAST only** | **No BLAST result (novel)** |
| --- | --- | --- | --- | --- | --- | --- |
| Alphonso | *M. altissima* InDel | 7,087 | 4,310 | 3,059 | 5 | 1,246 |
|  | *M. altissima* SNP | 3,340 | 2,582 | 1,726 | 1 | 855 |
|  | *M. indica* InDel | 6,364 | 3,987 | 2,814 | 5 | 1,168 |
|  | *M. indica* SNP | 2,918 | 2,249 | 1,473 | 3 | 773 |
|  | *M. odorata* InDel | 7,963 | 4,802 | 3,381 | 2 | 1,419 |
|  | *M. odorata* SNP | 4,583 | 3,363 | 2,171 | 4 | 1,188 |
| Tommy Atkins | *M. altissima* InDel | 5,301 | 3,200 | 2,672 | 5 | 523 |
|  | *M. altissima* SNP | 2,935 | 2,309 | 1,918 | 4 | 387 |
|  | *M. indica* InDel | 4,780 | 2,913 | 2,421 | 3 | 489 |
|  | *M. indica* SNP | 2,335 | 1,874 | 1,563 | 2 | 309 |
|  | *M. odorata* InDel | 5,812 | 3,419 | 2,833 | 5 | 581 |
|  | *M. odorata* SNP | 3,564 | 2,738 | 2,262 | 5 | 471 |
| Total | | 56,982 | 37,746 | 28,293 | 44 | 9,409 |
