# Supplementary material for: Genome-wide SNP and InDel analysis of three Philippine mango species inferred from whole-genome sequencing
Source: J Genet Eng Biotechnol. 2022 Mar 11;20:46. doi: 10.1186/s43141-022-00326-3 (PMC8917249; doi:10.1186/s43141-022-00326-3)
Supplement: Supplementary file 8 — Additional file 8: Supplemental Table 2. Alleles observed in the mango species. [file 43141_2022_326_MOESM8_ESM.docx]

**Supplemental Table 2. Alleles observed in the mango species.**

| **Species** | **Allele** | | **Total heterozygous allele** | **Total missing allele** | **Total polymorphic allele** |
| --- | --- | --- | --- | --- | --- |
|  | **Alphonso** | **Tommy Atkins** |  |  |  |
| *M. odorata* | 1,486,454 | 1,524,426 | 5,583,939 | 6,805 | 3,004,075 |
| *M. altissima* | 1,332,139 | 1,397,863 | 4,088,336 | 4,634 | 2,725,368 |
| *M. indica* | 1,056,497 | 1,175,803 | 3,356,816 | 7,449 | 2,224,851 |
