# Supplementary material for: Genome-wide SNP and InDel analysis of three Philippine mango species inferred from whole-genome sequencing
Source: J Genet Eng Biotechnol. 2022 Mar 11;20:46. doi: 10.1186/s43141-022-00326-3 (PMC8917249; doi:10.1186/s43141-022-00326-3)
Supplement: Supplementary file 9 — Additional file 9: Supplemental Table 3. Kinship analysis of mango species. [file 43141_2022_326_MOESM9_ESM.docx]

| **Species** | ***M. indica* ‘Carabao’** | ***M. altissima*** | ***M. odorata*** |
| --- | --- | --- | --- |
| ***M. indica* ‘Carabao’** | 2 |  |  |
| ***M. altissima*** | 1.531 | 2 |  |
| ***M. odorata*** | 1.594 | 1.589 | 2 |

**Supplemental Table 3.** Kinship analysis between the three Philippine mango species via identity-by-state (IBS) method.
